# Measuring Kinetics under Vibrational Strong Coupling: Testing for a Change in the Nucleophilicity of Water and Alcohols

**DOI:** 10.1002/anie.202410770

**Published:** 2024-10-24

**Authors:** Cyprien Muller, Robert J. Mayer, Maciej Piejko, Bianca Patrahau, Valentin Bauer, Joseph Moran

**Affiliations:** ^1^ University of Strasbourg CNRS, ISIS UMR 7006 67000 Strasbourg France; ^2^ School of Natural Sciences Department Chemie Technical University Munich (TUM) 85748 Garching Germany; ^3^ Department of Chemistry and Biomolecular Sciences University of Ottawa Ottawa Ontario K1N 6N5 Canada

**Keywords:** nucleophilicity, vibrational strong coupling, alcohols, benzhydrilium, kinetics

## Abstract

Vibrational Strong Coupling (VSC) has been reported to change the rate of organic reactions. However, a lack of convenient and reliable methods to measure reaction kinetics under VSC makes it challenging to obtain mechanistic insight into its influence, hindering progress in the field. Here, we use recently developed fixed‐width optical cavities to obtain large kinetic datasets under VSC with small errors (±1–5 %) in an operationally simple manner using UV/Vis spectroscopy. The setup is used to test whether VSC changes a fundamental kinetic property of polar reactions, nucleophilicity, for water and alcohols, species commonly used in VSC‐modified chemistry. We determined the rate constants for nucleophilic capture with a library of benzhydrilium ions as reference electrophiles with and without strong coupling of the nucleophile's key vibrations. For all investigated combinations of electrophiles and nucleophiles, only minor changes in the observed rate constants of the reactions were observed independently of the coupled bands. These results indicate that VSC does not substantially alter the nucleophilicity of water and alcohols, suggesting that polar reactions are modified through other, presently unknown mechanisms. Fixed‐width cavities allow for convenient and reproducible UV/Vis kinetics, facilitating mechanistic studies of VSC‐modified chemistry.

## Graphical abstract

Vibrational Strong Coupling (VSC) is reported to modify chemical reactivity, but its mode of action is not understood. Here, we provide a methodology using fixed‐width optical cavities for measuring kinetics under VSC in a reproducible way. The large kinetic dataset showed that VSC does not substantially alter the nucleophilicity of alcohols.

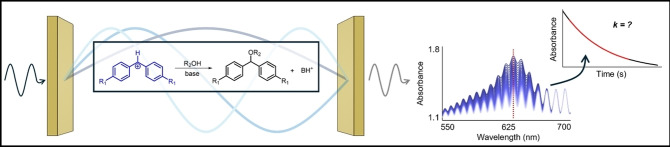

## Introduction

**Figure 1:**
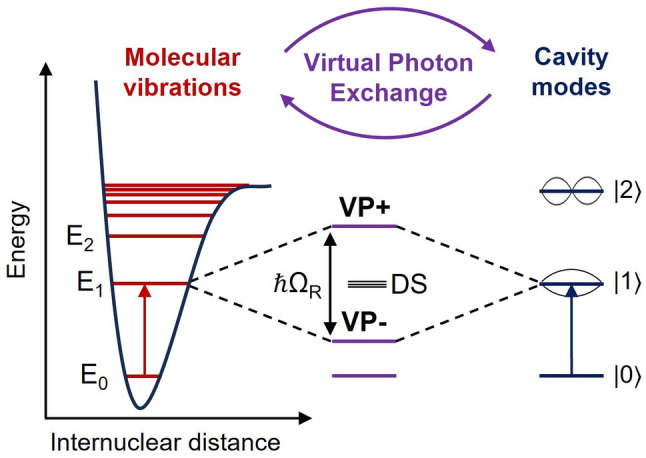
Schematic diagram of the formation of upper (P+) and lower (P−) vibro‐polaritons and degenerate dark states (DS) from resonant vibrational and optical transitions. Figure adapted from ref. [45].

At all times, vacuum fluctuations occur; that is, the amount of energy contained at a given point in any space varies randomly, akin to energy packets appearing and dissipating. In a confined space delimited by two reflective surfaces (later referred to as a “Fabry‐Perot cavity” or just a “cavity”), the restrained electromagnetic field forms discrete optical modes (Figure 1, right) whose energies vary as a function of the width of the cavity. Recently, it was reported that such modes can be tuned and coupled to molecular states,^[1]^ namely electronic and vibrational states (Figure 1, left),[^1^, ^2^, ^3^, ^4^, ^5^, ^6^] forming light‐matter hybrid states (Figure 1, center). In the case of molecular vibrations, such a phenomenon is called Vibrational Strong Coupling (VSC). The resulting hybrid states are referred to as vibropolaritonic states (VP+ and VP‐ in Figure 1), and the energy separating these states is called the Rabi splitting (*ħ*Ω_R_). When performing chemical reactions inside an optical cavity, VSC was shown for some specific systems to alter chemoselectivity,^[7]^ stereoselectivity,^[8]^ as well as reaction thermodynamics and kinetics.[^2^, ^7^, ^8^, ^9^, ^10^, ^11^] Importantly, chemical systems that were not affected by VSC were also reported recently, allowing to build a more complete image of cavity‐modified chemistry.[^12^, ^13^] However, experimental hints to the particular mechanisms through which VSC alters chemical reactivity still remain elusive, in contrast with the numerous theoretical studies on the topic.[^14^, ^15^, ^16^, ^17^, ^18^] Indeed, the complexity of the chemical systems that were studied, as well as the current analytical limitations under VSC, make extracting valuable conclusions complicated. For instance, the reliable measurement of reaction kinetics under VSC remains a challenge. To that end, the most common methods currently rely on (i) indirect monitoring through off‐resonance infrared modes,[^2^, ^7^, ^19^, ^20^] (ii) direct monitoring through spectral features of the starting material or product.[^11^, ^21^] In both cases, it has been pointed out that factors external to the reaction may contribute significantly, which may cause reproducibility issues and hinder the development of the field.[^22^, ^23^, ^24^, ^25^] These difficulties highlight the importance of developing new, more reliable ways to measure kinetics under VSC.

A common point among previous VSC studies is that most of the reactions surveyed follow polar organic mechanisms, thus involving electrophilic and nucleophilic species. Furthermore, alcohols and water were frequently used in these studies as solvents or nucleophiles.[^2^, ^7^, ^9^, ^11^, ^19^, ^26^] Therefore, quantifying the nucleophilicity of common alcohols and water under VSC provides a direct entry point to evaluate which chemical parameters VSC may modify.

Nucleophilicity is a kinetic property quantified by the rate at which a nucleophile reacts with a reference electrophile. Currently, the most widely accepted method to quantify nucleophilicity relies on benzhydrylium ions as reference electrophiles.[^27^, ^28^] Benzhydrylium ions are advantageous reference compounds, as (i) they are intensely colored and yield colorless reaction products, which facilitates the analysis of their reactions by UV/Vis spectroscopy, (ii) their electrophilicity can be tuned over a very broad range by varying their substituents, and (iii) these substituents do not modify the sterics of the reaction center, eliminating steric effects.[^27^, ^28^] Based on systematic kinetic studies with benzhydrylium ions and structurally related electrophiles, Mayr and co‐workers have developed what, to date, marks the most extensive reactivity scale to quantify polar organic reactivity.[^29^, ^30^, ^31^]

To investigate the effect of VSC on nucleophilicity, we herein measured the rate constants of water and various alcohol nucleophiles toward a library of benzhydrilium ions (**E1**–**5**) with and without coupling the nucleophile's key vibrations inside Fabry‐Perot cavities. The use of fixed‐width cavities unlocked highly reproducible kinetics, which revealed that VSC does not substantially alter the nucleophilicity of water and the alcohols studied in this work.

## Results and Discussion

### Experimental Setup for Kinetic Studies under VSC

**Scheme 1:**
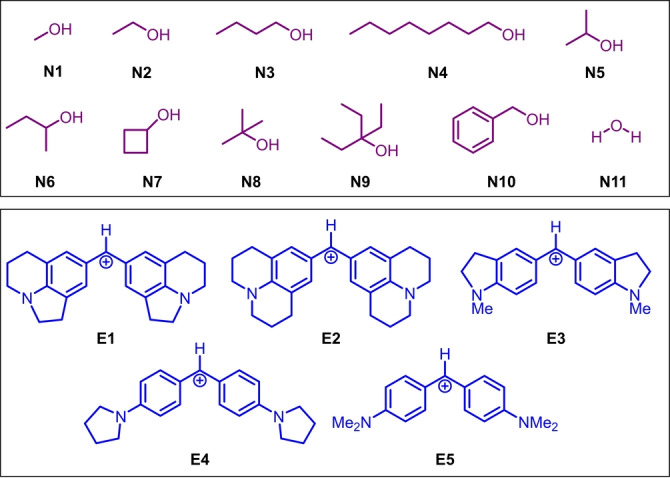
Nucleophiles **N** (top) and electrophiles **E** (bottom) investigated in this study (counterion=BF_4_^−^).

**Selection of Reaction Partners**. We started by choosing a set of alcohols **N** as nucleophiles that fulfilled some practical criteria for our studies (Scheme 1). The alcohols should have characteristic IR absorbances of high intensity to facilitate coupling in the Fabry‐Perot cavities. As electrophiles, we selected amino‐substituted benzhydrilium ions **E1**–**5**, as they were previously used in kinetic studies involving aqueous solvent mixtures and alcohols.[^32^, ^33^] The list of suitable reaction partners that allow for studies under VSC is shown in Scheme 1.

**Figure 2:**
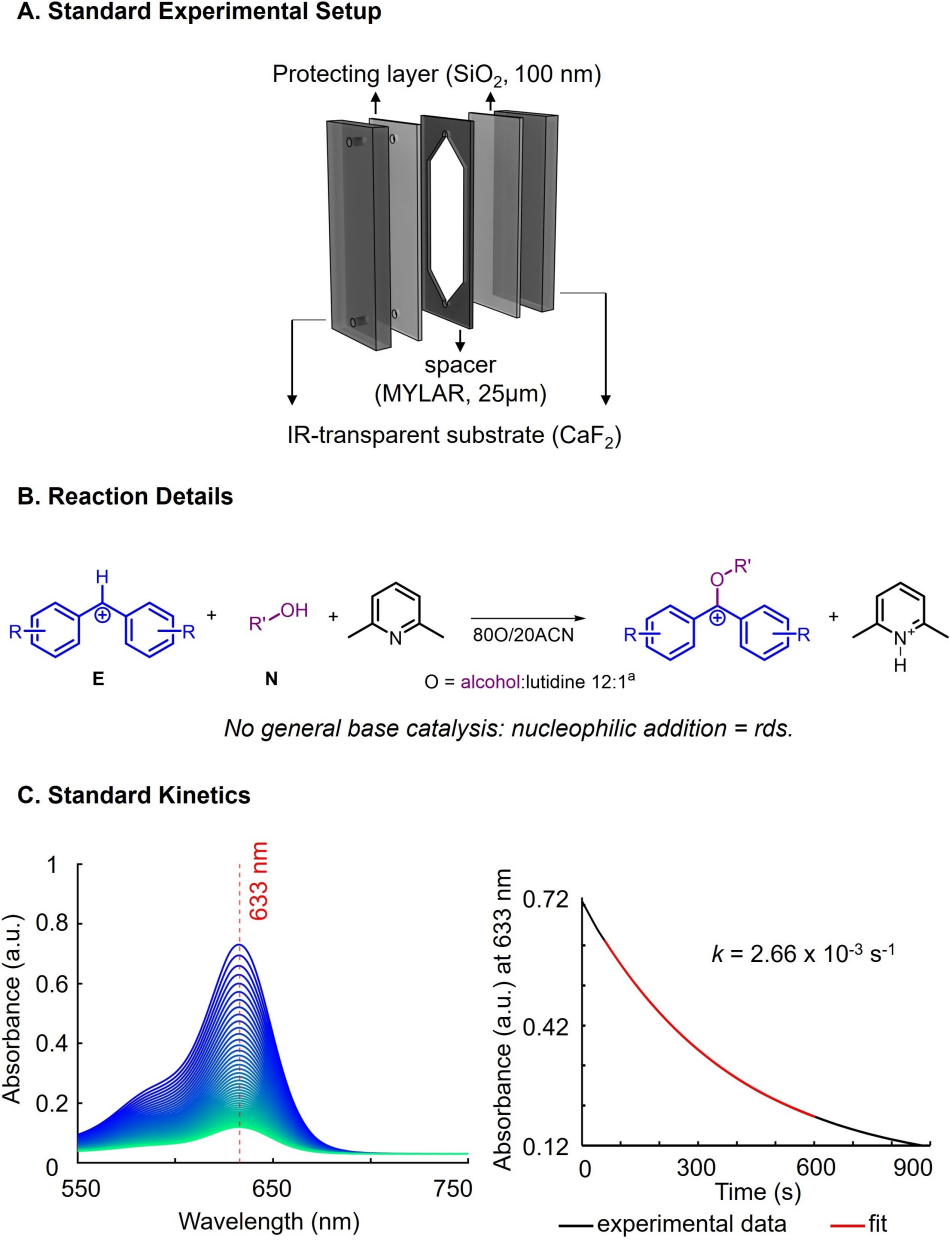
A. Schematic representation of our standard experimental setup. B. General reaction conditions, ^a^ for **N11**, 50 mM MOPS buffer solution was used instead of the alcohol:lutidine mixture at pH=7.0. C. Standard UV/Vis spectra resulting from the reaction of benzhydrilium ion **E1** (2.4 mM) with EtOH (**N2**) (left) and kinetic trace (black) with fit, which yielded a first‐order rate constant (red, right).

**Experimental Setup and Conditions**. We initially established a method for measuring standard kinetics that could serve as a reference for the envisioned kinetics studies under vibrational strong coupling. As was commonly done in the past, we opted to use IR‐transparent windows (CaF_2_) coated with a SiO_2_ layer and separated by a MYLAR spacer (Figure 2A).^[34]^ Within this space, the reaction mixture could be injected to measure kinetics.

Owing to the thickness of the MYLAR spacer (25 μm), the light pathlength in our experiments was considerably shorter than that of the typical quartz cells used by Mayr and co‐workers (5–10 mm) for the study of the reactions of nucleophiles with benzhydrilium ions. To still observe sufficient absorbance to obtain a reliable kinetic trace (i.e., A≈0.6 A.U.), we thus had to adapt the conditions typically used by Mayr and co‐workers. Given the extinction coefficients of **E** being in the range of 10^5^,^[27]^ kinetic measurements in standard quartz cuvettes (d=10 mm) are typically performed at concentrations of approx. 10^−5^ M to obtain absorbance values of around 1.[^28^, ^35^, ^36^] In contrast, we used significantly higher concentrations of **E** of approx. 2.4 mM.

Similarly to previous work by Mayr, the electrophiles **E1**–**5** were then dissolved in acetonitrile ‐ a non‐nucleophilic solvent ‐ to prepare stock solutions, thus implying the presence of acetonitrile in later kinetic experiments.

While the concentration of **E** is higher than usual, it is still too low for the electrophiles to be coupled directly, as VSC is highly concentration‐dependent and typically requires concentrations>1 M for coupling.

Thus, our study exclusively focuses on coupling the nucleophilic reaction partners: we envisioned that using the nucleophiles also as solvents ‐ and thus in high concentrations ‐ could facilitate the coupling of their characteristic IR bands. The large excess of the nucleophiles with respect to **E** further results in pseudo‐first‐order conditions, simplifying the analysis of the reaction kinetics.

To enable sufficient conversion in the kinetic measurements of the reaction of **E1**–**5** and alcohols **N**, a base or buffer is necessary to shift the equilibrium to the side of the products, as one equivalent of H^+^ is generated in the reaction. Here, we selected 2,6‐lutidine as a base for all organic nucleophiles because it does not react as a nucleophile with the selected electrophiles **E1**–**5**, as previously shown by Mayr and co‐workers.[^37^, ^38^, ^39^, ^40^] We found that with a 12 : 1 v/v ratio of nucleophile and base, respectively, consistent conversions>70 % could be observed. For the reactions involving water as the nucleophile, 50 mM 3‐(*N*‐morpholino)propanesulfonic acid (MOPS) at pH 7.0 was used as a buffer. All kinetic runs were performed at temperatures between 18 and 24 °C, and the room temperature was monitored and logged for each measurement. The general conditions for our kinetic studies can be found in Figure 2B.

**Product Analysis**. In the case of linear *n*‐butanol (**N3**) and branched *tert*‐butanol (**N8**), representative product analyses of in situ prepared reactions were conducted by ^1^H and HSQC nuclear magnetic resonance (NMR). These analyses revealed that the benzhydrilium ions are primarily converted to their corresponding benzhydryl ether, further confirmed by high‐resolution mass spectrometry (HRMS, see Supporting Information Section V).

**Kinetic Studies with and without VSC**. We began our kinetic investigation by studying the effect of lutidine on our measurements. Numerous studies of the capture of carbocations by alcohols and water report that this reaction can proceed with or without general base catalysis.[^29^, ^30^, ^31^, ^32^] In other words, a base like lutidine may be involved in the rate‐determining step. Thus, we initially studied the dependency of the reaction rate on the concentration of lutidine to investigate whether general base catalysis is relevant in our system. However, in the reaction of methanol with **E5**, we only observed a slight dependence of the rate on lutidine concentration. With 400 equivalents of lutidine compared to **E5**, the reaction was only 19 % faster than with ten equivalents, likely ruling out general base catalysis to be of significant importance (see Supporting Information Section IX). Accordingly, the rate‐determining step (rds) of the reaction of benzhydrylium ions with alcohols corresponds to the nucleophilic attack at the carbocation, and deprotonation only occurs in a fast subsequent reaction.

Having defined the rds of the reaction, we next studied the reaction kinetics without VSC, i.e., our standard kinetic measurements. The solution of lutidine in alcohol and that of the electrophile **E** in acetonitrile were mixed in an 8 : 2 ratio, and within 20 seconds after mixing, the solution was injected into the IR‐transparent windows and placed in the chamber of a UV/Vis spectrometer. As the reaction products of the cations **E** are colorless, monitoring the disappearance of the E's blue color affords a kinetic trace (see Figure 2C, left, for the reaction of ethanol (**N2**) with electrophile **E1**). Due to the large excess of the alcohols over the electrophiles, pseudo‐first‐order kinetics resulted. The fitting of a first‐order decay (*A* exp (−*k*_obs_ t)+*C*) to the decay curves afforded the rate constants *k*_obs_ (Figure 2C, right). Each experiment was reproduced at least three times, and the standard error was determined for both standard and later cavity kinetic measurements.

**Figure 3:**
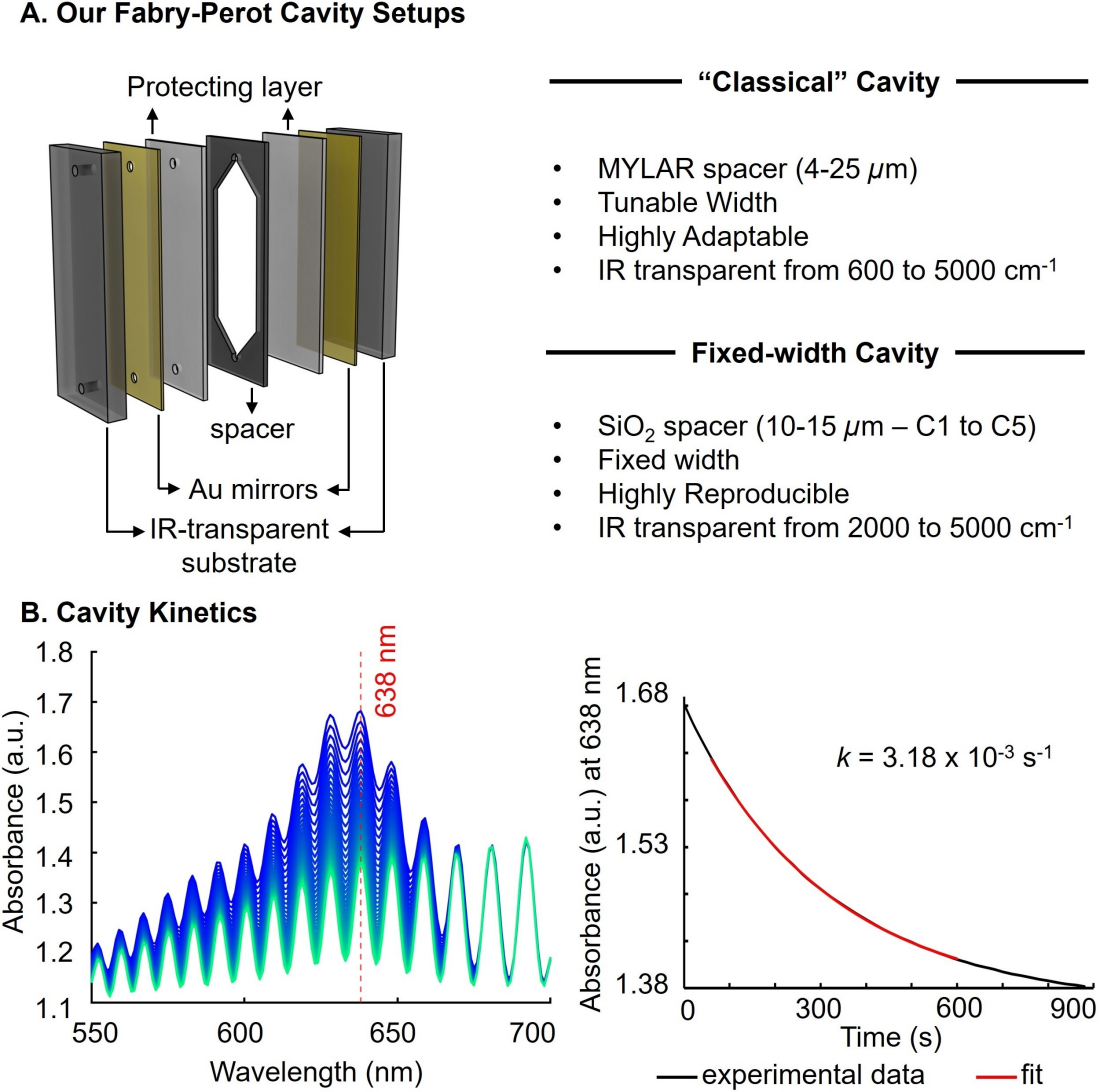
A. Schematic representation of our different Fabry‐Perot cavities setups and summary of their key properties. B. Fixed‐width cavity UV/Vis spectra resulting from the reaction of benzhydrilium ion **E1** (2.4 mM) with EtOH (**N2**) (left) and kinetic trace (black) fitted to a first‐order rate constant (red, right). The reaction conditions are the same as in Figure 2B−C.

Next, we performed all cavity measurements in our recently developed fixed‐width cavities,^[45]^ as minor variations in width in the classical cavity setup were found to cause significant errors due to baseline variations in our system (see Supporting Information Section III). The fixed‐width cavities also made repeating experiments easier because their width does not have to be tuned manually, like in the classical setup (Figure 3A). They come in five different thicknesses ranging from 10–15 μm, referred to as C1–5. Each of these cavities has optical modes of different energies and may couple different bands in each system. An example of the UV/Vis spectra, kinetic trace, and fitting obtained from a fixed‐width cavity and the reaction of **E1** and ethanol (**N2**) is given in Figure 3B.

Of note is that the large end absorbance (≈1.4 A.U.) that can be seen in Figure 3B results primarily from the intrinsic absorbance of the gold mirrors of the cavity (cf. Figure 3A), as we did not perform a background subtraction of the empty cavity.^[46]^ The oscillatory line shape observed in the UV/Vis spectrum can be seen as the extension of the modes observed in the IR region, albeit with a lower finesse due to the lower reflectance of the gold layer at visible wavelength compared to infrared wavelengths.[^47^, ^48^, ^49^] Finally, in light of recent work showing the difficulties that can be met when measuring UV/Vis spectra in cavities,^[50]^ we tested the validity of the Beer–Lambert law within our Fabry‐Pérot cavities. As shown in Supporting Information Section IV, we observed an excellent linear correlation between concentration and absorbance of the benzhydrylium ion **E1** for measurements inside fixed‐width cavity **C1**.

**Figure 4:**
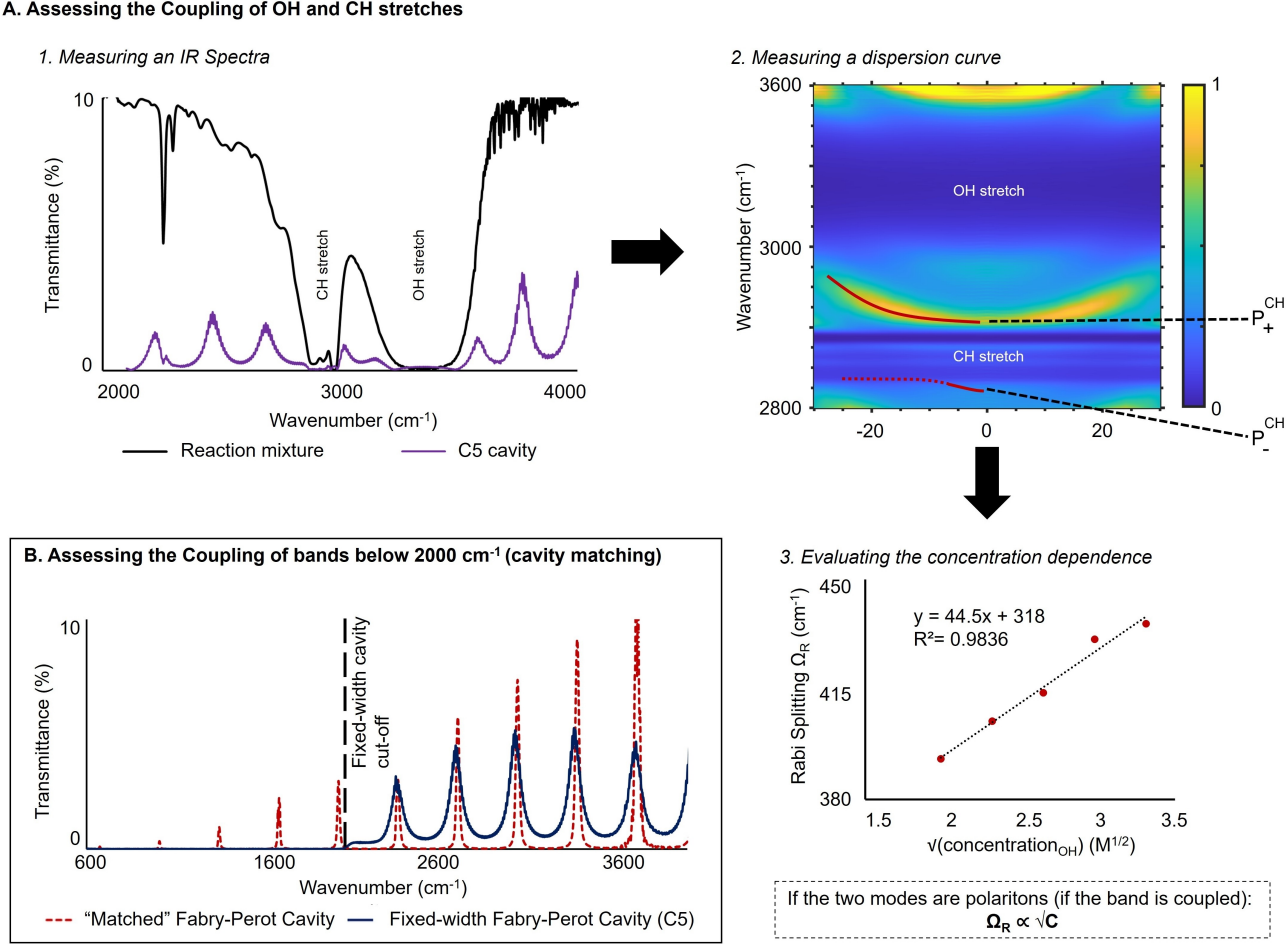
Workflow for the assessment of coupling used in our study. A. Methodology for verifying the coupling of OH and CH stretches. 1. Infrared spectra are measured to qualitatively estimate the potential coupling of the bands. In this specific IR spectrum, the OH and CH stretches might be coupled, but the presence of polaritons is not obvious 2. This infrared spectrum is measured at different incident angles to measure a dispersion curve. As shown here by the red lines following the spectral features of interest, an anti‐crossing, which is a signature of strong coupling, is observed. In this case, the CH stretch is clearly strongly coupled, but coupling remains unclear for the OH stretch. 3. Concentration dependence experiments are subsequently performed, and the position of the features of the cavity IR spectrum is followed. If their position varies as a function of the square root of the concentration, these features are due to the formation of polaritons (cf. Figure 1). B. Methodology for verifying the coupling of vibrational bands below 2000 cm^−1^. Specific examples are shown later in Figure 5.

**Assessment of coupling**. We used a systematic workflow to establish the coupling of all bands for all nucleophiles, which is summarized in the specific example of ethanol (**N2**) in Figure 4. To define which bands were coupled in each size of the fixed‐width cavity, we started by measuring an infrared spectrum to qualitatively examine the bands that could be coupled (Figure 4A–1). Then, a dispersion curve at varying angles of incidence was measured (Figure 4A–2). In some cases, a clear anti‐crossing, a signature of strong coupling, allowed us to determine that a given band was coupled (as indicated by a red line for the CH stretch in Figure 4A–2). For reference on how the anti‐crossing in question can be identified, we suggest the reviews of Ebbesen^[1]^ and Simpkins.^[6]^ Noteworthily, in our case, some of the smaller features in the spectra are sometimes difficult to see owing to the optical quality of the fixed‐width cavities, which are still prototypical (as is, for instance, the case of P‐ in Figure 4A–2, dotted red line).

In some cases, concentration‐dependence studies were performed to confirm the coupling of OH and CH stretches, taking advantage of the proportionality of the Rabi Splitting with the square root of the concentration (as shown by the example of the OH stretch in Figure 4A–3, also see Supporting Information section VI).

Because the infrared cut‐off of the fixed‐width cavities is at 2000 cm^−1^ (Figure 3A and 4B, dotted black line), the presence or absence of strong coupling of bands below that threshold cannot be established using the two latter methods.

Bands below 2000 cm^−1^ were therefore examined within a classical Fabry‐Perot cavity setup (for which the cut‐off is around 600 cm^−1^, see Figure 3A). The width of the cavity was tuned to match the width of each given fixed‐width cavity of interest, which could be verified by the overlap of their IR spectra (example in Figure 4B; this method will be referred to as the “matched” spectrum for the rest of the study).

Using this strategy, we could reproduce all cavities of interest almost exactly (±2 cm^−1^ free spectral range variation), thus gaining additional insight into the coupling of bands other than CH (~2900 cm^−1^) and OH (~3300 cm^−1^) stretches. All information regarding the assessment of coupling is included in Supporting Information Section V.

### Testing the Effect of VSC on Reaction Kinetics

**Observed Rate Constants**. Gratifyingly, using our methodology for measuring kinetics under VSC, we obtained a very high level of reproducibility, with errors generally being contained between 1 and 5 % between different measurements of first‐order rate constants (Table 1). We first used the workflow outlined above to investigate the effect of coupling OH and CH stretches on the rate constants of the reactions of alcohols and electrophiles **E**. We started by studying the reaction of methanol (**N1**) with **E1** as the electrophile. When coupling the OH and CH stretches of **N1**, no change in the observed first‐order rate constant was observed compared to the uncoupled standard reaction (i.e., IR‐transparent windows, as shown in the Experimental Setup section, were used). With ethanol (**N2**), we found that coupling of both OH and CH stretches resulted in a 9 % increase in the observed rate constant relative to the non‐coupled control experiment. Similarly, a 23 % increase in *k*_obs_ with respect to the control was observed for *n*‐butanol (**N3**) when coupling both bands. Finally, for nonanol (**N4**), we could not couple the OH stretch using the available fixed‐width cavities, and coupling the CH stretch alone did not result in any significant change in the rate constant. Moving on, we investigated secondary alcohols. For isopropanol (**N5**), isobutanol (**N6**), and cyclobutanol (**N7**), no difference in rate constants between coupled and non‐coupled measurements was found when simultaneously coupling the OH and CH stretches. Similarly, for the tertiary alcohols *tert*‐butanol (**N8**) and 3‐ethyl‐3‐pentanol (**N9**, only CH coupled), no change in the rate constant was observed. Finally, we looked at benzyl alcohol (**N10**) and again found no change in kinetics when coupling the OH stretch. Lastly, we studied the nucleophilicity of water (**N11**). With both **E3** and **E4**, we again observed no significant change when coupling the OH stretch of water (see Supporting Information Section VI).

**Table 1:**
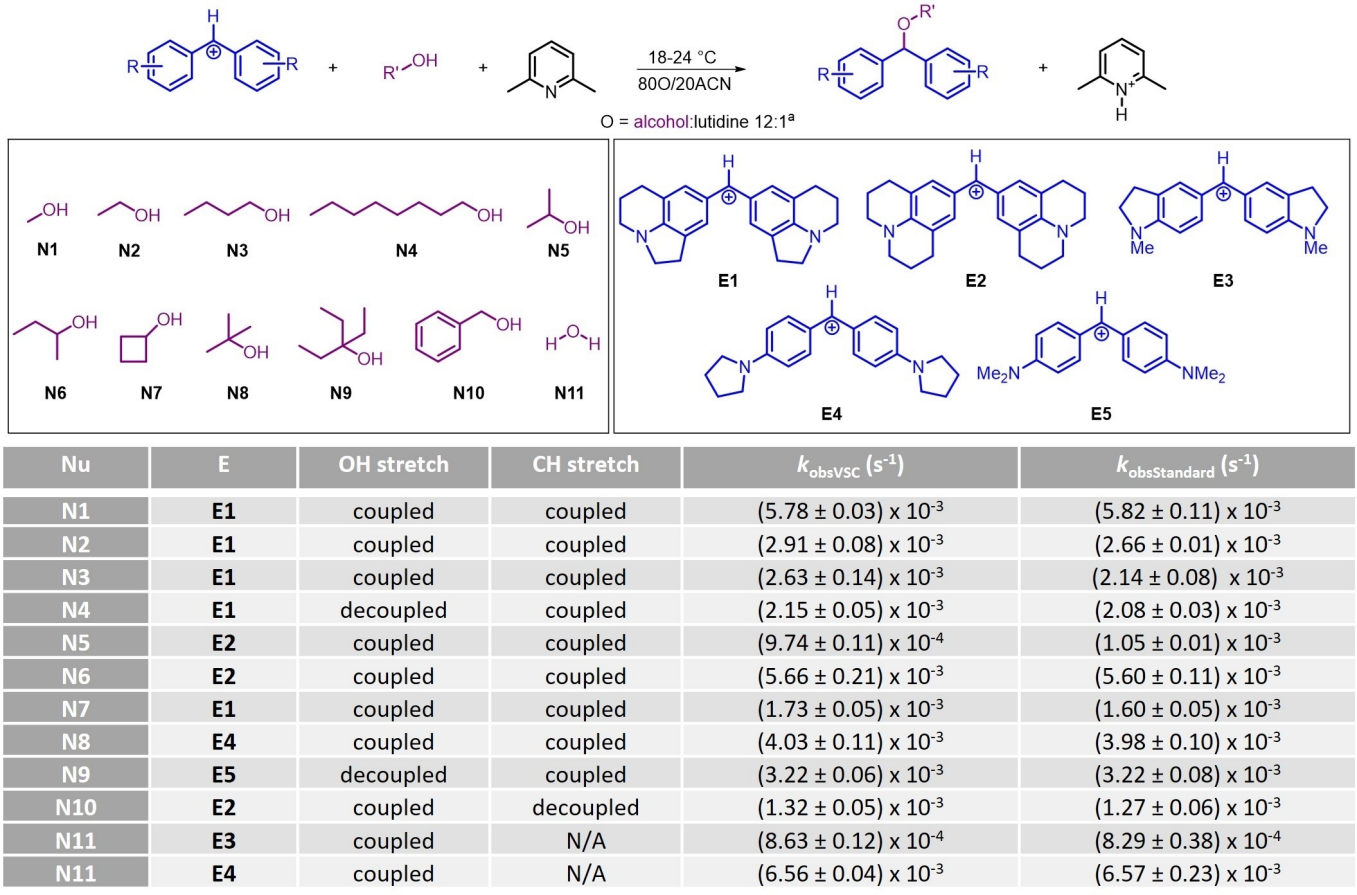
Reaction conditions, substrates, and observed first‐order rate constants (average over at least three measurements; the error corresponds to the standard error). For the benzhydrylium ion E, the counter ion is BF_4_^−^ in all cases. ^a^ for **N11**, 50 mM MOPS buffer solution was used instead of the alcohol:lutidine mixture at pH=7.0.

**Figure 5:**
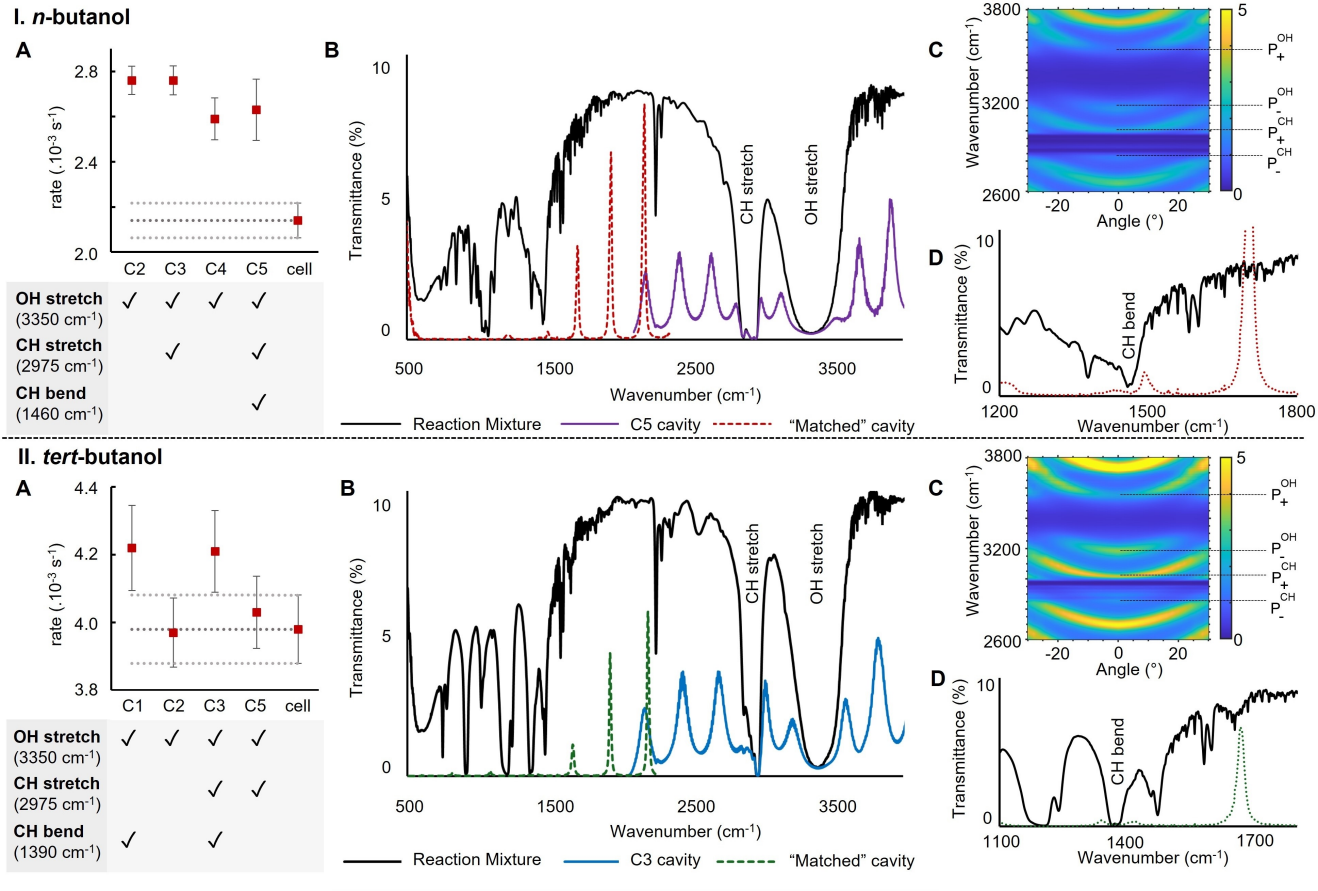
A. Effect of coupling on the observed rate constant for (I) *n*‐butanol and (II) *tert*‐butanol. B. Infrared spectra of the reaction mixture, cavity, and “matched” cavity. C. Angle dependence of the coupling. D. Infrared spectra showing the coupling of bands below the IR cutoff of 2000 cm^−1^ as observed in “matched” cavities.

**Systematic Coupling‐Rate Study**. Having not observed a clear effect on the rate constants after coupling the C−H or O−H vibrations, we set out to investigate the coupling of other characteristic bands of alcohols. We tested whether any bands were also likely to be coupled below the cut‐off of 2000 cm^−1^ by matching the different wafers of interest with classical cavities, as described above (Figure 5, IB and IIB). For *n*‐butanol (**N3**), we found that cavity C5, which couples the OH and CH stretches (Figure 5, IC), likely also couples the CH bending mode (1460 cm^−1^,

Figure 5, ID), while cavities C4 and C2 coupled the OH stretch only, and C3 both the CH and OH stretches (Figure 5, IA). Thus, we could vary the coupling scenario by changing the cavity used. However, upon measuring kinetics in these additional fixed‐width cavities, we found the difference in rate constant compared to the standard independent of the coupled bands, provided that the OH stretch was coupled (Figure 5, IA).

Next, we proceeded to analogously study the coupling of further bands for *tert*‐butanol (**N8**). Once again, we found that depending on the fixed‐width cavity used, different bands could be coupled and decoupled. For instance, in C3, the CH bending mode could be coupled (Figure 5, IID), and the rate constant slightly increased (Figure 5, IIA). C3 was also shown to couple the OH and CH stretches through angle‐dependence measurements (Figure 5, IIC).

On the other hand, cavity C2, which showed coupling to only the OH stretch, yielded the same rate constant as the standard (Figure 5, IIA). Finally, when using cavity C1, the OH stretch and CH bending modes could be coupled, yielding a slight increase in rate constant that barely exceeded the experimental error.

Looking at *n*‐butanol (**N3**), only coupling of the OH stretch might impact the reaction rate constant. However, as all fixed‐width cavities C1−C5 available to us coupled the OH stretch, we could not verify changes in the rate constants without specifically coupling this band, thus precluding any conclusions. Regarding *tert*‐butanol (**N8**), coupling the CH bending mode slightly increases the rate constants with respect to the non‐coupled control measurements. Noteworthily, coupling the OH stretch of *tert*‐butanol did not result in an altered rate constant, in contrast to *n*‐butanol. Therefore, from one alcohol to another, there does not appear to be a clear relationship between which band is coupled and any resulting change in the rate constant.

To gain further insight on the cause of the slight rate increase we observed between cavity and cell scenarios for *n*‐butanol, we then attempted to see if the acceleration of the reaction by cavities (k_VSC_/k_cell_) could be correlated to the concentration of the alcohol (Figure S16). To do so, we performed two more series of kinetic experiments with alcohol/acetonitrile ratios of 90/10 and 70/30 (v/v)—in contrast to the original experiment where the ratio was 80/20. For these experiments, we selected cavity C2, as it showed the strongest acceleration and only couples the OH stretch, allowing to selectively look at the importance of the alcohol. Doing so, we found that the acceleration caused by the cavity did not seem related to the concentration in alcohol, thus suggesting once more that VSC is not responsible for the observed change. Finally, we note that parameters such as temperature and cavity surface/volume ratio were not found to be significant contributors to the measured rate constants (see Supporting Information Section VIII).

## Conclusion

Herein, a methodology for the measurement of reaction kinetics was developed using fixed‐width microfluidic cavities. Using this setup, we studied the nucleophilicity of alcohols and water under VSC. The disappearance of the strong blue color of established benzhydrylium ions in their reactions with alcohols and water was used to study the kinetics of their reaction with UV/Vis spectroscopy. In most cases, the rate constant measured when coupling the OH and/or CH stretches proved identical to that of the control (without VSC). In a few cases, we found a small increase in the reaction rate constant under VSC (at maximum by 23 %). We then systematically studied the impact of coupling and decoupling important vibrational bands of the alcohol (OH and CH stretching modes and CH bending mode) and could not identify a trend between the coupling scenario and the rate constant increase. Further, concentration dependence studies show no correlation between alcohol concentration and rate acceleration. The rate constant changes observed are thus unlikely to be due to VSC. Accordingly, VSC does not substantially alter the nucleophilicity of the nucleophiles studied herein, or any effect is so small that it cannot be accurately quantified with our experimental approach.

With the new method reported herein, we significantly facilitate access to large and reproducible kinetic datasets, representing significant progress in VSC as a field of study. Our data, as well as future data collected in this way, will help shape the interpretation of studies using water or alcohol as nucleophilic reaction partners in VSC. By focusing on the crucial property of nucleophilicity, this work helps deconvolute the complex issue of rate modification under VSC.

## Conflict of Interests

The authors declare no conflict of interest.

1

## Supporting information

As a service to our authors and readers, this journal provides supporting information supplied by the authors. Such materials are peer reviewed and may be re‐organized for online delivery, but are not copy‐edited or typeset. Technical support issues arising from supporting information (other than missing files) should be addressed to the authors.

Supporting Information

## Data Availability

The data that support the findings of this study are available in the supplementary material of this article.
