# Supplementary material for: Measuring Kinetics under Vibrational Strong Coupling: Testing for a Change in the Nucleophilicity of Water and Alcohols
Source: Angew Chem Int Ed Engl. 2024 Oct 24;63(49):e202410770. doi: 10.1002/anie.202410770 (PMC11586696; doi:10.1002/anie.202410770)
Supplement: Supplementary file 1 — Supporting Information [file ANIE-63-e202410770-s001.pdf]

Angewandte Chemie
Eine Zeitschrift der Gesellschaft Deutscher Chemiker

Supporting Information

Measuring Kinetics under Vibrational Strong Coupling: Testing for a Change in the Nucleophilicity of Water and Alcohols

*C. Muller, R. J. Mayer, M. Piejko, B. Patrahau, V. Bauer, J. Moran**

Table of Contents

I. General Information **4**

II. Sample Preparation **7**

III. Experimental Setup **8**

IV. Validity of the Beer-Lambert law in cavity **11**

V. Product Analysis **13**

n-butanol: 14

tert-butanol: 17

VI. Assessing Coupling **21**

General Procedure: 21

N1 (Methanol, C5) 22

N2 (Ethanol, C5) 23

N3 (*n*-butanol, C5) 24

N3 (*n*-butanol, C4) 24

N3 (*n*-butanol, C3) 25

N3 (*n*-butanol, C2) 25

N4 (Nonanol, C5) 26

N5 (*iso*-propanol, C5) 26

N6 (*iso*-butanol, C5) 27

N7 (*cyclo*-butanol, C4) 27

N8 (*tert*-butanol, C5) 28

N8 (*tert*-butanol, C3) 28

N8 (*tert*-butanol, C2) 29

N8 (*tert*-butanol, C1) 29

N9 (3-ethyl-3-pentanol, C5) 30

N10 (Benzyl Alcohol, C5) 30

N11 (Water, C5) 31

VII. Kinetics **32**

Reaction of methanol (N1) with electrophile E1: 32

Cavity measurements (C5) 32

Cell measurements (standard) 35

Results 38

Reaction of ethanol (N2) with electrophile E1: 39

Cavity measurements (C5) 39

Cell measurements (standard) 44
Results 47
Reaction of *n*-butanol (N3) with electrophile E1: 48
Cavity measurements (C2) 48
Cavity measurements (C3) 51
Cavity measurements (C4) 54
Cavity measurements (C5) 60
Cell measurements (standard) 63
Results 66
Reaction of *n*-butanol (N3) with electrophile E1 (90/10 v/v): 67
Cavity measurements (C2) 67
Cell measurements (standard) 70
Results 73
Reaction of *n*-butanol (N3) with electrophile E1 (70/30 v/v): 74
Cavity measurements (C2) 74
Cell measurements (standard) 77
Results 80
Dependence of the k_{vsc}/k_{cell} ratio on the concentration of alcohol: 80
Reaction of nonanol (N4) with electrophile E1: 81
Cavity measurements (C5): 81
Cell measurements (standard) 84
Results 87
Reaction of *iso*-propanol (N5) with electrophile E2: 88
Cavity measurements (C5) 88
Cell measurements (standard) 91
Results 94
Reaction of *iso*-butanol (N6) with electrophile E2: 95
Cavity measurements (C5) 95
Cell measurements (standard) 98
Results 101
Reaction of *cyclo*-butanol (N7) with electrophile E1: 102
Cavity measurements (C4) 102
Cell measurements (standard) 105
Results 108
Reaction of *tert*-butanol (N8) with electrophile E4: 109

Cavity measurements (C1) 109
Cavity measurements (C2) 112
Cavity measurements (C3) 115
Cavity measurements (C5) 118
Cell measurements (standard) 121
Results 124
Reaction of 3-ethyl-3-pentanol (N9) with electrophile E5: 125
Cavity measurements (C5) 125
Cell measurements (standard) 130
Results 133
Reaction of benzyl alcohol (N10) with electrophile E2: 134
Cavity measurements (C5) 134
Cell measurements (standard) 137
Results 140
Reaction of water (N11) with electrophile E4: 141
Cavity measurements (C5) 141
Cell measurements (standard) 144
Results 147
Reaction of water (N11) with electrophile E3: 148
Cavity measurements (C5) 148
Cell measurements (standard) 151
Results 154
VIII. Other parameters 155
Temperature 155
Surface 156
Linearity of the Spectrometer 157
IX. Importance of lutidine 158
X. References 159

I. General Information

All compounds were used as received from commercial suppliers. Benzhydrilium ions **E1-E5** were obtained from the group of Prof. Herbert Mayr and Dr. Armin Ofial (LMU Munich) where they were prepared using published procedures.[1] The cavities referred to as "fixed-width" were purchased according to our specifications by LioniX International. Their composition and design were recently reported.[2] Standard measurements were performed using calcium fluoride substrates separated by a 25 µm MYLAR spacer purchased from Specac (this setup is referred to herein as "cell").

The IR spectra of Fabry-Perot cavities and cells were acquired using a standard FT-IR (Fourier Transform Infrared) spectrometer (Bruker INVENIO®) in transmission mode with an MCT (Mercury-Cadmium-Telluride) detector with 2 cm^{-1} resolution and averaged over 16 scans. UV-vis spectra were recorded with a Shimadzu UV-2600i spectrometer using an aperture of 5 mm and 0.1 nm resolution. All spectra were recorded at temperatures between 18 and 24 °C, and temperature variations in the room were monitored and noted for each measurement to exclude temperature variations from being the main factor in affecting reaction rates. For standard measurements, a simple background of the air was performed. For cavity measurements, the background was measured through the empty customized setup shown in Figures S3-4. After measuring the background, the fixed-width cavity was then inserted within the setup, and the kinetics were measured.

Due to the large excess of the nucleophilic alcohols over the benzhydrylium ions, pseudo-first-order kinetics resulted, which greatly simplifies the kinetic analysis. Most importantly, in a first-order process, the change in concentration over time is proportional to the concentration itself. Thus, any measurement that is proportional to the concentration can be used to analyze the kinetics, and the absolute concentration of the reactant is not needed. In our case, we opted for absorbance spectroscopy.

E.g., for a first-order process (eq. I), the integrated rate law takes the form of eq. II. The Lambert-Beer law (eq. III) can be re-arranged to express the concentration of species C as a function of the absorbance A, the extinction coefficient ε, and the path length l.

$$\frac{d[C]}{dt} = -k[C]\ (I)$$

$$[C] = [C]_0 e^{-kt}\ (II)$$

$$[C] = \frac{A}{\varepsilon * l}\ (III)$$

Substituting (III) in (II) yields expression (IV):

$$\frac{A}{e * l} = \frac{A_0}{e * l} e^{-kt}\ (IV)$$

Under the assumption that the pathlength l and the extinction coefficient ε do not change throughout the reaction, eq. (IV) simplifies to (V).

$$A = A_0\, e^{-kt} \qquad (V)$$

All absorbance-time profiles were analyzed using Matlab, and pseudo-first-order rate constants were obtained by fitting single exponential V to the kinetic data. All reported errors correspond to standard errors.

For product analyses, NMR spectra were recorded on a Bruker 400 MHz spectrometer at a sample temperature maintained at 20 °C. High-resolution mass spectrometry (HRMS) analysis was performed on a Thermo Scientific Exactive Plus EMR (ESI-Orbitrap) using electrospray ionization (ESI).

To study the effect of lutidine on the reaction, kinetics were measured on a Jasco V-670 spectrophotometer equipped with a Peltier-cooled PAC-743 sample changer and an integrated magnetic stirrer at 20 °C. For measurements, Hellma 10 mm quartz glass cuvettes containing a small magnetic stirring bar were employed.

The 3D-printed parts were fabricated from generic yellow 1.75 mm PLA filament using a Creality CR-10 FDM 3D printer equipped with a 0.4 mm nozzle. STL files were sliced with Ultimaker CURA software, using the following parameters: an extrusion temperature of 210 °C, a bed temperature of 60 °C with an additional brim for enhanced bed adhesion, a printing speed of

40 mm/s, a layer height of 0.2 mm, and three wall lines. After printing the brim and any small defects were trimmed off using an exactor knife.

II. Sample Preparation

For each kinetic run, 1.25 mg of benzhydrilium salt **E** was dissolved in 250 µL of acetonitrile. Alcohol solutions were prepared by mixing 3 mL of each alcohol and 250 µL 2,6-lutidine. Right before starting the measurements, in a 1.5 mL glass vial, 10 µL (cavity experiments) or 50 µL (standard experiments) of the acetonitrile solution containing the cation **E** were pipetted into 40 µL (cavity experiments) or 200 µL (standard experiments) of the alcohol/lutidine solution. The subsequent reaction mixture was injected into the cell with a syringe, and into cavities with a micropipette. Both cavities and cells were sealed with parafilm and placed inside the chamber of the UV-Vis spectrometer. After the reaction reached significant conversion (70-90%), the experiment was stopped, and an infrared spectrum was measured. The cells and cavities were then washed with acetone and re-used. It should be noted that even for *tert*-butanol, for which the melting point lies at 25 °C in the pure state, the solution obtained upon mixing with lutidine was a clear liquid, which stayed a liquid even at 4 °C likely due to melting point depression.

For the concentration dependence study for *n*-butanol, the solutions of E1 were prepared by dissolving 0.63 mg of E1 into 125 µL of acetonitrile (for the 90/10 *n*-butanol/acetonitrile) and 1 mg into 300 µL (for the 70/30 *n*-butanol/acetonitrile). The *n*-butanol/lutidine solution was prepared as previously stated.

III. Experimental Setup

A customized setup was used for our cavity experiments (Figure S3-4). When using our “classical” Fabry-Perot cavity setup, we observed an oscillatory pattern as shown in Figure S1. This pattern results from the high reflectivity of the gold layers inside the cavity, which causes a similar cavity effect as that observed in the infrared region in the UV/Vis region of the spectrum.[3] This phenomenon is also referred to as etaloning. We observed that the etaloning pattern inside of the cavity shifted unpredictably, leading to unusable results (see below in Figure S1).

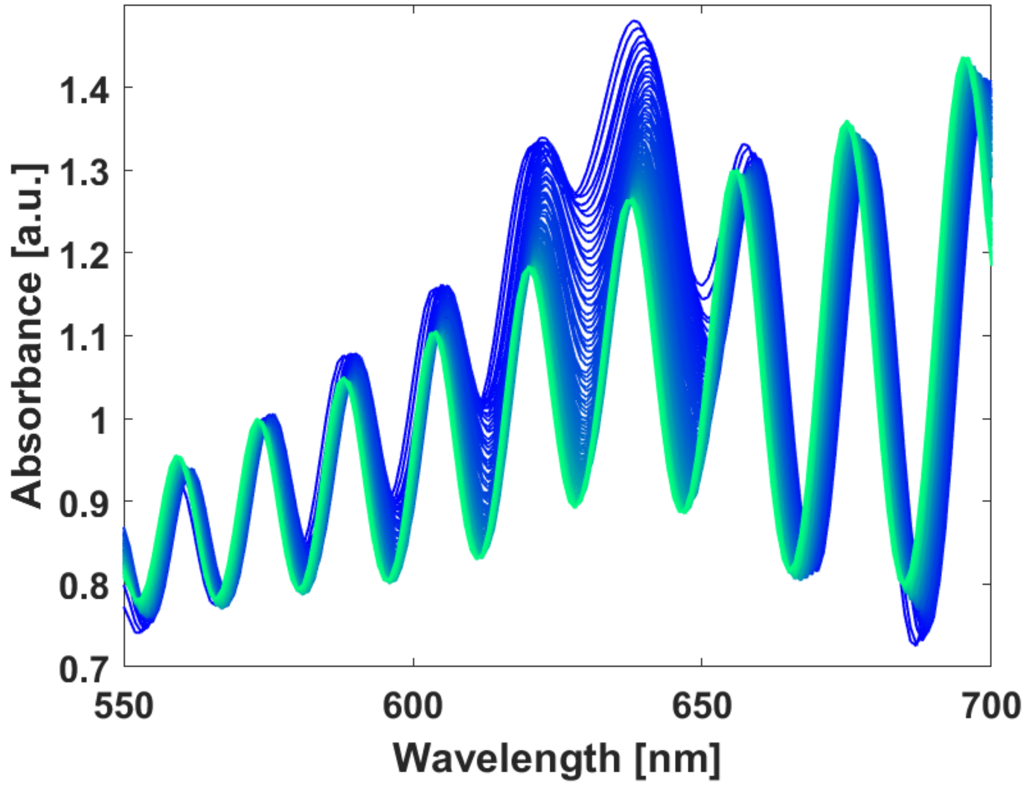

***Figure S1**: Kinetic trace obtained from a "classical" Fabry-Perot cavity and the reaction of methanol (N1) and E1.*

We hypothesized that these variations arose from slight thickness variations that could occur throughout the course of the reaction. With that idea in mind, we used fixed-width cavities, which solved the problem (see Figure S2). To place these cavities in front of the light beam, we constructed a holder using AutoCAD (Figure S3), 3D-printed it, and covered the resulting structure with black opaque tape to avoid any issues with stray light

(see Figure S4). For all our kinetics, we used 5 different wafers of fixed-width cavities at our disposal, which were arbitrarily named C1 (~10 μm), C2 (~11 μm), C3 (~13 μm), C4 (~14 μm), and C5 (~15 μm).

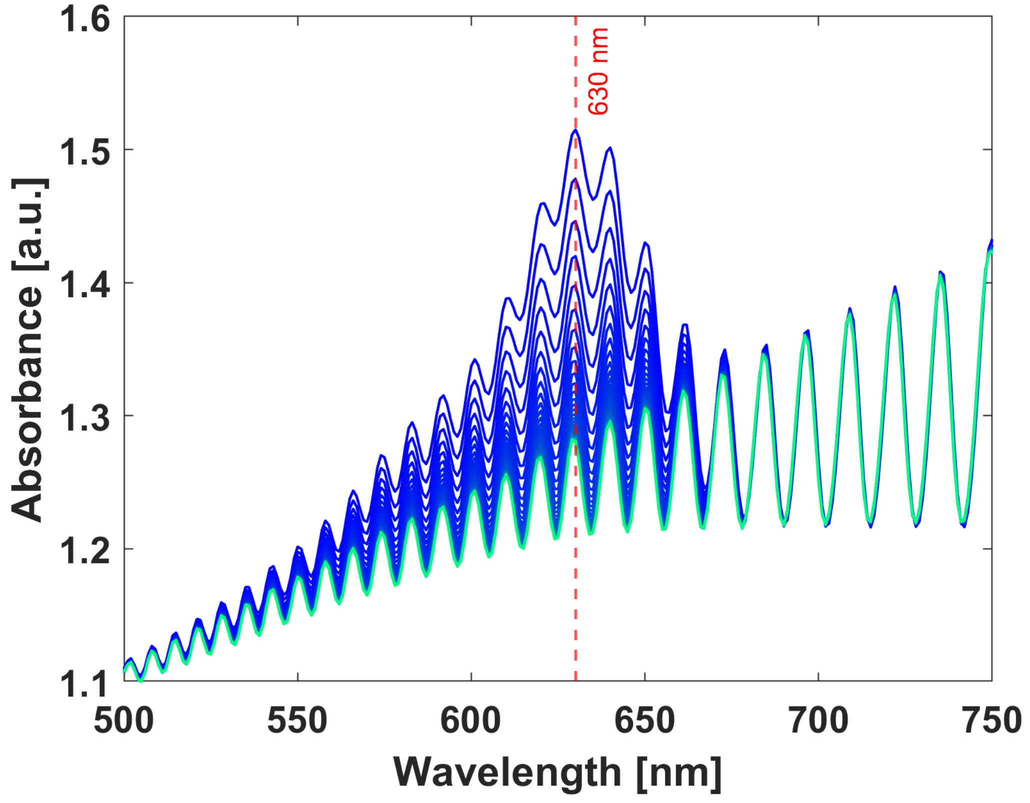

Figure S2: *Kinetic trace obtained from a fixed-width Fabry-Perot cavity and the reaction of methanol (N1) with E1.*

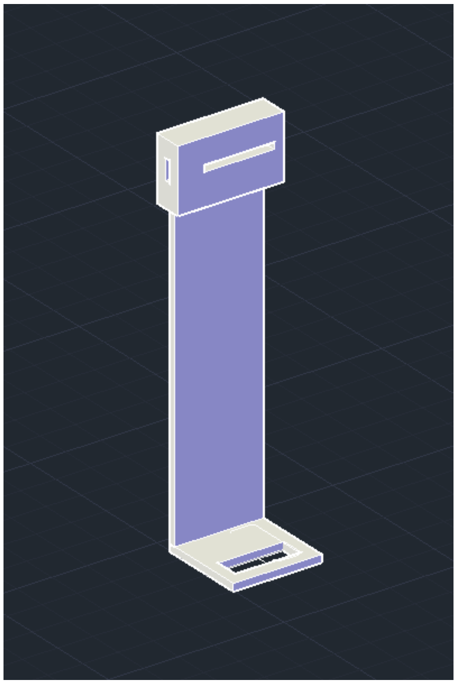

Figure S3: *3D model of the experimental setup for the measurement of UV/Vis spectra with fixed-width cavities.*

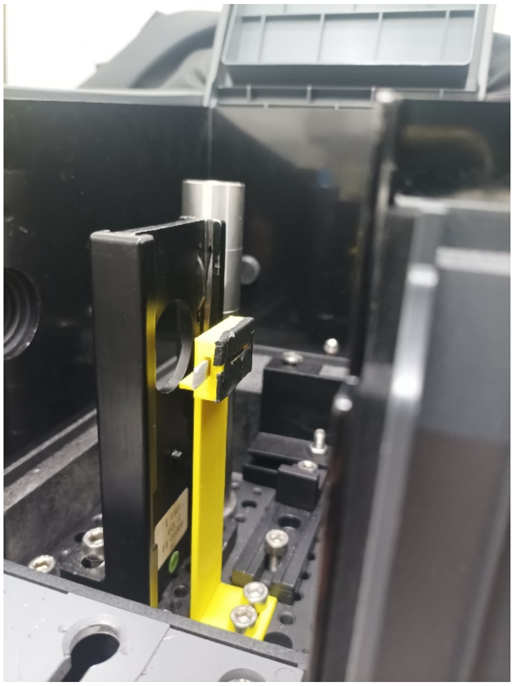

Figure S4: *Photograph of a fixed-width cavity inserted in the experimental setup.*

IV. Validity of the Beer-Lambert law in cavity

In light of recent work highlighting the difficulties that can be met upon measuring UV/Vis spectra in cavity,[4] we performed a linearity study within one of our fixed-width cavities to confirm that the Beer-Lambert law still holds within the context of our study (cf. the assumption in section I that leads from eq. IV to V). To do so, we selected cavity **C1**, and prepared a stock solution of carbocation **E1** which was dissolved in 400 µL of acetonitrile. The stock solution was diluted to cover a difference of absorbance of around 0.4 a. u. (corresponding to a concentration window of 0 to 3.78 mM) which is comparable to the difference in absorbance we observed in our kinetic runs. Each solution was injected within the cavity, and the cavity was subsequently placed within our setup in the UV/Vis spectrometer. To avoid mismatches in baselines between different measurements, we made sure to position the cavities as identically as possible for each measurement. After each measurement, the cavity was flushed and washed before the next solution was injected, and this procedure was repeated until all spectra below were obtained (Fig S5). Following the absorbance at λ_{max} = 639 nm, we observed excellent linearity (Fig S6), which confirms the validity of the Beer-Lambert law within our Fabry-Pérot cavities under the investigated concentrations.

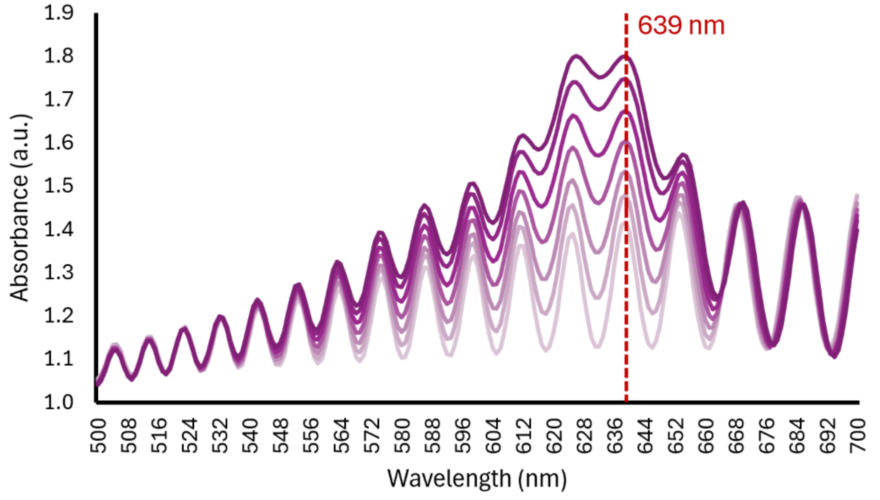

***Figure S5**: UV/vis spectra of carbocation **E1** in acetonitrile at different concentrations (increasing color saturation corresponds to increasing concentration). The red line marks the wavelength that was followed for the subsequent linear correlation in Fig S6.*

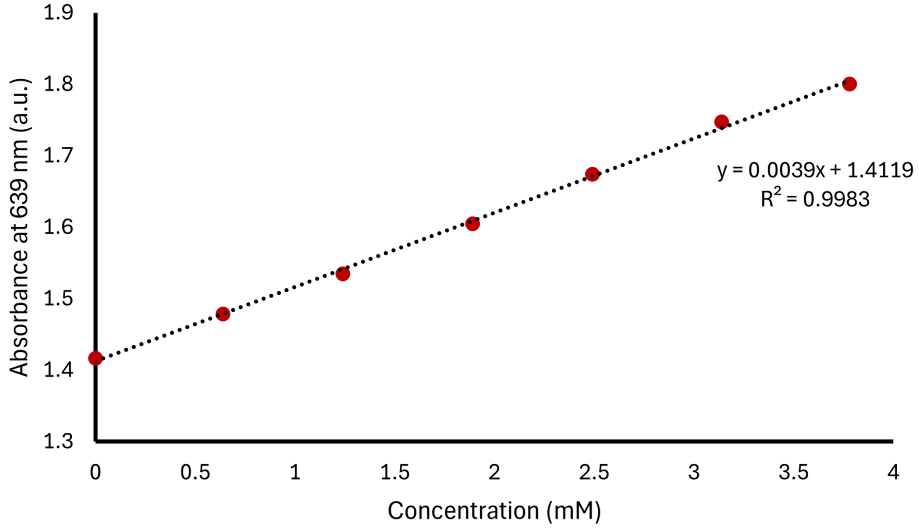

***Figure S6**: Plot of the absorbance value at 639 nm at different concentrations of E1 in acetonitrile and linear fitting (dashed line).*

V. Product Analysis

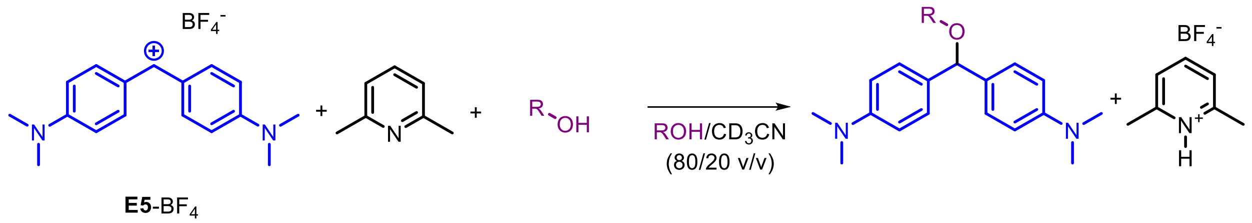

***Figure S7**: Reaction scheme for the product analysis of the reaction of butanol (N3) with electrophile E5.*

The benzhydrilium salt **E5**-BF_4 (2.00 mg, 0.0059 mmol) was dissolved in CD_3CN (100 µL). 400 µL of the previously prepared alcoholic solution (*n*-butanol or *tert*-butanol + 2,6-lutidine, 12:1 v:v) were added to an NMR tube. The benzhydrilium solution was added to yield a deep blue solution, and the tube was closed and shaken. The solution was subjected to analysis by ^{1}H and ^{1}H-^{13}C HSQC NMR. Samples prepared in an analogous way were immediately analyzed by HRMS.

***n*-butanol:**

In the case of *n*-butanol, the blue color disappeared almost immediately.

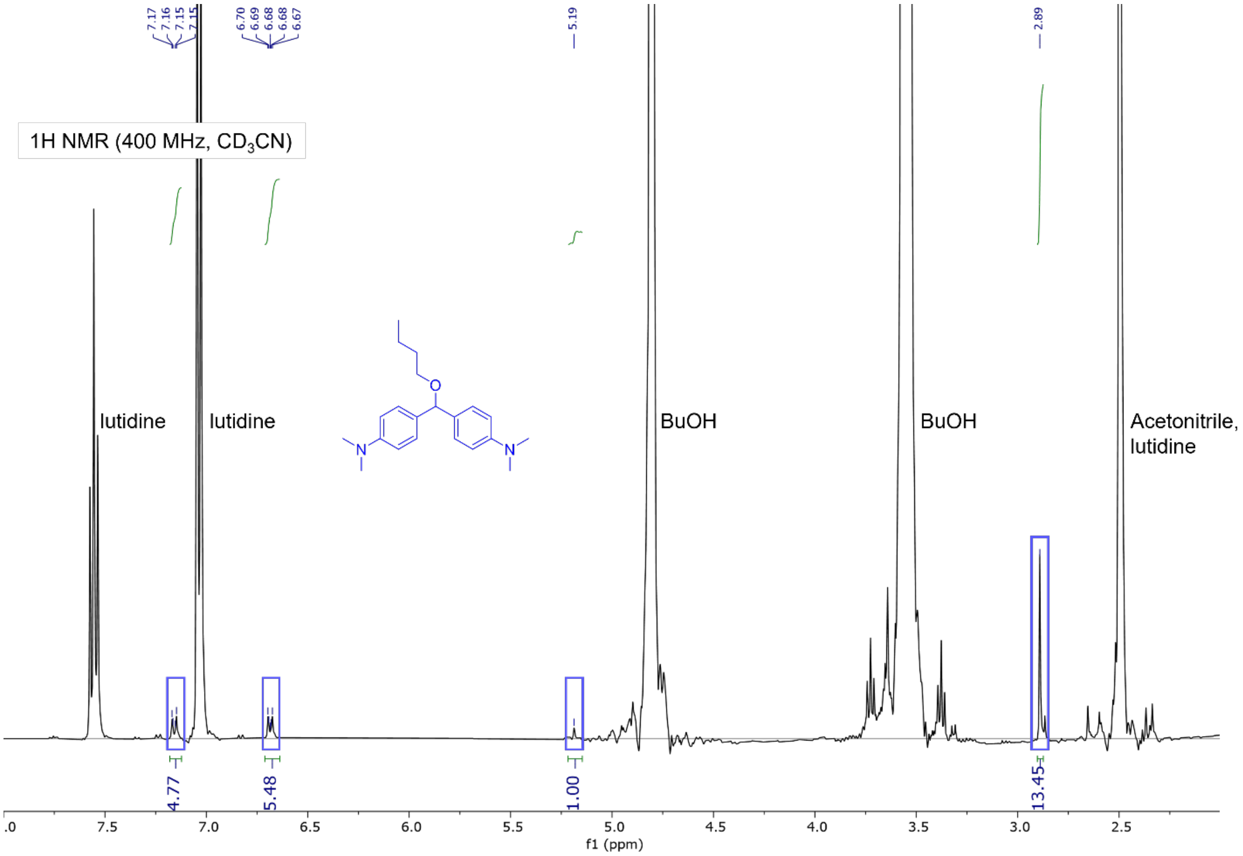

***Figure S8**: 1H NMR spectrum (400 MHz, CD_3CN) of the reaction mixture of* n-*butanol (**N3**) and electrophile **E5**.*

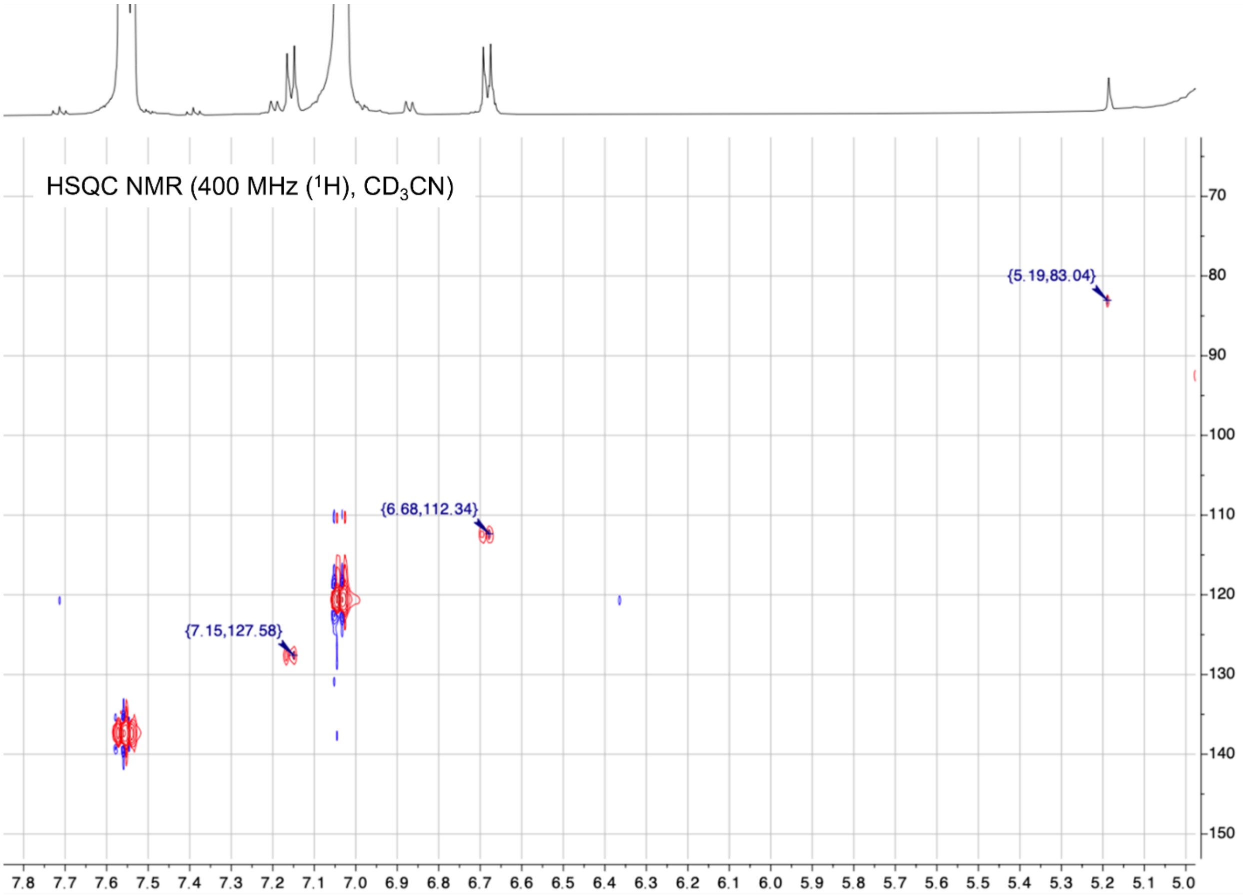

Figure S9: *HSQC NMR spectrum (400MHz (^{1}H), 100MHz (^{13}C), CD_3CN) of the reaction mixture of* n-*butanol (**N3**) and electrophile **E5**.*

The HSQC NMR spectrum shows a correlation between the proton at 5.19 ppm and a carbon nucleus at 83.04 ppm, which is typical for a carbon atom attached to an oxygen atom.

HRMS analysis revealed mainly the presence of lutidine (108 m/z) and a fragment (253 m/z) which can be assigned to the carbocation reforming under the conditions of the measurement, as the reaction of the carbocation could be observed by naked eye (Figure S10). When zooming upon the zone between 240 and 360 m/z, traces of the product can indeed be observed with either a proton or a sodium cation (Figure S11).

Put together, these analyses point towards the formation of the expected ether which almost completely fragments in HRMS.

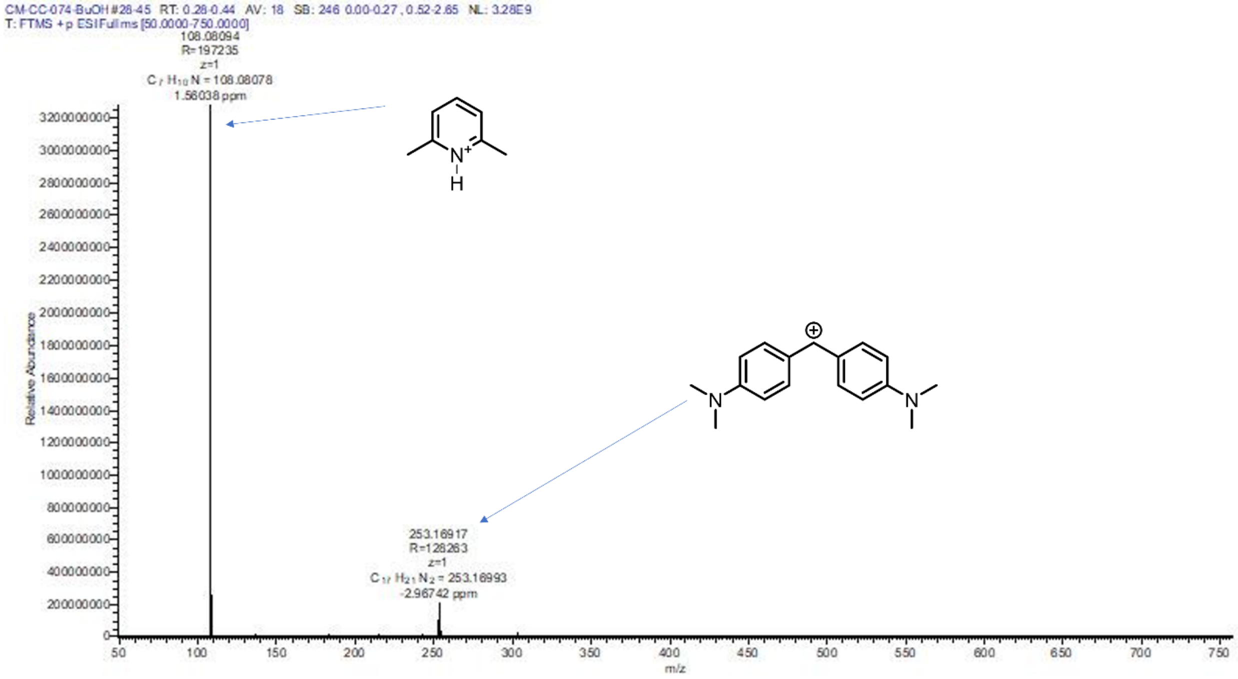

***Figure S10**: HRMS spectrum of the reaction mixture of* n-*butanol (**N3**) and electrophile **E5**.*

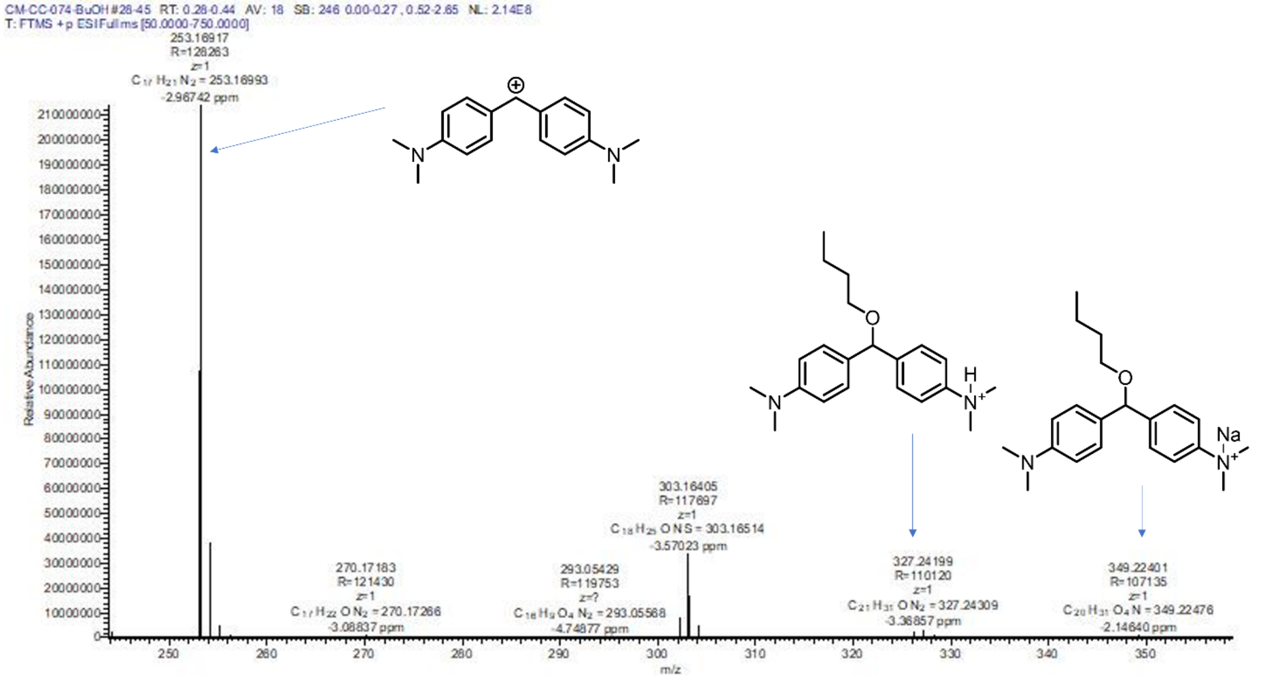

Figure S11**: Zoomed HRMS spectrum of the reaction mixture of* n-*butanol (**N3**) and electrophile **E5.

tert-butanol:

In the case of *tert*-butanol, the blue color faded after 5 minutes.

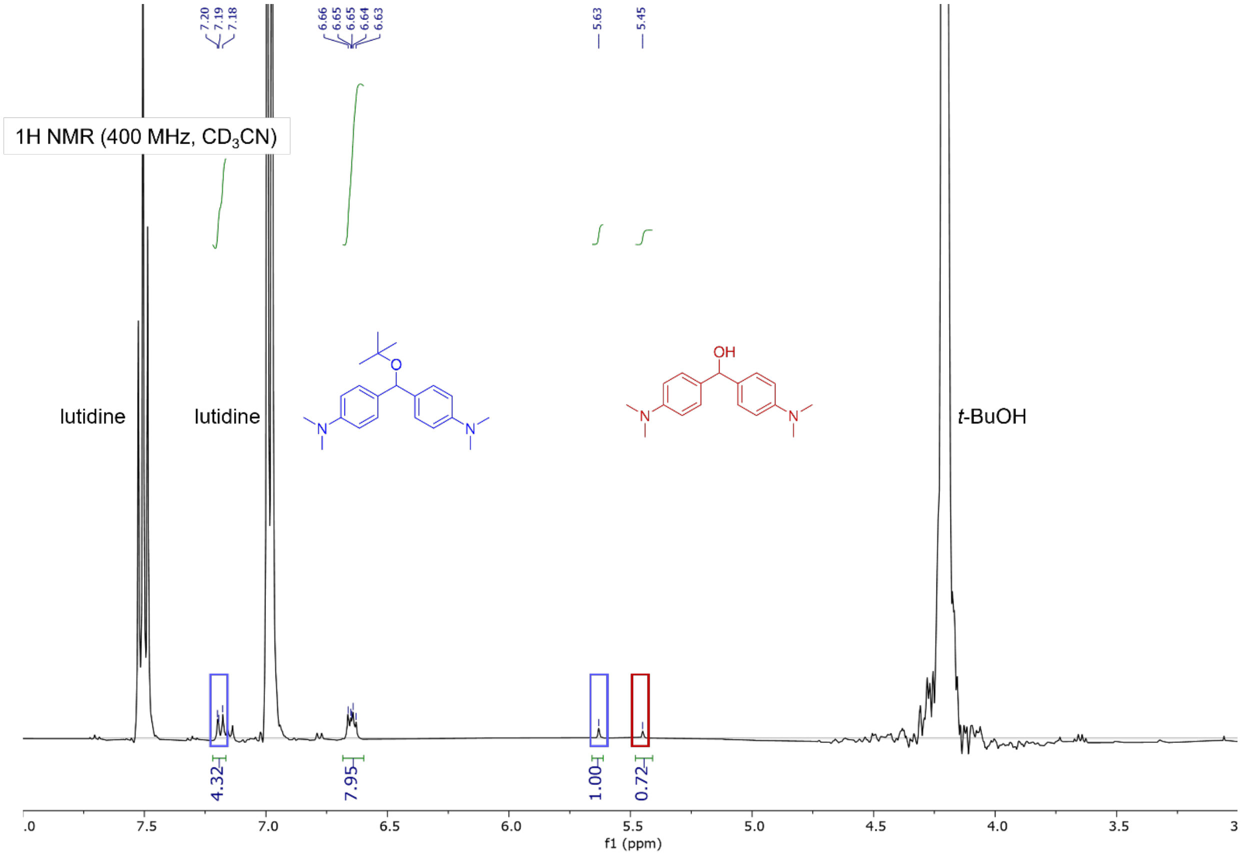

***Figure S12**: 1H NMR spectrum (400 MHz, CD_3CN) of the reaction mixture of* tert-*butanol (**N8**) and electrophile **E5**.*

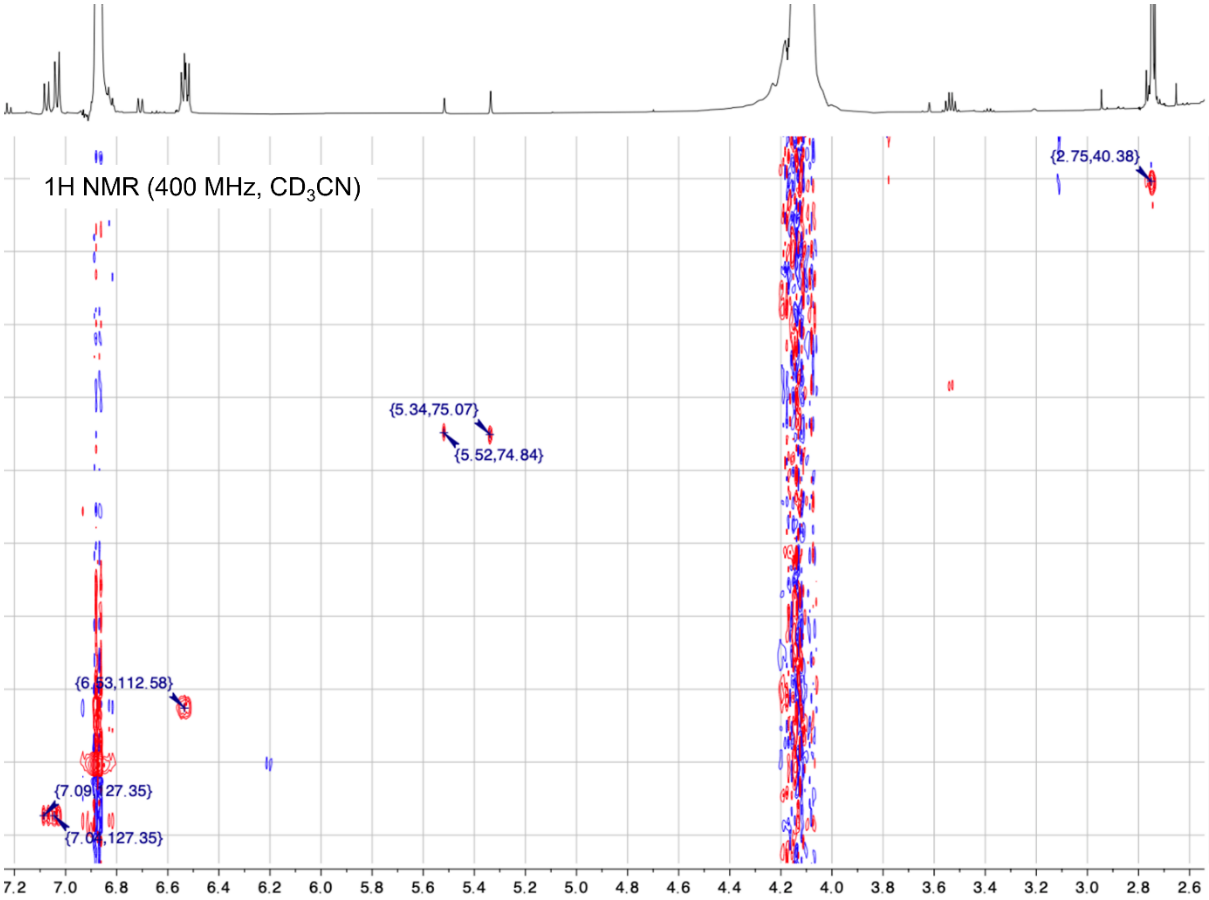

***Figure S13**: HSQC NMR spectrum (400MHz (^{1}H), 100MHz (^{13}C), CD_3CN) of the reaction mixture of* tert-*butanol (**N8**) and electrophile **E5**.*

^{1}H NMR analysis indicates the formation of the expected ether. Additionally, after prolonged standing, the benzhydrol formed as a second product arising from the hydrolysis of the ether by residual water contained in acetonitrile. The HSQC reveals that the protons at 5.34 and 5.52 ppm correlate with carbons at 75.07 and 74.84 ppm, respectively, which is expected for a carbon atom carrying an oxygen atom. Considering the comparable reactivites of water and *tert*-butanol that were observed in our study as well as the significantly bigger amount of *tert*-butanol compared to water in this experiment, the most reasonable explanation for the presence of the benzhydrol product is that it arises from the decomposition of the ether. As our method of analysis for kinetics only measures the disappearance of the blue color of the sample, we consider that only the initial reaction matters and that any decomposition pathway is not having any impact on that region of the UV-Vis spectrum.

HRMS analyses revealed similarly to those conducted for *n*-butanol the main presence of a peak at 108 m/z assigned to lutidine and one at 253 m/z assigned to the carbocation (Figure S14). When zooming upon the zone between 240 and 360 m/z, the mass of the expected product is once again observed (Figure S15). The fragment at 253 m/z could well have been formed from either the ether or the benzhydrol, which thus confirms our NMR analyses.

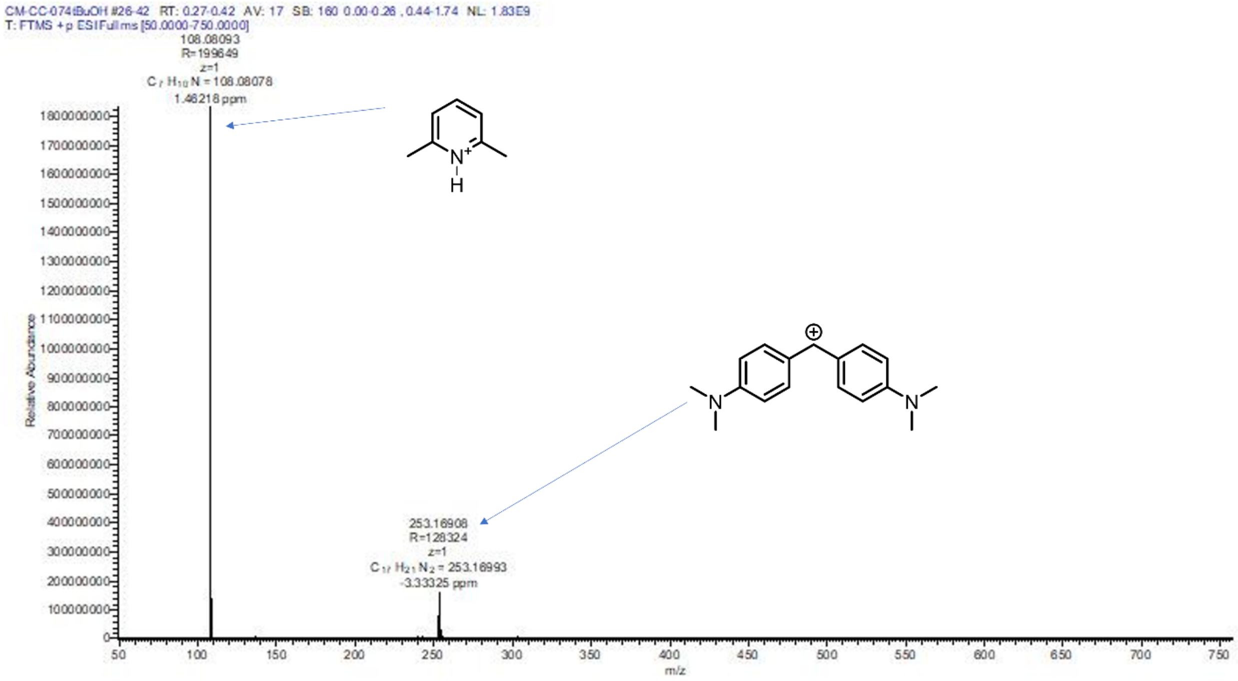

***Figure S14**: HRMS spectrum of the reaction mixture of* tert-*butanol (**N8**) and electrophile **E5**.*

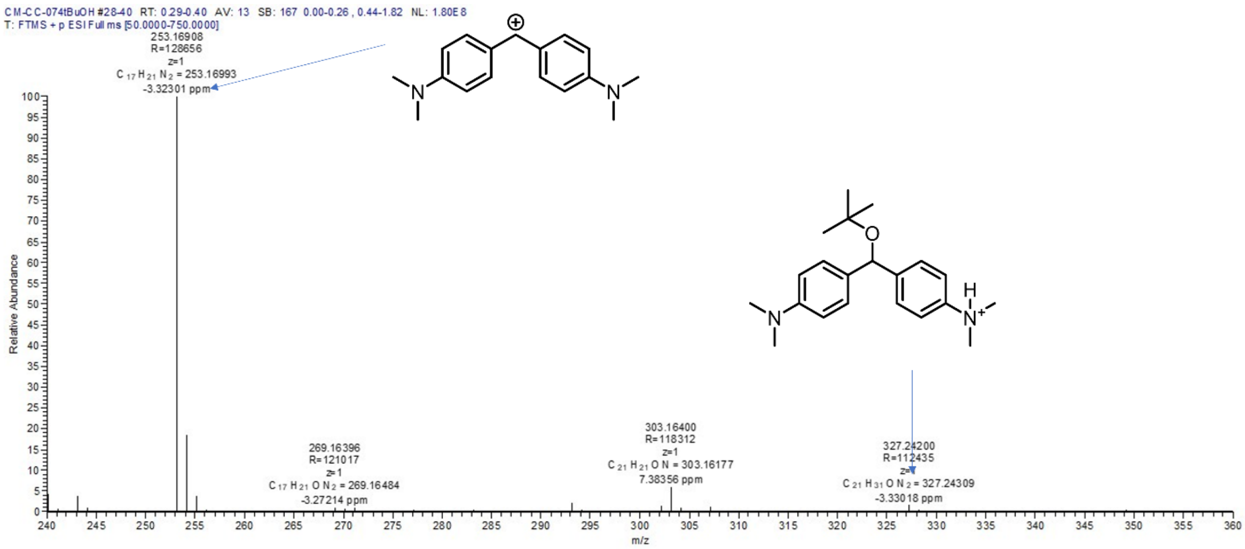

Figure S15**: Zoomed HRMS spectrum of the reaction mixture of* tert-*butanol (**N8**) and electrophile **E5.

VI. Assessing Coupling

General Procedure:

Before kinetics were measured, for each system, the different fixed-width cavities were screened to estimate which cavities could couple both OH and CH stretches in the case of alcohols, and the OH stretch in the case of water. This estimation was later confirmed by measuring the infrared spectrum of said cavity upon injection at different angles of incidence (0-30°).

In some cases, this analysis did not suffice to unambiguously describe the coupling of both bands, owing to noisy baselines within the fixed-width cavities. As it is known that the Rabi splitting is proportional to the concentration of the solution, we additionally performed titrations with deuterated solvents (D_2O, methanol-*d*$_4$, or CD_2Cl_2 were selected because they do not display any significant vibrational bands in the same regions). Using this technique, the coupling of OH and CH stretches could be clearly determined.

Finally, for *n*-butanol and *tert*-butanol, the region below the IR cutoff of our fixed-width cavities (2000 cm^{-1}) was investigated by mounting a cavity of the same free spectral range (FSR, the energy difference between two consecutive cavity modes) as the different fixed-width cavities using the classical setup previously described for VSC studies.[5] When a satisfactory overlap was obtained, we looked at the region below 2000 cm^{-1} and the coupling of the various bands present in that area was estimated based on the shape of the spectra.

N1 (Methanol, C5)

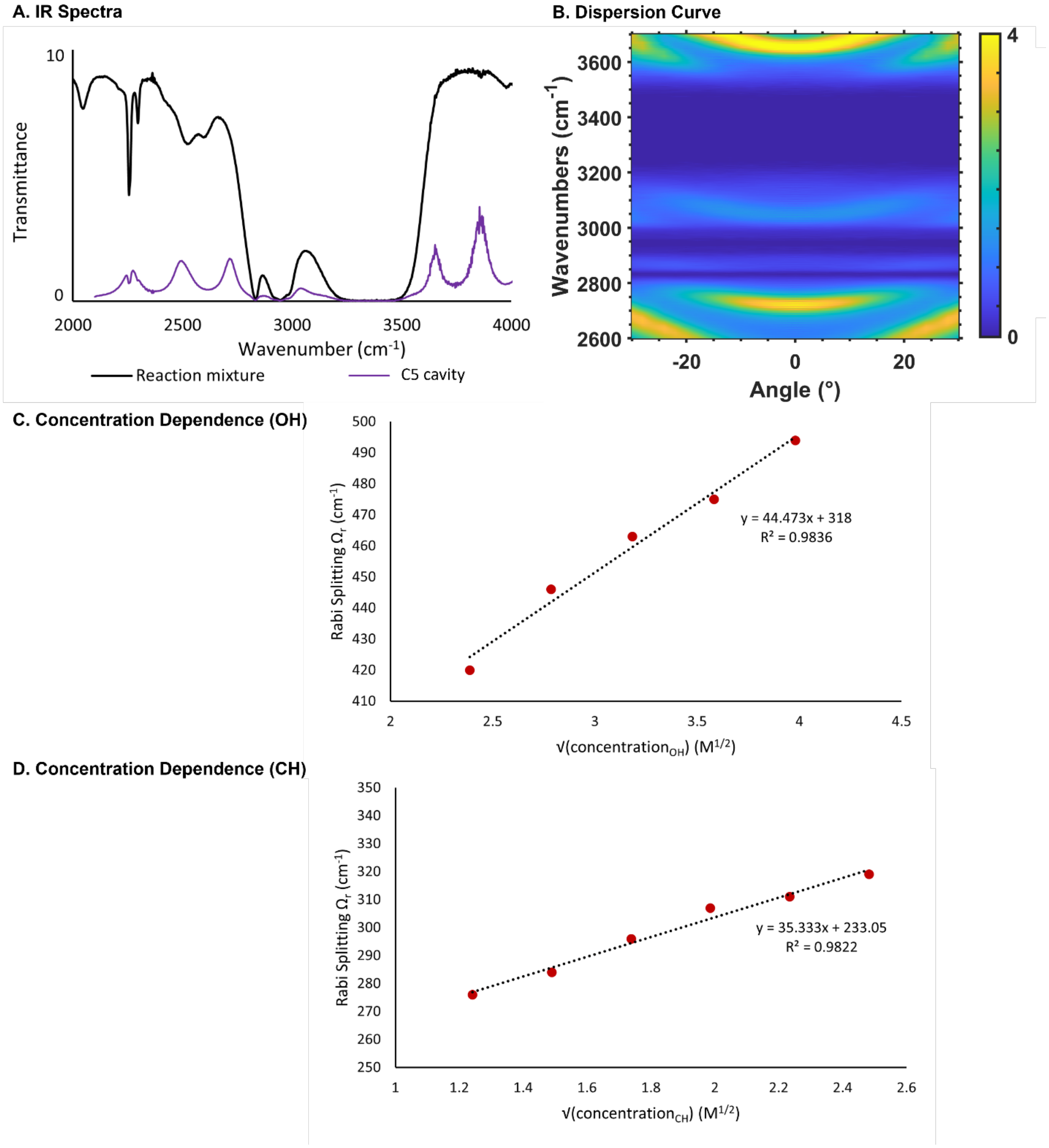

N2 (Ethanol, C5)

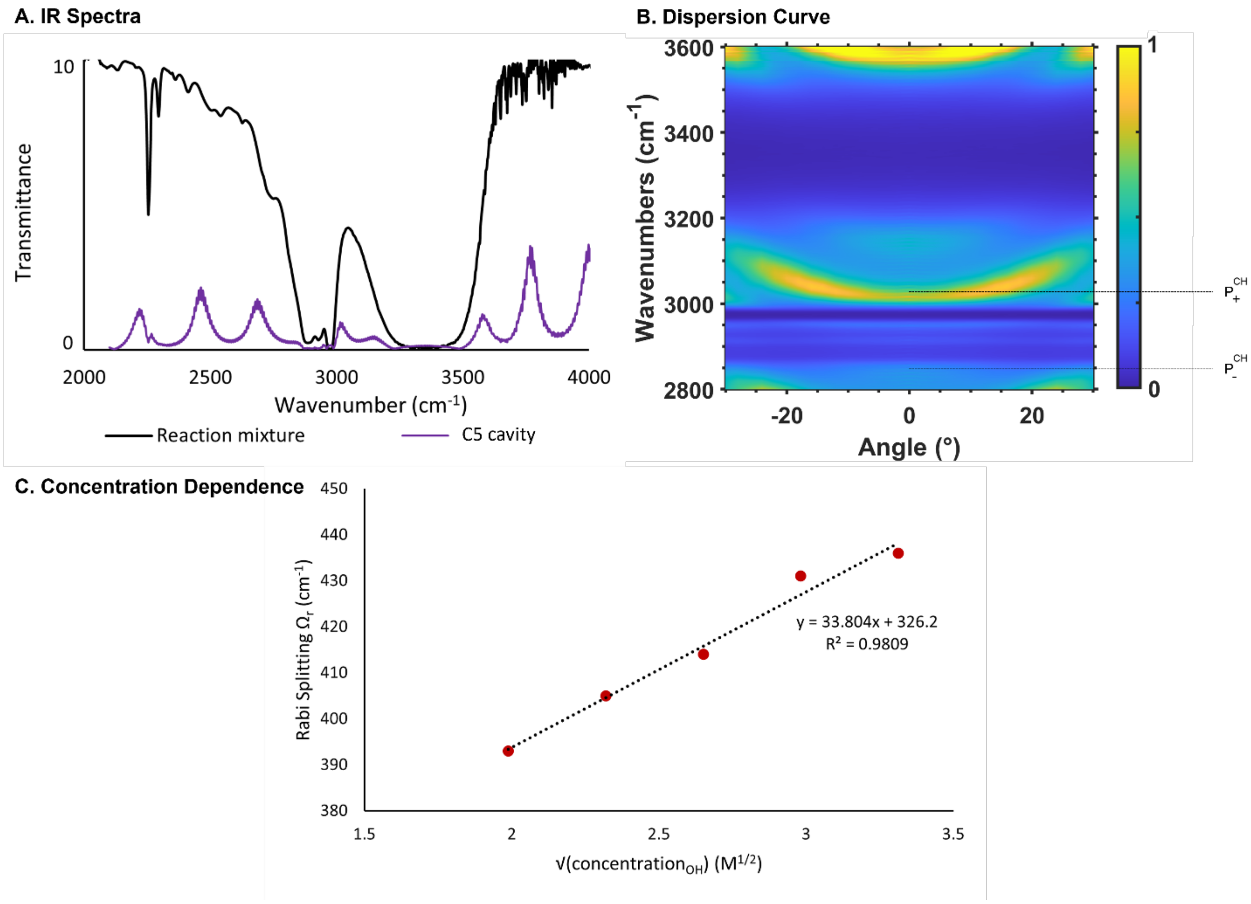

N3 (*n*-butanol, C5)

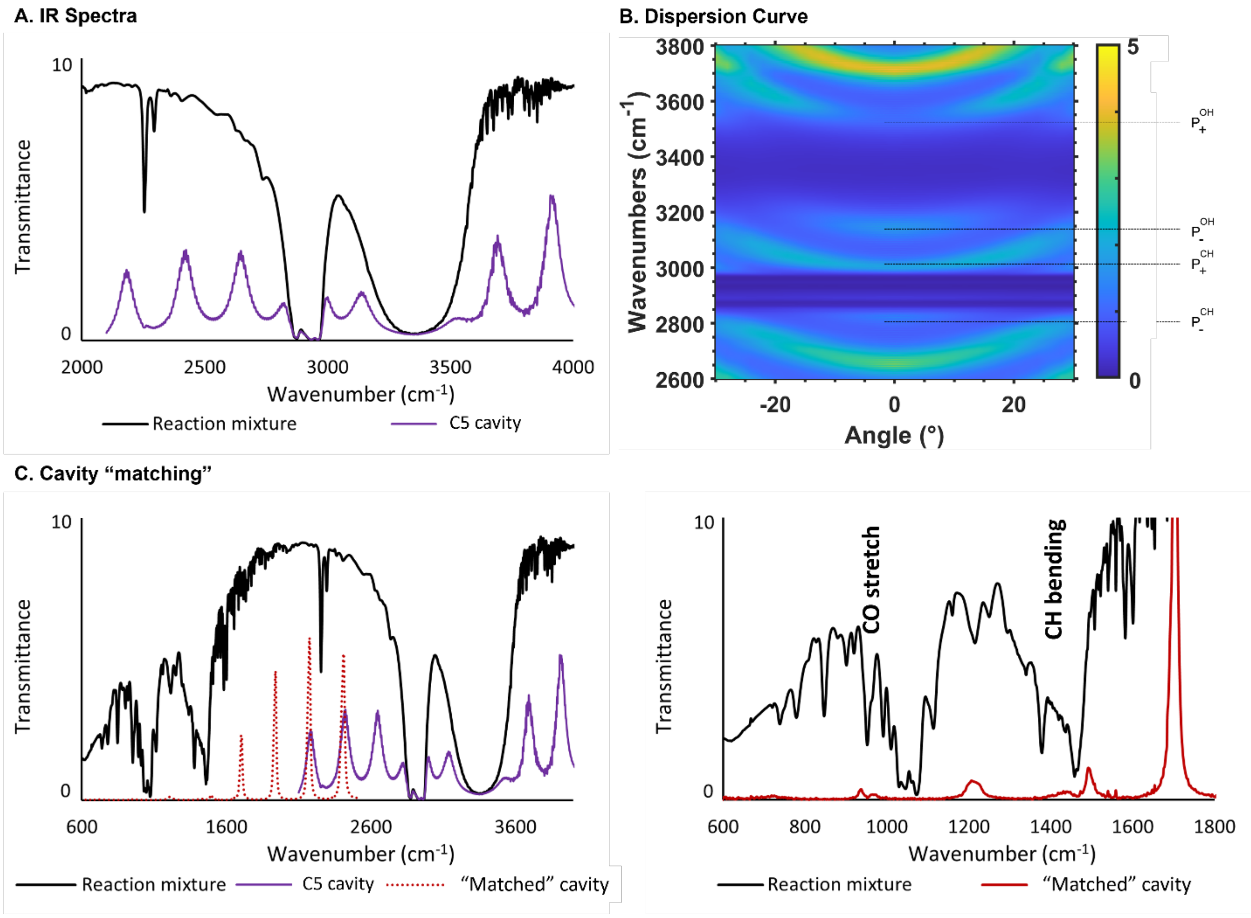

N3 (*n*-butanol, C4)

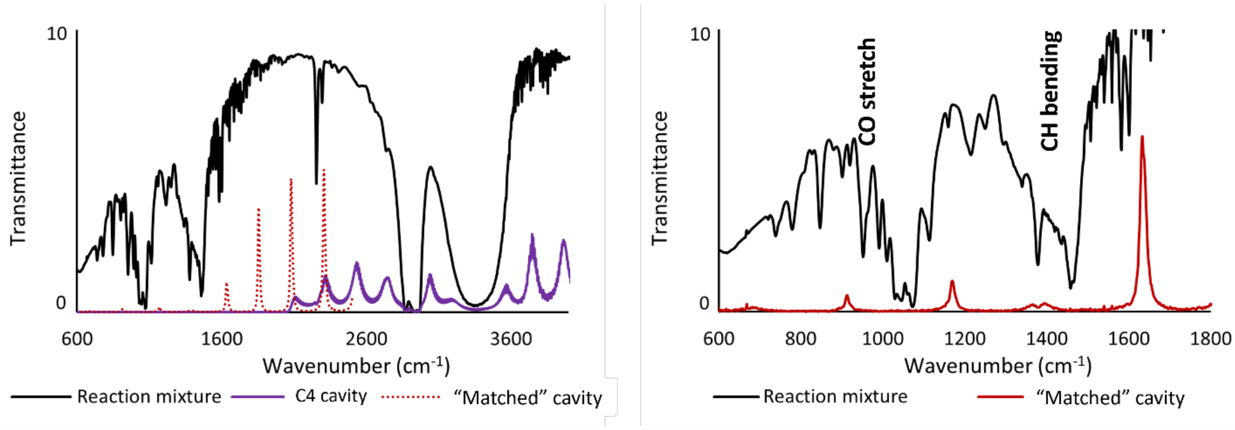

N3 (*n*-butanol, C3)

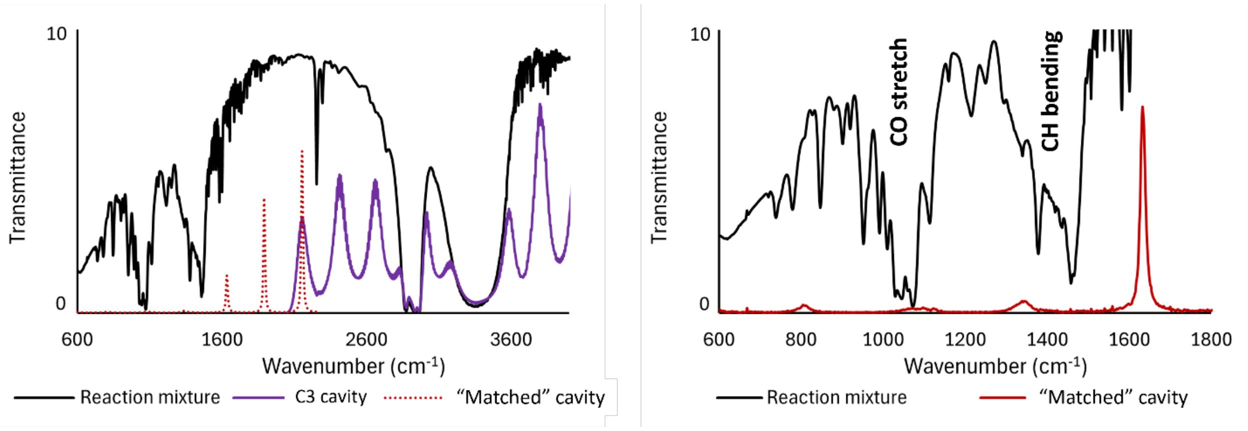

N3 (*n*-butanol, C2)

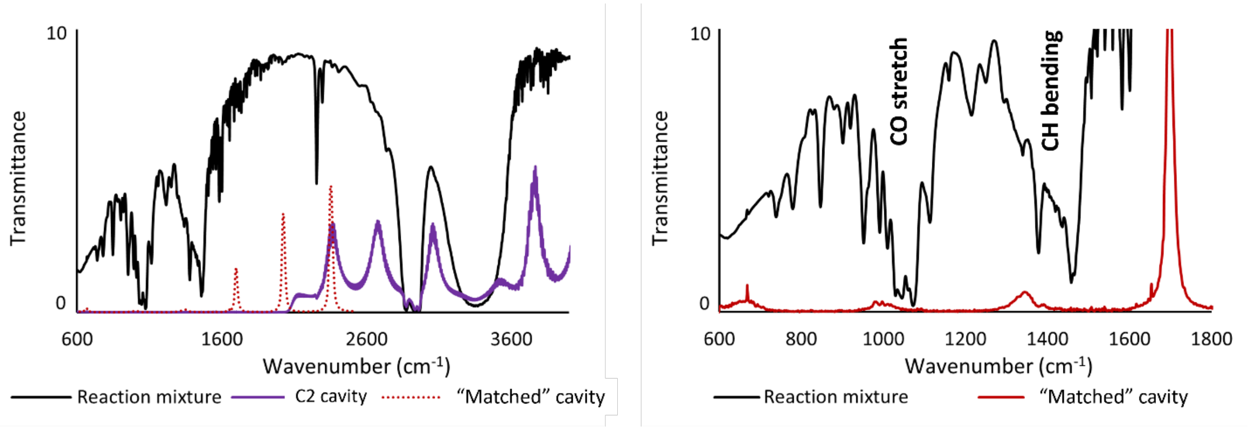

N4 (Nonanol, C5)

A. IR Spectra

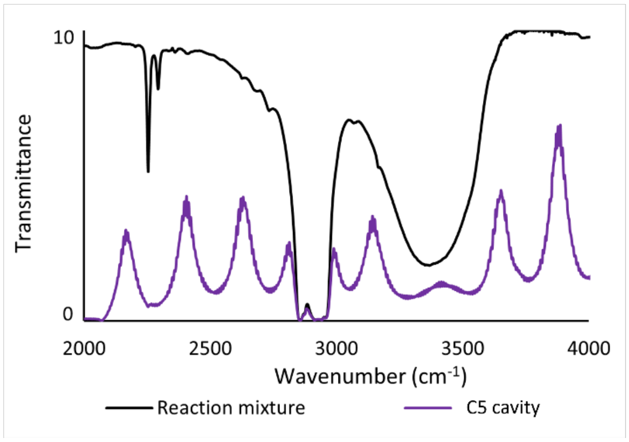

B. Dispersion Curve

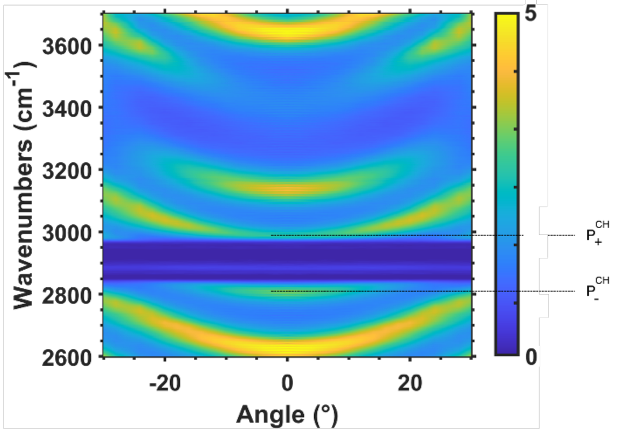

N5 (*iso*-propanol, C5)

A. IR Spectra

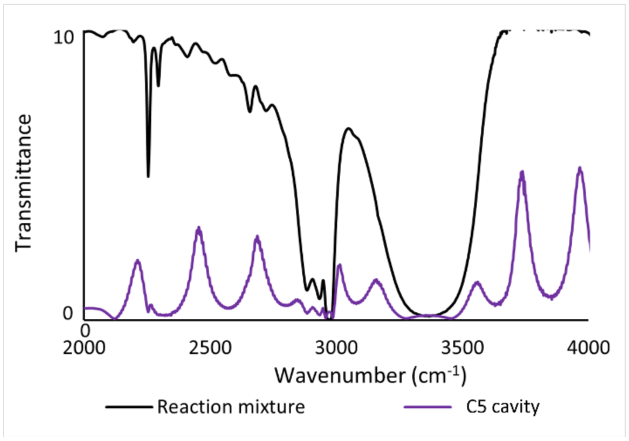

B. Dispersion Curve

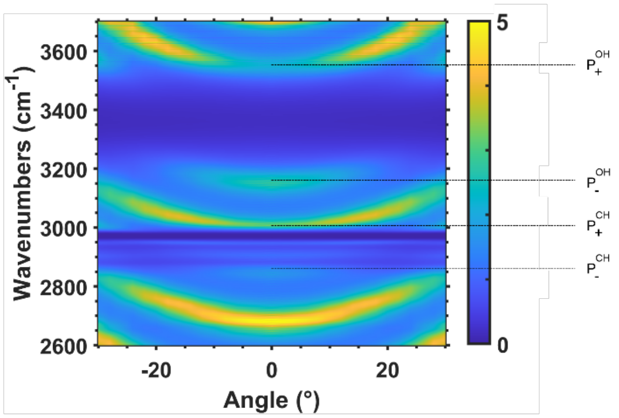

N6 (*iso*-butanol, C5)

A. IR Spectra

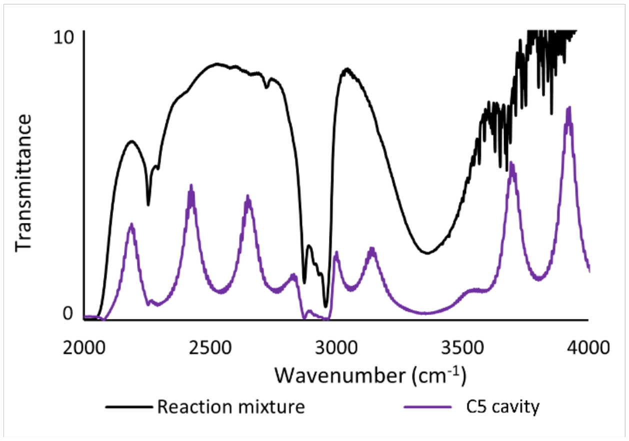

B. Dispersion Curve

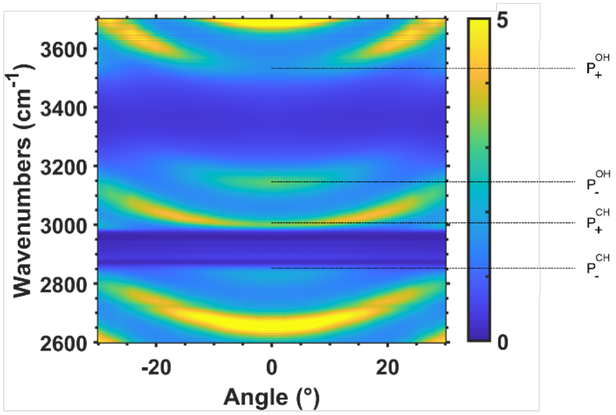

N7 (*cyclo*-butanol, C4)

A. IR Spectra

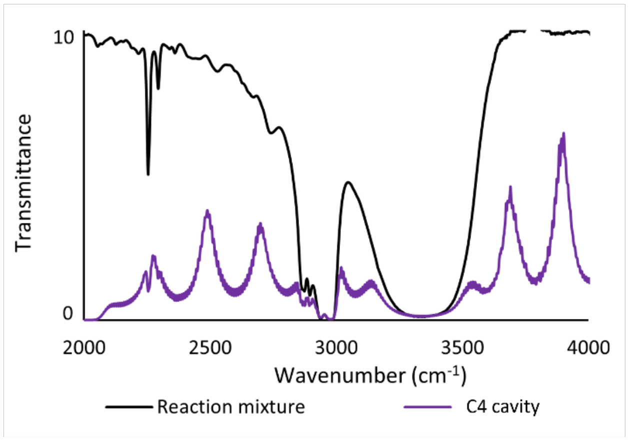

B. Dispersion Curve

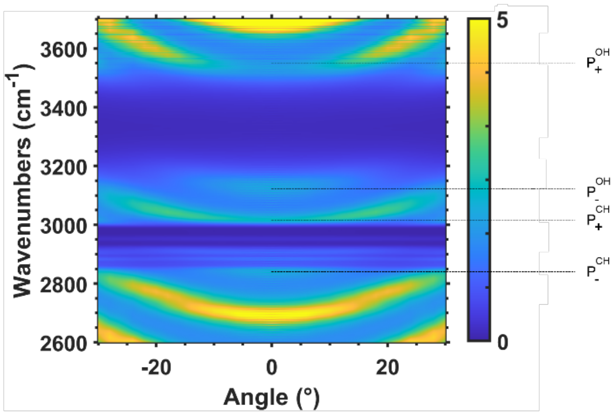

N8 (*tert*-butanol, C5)

A. IR Spectra

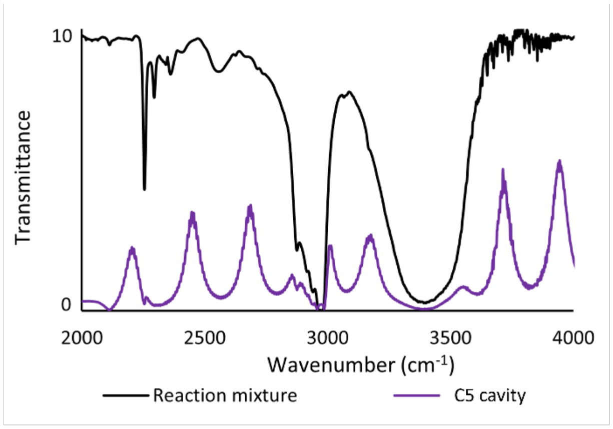

B. Dispersion Curve

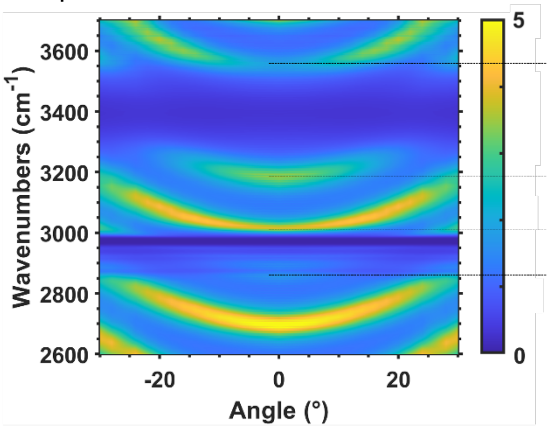

C. Cavity "matching"

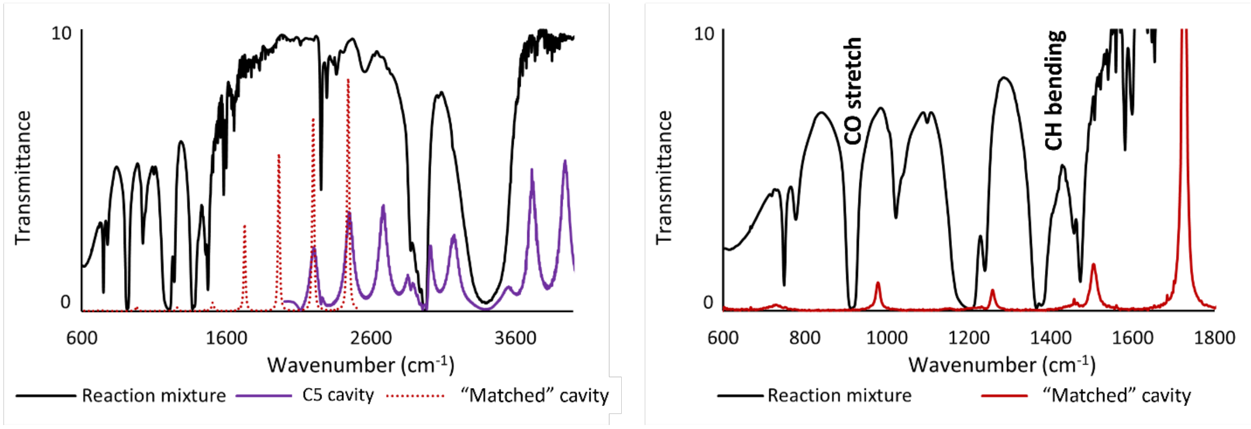

N8 (*tert*-butanol, C3)

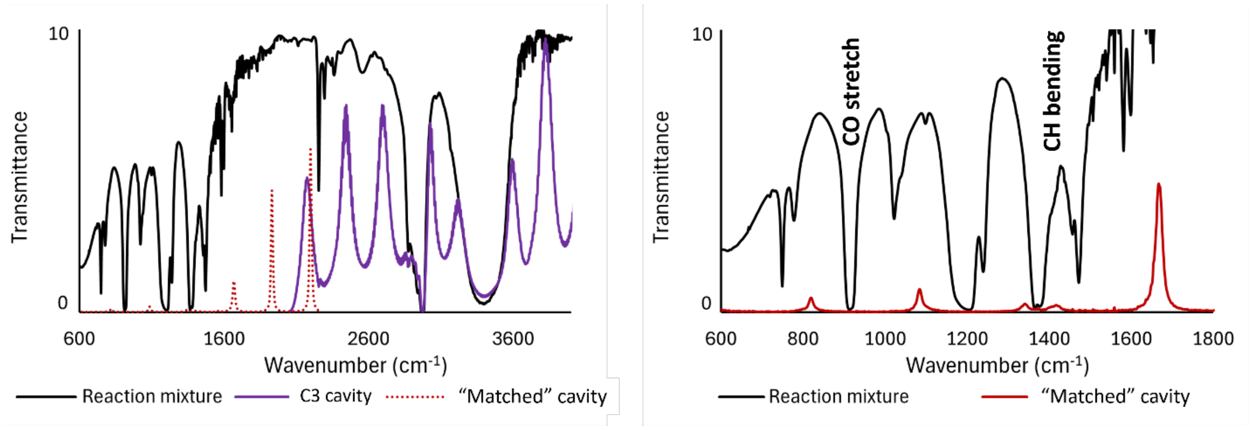

N8 (*tert*-butanol, C2)

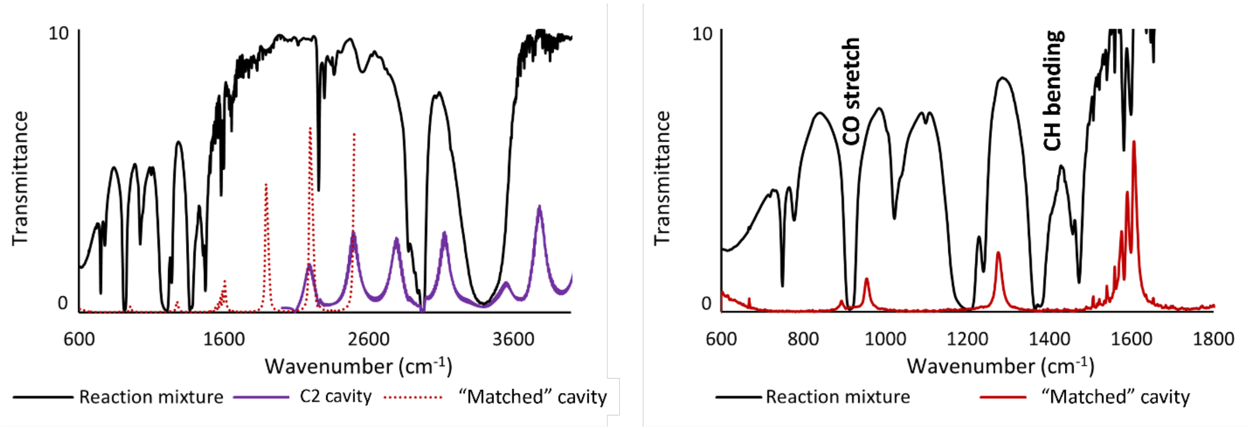

N8 (*tert*-butanol, C1)

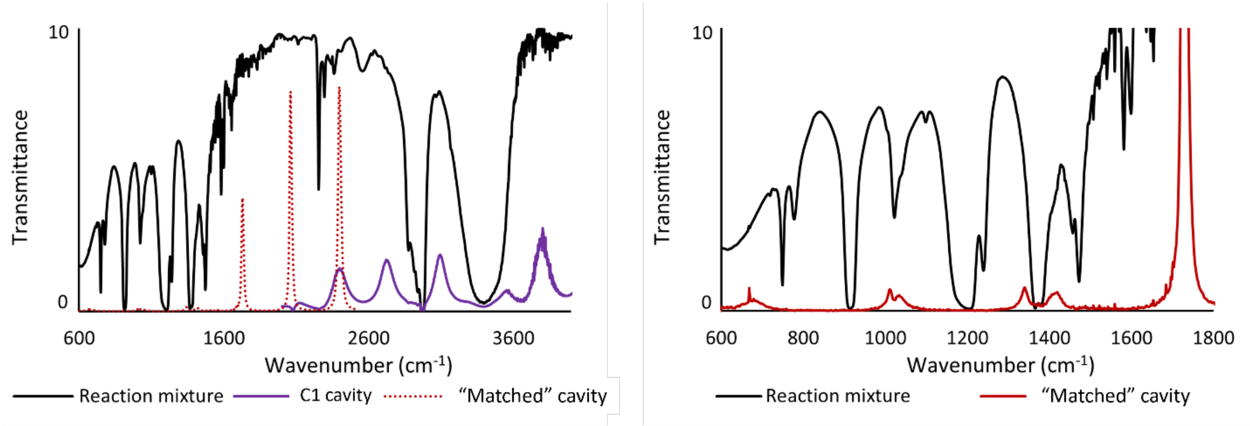

N9 (3-ethyl-3-pentanol, C5)

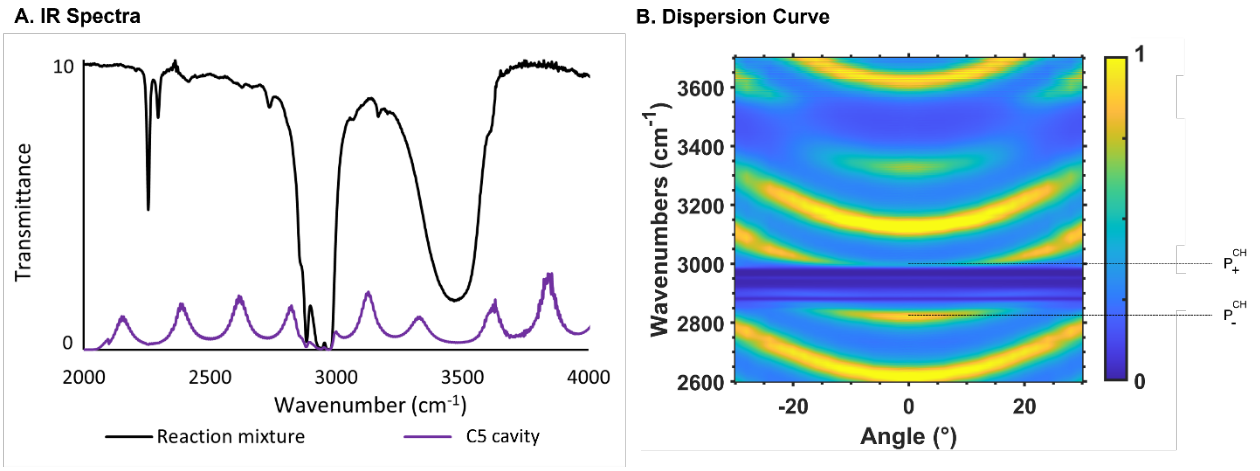

N10 (Benzyl Alcohol, C5)

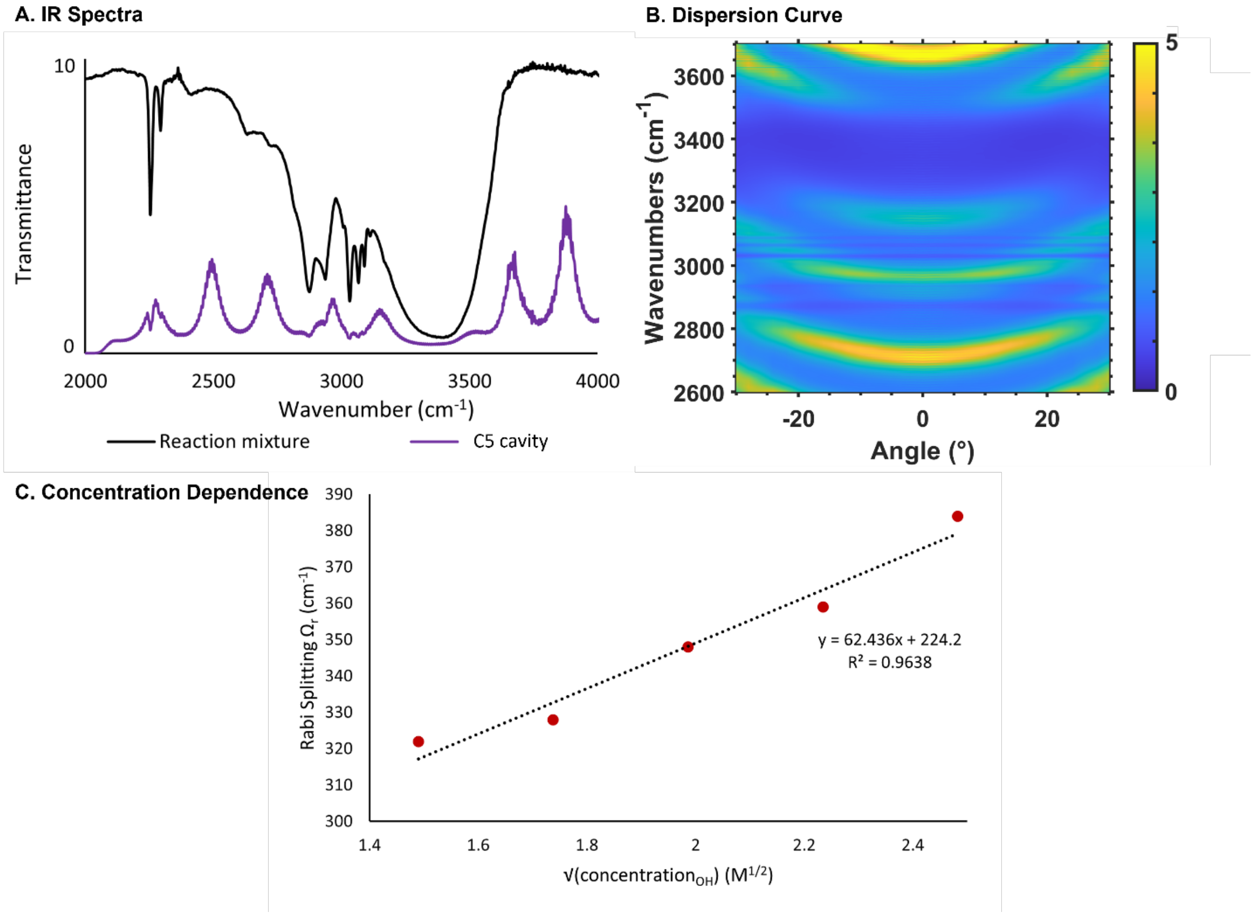

N11 (Water, C5)

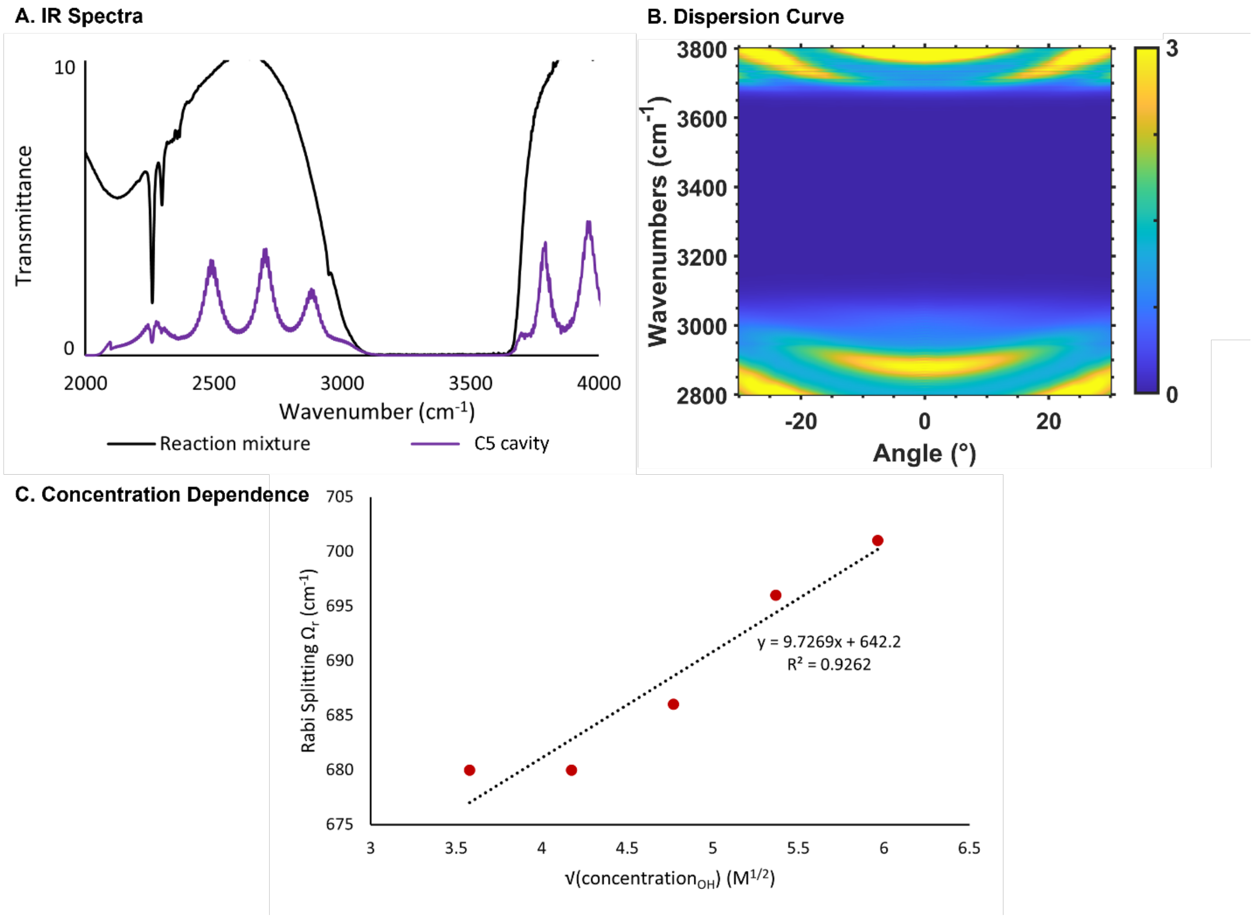
A. IR Spectra
10
0
Transmittance
2000
2500
3000
3500
4000
Wavenumber (cm-1)
Reaction mixture
C5 cavity
B. Dispersion Curve
3800
3600
3400
3200
3000
2800
Wavenumbers (cm-1)
-20
0
20
Angle (°)
3
0
C. Concentration Dependence
705
700
695
690
685
680
675
Rabi Splitting Ωr (cm-1)
y = 9.7269x + 642.2
R² = 0.9262
3
3.5
4
4.5
5
5.5
6
6.5
√(concentrationOH) (M1/2)

VII. Kinetics

Reaction of methanol (N1) with electrophile E1:

Cavity measurements (C5)

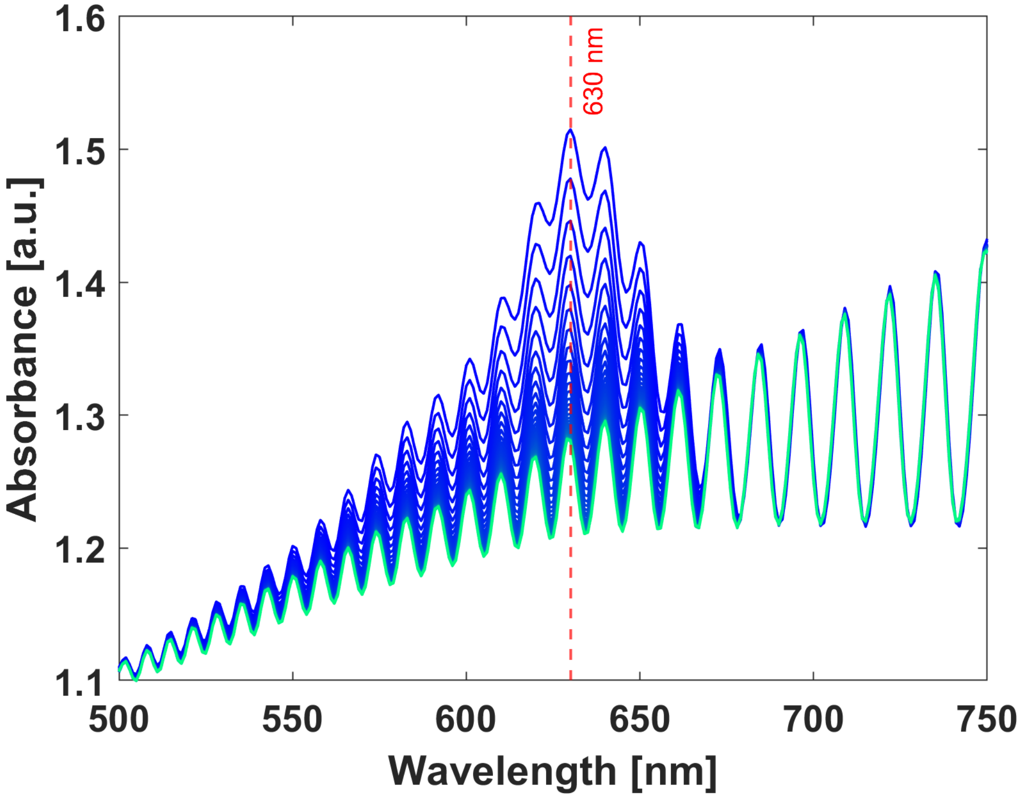

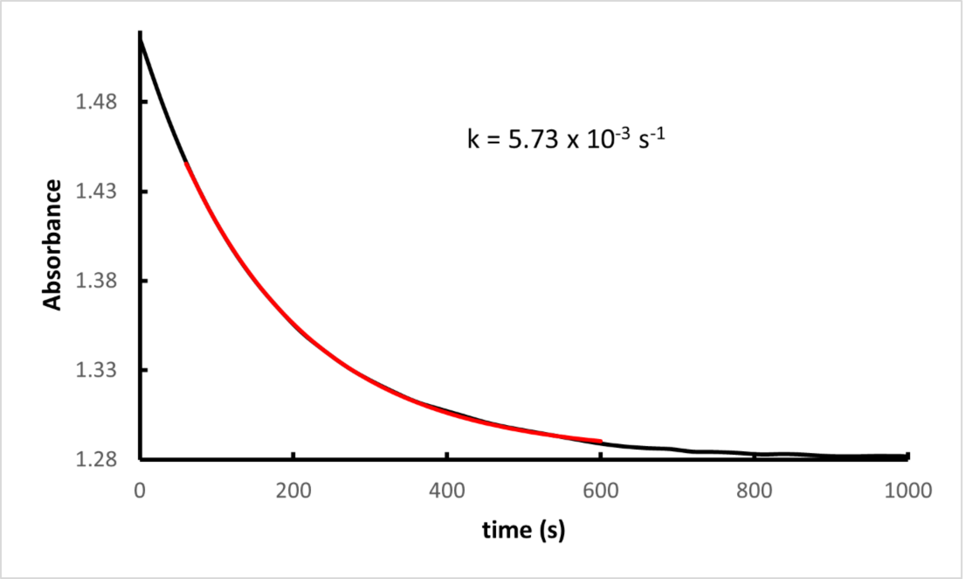

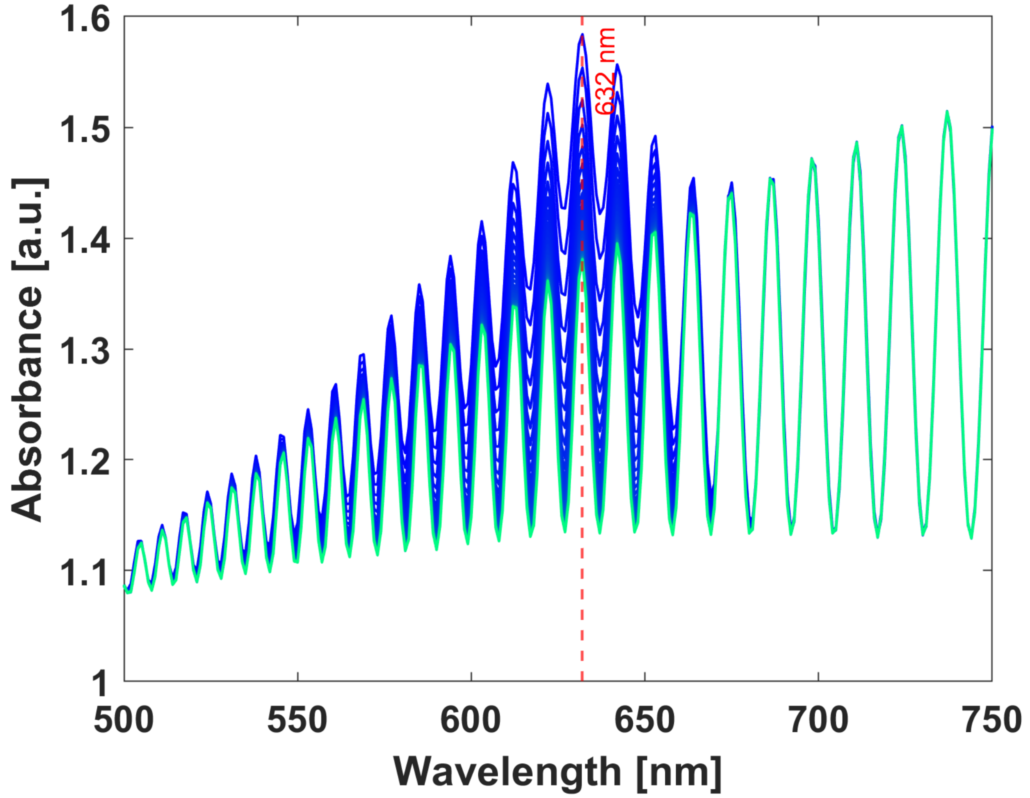
Absorbance [a.u.]
Wavelength [nm]
632 nm
1.6
1.5
1.4
1.3
1.2
1.1
1
500
550
600
650
700
750

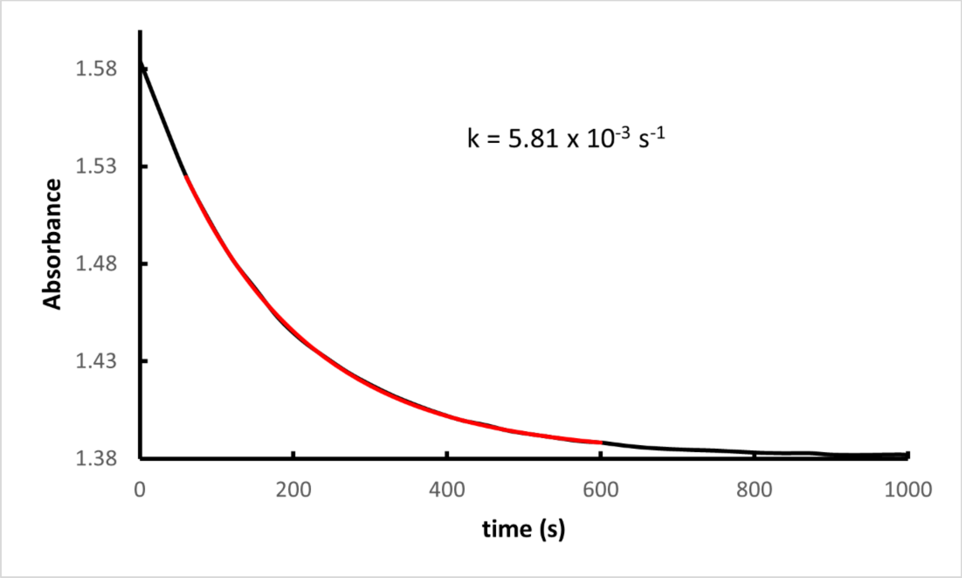
k = 5.81 x 10-3 s-1
Absorbance
time (s)
1.58
1.53
1.48
1.43
1.38
0
200
400
600
800
1000

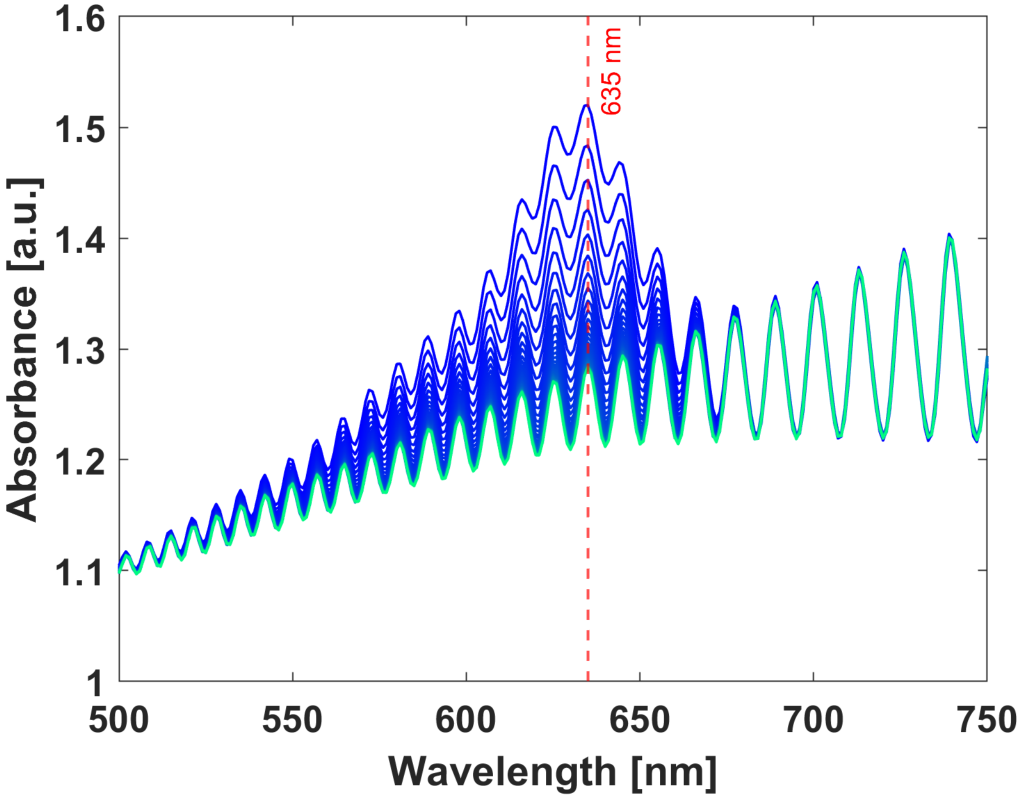
635 nm
Absorbance [a.u.]
Wavelength [nm]
1.6
1.5
1.4
1.3
1.2
1.1
1
500
550
600
650
700
750

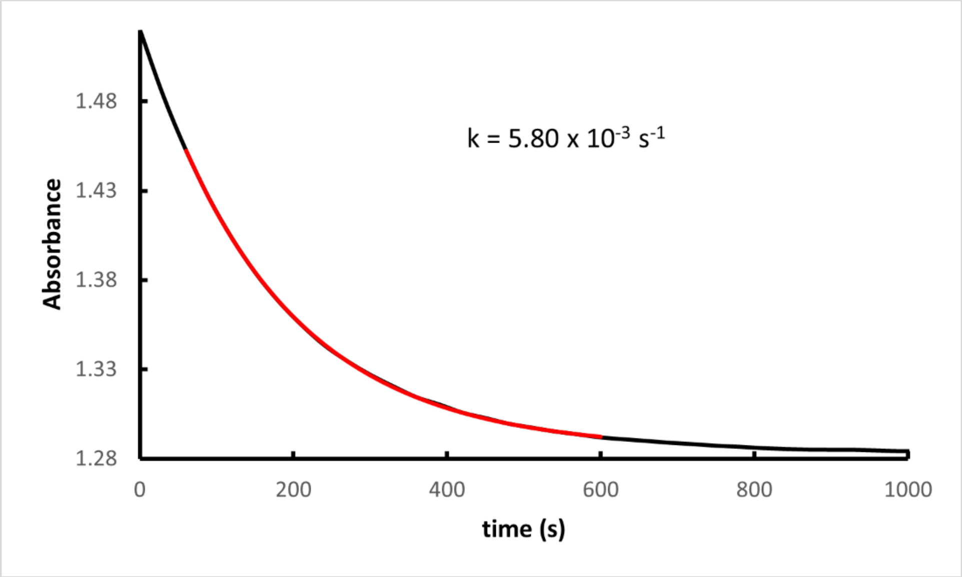
k = 5.80 x 10-3 s-1
Absorbance
time (s)
1.48
1.43
1.38
1.33
1.28
0
200
400
600
800
1000

Cell measurements (standard)

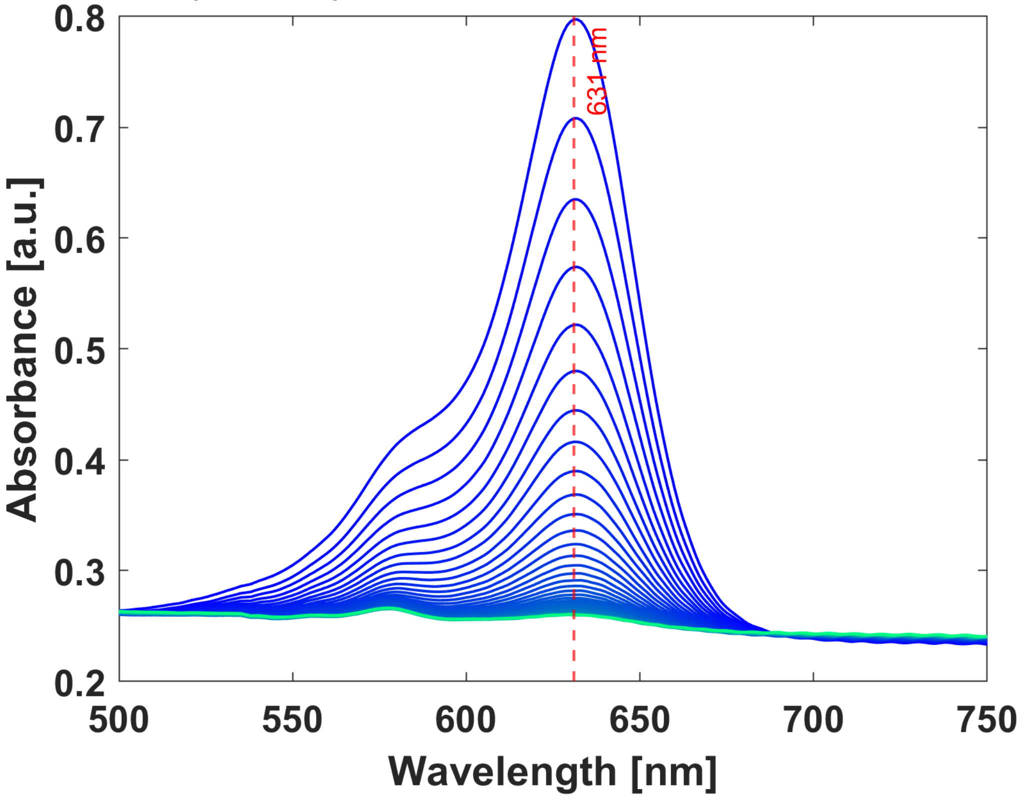

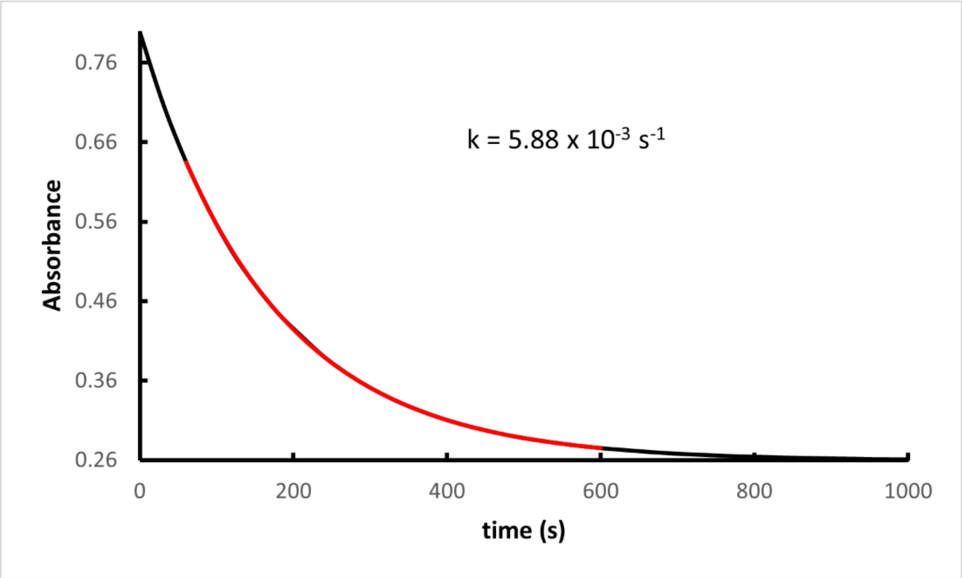

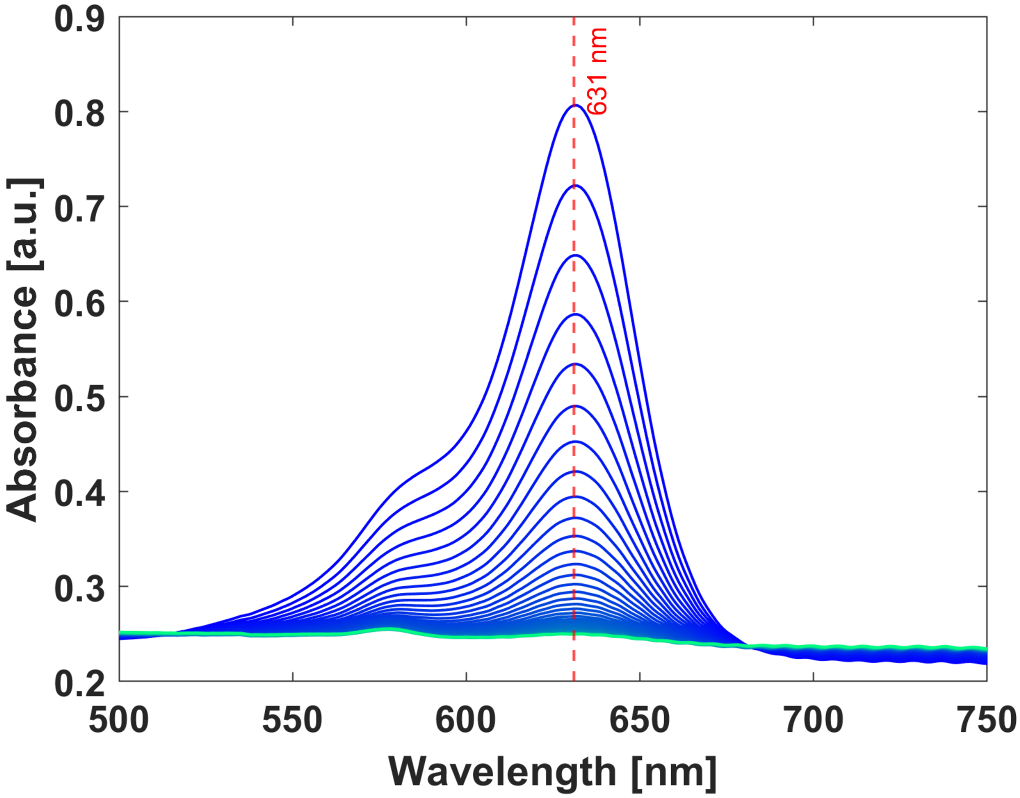
Absorbance [a.u.]
0.9
0.8
0.7
0.6
0.5
0.4
0.3
0.2
631 nm
500
550
600
650
700
750
Wavelength [nm]

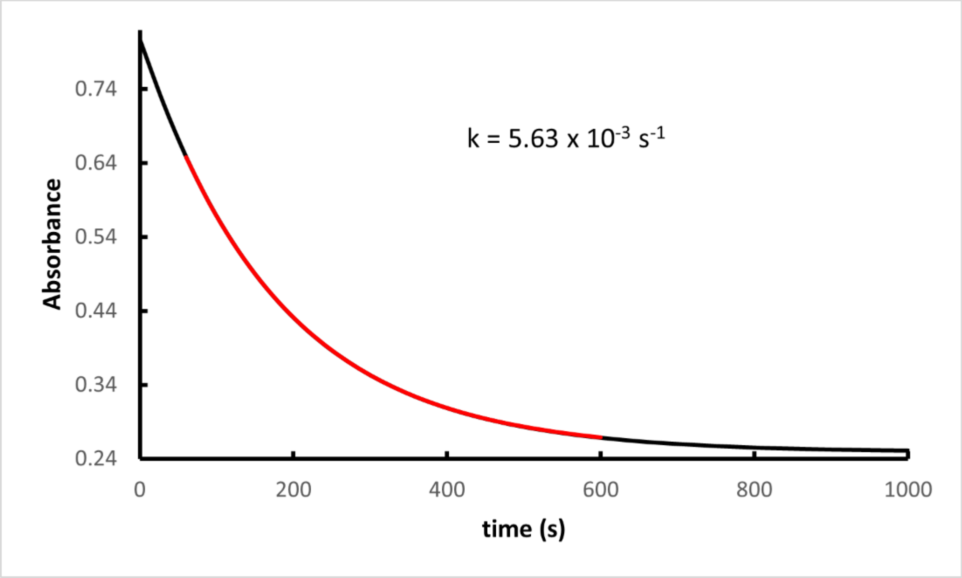
Absorbance
0.74
0.64
0.54
0.44
0.34
0.24
k = 5.63 x 10-3 s-1
0
200
400
600
800
1000
time (s)

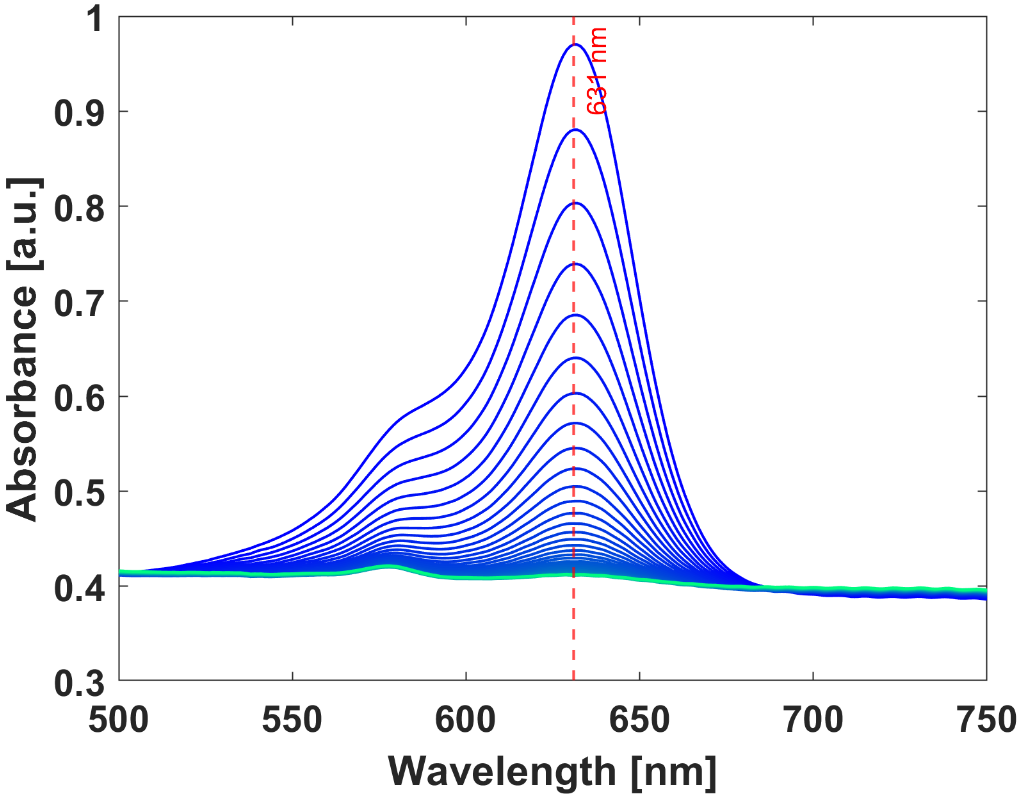
631 nm
Absorbance [a.u.]
Wavelength [nm]
1
0.9
0.8
0.7
0.6
0.5
0.4
0.3
500
550
600
650
700
750

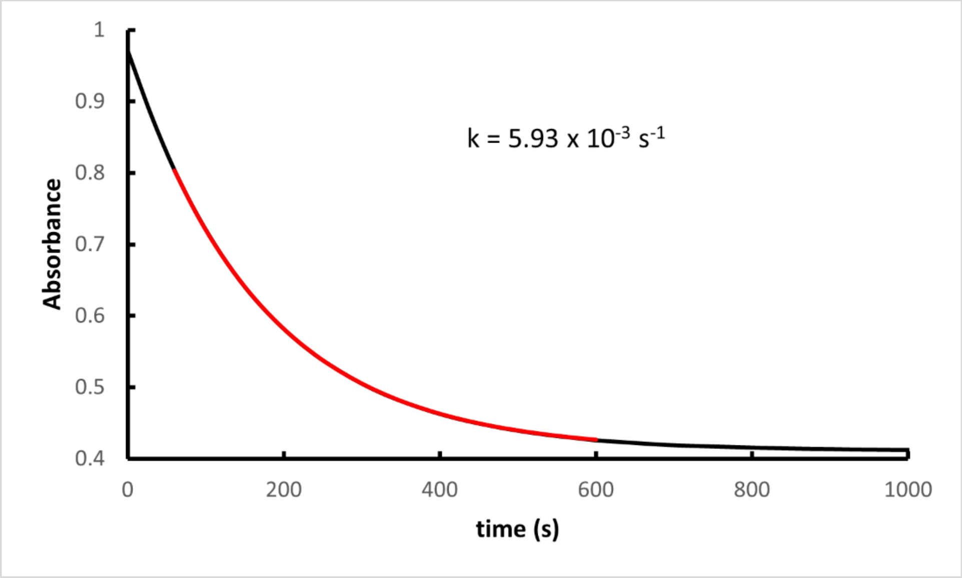
k = 5.93 x 10-3 s-1
Absorbance
time (s)
1
0.9
0.8
0.7
0.6
0.5
0.4
0
200
400
600
800
1000

Results

type	Rate (s^{-1})	Temperature	type	Rate (s^{-1})	Temperature
C5	5.73×10^{-3}	23.8	cell	5.92×10^{-3}	23.7
C5	5.81×10^{-3}	23.7	cell	5.60×10^{-3}	23.7
C5	5.80×10^{-3}	23.7	cell	5.93×10^{-3}	23.7
Average:	**5.78×10^{-3}**	23.7	Average:	**5.82×10^{-3}**	23.7
Standard error:	**2.50×10^{-5}**		Standard error:	**1.07×10^{-4}**	

Reaction of ethanol (N2) with electrophile E1:

Cavity measurements (C5)

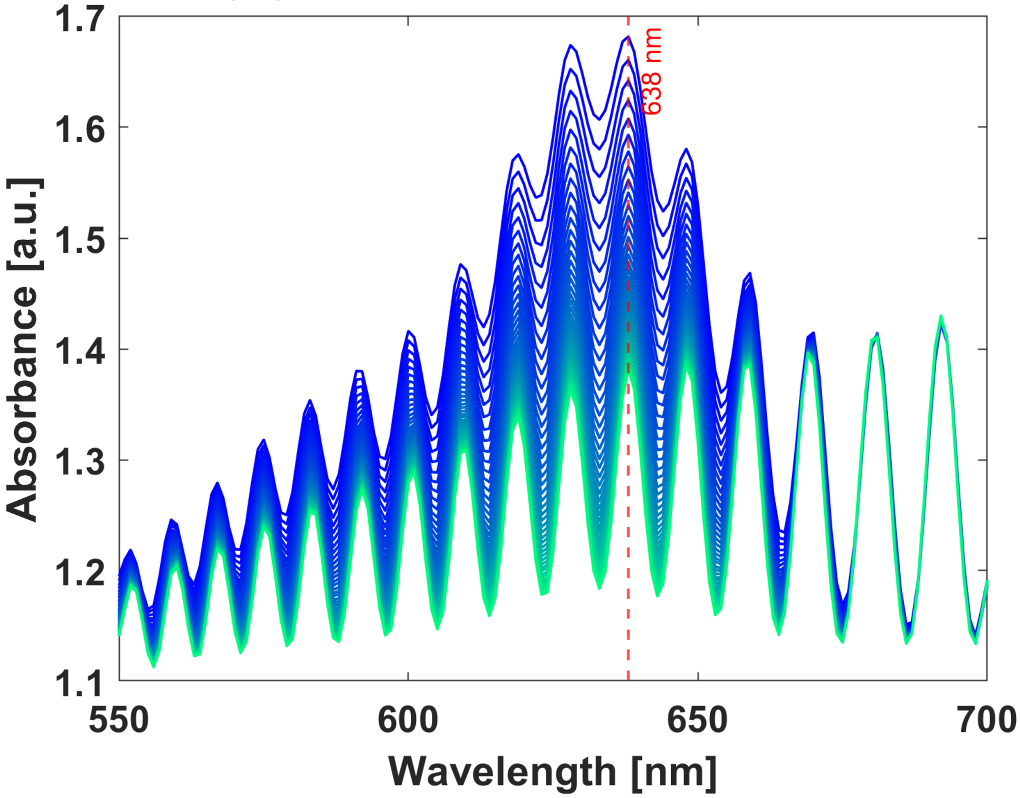

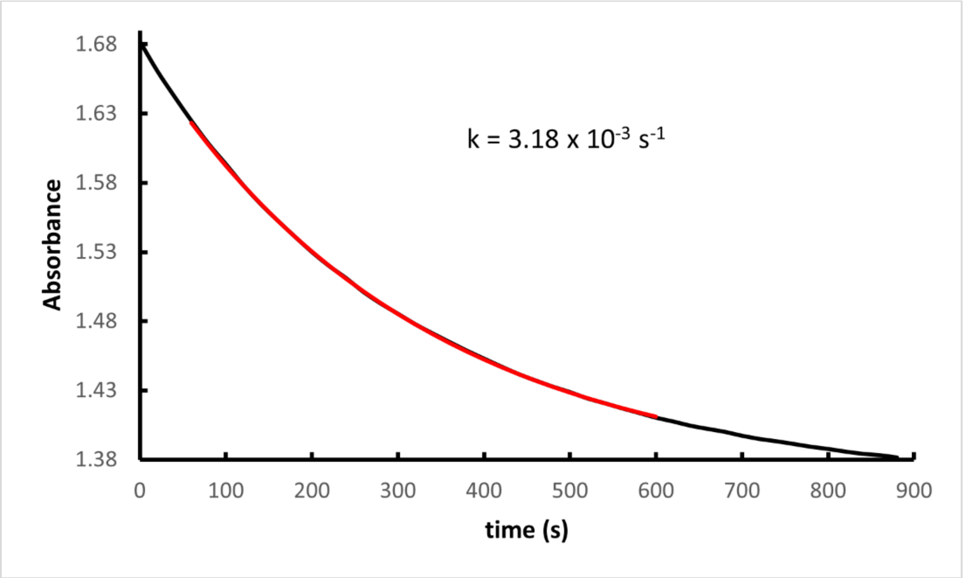

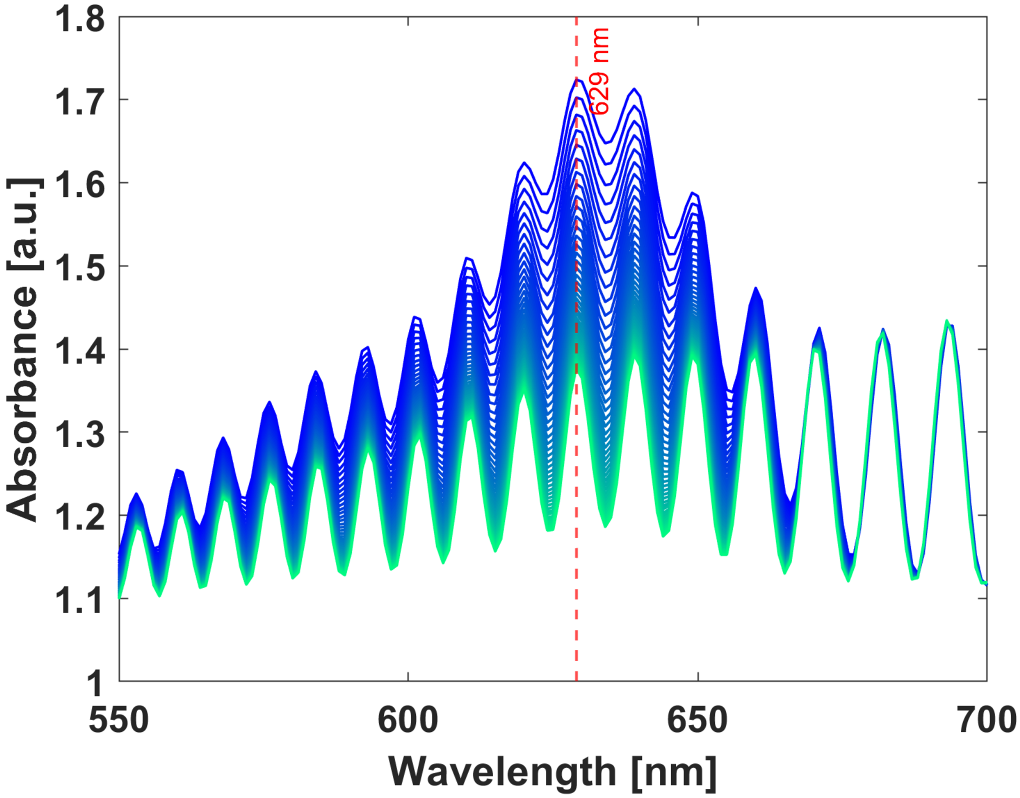
629 nm
Absorbance [a.u.]
Wavelength [nm]
1.8
1.7
1.6
1.5
1.4
1.3
1.2
1.1
1
550
600
650
700

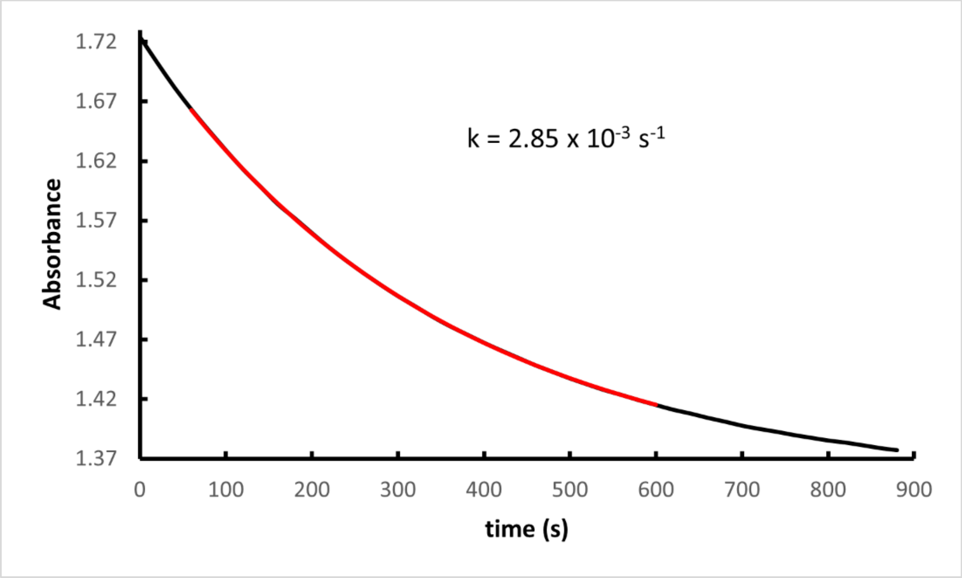
k = 2.85 x 10-3 s-1
Absorbance
time (s)
1.72
1.67
1.62
1.57
1.52
1.47
1.42
1.37
0
100
200
300
400
500
600
700
800
900

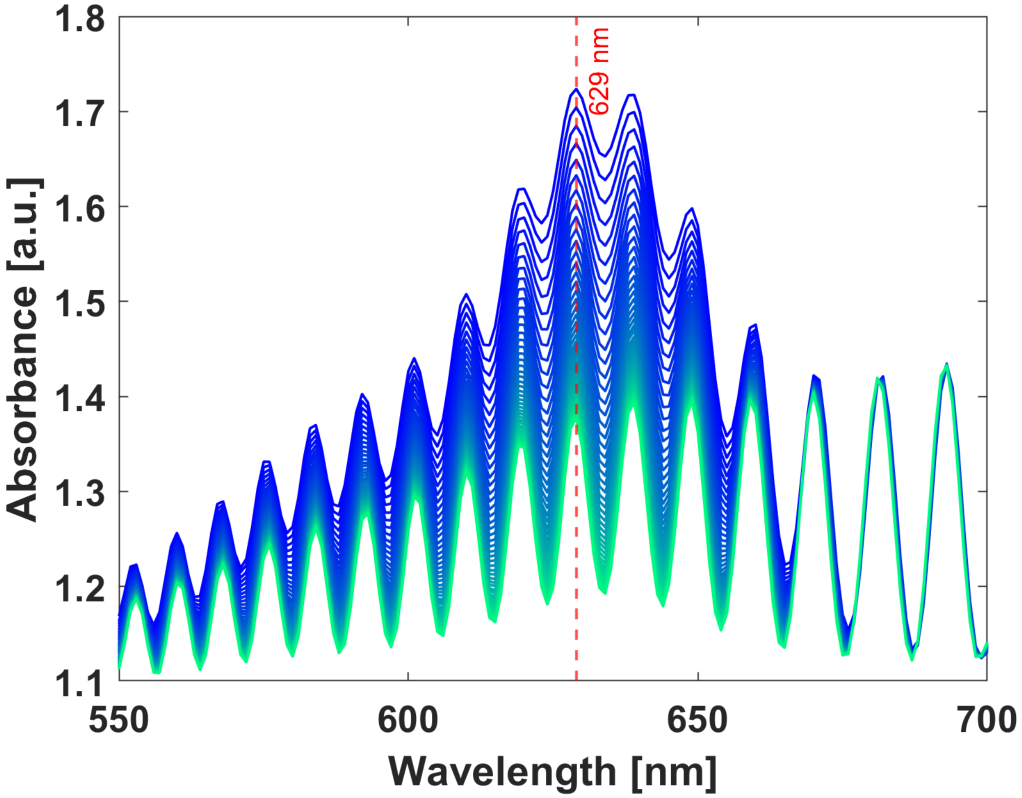
1.8
1.7
1.6
1.5
1.4
1.3
1.2
1.1
Absorbance [a.u.]
550
600
650
700
Wavelength [nm]
629 nm

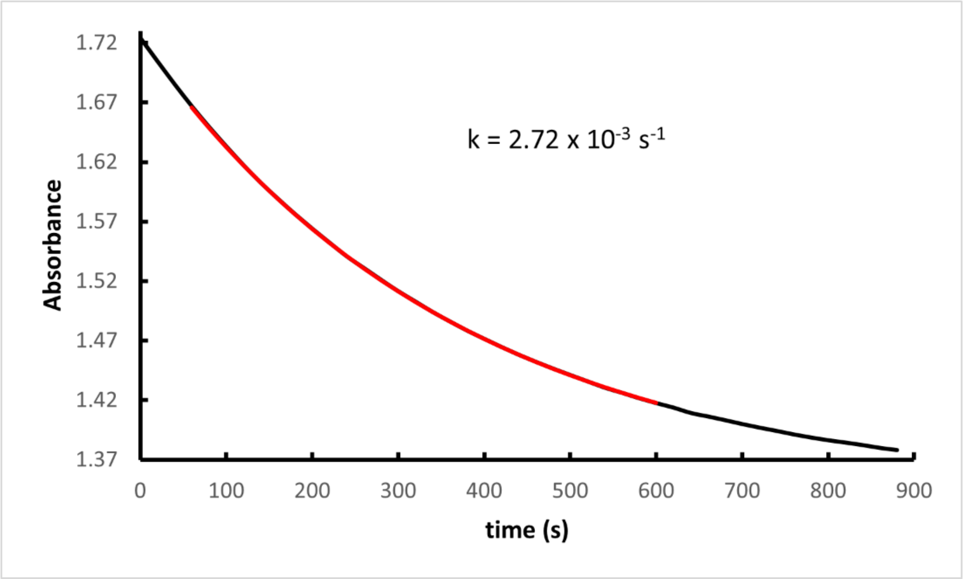
1.72
1.67
1.62
1.57
1.52
1.47
1.42
1.37
Absorbance
0
100
200
300
400
500
600
700
800
900
time (s)
k = 2.72 x 10-3 s-1

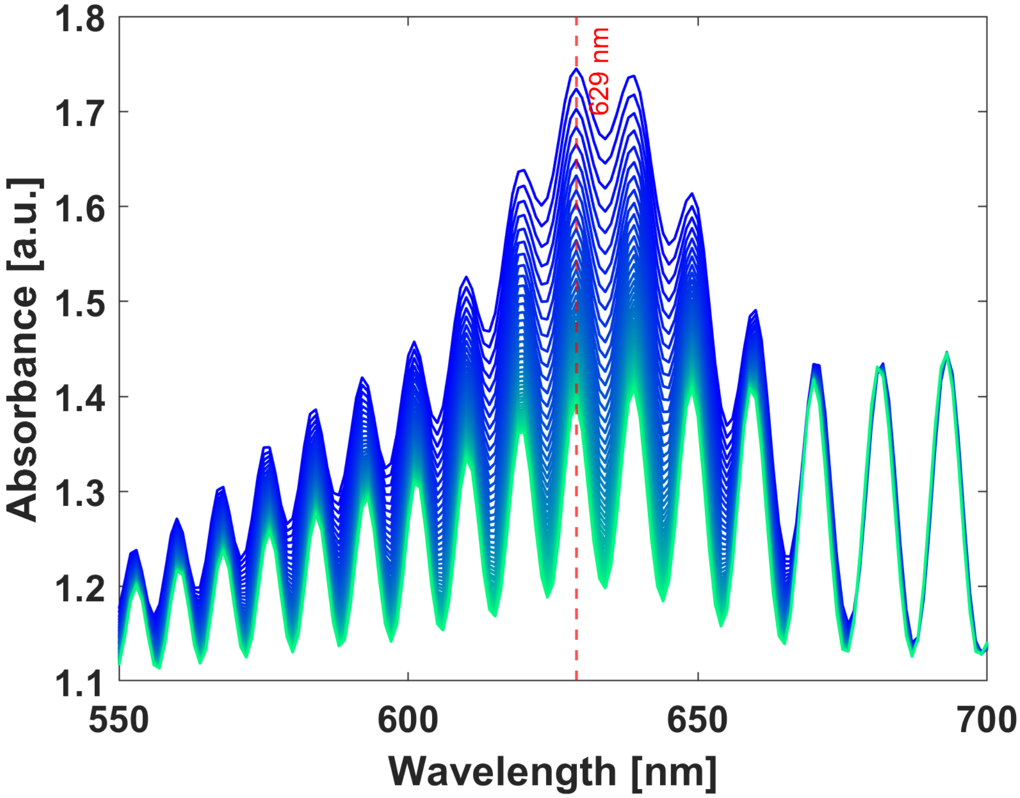

629 nm
Absorbance [a.u.]
Wavelength [nm]
1.8
1.7
1.6
1.5
1.4
1.3
1.2
1.1
550
600
650
700

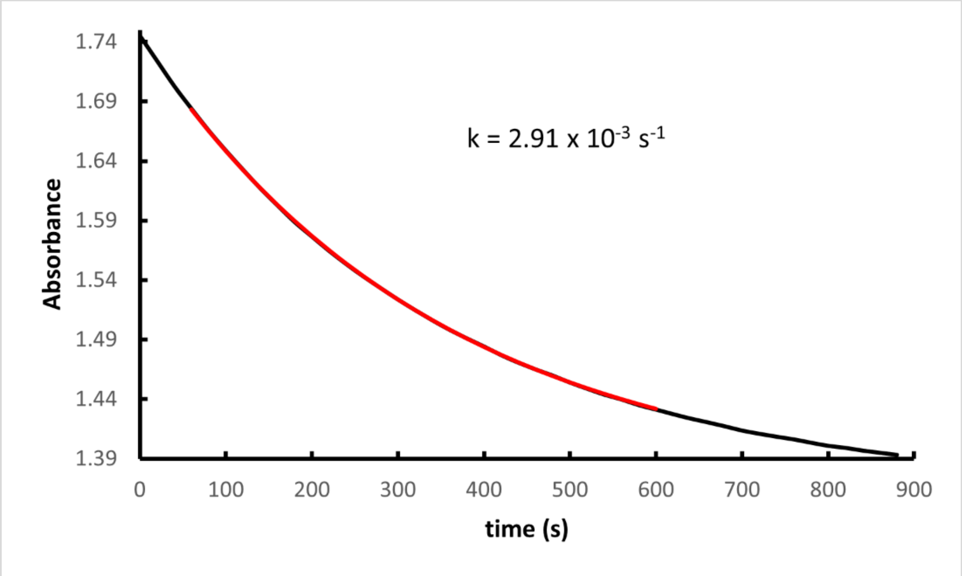

k = 2.91 x 10-3 s-1
Absorbance
time (s)
1.74
1.69
1.64
1.59
1.54
1.49
1.44
1.39
0
100
200
300
400
500
600
700
800
900

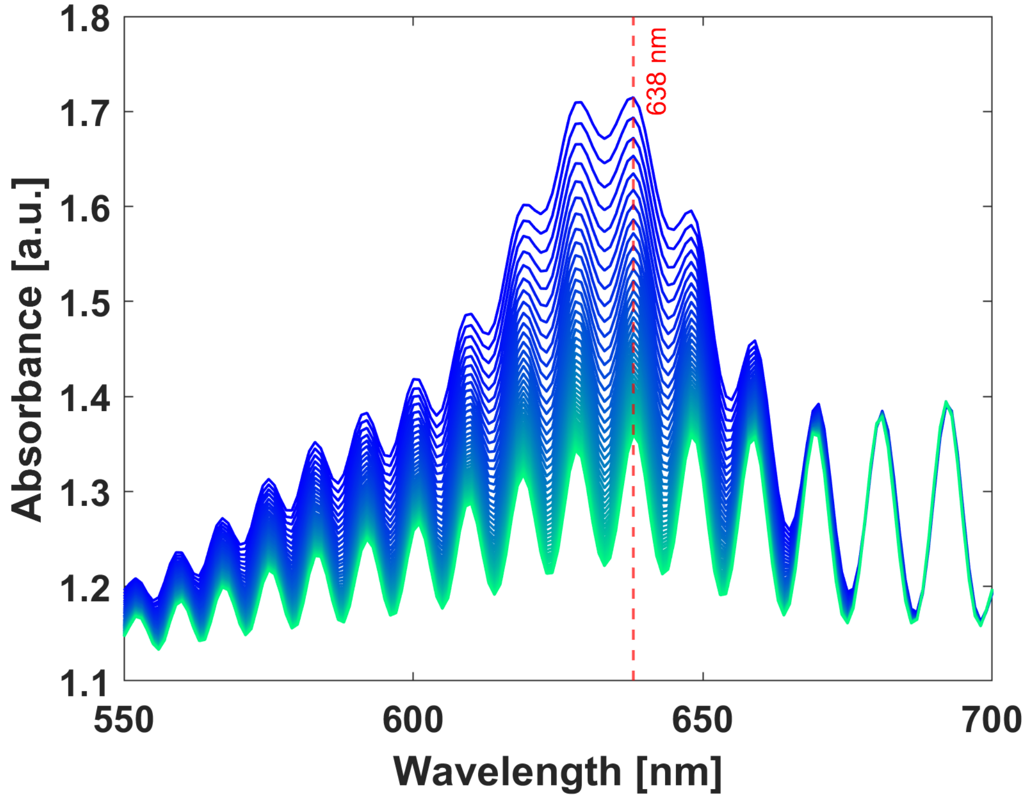

1.8
1.7
1.6
1.5
1.4
1.3
1.2
1.1
Absorbance [a.u.]
550
600
650
700
Wavelength [nm]
638 nm

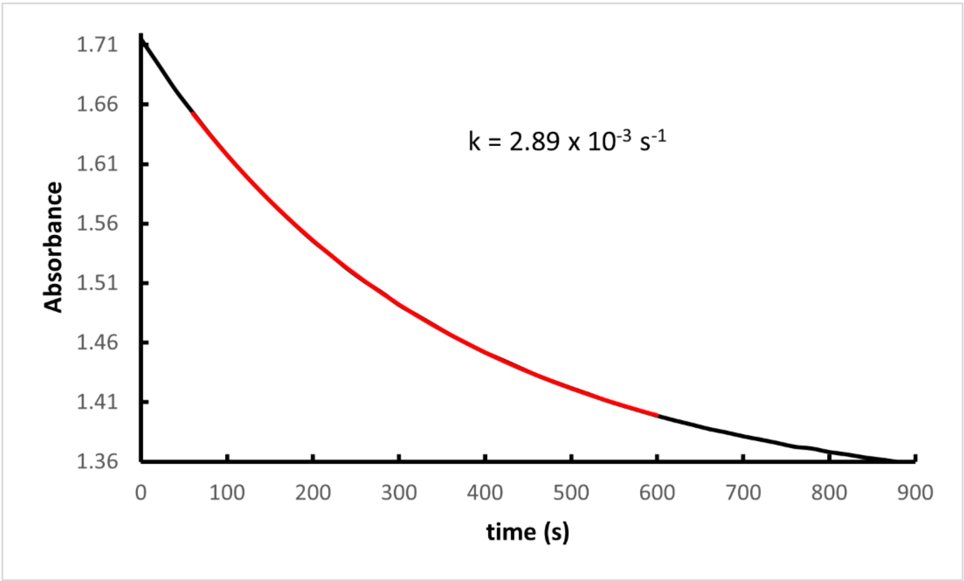

1.71
1.66
1.61
1.56
1.51
1.46
1.41
1.36
Absorbance
0
100
200
300
400
500
600
700
800
900
time (s)
k = 2.89 x 10-3 s-1

Cell measurements (standard)

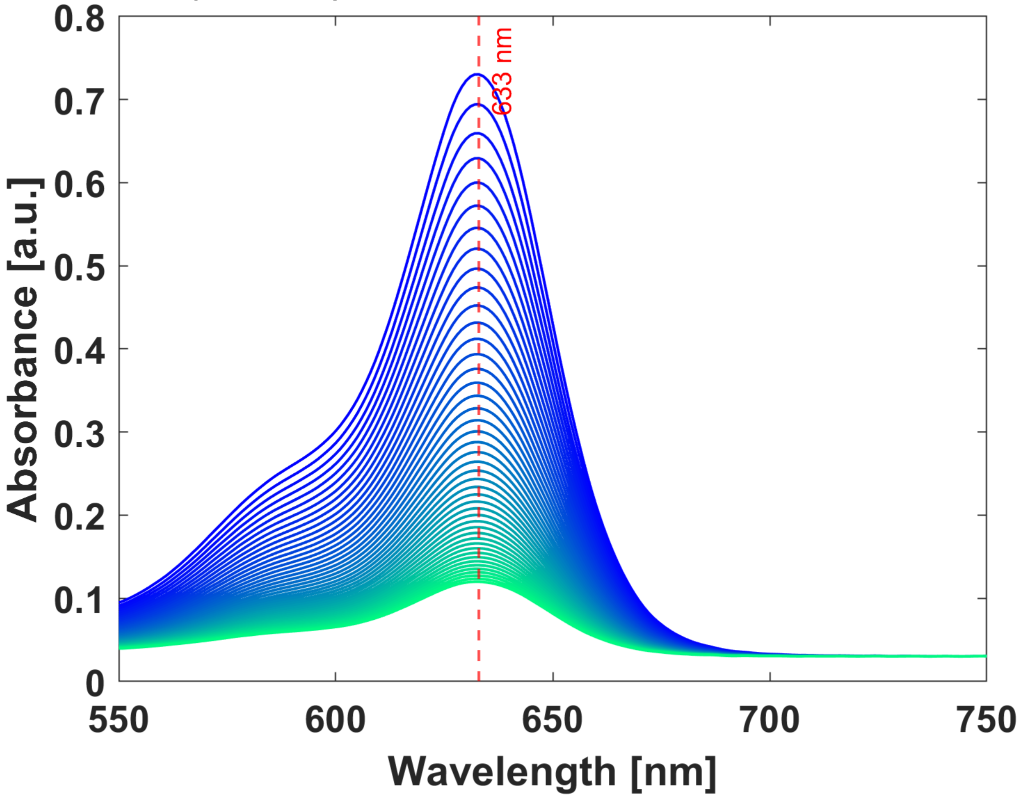

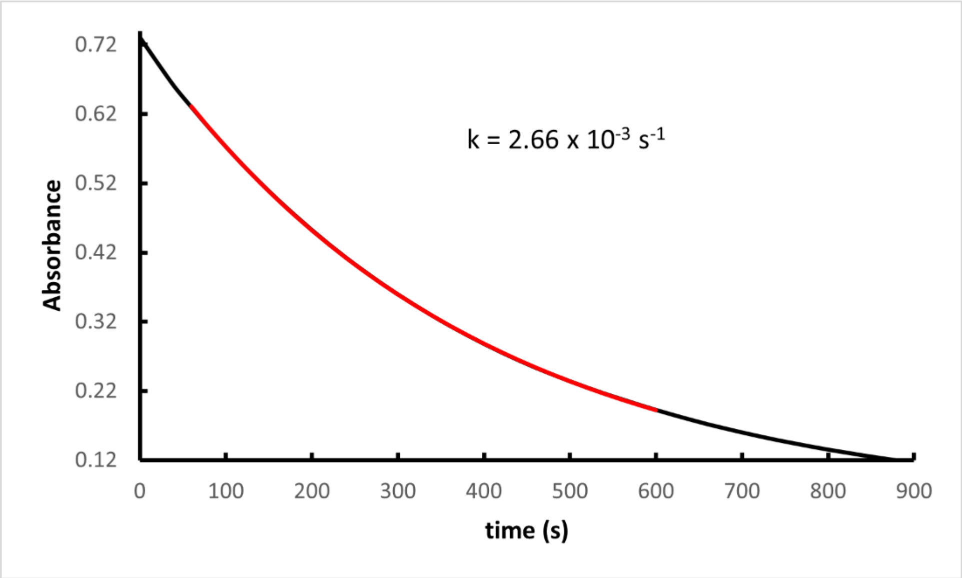

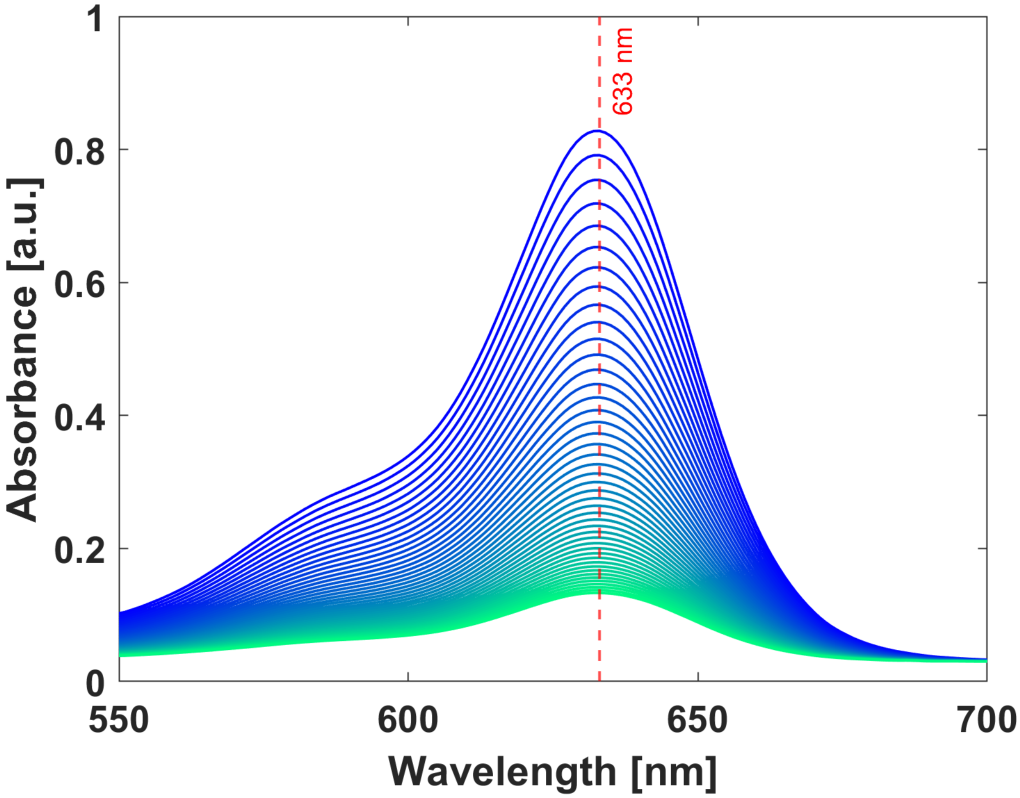
633 nm
Absorbance [a.u.]
Wavelength [nm]
1
0.8
0.6
0.4
0.2
0
550
600
650
700

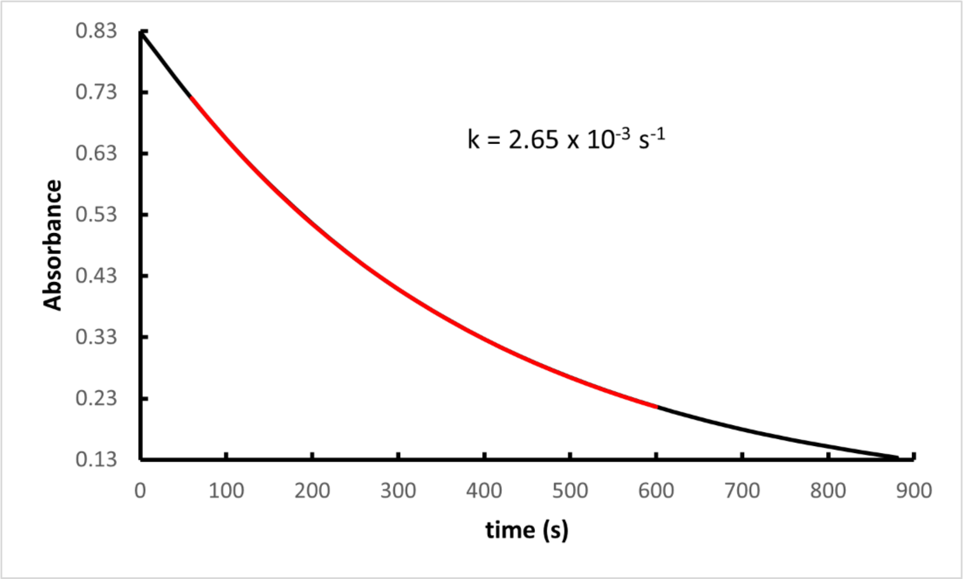
k = 2.65 x 10-3 s-1
Absorbance
time (s)
0.83
0.73
0.63
0.53
0.43
0.33
0.23
0.13
0
100
200
300
400
500
600
700
800
900

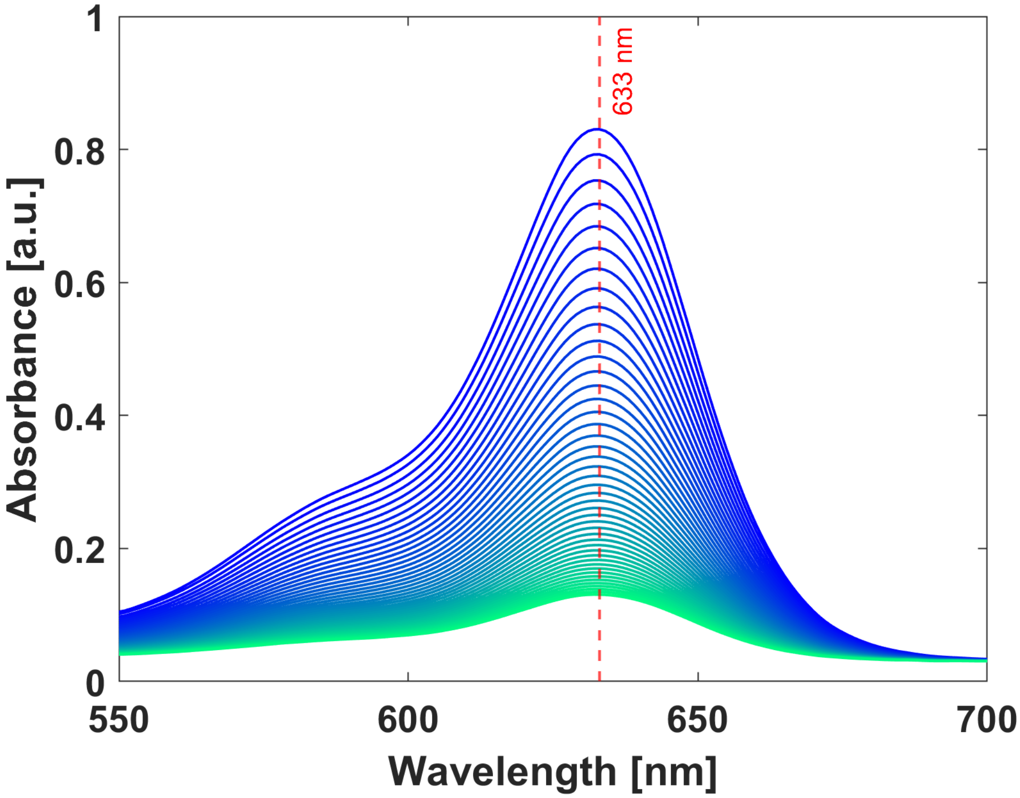

633 nm
Absorbance [a.u.]
Wavelength [nm]
0
0.2
0.4
0.6
0.8
1
550
600
650
700

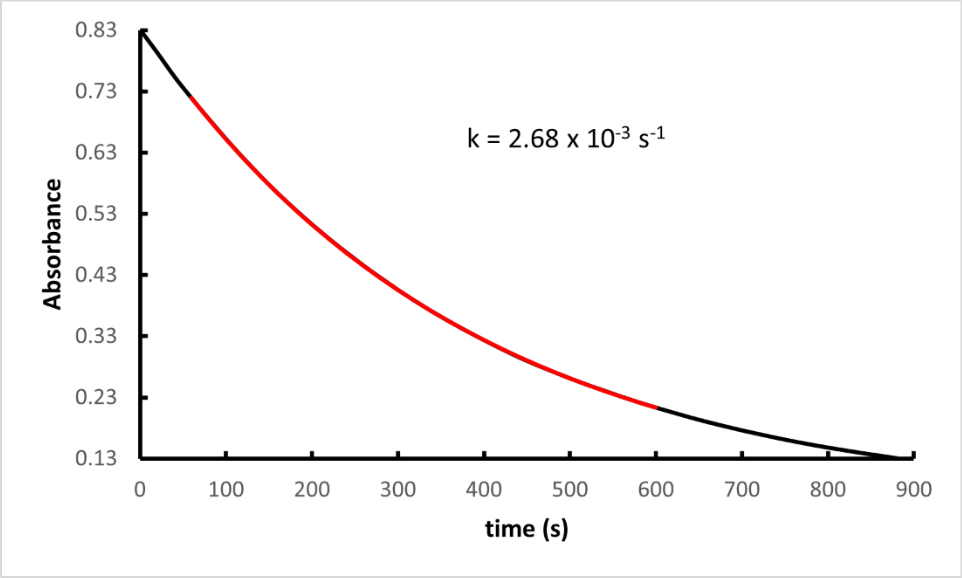

k = 2.68 x 10-3 s-1
Absorbance
time (s)
0.83
0.73
0.63
0.53
0.43
0.33
0.23
0.13
0
100
200
300
400
500
600
700
800
900

Results

Type	Rate (s^{-1})	Temperature	Type	Rate (s^{-1})	Temperature
C5	$3.18x10^{-3}$	20.2	cell	$2.66x10^{-3}$	20.3
C5	$2.85x10^{-3}$	20.3	cell	$2.65x10^{-3}$	20.4
C5	$2.72x10^{-3}$	20.3	cell	$2.68x10^{-3}$	20.3
C5	$2.91x10^{-3}$	20.3			
C5	$2.89x10^{-3}$	20.4			
Average:	**$2.91x10^{-3}$**	20.3	Average:	**$2.66x10^{-3}$**	20.3
Standard error:	**$7.51x10^{-5}$**		Standard error:	**$7.96x10^{-6}$**	

Reaction of *n*-butanol (N3) with electrophile E1:
Cavity measurements (C2)

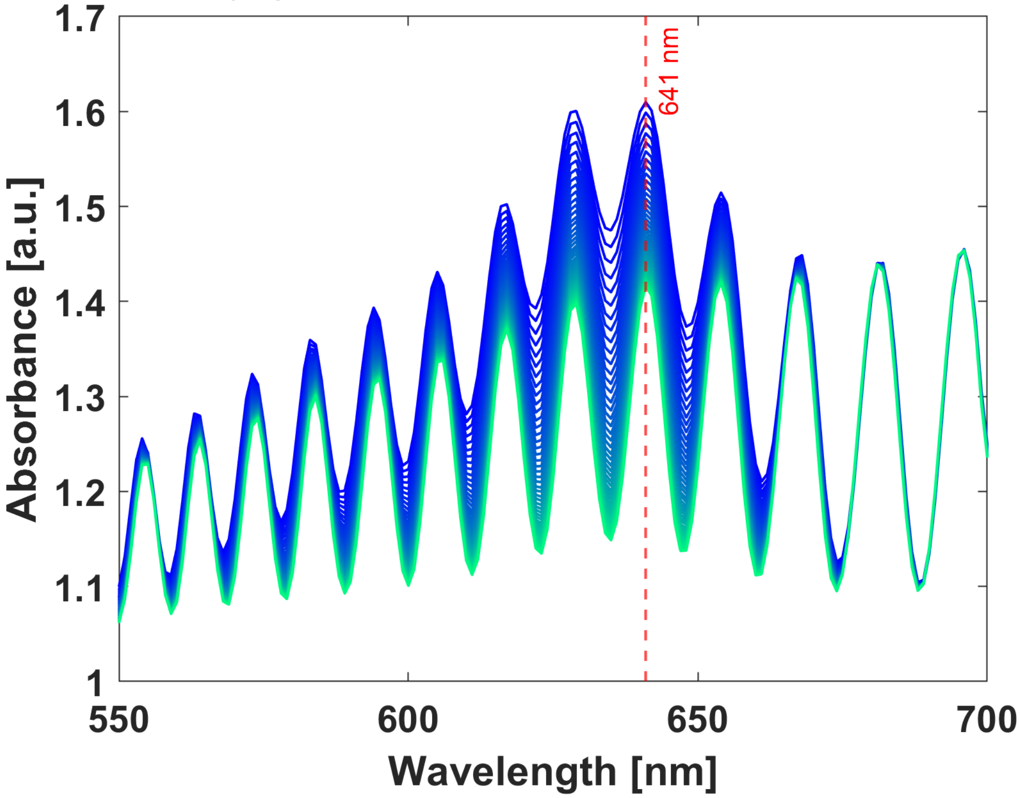

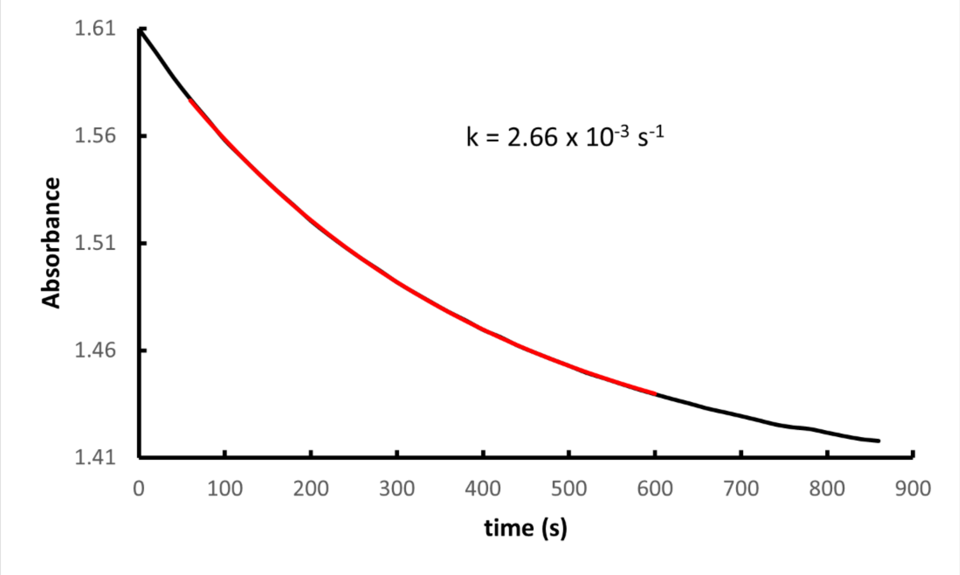

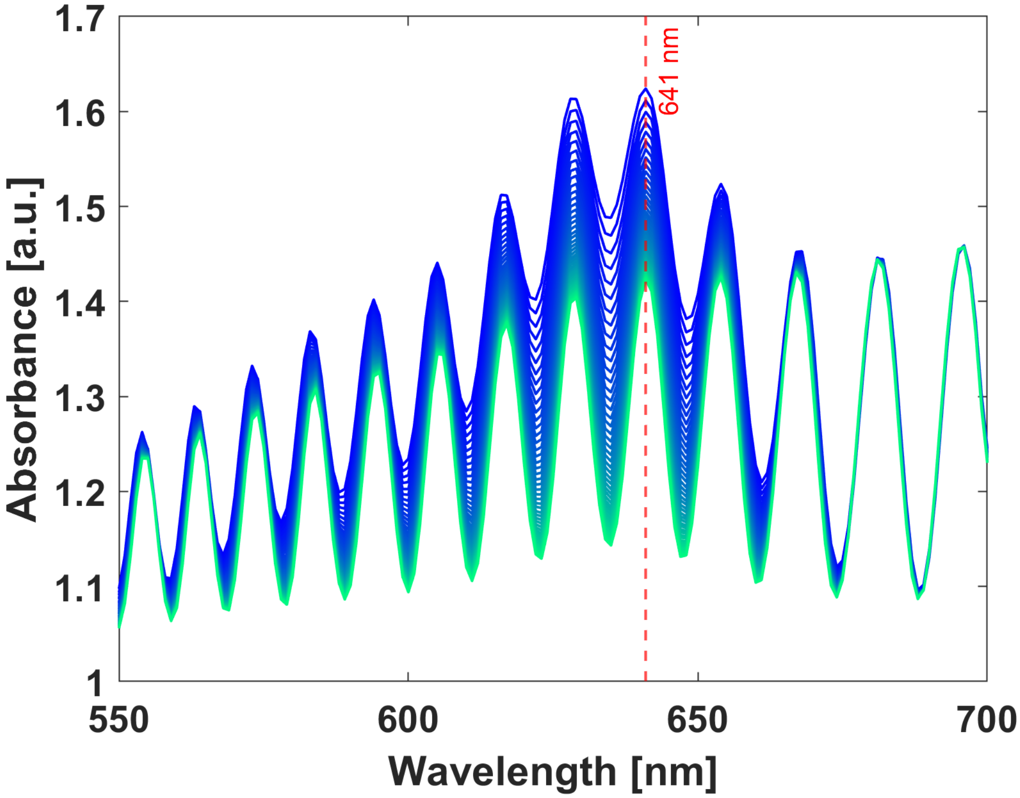
1.7
1.6
1.5
1.4
1.3
1.2
1.1
1
Absorbance [a.u.]
641 nm
550
600
650
700
Wavelength [nm]

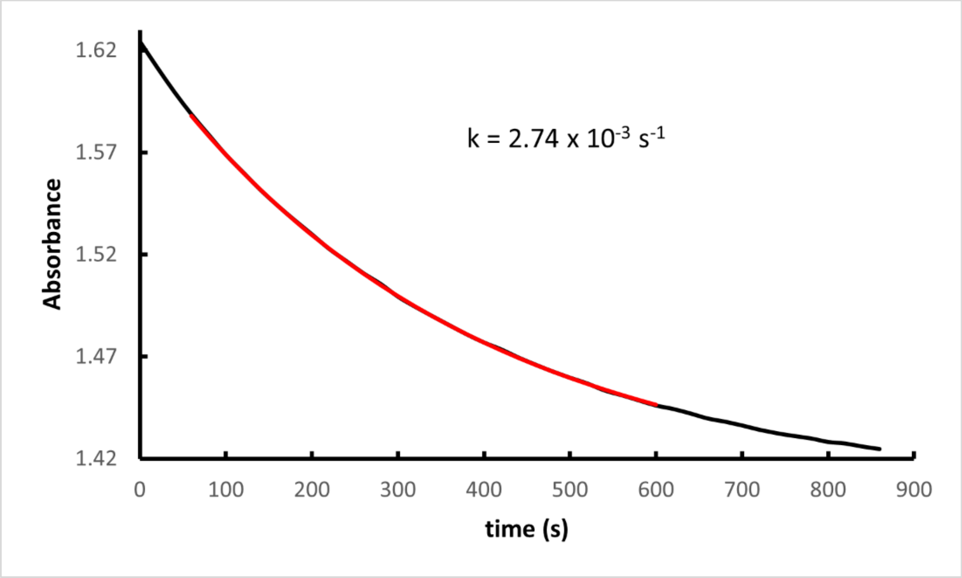
1.62
1.57
1.52
1.47
1.42
Absorbance
k = 2.74 x 10-3 s-1
0
100
200
300
400
500
600
700
800
900
time (s)

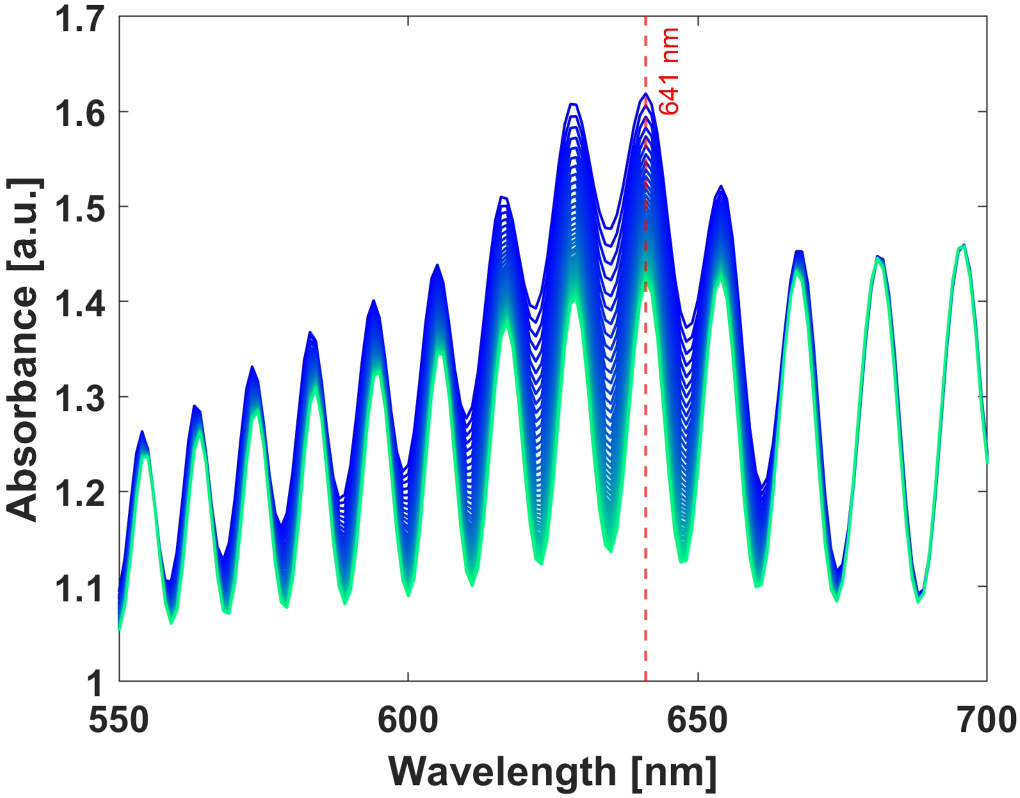

641 nm
Absorbance [a.u.]
Wavelength [nm]

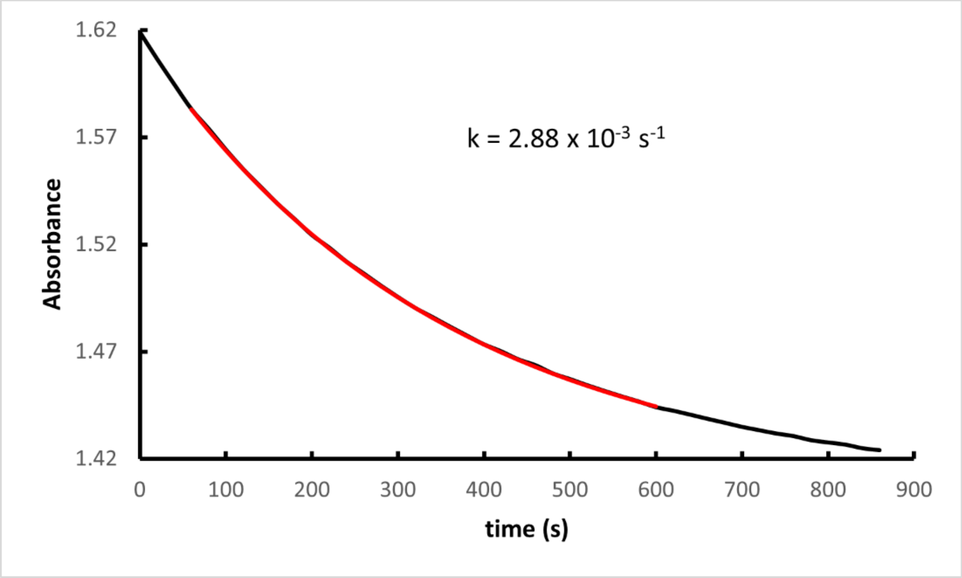

k = 2.88 x 10^-3 s^-1
Absorbance
time (s)

Cavity measurements (C3)

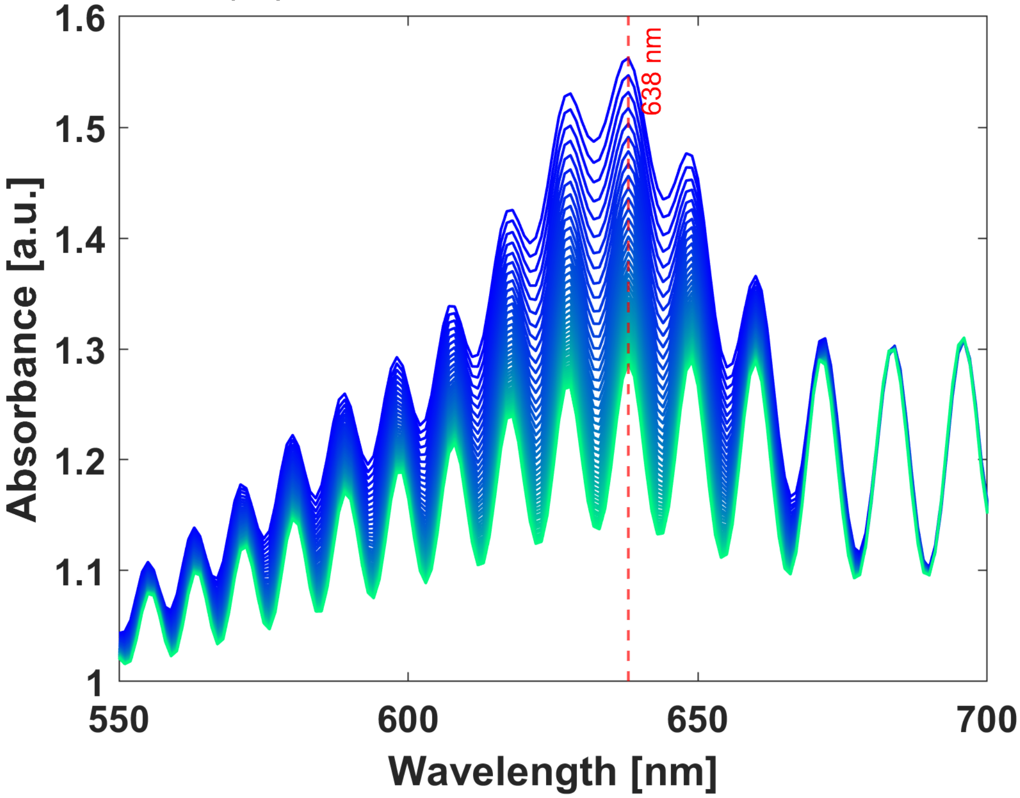

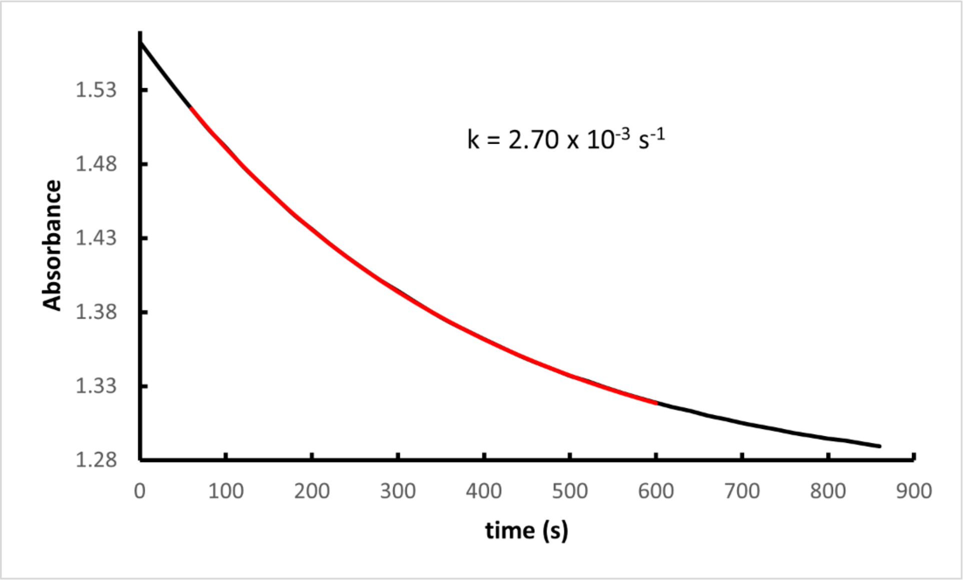

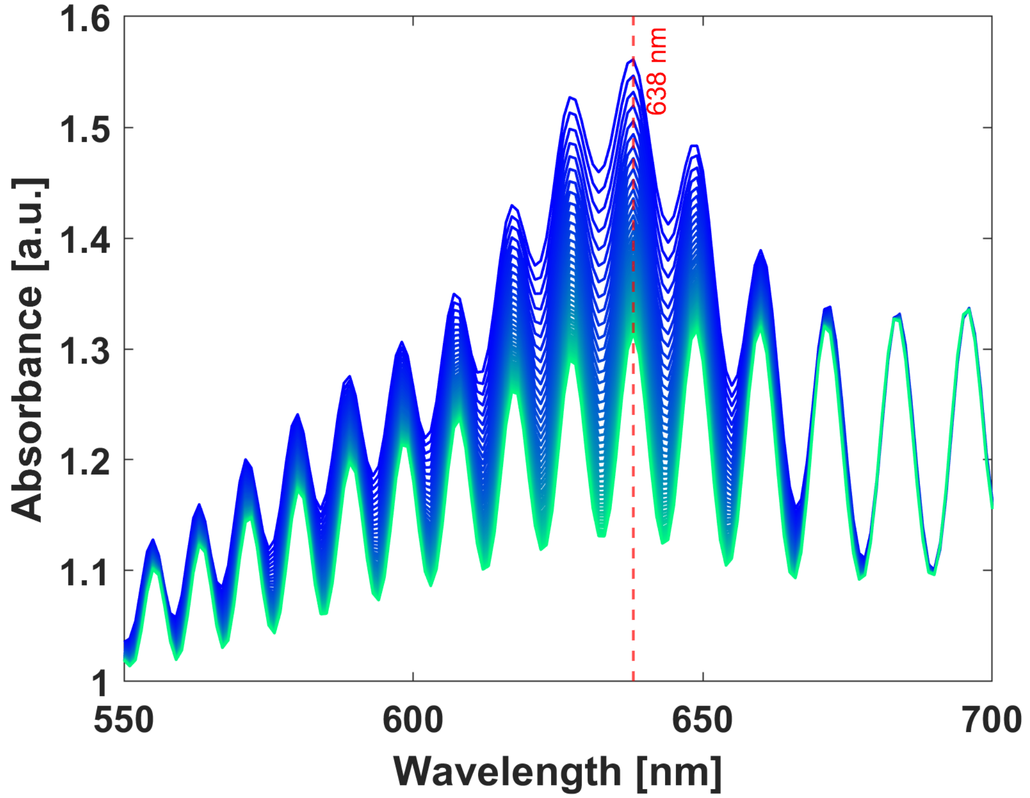

1.6
1.5
1.4
1.3
1.2
1.1
1
Absorbance [a.u.]
550
600
650
700
Wavelength [nm]
638 nm

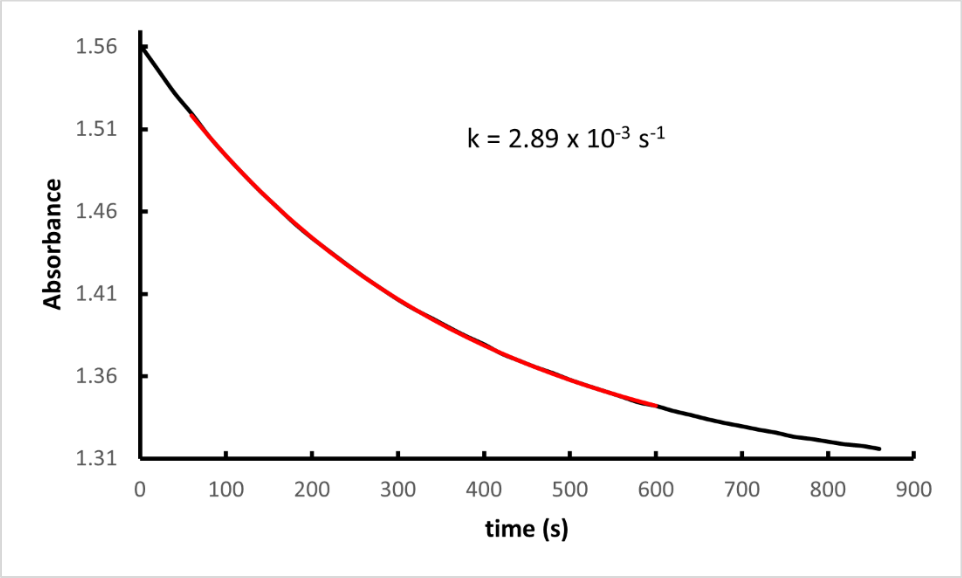

1.56
1.51
1.46
1.41
1.36
1.31
Absorbance
0
100
200
300
400
500
600
700
800
900
time (s)
k = 2.89 x 10-3 s-1

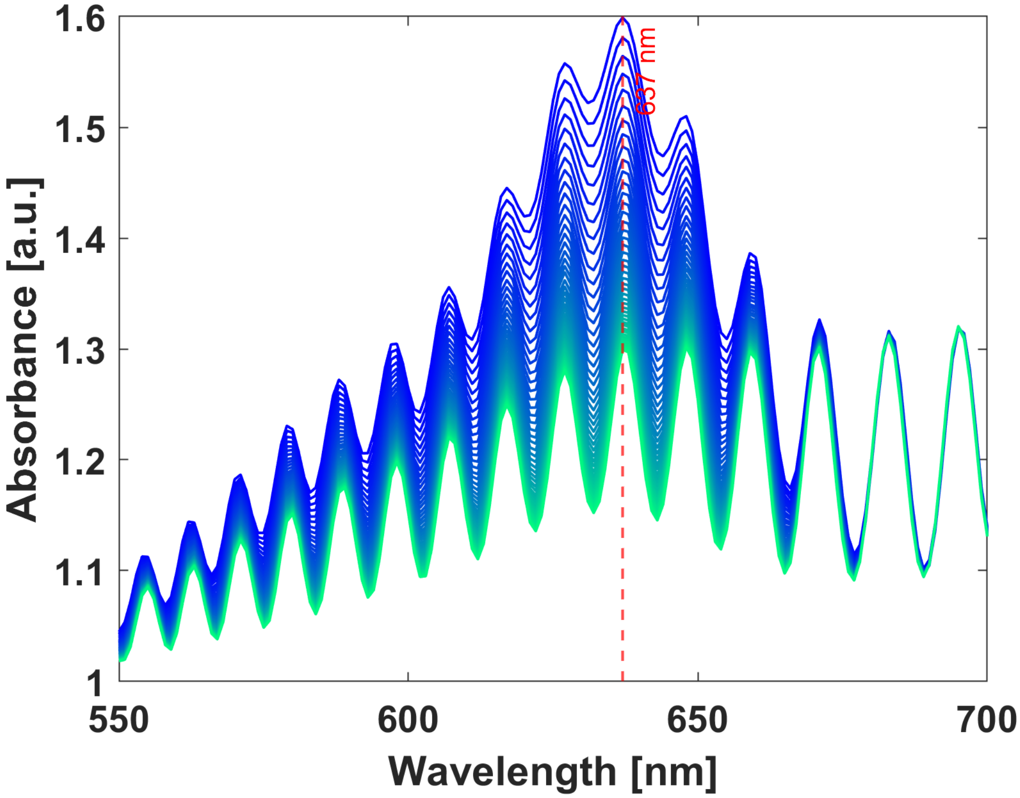

637 nm
1.6
1.5
1.4
1.3
1.2
1.1
1
Absorbance [a.u.]
550
600
650
700
Wavelength [nm]

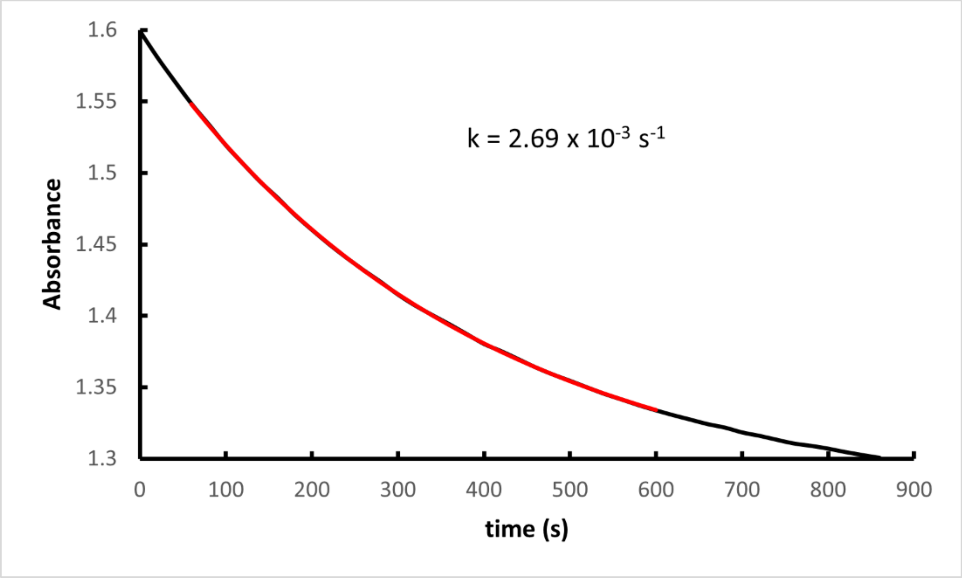

k = 2.69 x 10-3 s-1
1.6
1.55
1.5
1.45
1.4
1.35
1.3
Absorbance
0
100
200
300
400
500
600
700
800
900
time (s)

Cavity measurements (C4)

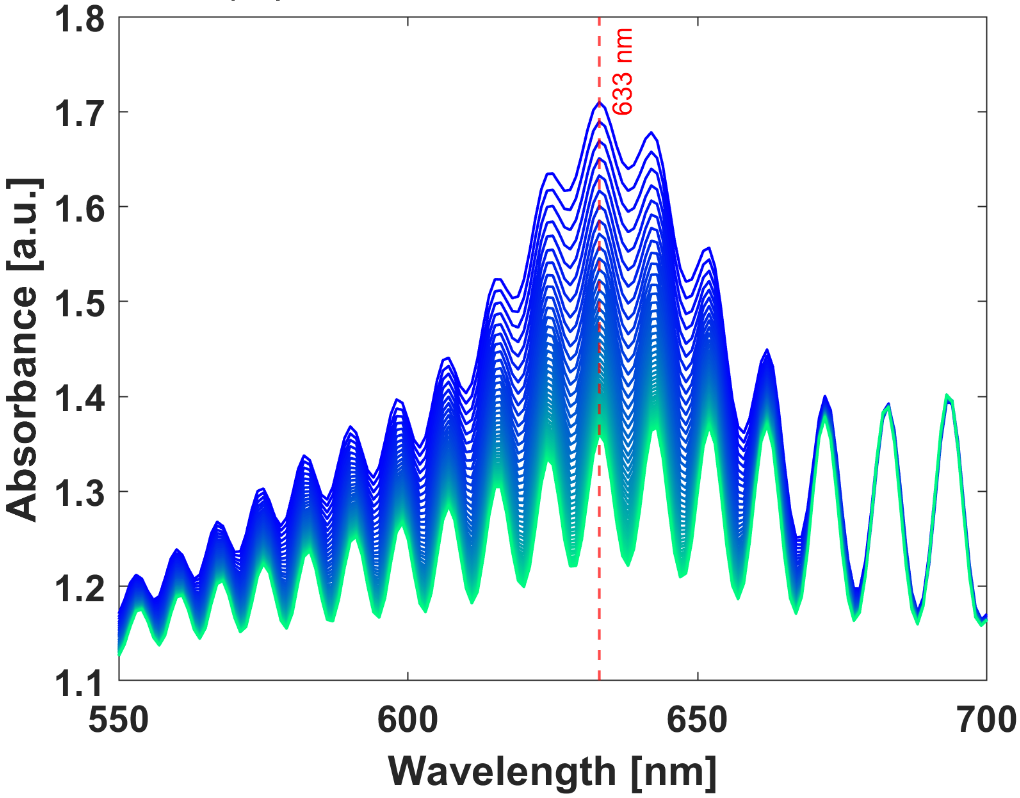

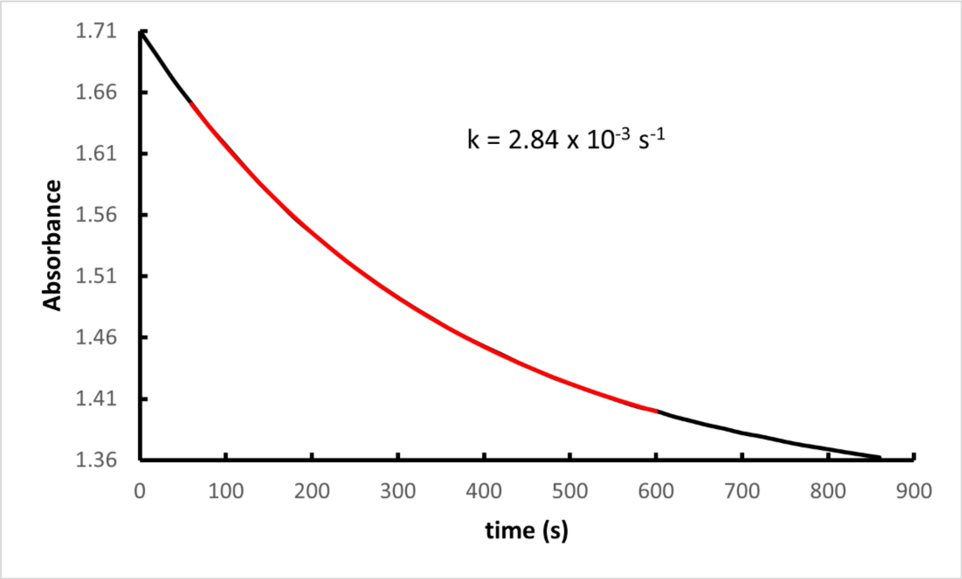

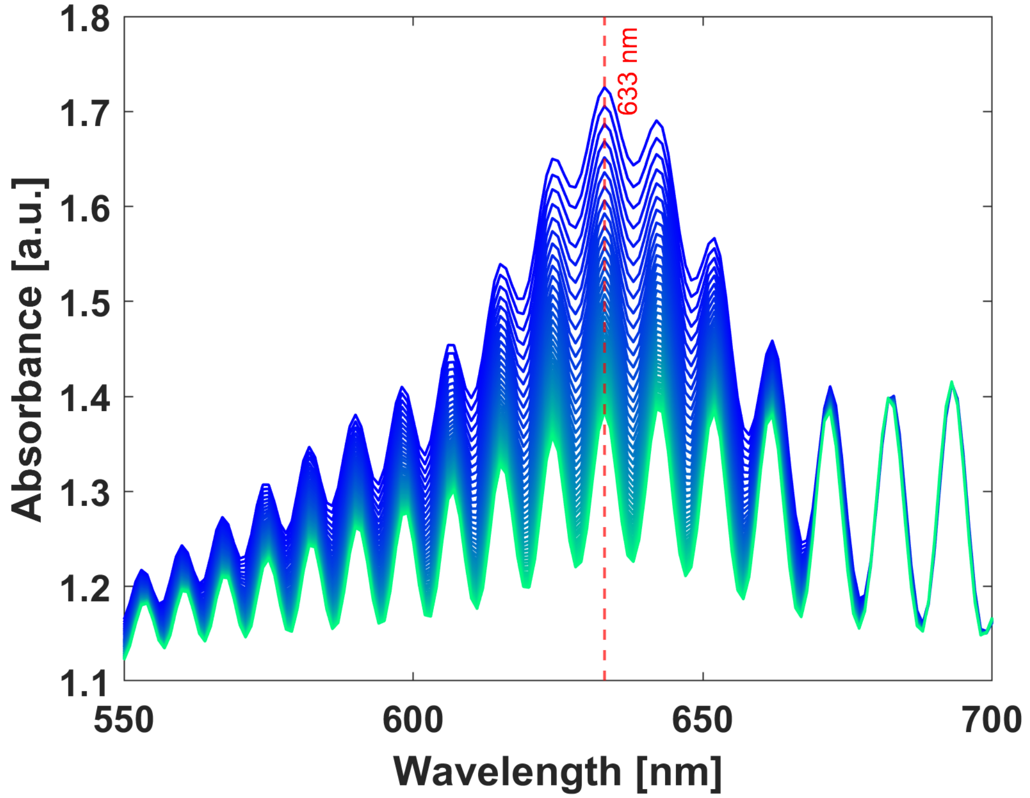

1.8
1.7
1.6
1.5
1.4
1.3
1.2
1.1
Absorbance [a.u.]
633 nm
550
600
650
700
Wavelength [nm]

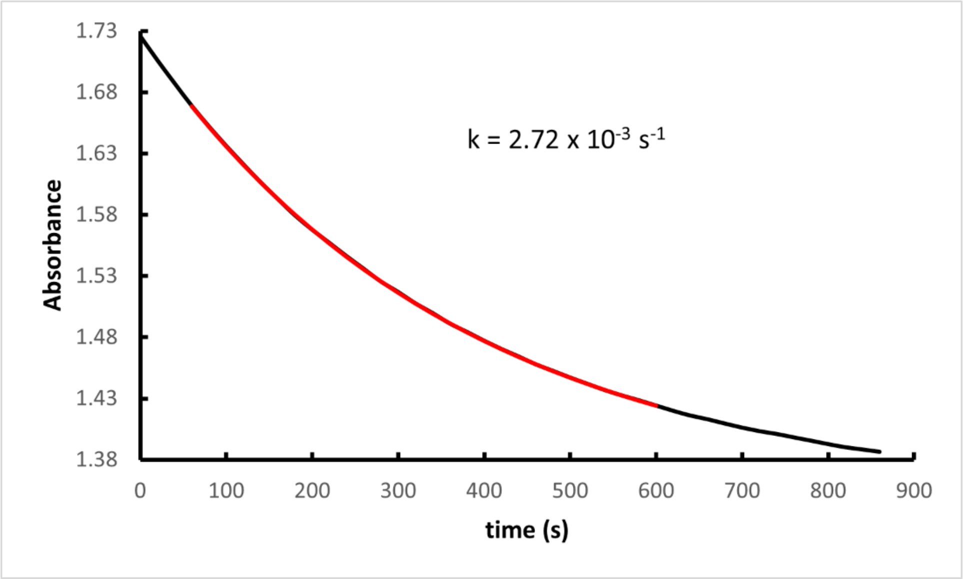

1.73
1.68
1.63
1.58
1.53
1.48
1.43
1.38
Absorbance
k = 2.72 x 10-3 s-1
0
100
200
300
400
500
600
700
800
900
time (s)

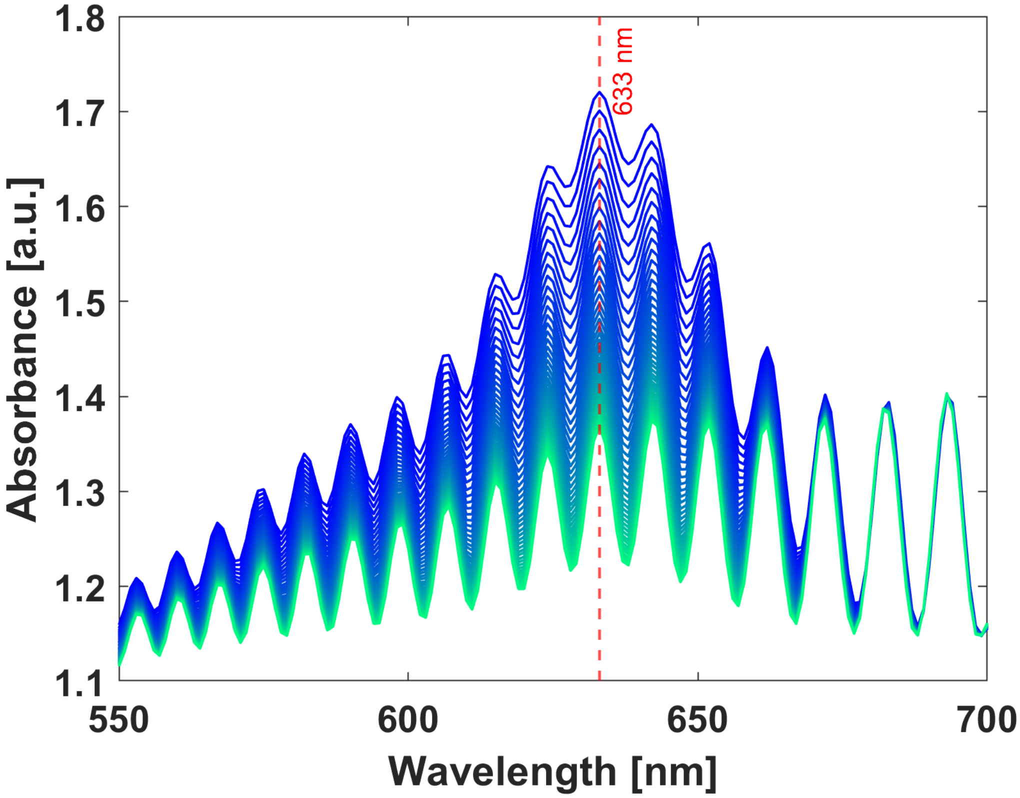

633 nm
Absorbance [a.u.]
Wavelength [nm]
1.8
1.7
1.6
1.5
1.4
1.3
1.2
1.1
550
600
650
700

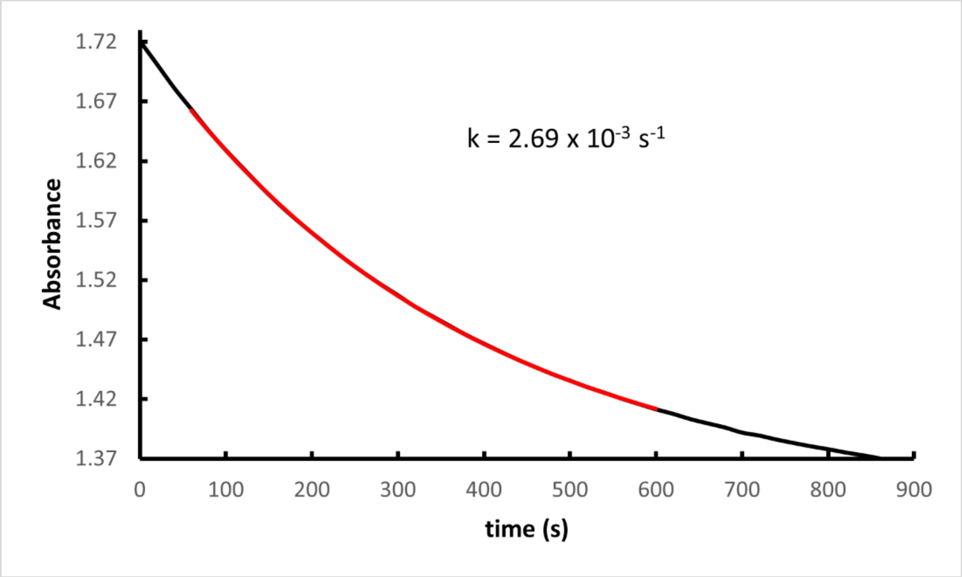

k = 2.69 x 10-3 s-1
Absorbance
time (s)
1.72
1.67
1.62
1.57
1.52
1.47
1.42
1.37
0
100
200
300
400
500
600
700
800
900

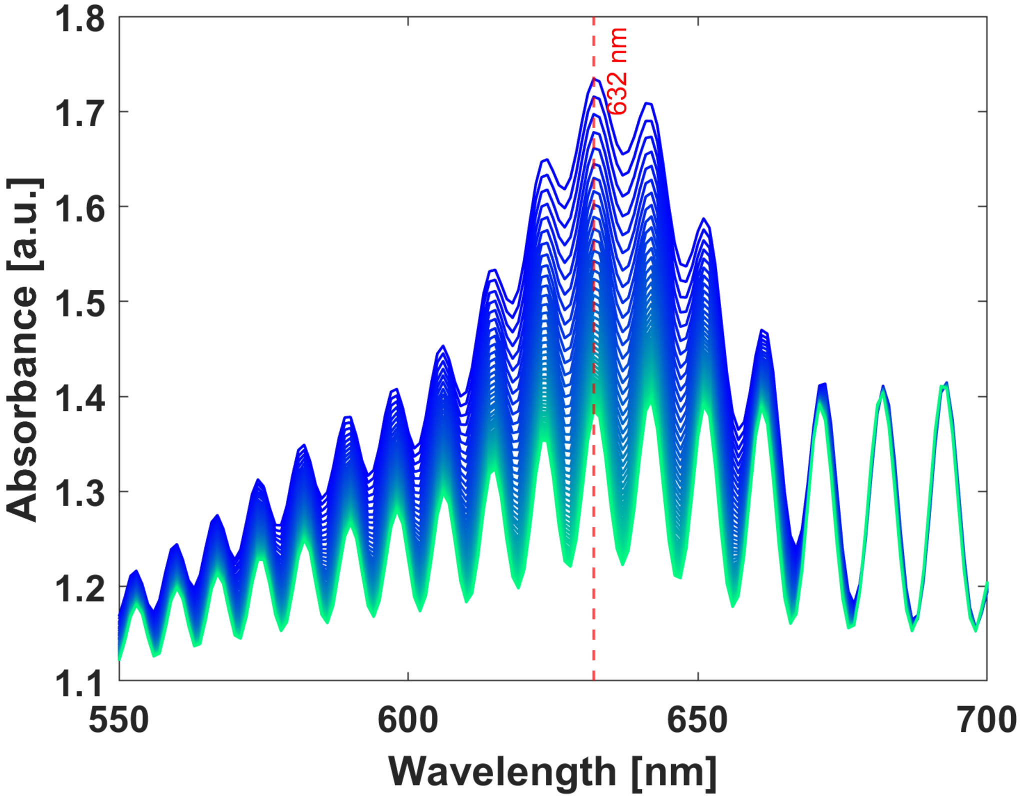

632 nm
Absorbance [a.u.]
Wavelength [nm]
1.8
1.7
1.6
1.5
1.4
1.3
1.2
1.1
550
600
650
700

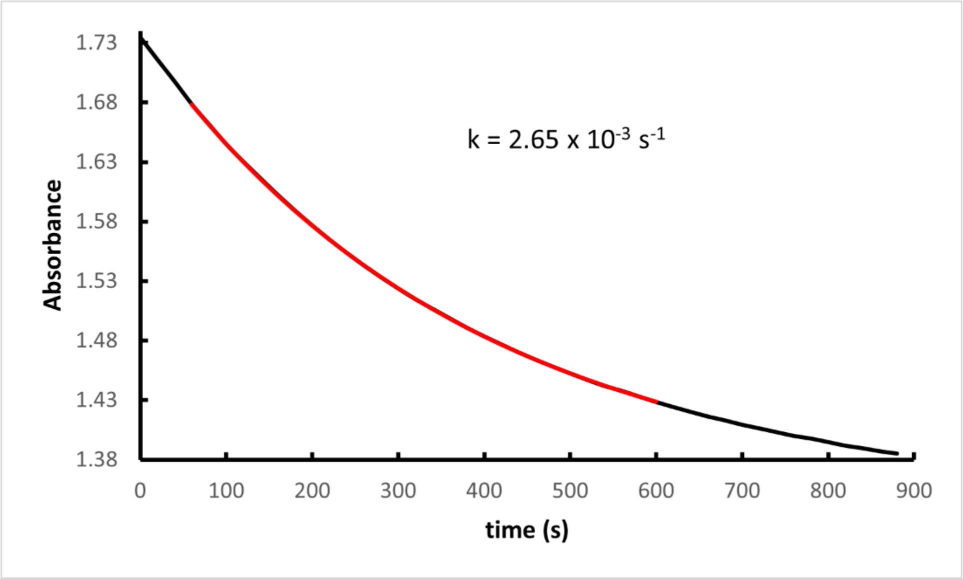

k = 2.65 x 10-3 s-1
Absorbance
time (s)
1.73
1.68
1.63
1.58
1.53
1.48
1.43
1.38
0
100
200
300
400
500
600
700
800
900

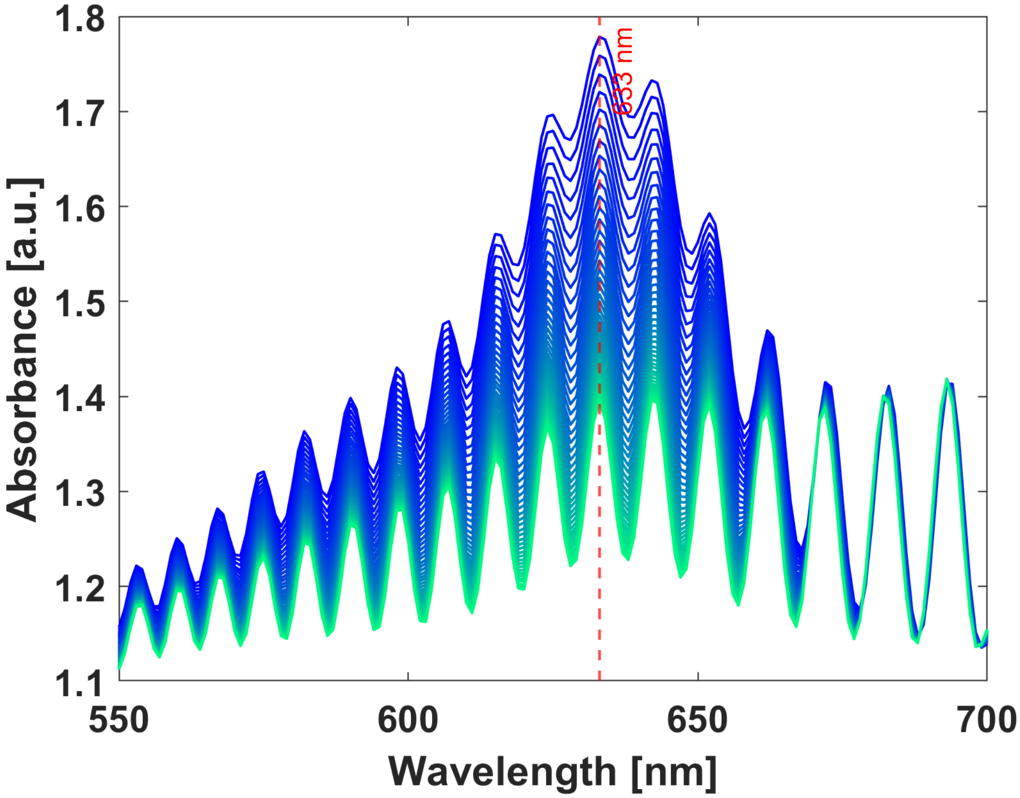

1.8
1.7
1.6
1.5
1.4
1.3
1.2
1.1
Absorbance [a.u.]
633 nm
550
600
650
700
Wavelength [nm]

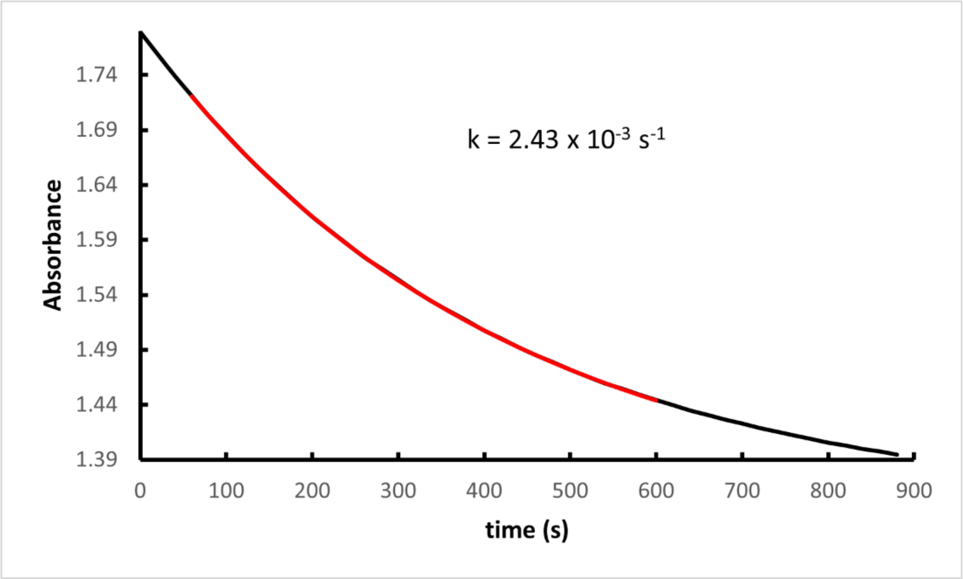

1.74
1.69
1.64
1.59
1.54
1.49
1.44
1.39
Absorbance
k = 2.43 x 10-3 s-1
0
100
200
300
400
500
600
700
800
900
time (s)

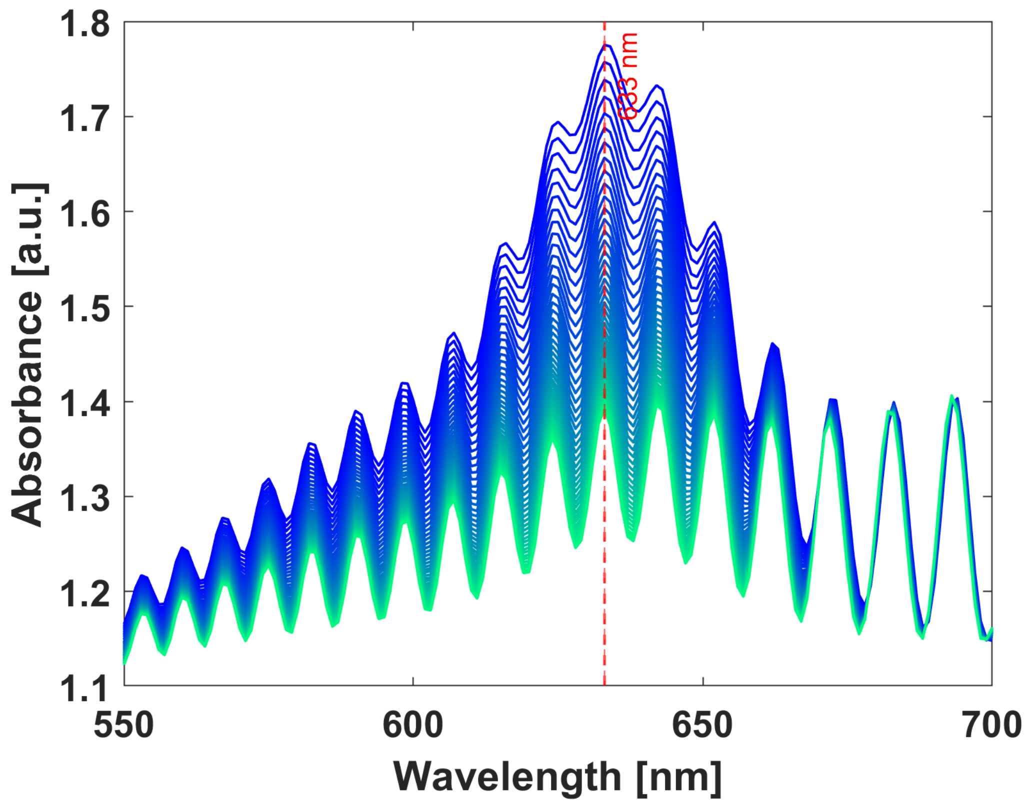

633 nm
Absorbance [a.u.]
Wavelength [nm]
1.8
1.7
1.6
1.5
1.4
1.3
1.2
1.1
550
600
650
700

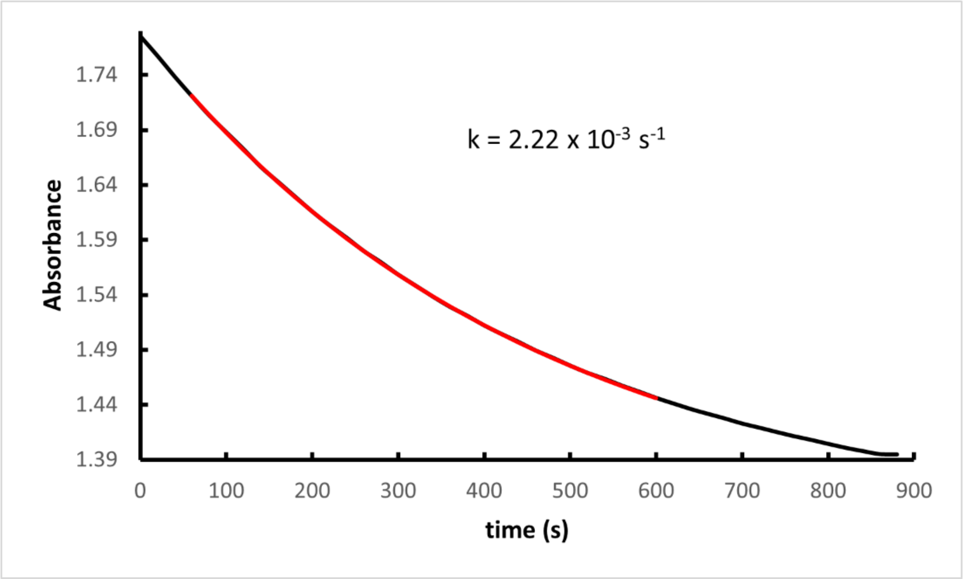

k = 2.22 x 10^{-3} s^{-1}
Absorbance
time (s)
1.74
1.69
1.64
1.59
1.54
1.49
1.44
1.39
0
100
200
300
400
500
600
700
800
900

Cavity measurements (C5)

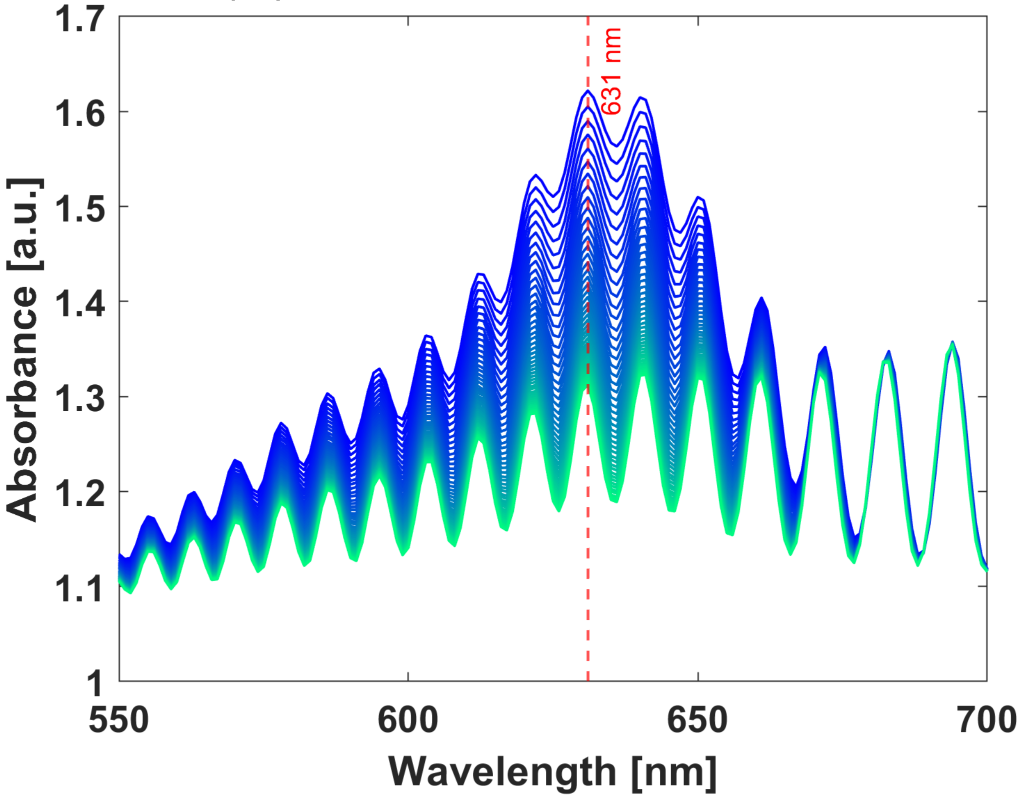

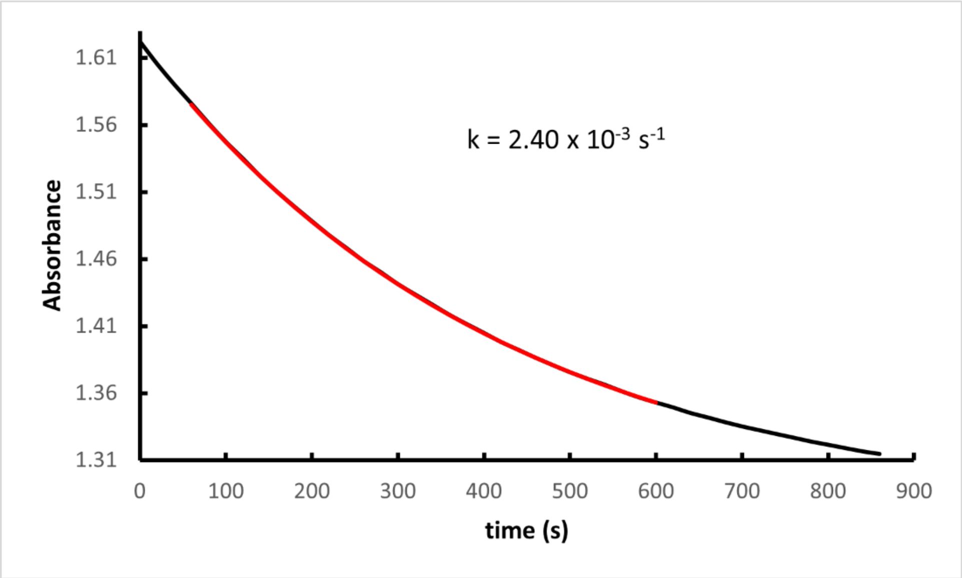

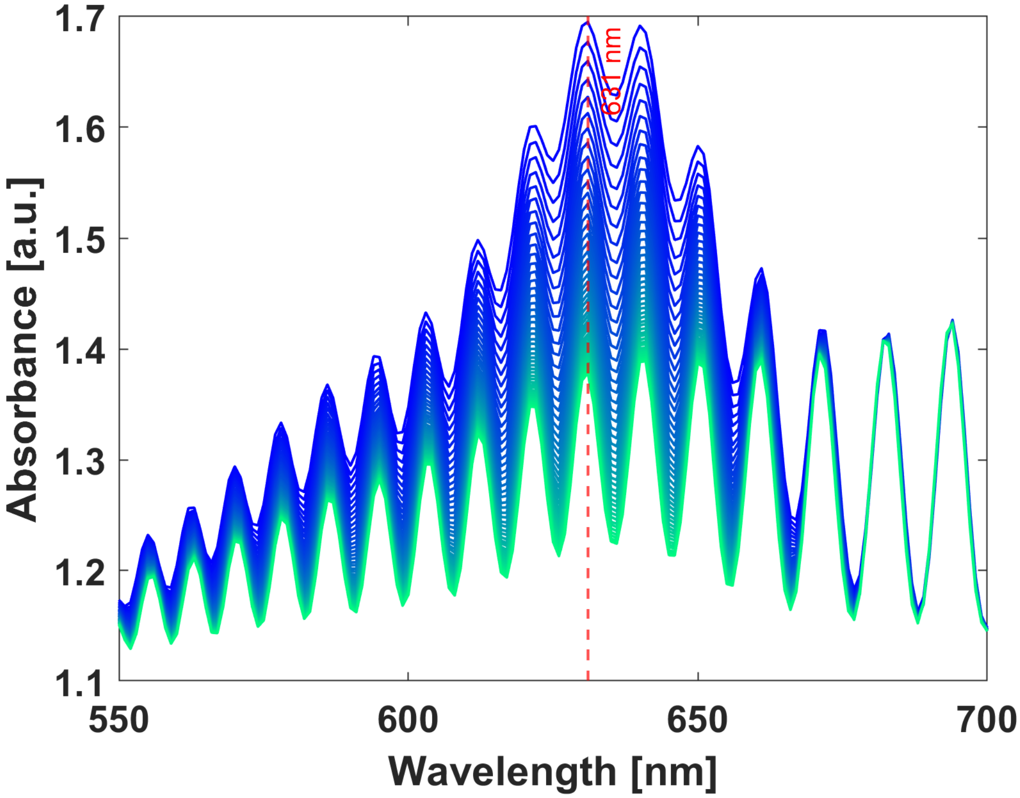

1.7
1.6
1.5
1.4
1.3
1.2
1.1
Absorbance [a.u.]
631 nm
550
600
650
700
Wavelength [nm]

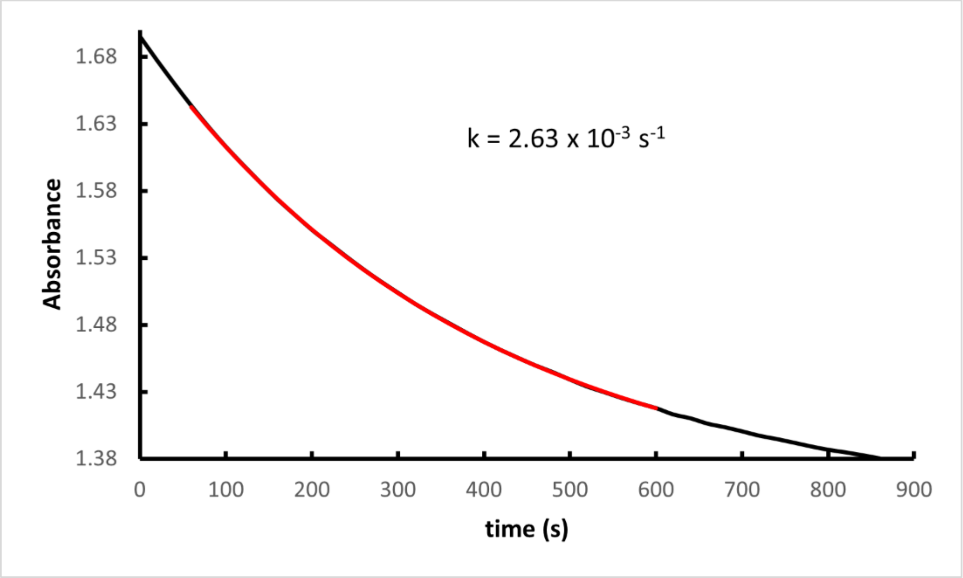

1.68
1.63
1.58
1.53
1.48
1.43
1.38
Absorbance
k = 2.63 x 10-3 s-1
0
100
200
300
400
500
600
700
800
900
time (s)

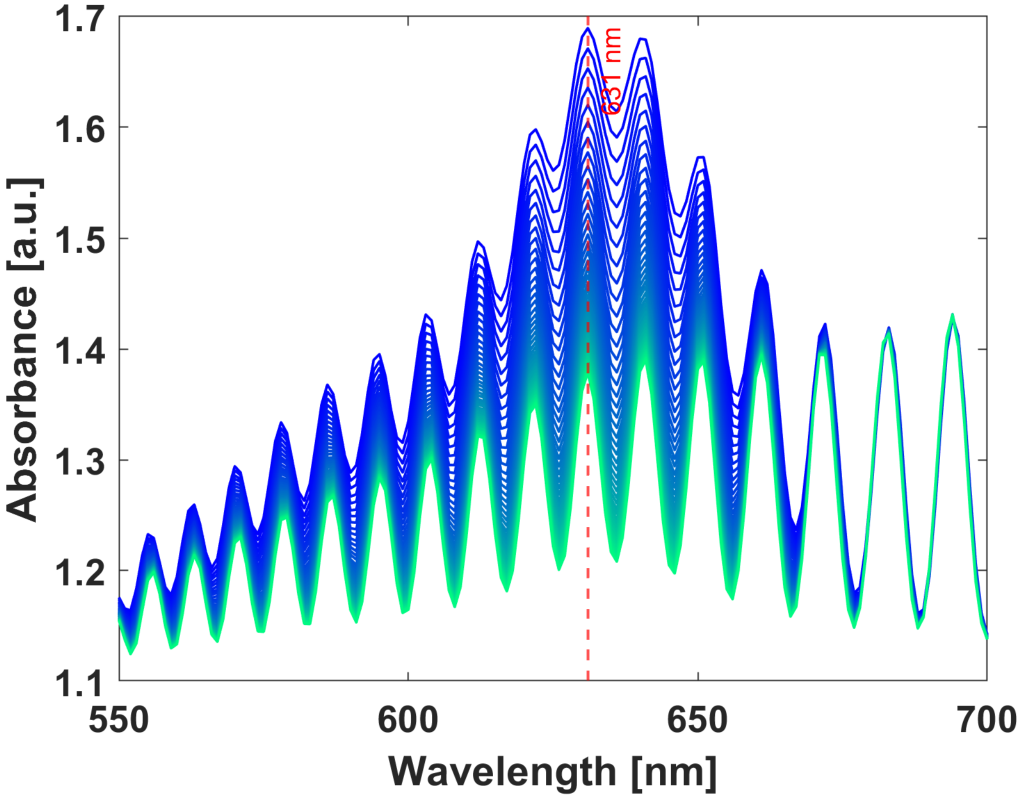

1.7
1.6
1.5
1.4
1.3
1.2
1.1
Absorbance [a.u.]
631 nm
550
600
650
700
Wavelength [nm]

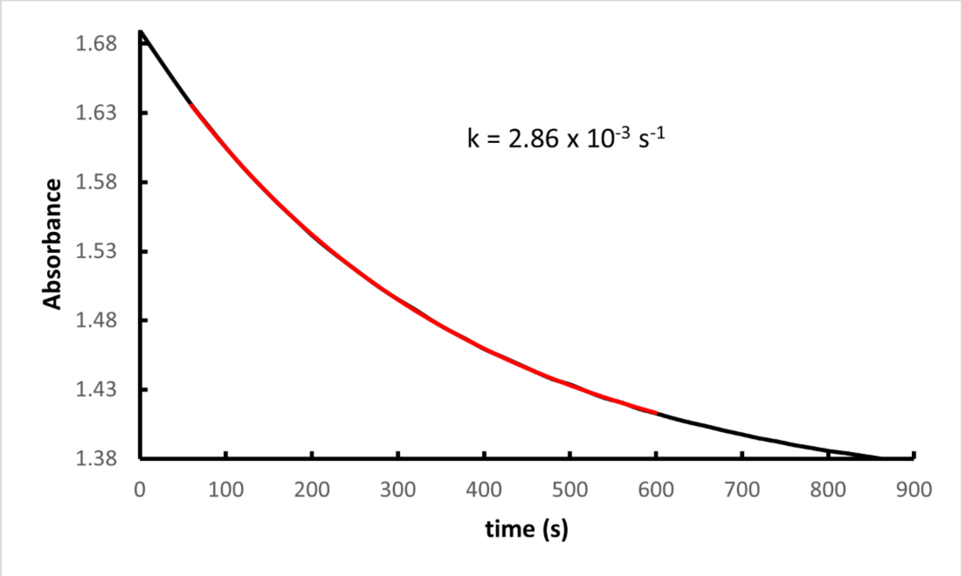

1.68
1.63
1.58
1.53
1.48
1.43
1.38
Absorbance
k = 2.86 x 10-3 s-1
0
100
200
300
400
500
600
700
800
900
time (s)

Cell measurements (standard)

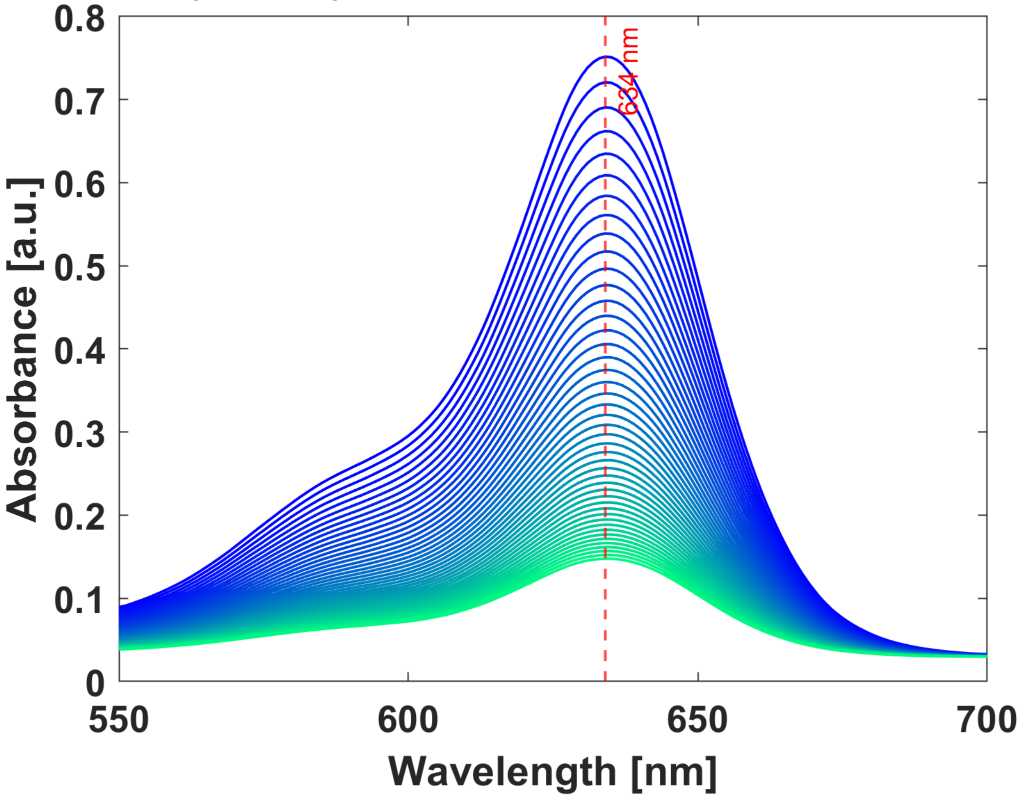

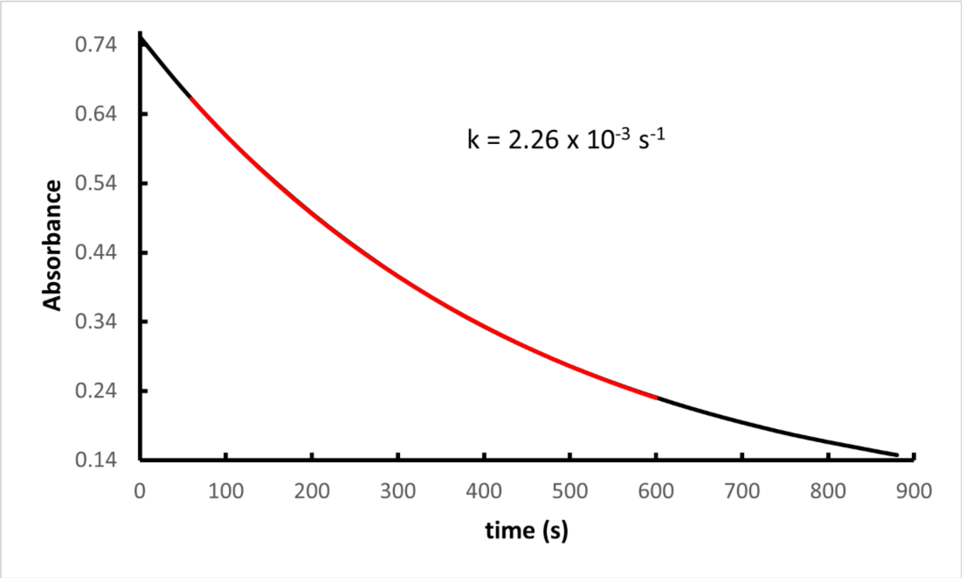

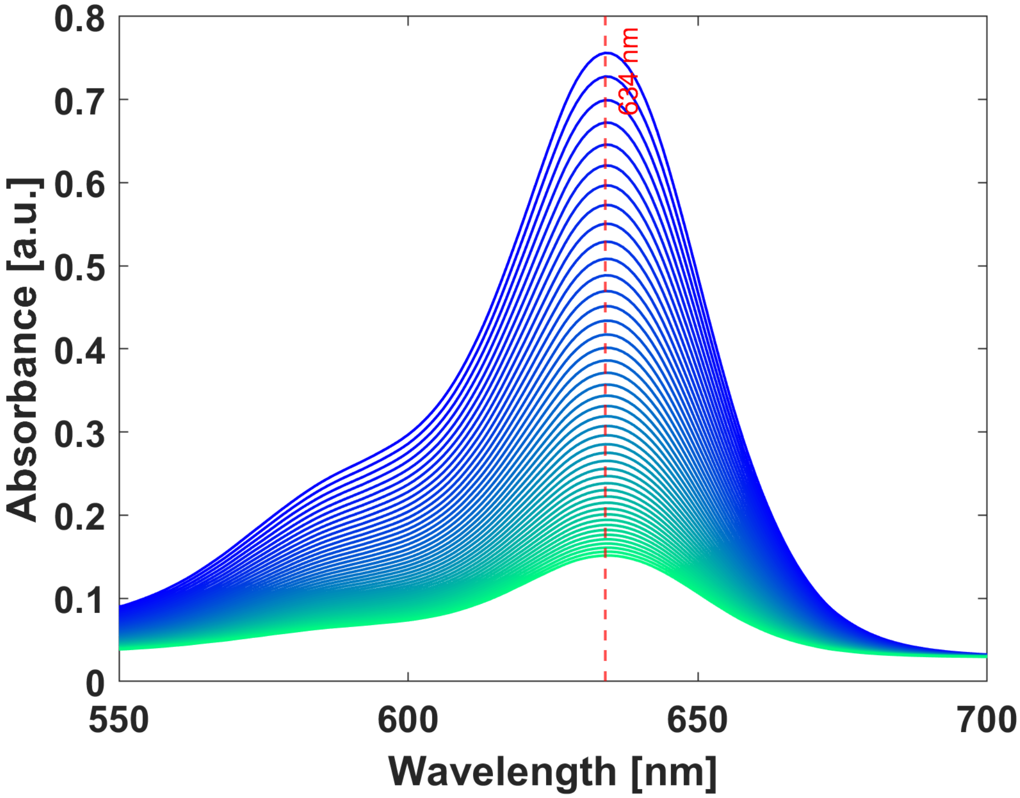

634 nm
Absorbance [a.u.]
Wavelength [nm]
0.8
0.7
0.6
0.5
0.4
0.3
0.2
0.1
0
550
600
650
700

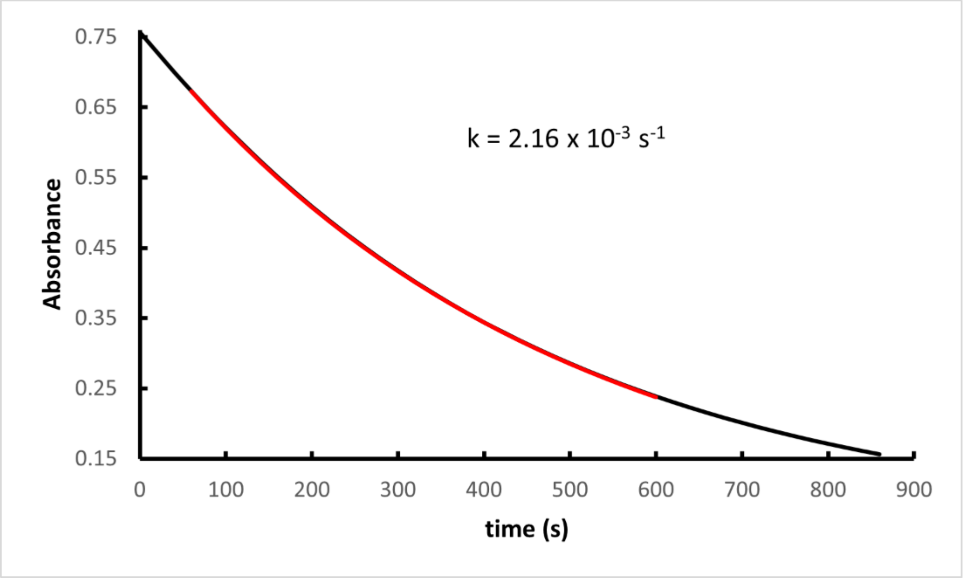

k = 2.16 x 10-3 s-1
Absorbance
time (s)
0.75
0.65
0.55
0.45
0.35
0.25
0.15
0
100
200
300
400
500
600
700
800
900

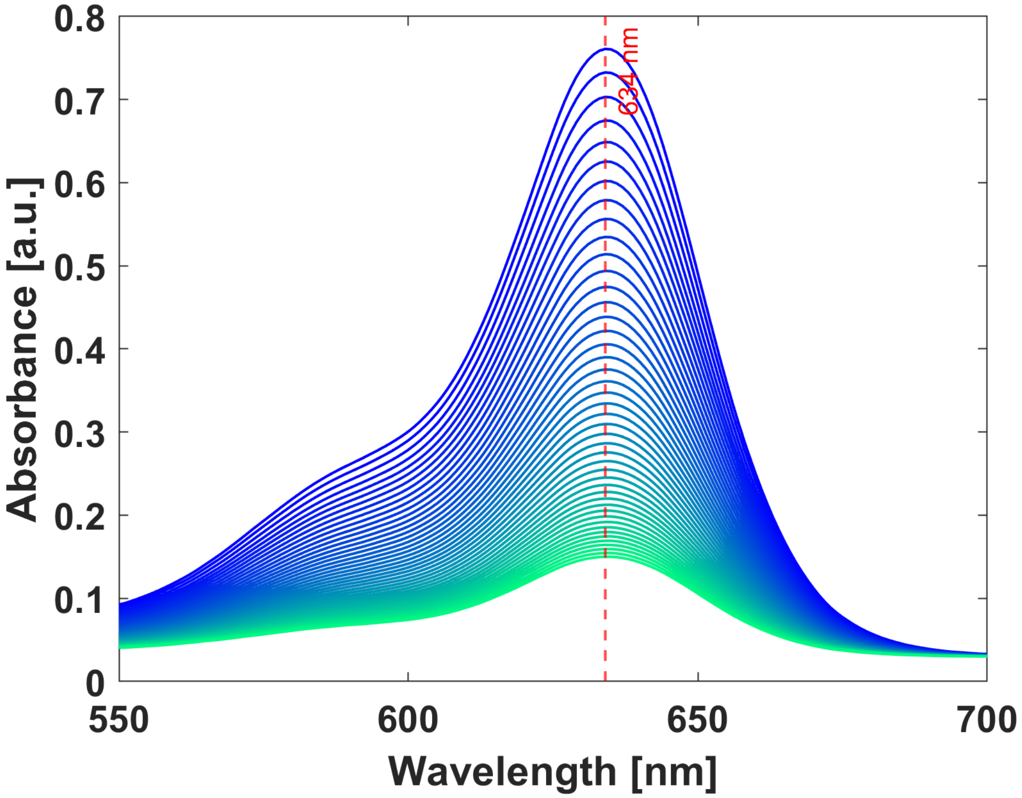
0.8
0.7
0.6
0.5
0.4
0.3
0.2
0.1
0
550
600
650
700
Absorbance [a.u.]
Wavelength [nm]
634 nm

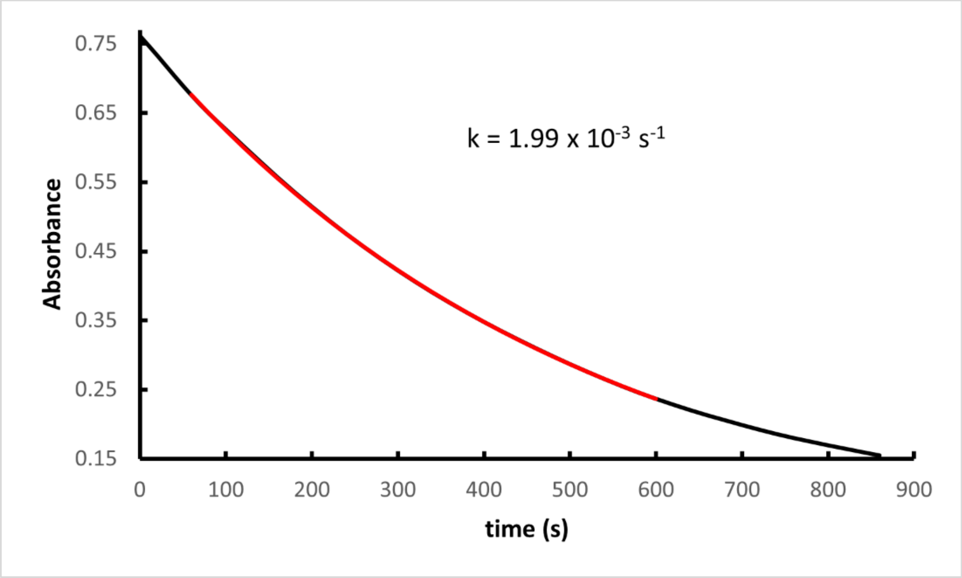
0.75
0.65
0.55
0.45
0.35
0.25
0.15
0
100
200
300
400
500
600
700
800
900
Absorbance
time (s)
k = 1.99 x 10-3 s-1

Results

type	Rate (s^{-1})	Temperature	type	Rate (s^{-1})	Temperature
C2	$2.66x10^{-3}$	20	C3	$2.70x10^{-3}$	20.3
C2	$2.74x10^{-3}$	20.1	C3	$2.89x10^{-3}$	20.3
C2	$2.88x10^{-3}$	20.1	C3	$2.69x10^{-3}$	20.2
Average:	**$2.76x10^{-3}$**	20.1	Average:	**$2.76x10^{-3}$**	20.3
Standard error:	**$6.24x10^{-5}$**		Standard error:	**$6.38x10^{-5}$**	

type	Rate (s^{-1})	Temperature	type	Rate (s^{-1})	Temperature
C4	$2.84x10^{-3}$	20.3	C5	$2.40x10^{-3}$	20
C4	$2.72x10^{-3}$	20.2	C5	$2.63x10^{-3}$	20.1
C4	$2.69x10^{-3}$	20.2	C5	$2.86x10^{-3}$	20.1
C4	$2.65x10^{-3}$	19.7	Average:	**$2.63x10^{-3}$**	20.1
C4	$2.43x10^{-3}$	19.6	Standard error:	**$1.35x10^{-4}$**	
C4	$2.22x10^{-3}$	19.6			
Average:	**$2.59x10^{-3}$**	19.9			
Standard error:	**$9.24x10^{-5}$**				

type	Rate (s^{-1})	Temperature
cell	$2.26x10^{-3}$	19.9
cell	$2.16x10^{-3}$	19.9
cell	$1.99x10^{-3}$	19.9
Average:	**$2.14x10^{-3}$**	19.9
Standard error:	**$7.67x10^{-5}$**	

Reaction of *n*-butanol (N3) with electrophile E1 (90/10 v/v):
Cavity measurements (C2)

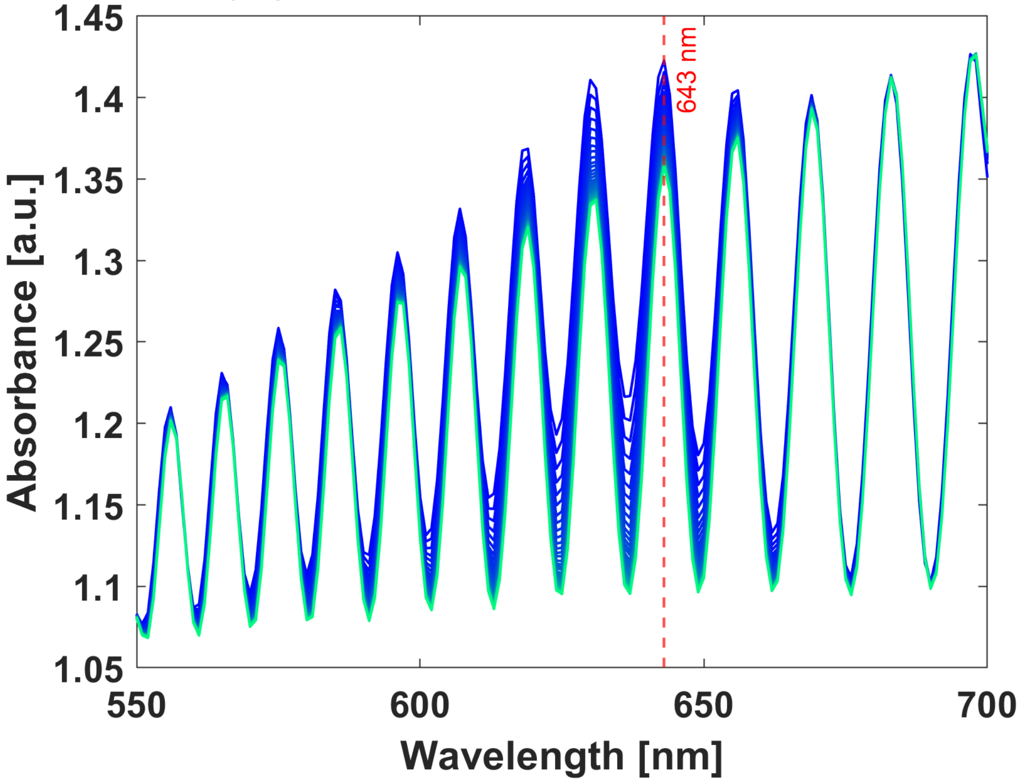

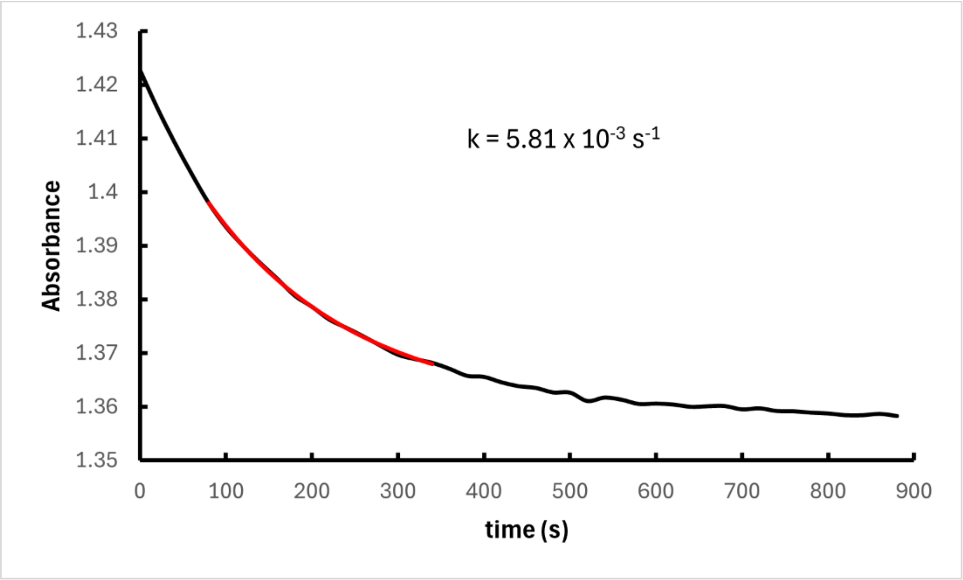

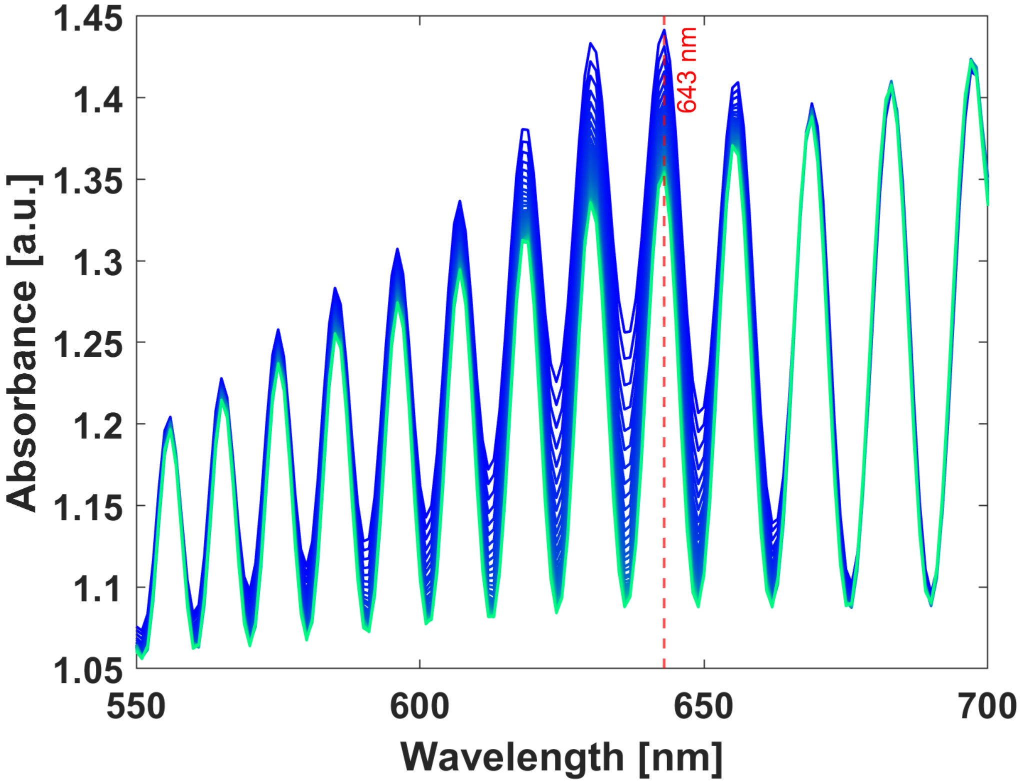

Absorbance [a.u.]
Wavelength [nm]
643 nm
1.45
1.4
1.35
1.3
1.25
1.2
1.15
1.1
1.05
550
600
650
700

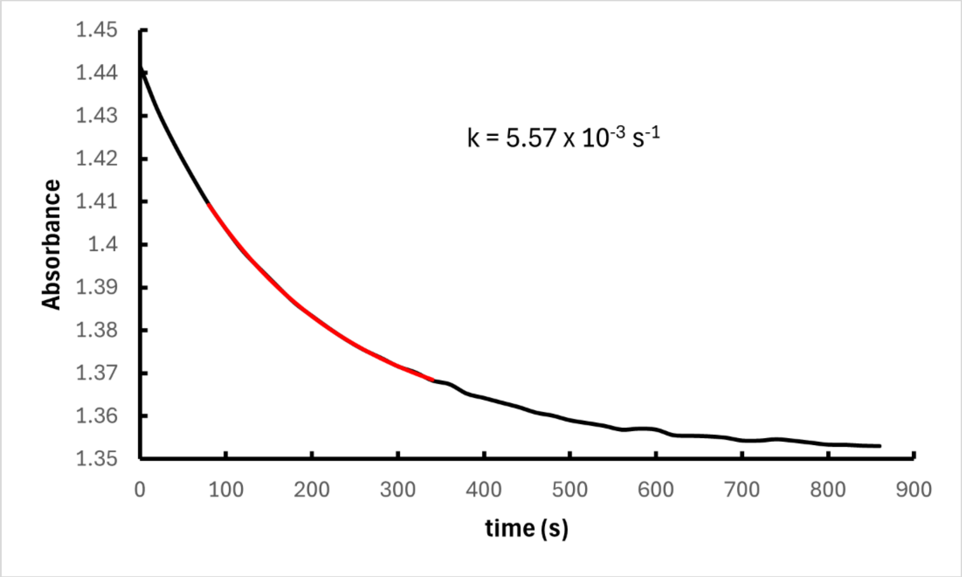

k = 5.57 x 10-3 s-1
Absorbance
time (s)
1.45
1.44
1.43
1.42
1.41
1.4
1.39
1.38
1.37
1.36
1.35
0
100
200
300
400
500
600
700
800
900

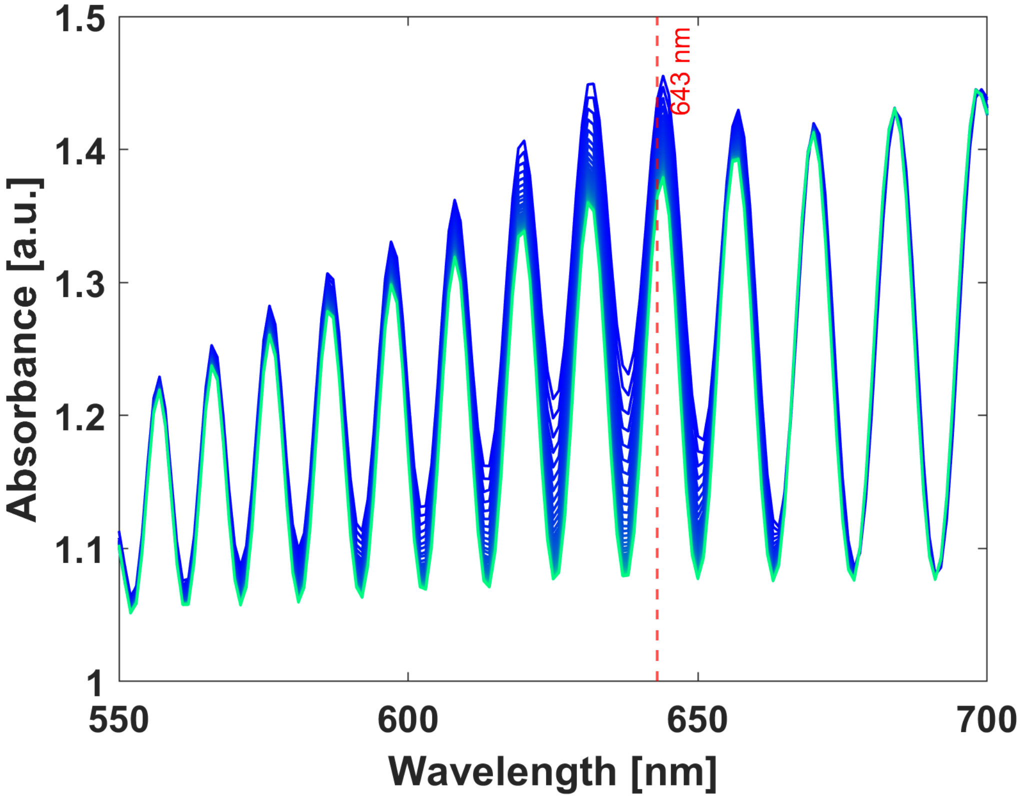

1.5
1.4
1.3
1.2
1.1
1
550
600
650
700
Wavelength [nm]
Absorbance [a.u.]
643 nm

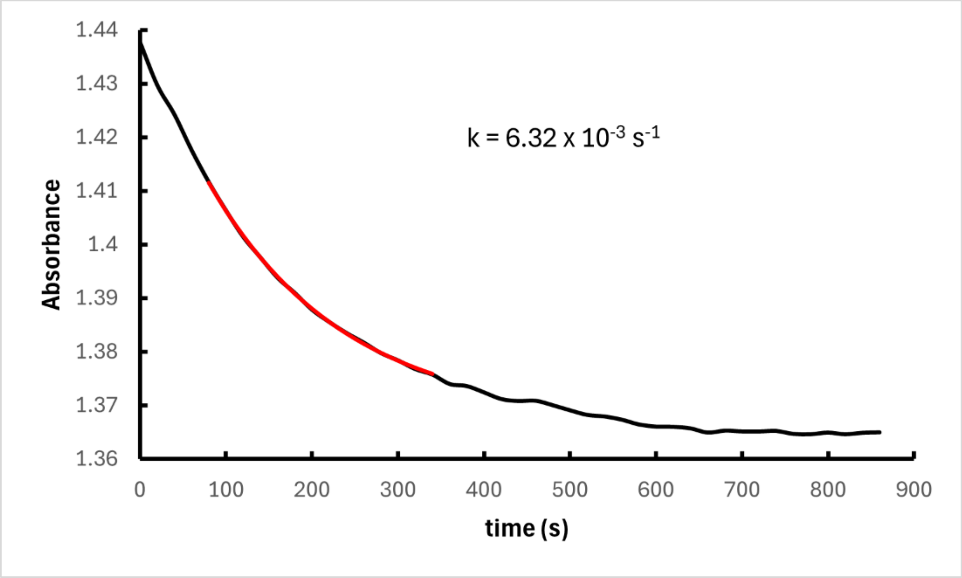

1.44
1.43
1.42
1.41
1.4
1.39
1.38
1.37
1.36
0
100
200
300
400
500
600
700
800
900
time (s)
Absorbance
k = 6.32 x 10-3 s-1

Cell measurements (standard)

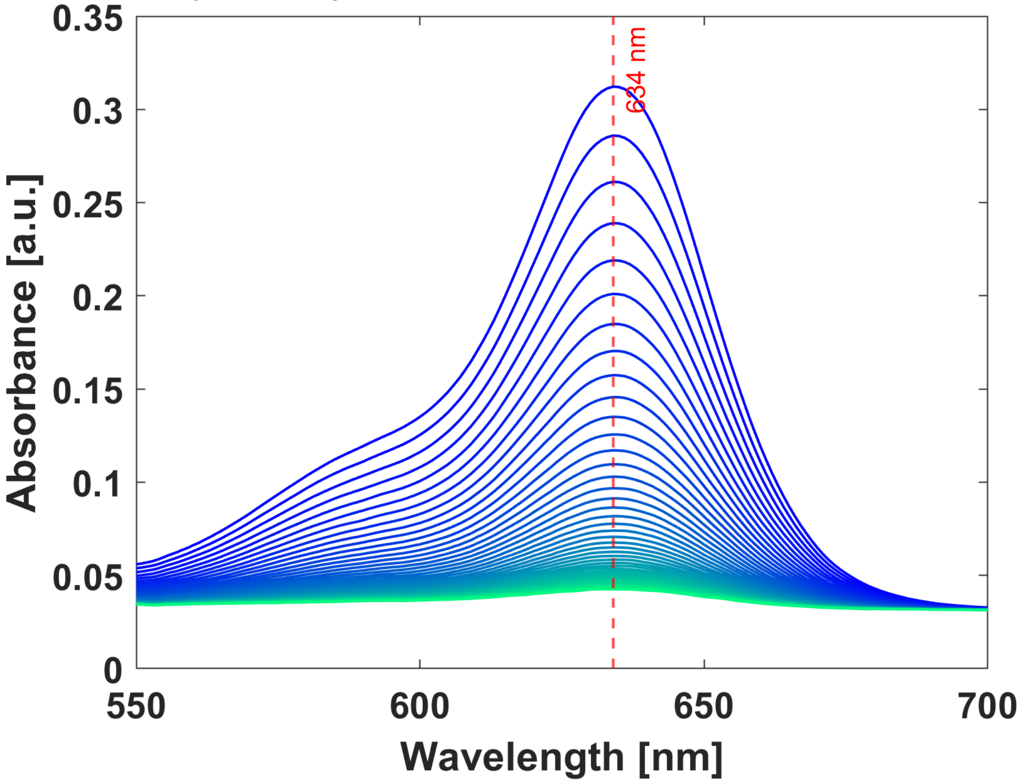

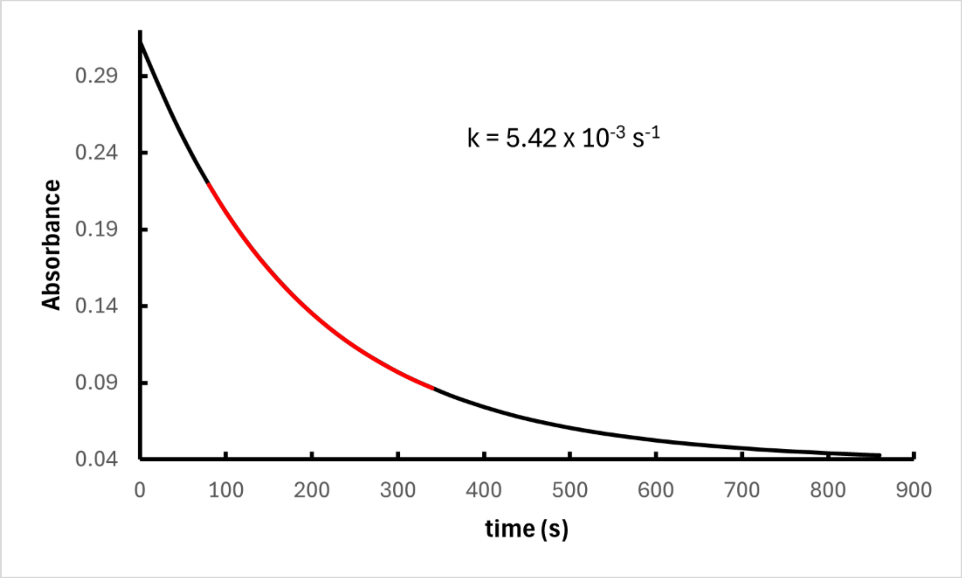

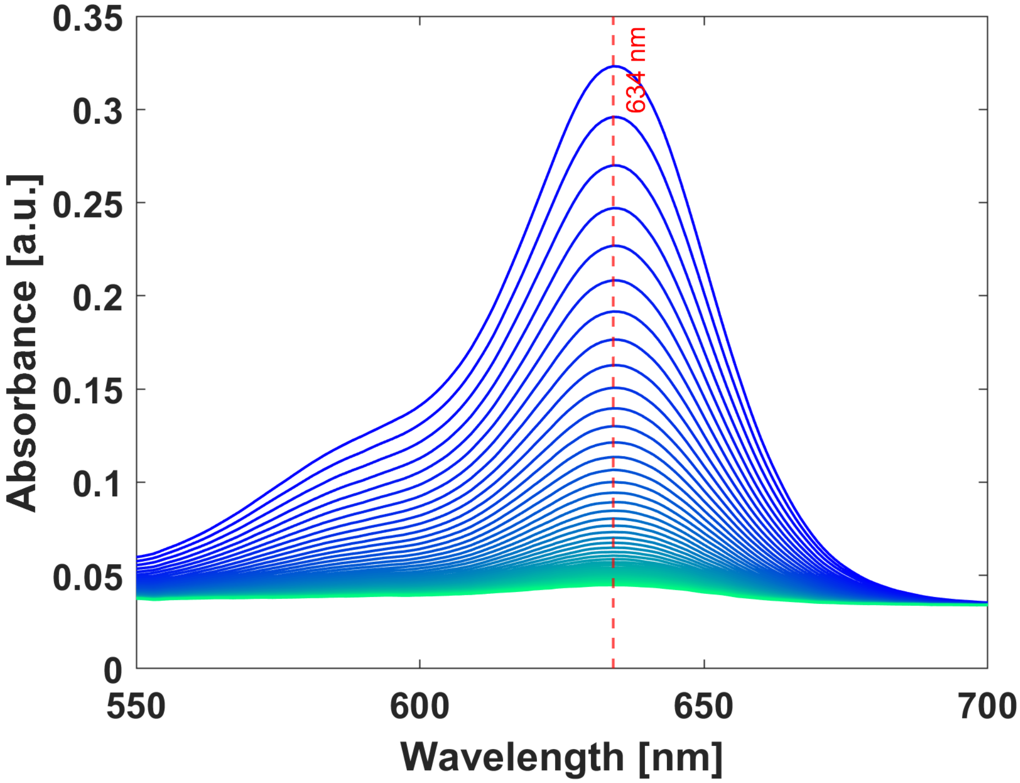
0.35
0.3
0.25
0.2
0.15
0.1
0.05
0
Absorbance [a.u.]
634 nm
550
600
650
700
Wavelength [nm]

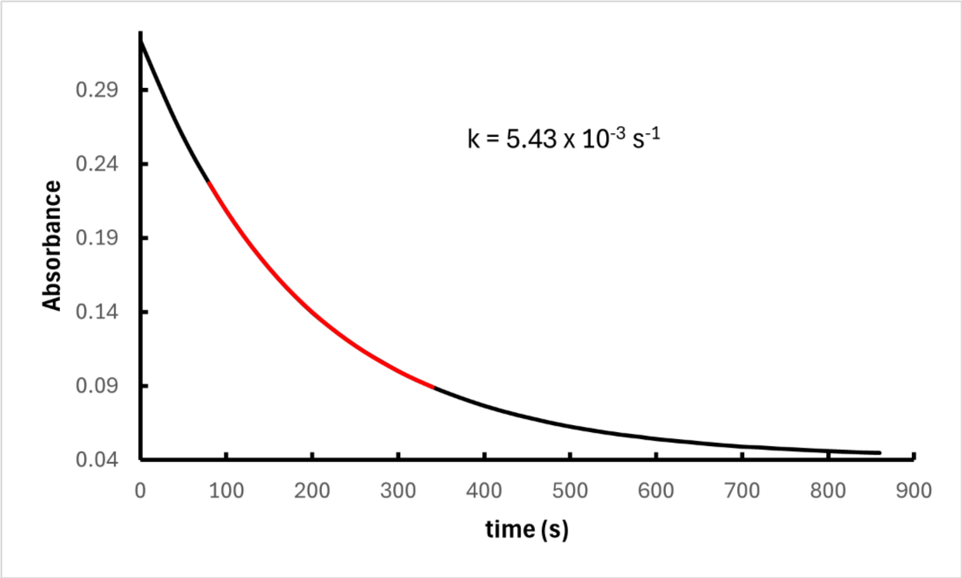
0.29
0.24
0.19
0.14
0.09
0.04
Absorbance
k = 5.43 x 10-3 s-1
0
100
200
300
400
500
600
700
800
900
time (s)

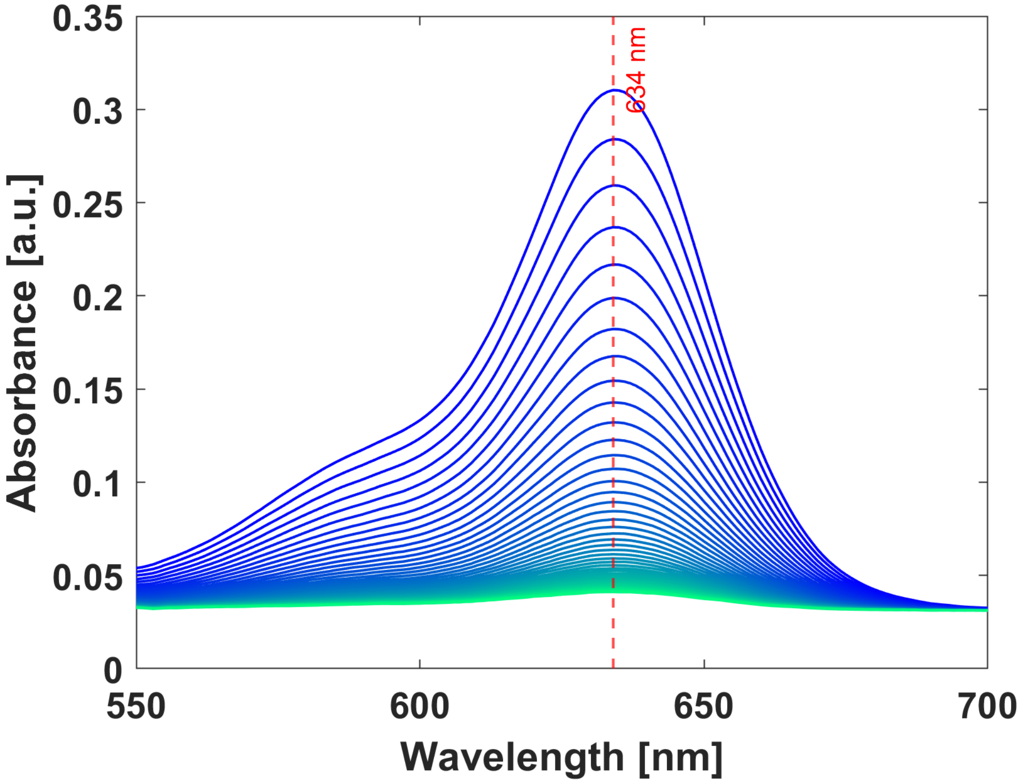

0.35
0.3
0.25
0.2
0.15
0.1
0.05
0
Absorbance [a.u.]
634 nm
550
600
650
700
Wavelength [nm]

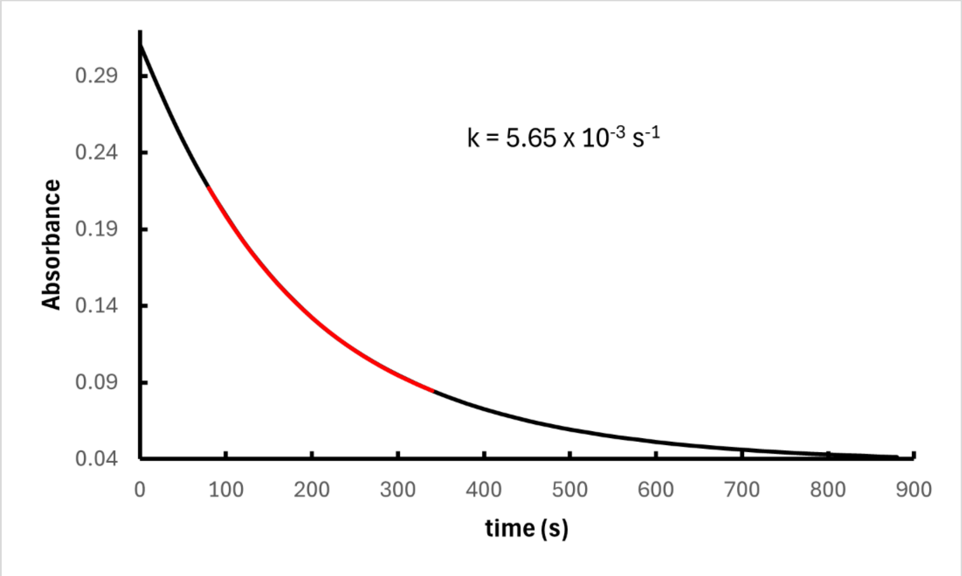

0.29
0.24
0.19
0.14
0.09
0.04
Absorbance
k = 5.65 x 10-3 s-1
0
100
200
300
400
500
600
700
800
900
time (s)

Results

Type	Rate (s^{-1})	Temperature	Type	Rate (s^{-1})	Temperature
C2	$5.81x10^{-3}$	25.6	cell	$5.42x10^{-3}$	25.4
C2	$5.57x10^{-3}$	25.6	cell	$5.43x10^{-3}$	25.5
C2	$6.32x10^{-3}$	25.7	cell	$5.65x10^{-3}$	25.6
Average:	**$5.90x10^{-3}$**	25.6	Average:	**$5.50x10^{-3}$**	25.5
Standard error:	**$2.21x10^{-4}$**		Standard error:	**$7.59x10^{-5}$**	

Reaction of *n*-butanol (N3) with electrophile E1 (70/30 v/v):
Cavity measurements (C2)

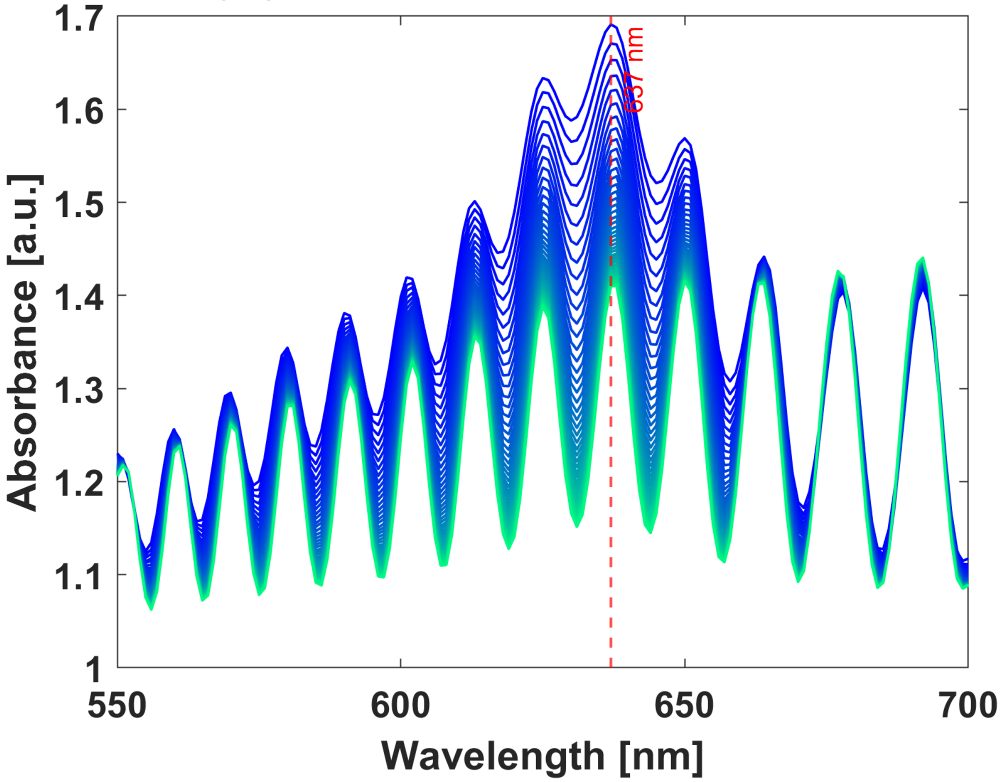

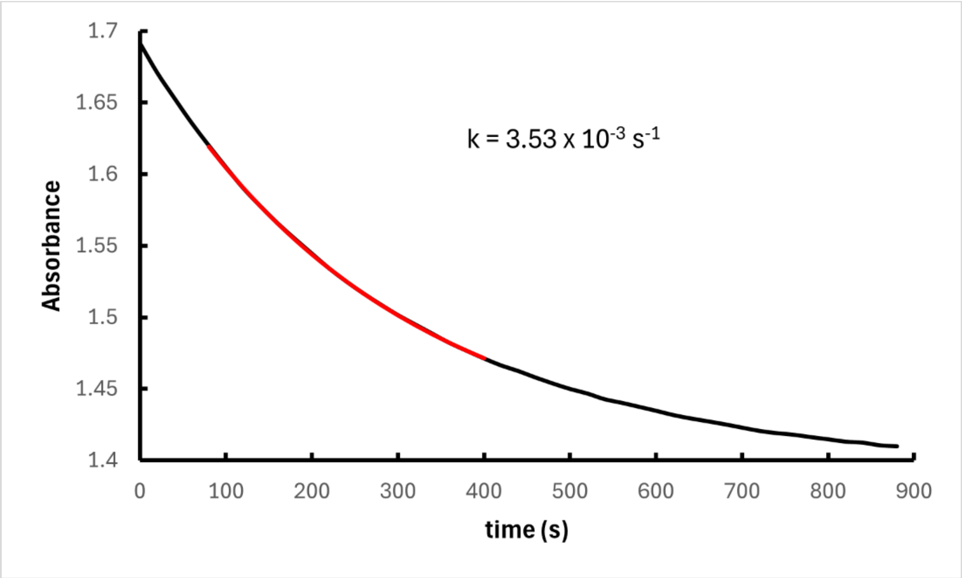

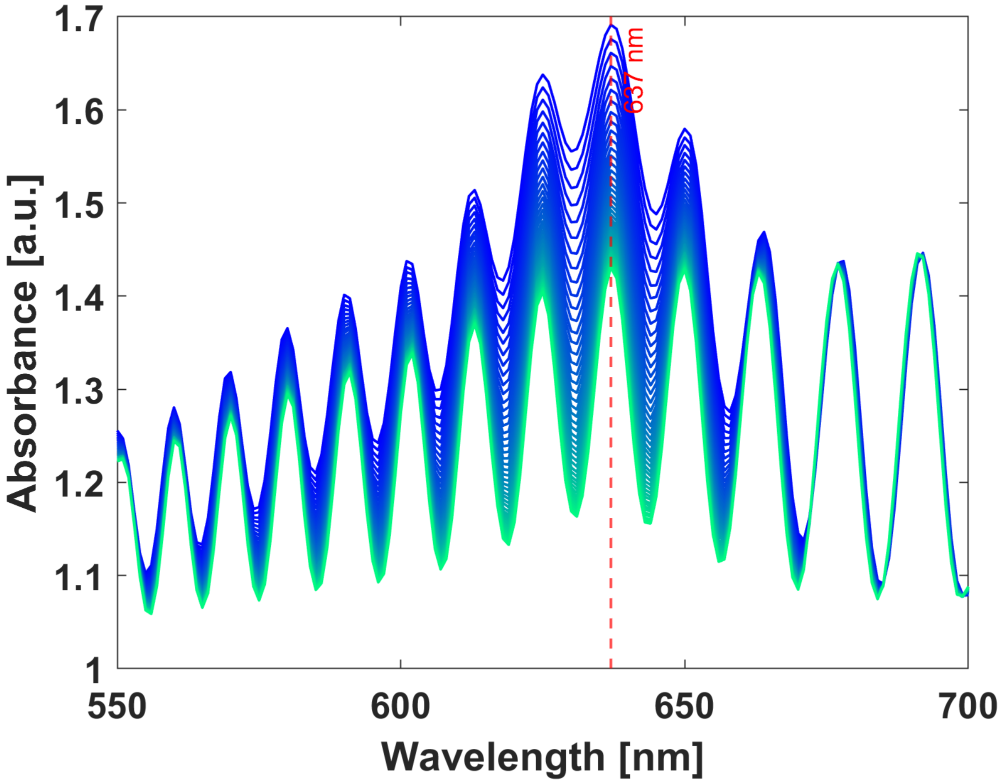

Absorbance [a.u.]
Wavelength [nm]
637 nm
1.7
1.6
1.5
1.4
1.3
1.2
1.1
1
550
600
650
700

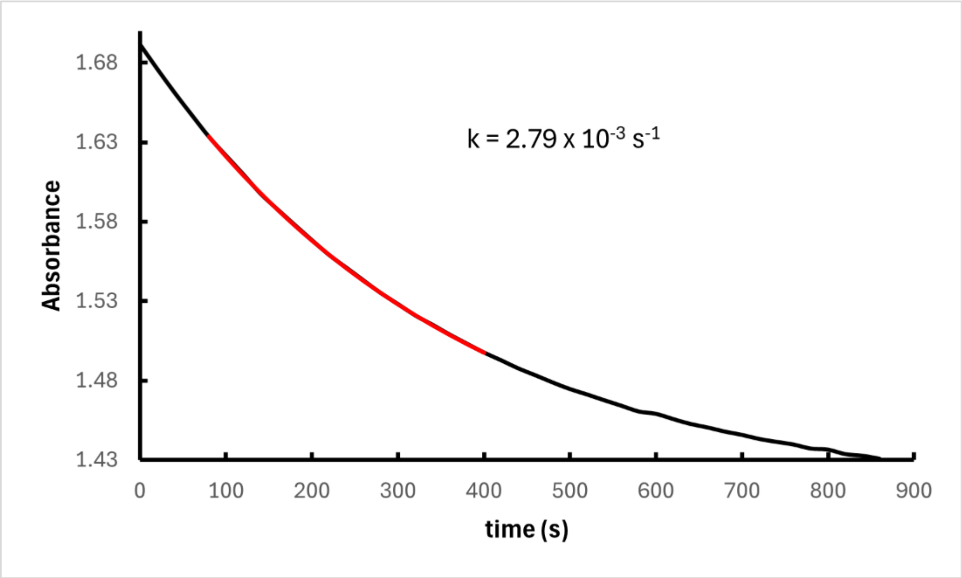

k = 2.79 x 10-3 s-1
Absorbance
time (s)
1.68
1.63
1.58
1.53
1.48
1.43
0
100
200
300
400
500
600
700
800
900

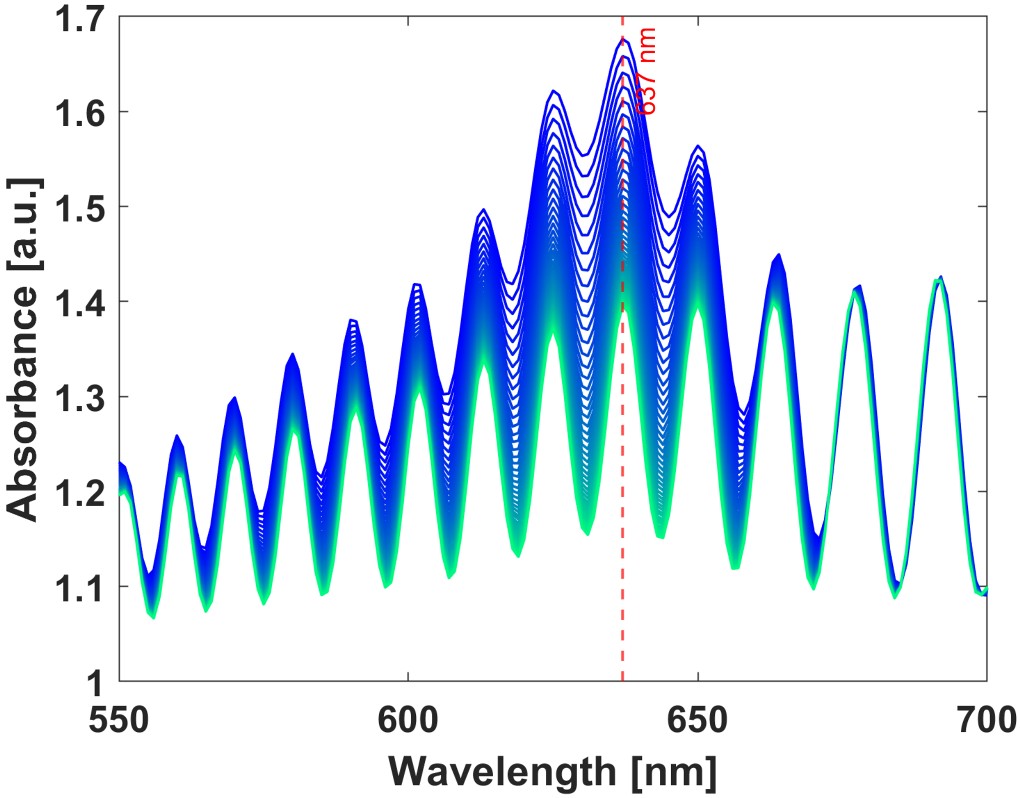
1.7
1.6
1.5
1.4
1.3
1.2
1.1
1
Absorbance [a.u.]
550
600
650
700
Wavelength [nm]
637 nm

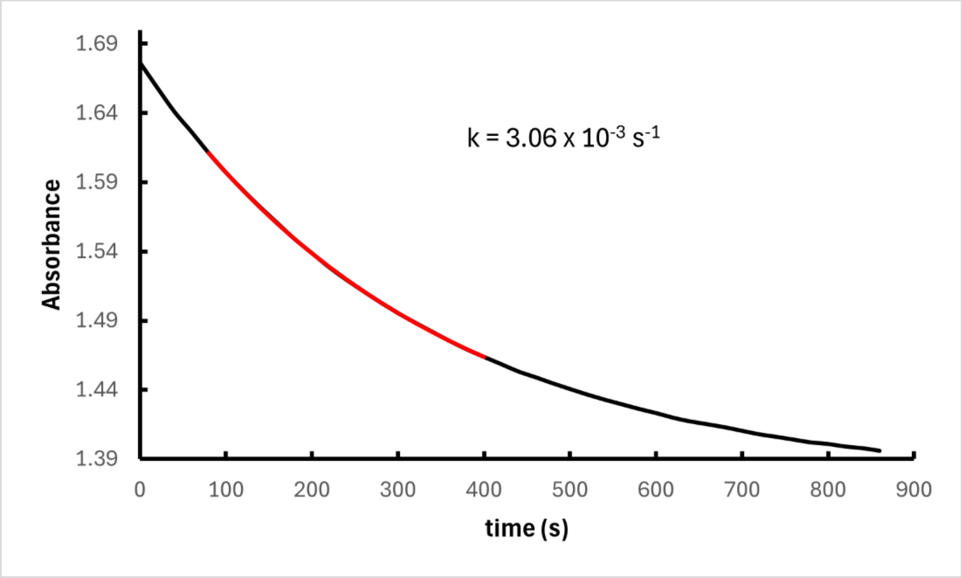
1.69
1.64
1.59
1.54
1.49
1.44
1.39
Absorbance
0
100
200
300
400
500
600
700
800
900
time (s)
k = 3.06 x 10-3 s-1

Cell measurements (standard)

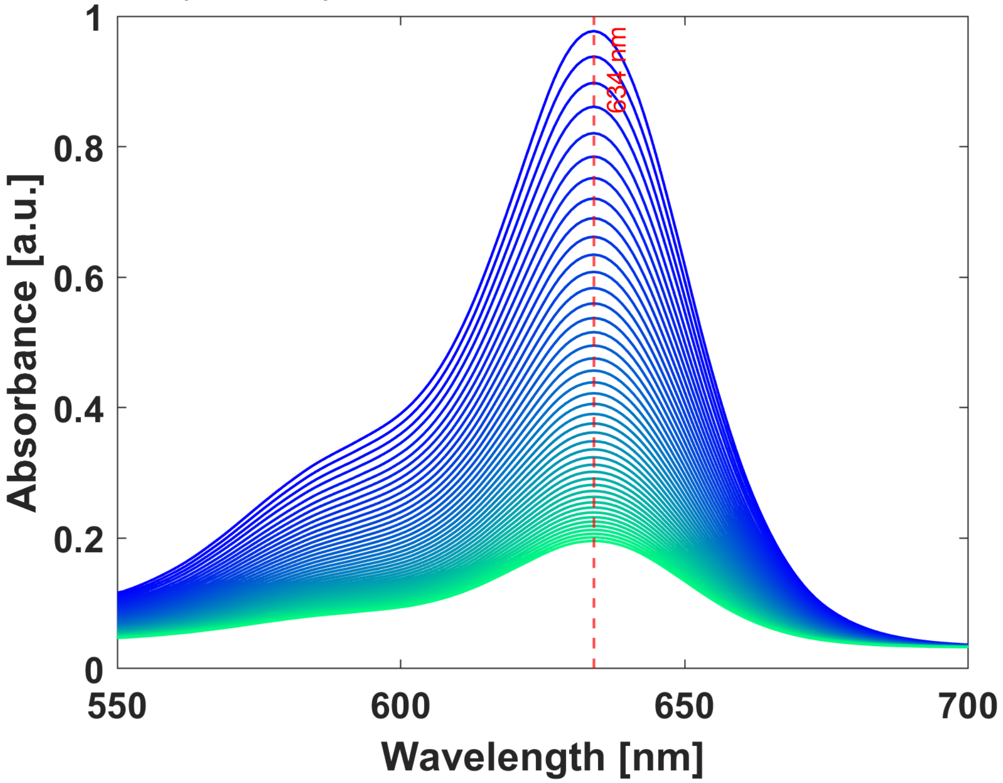

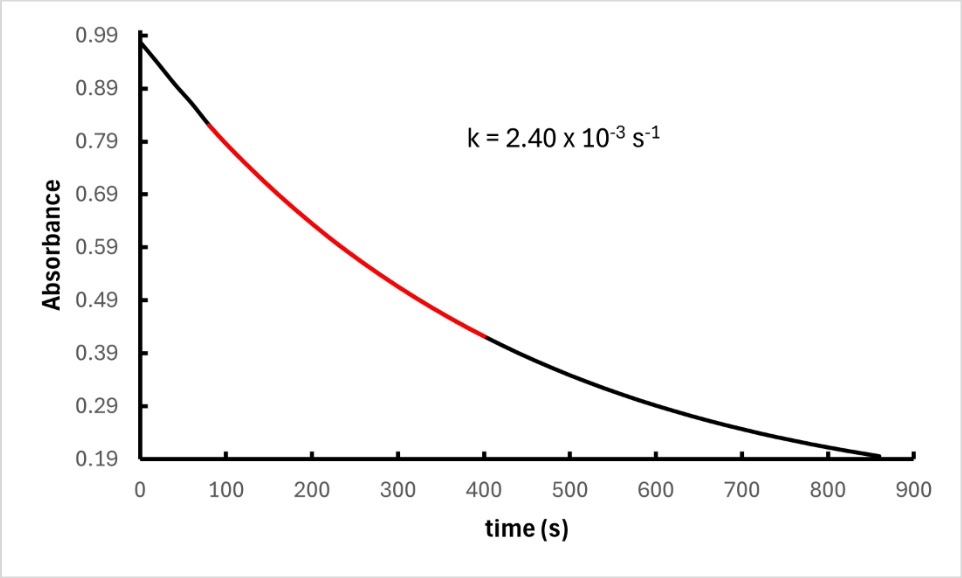

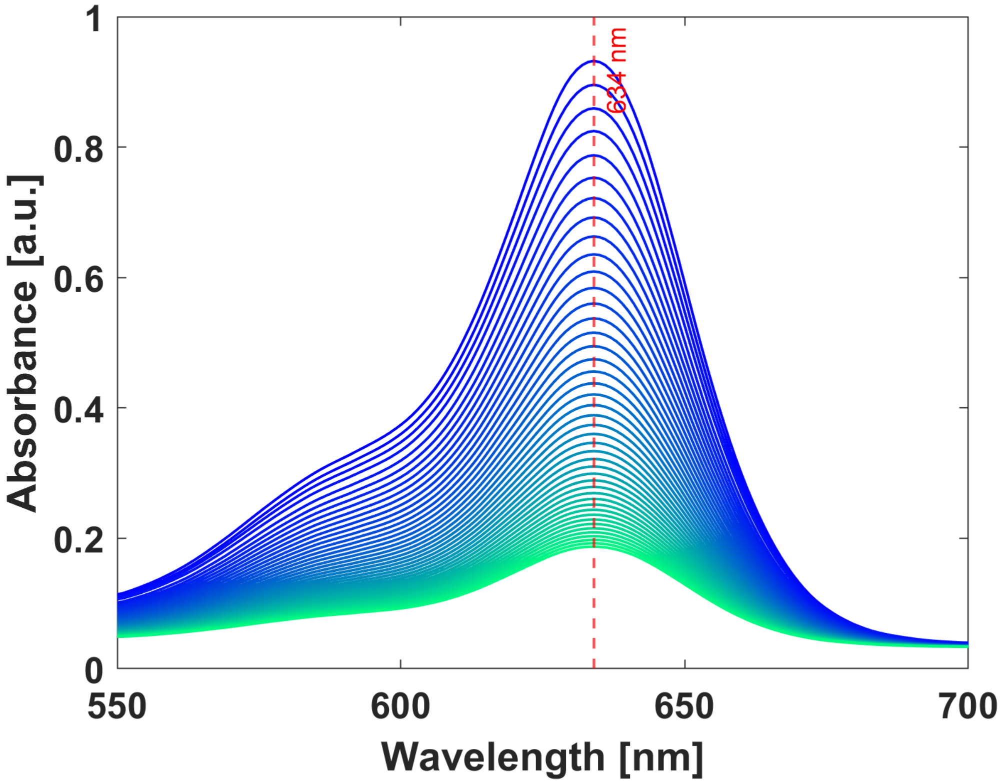
634 nm
Absorbance [a.u.]
0
0.2
0.4
0.6
0.8
1
550
600
650
700
Wavelength [nm]

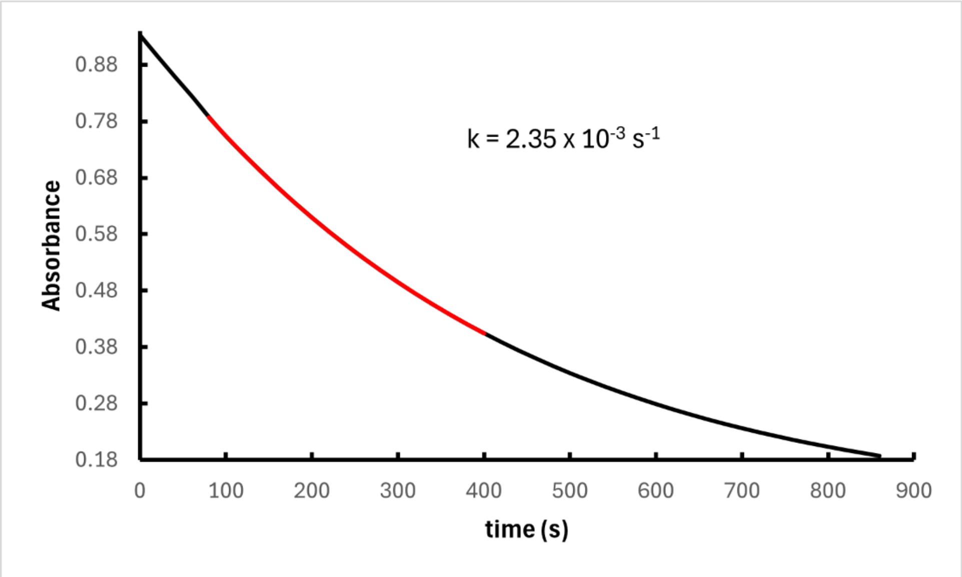
k = 2.35 x 10^{-3} s^{-1}
Absorbance
0.88
0.78
0.68
0.58
0.48
0.38
0.28
0.18
0
100
200
300
400
500
600
700
800
900
time (s)

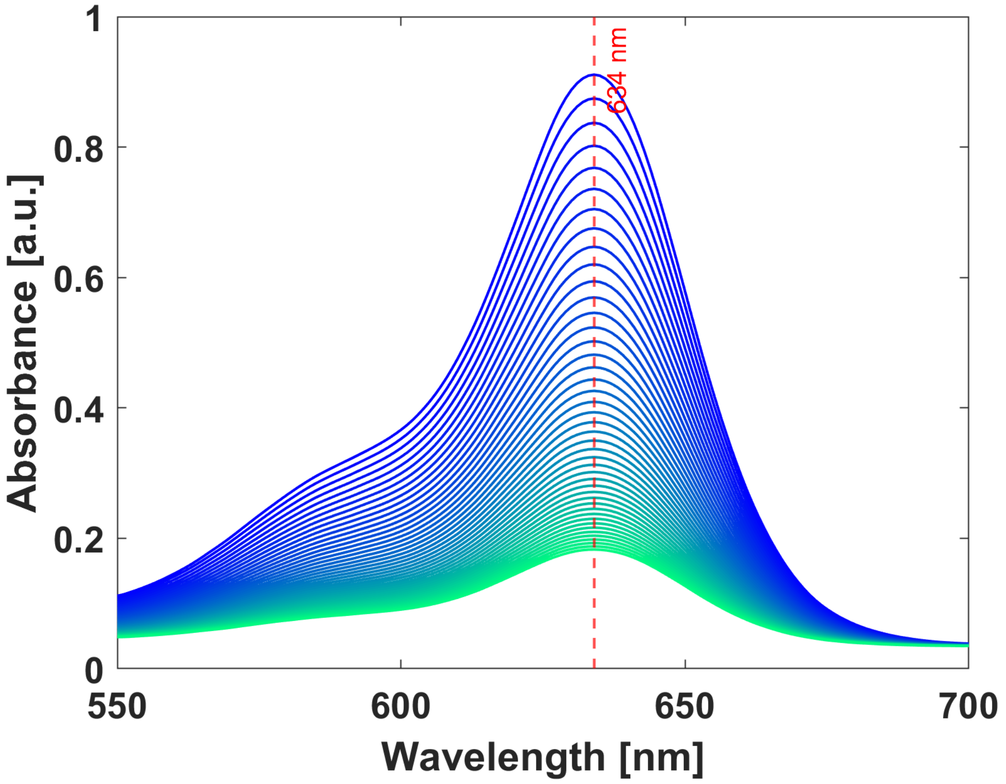
Absorbance [a.u.]
1
0.8
0.6
0.4
0.2
0
550
600
650
700
Wavelength [nm]
634 nm

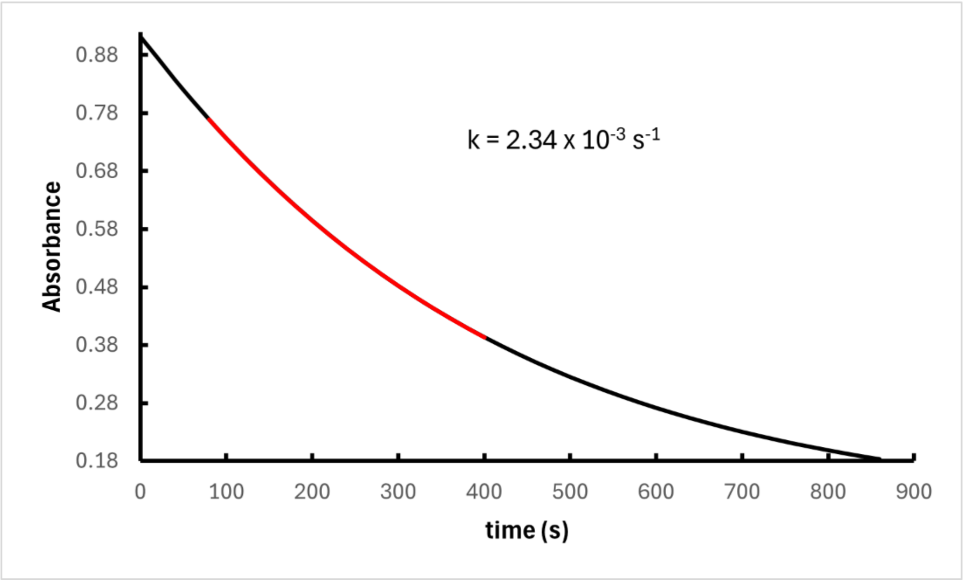
Absorbance
0.88
0.78
0.68
0.58
0.48
0.38
0.28
0.18
0
100
200
300
400
500
600
700
800
900
time (s)
k = 2.34 x 10-3 s-1

Results

Type	Rate (s^{-1})	Temperature	Type	Rate (s^{-1})	Temperature
C2	$3.53x10^{-3}$	25.8	cell	$2.40x10^{-3}$	25.9
C2	$2.79x10^{-3}$	25.9	cell	$2.35x10^{-3}$	25.9
C2	$3.06x10^{-3}$	25.9	cell	$2.34x10^{-3}$	26.0
Average:	**$3.12x10^{-3}$**	25.9	Average:	**$2.36x10^{-3}$**	25.9
Standard error:	**$2.16x10^{-4}$**		Standard error:	**$1.72x10^{-5}$**	

Dependence of the k_{vsc}/k_{cell} ratio on the concentration of alcohol:

Taking the data that was found for all different n-butanol/acetonitrile ratios (70/30 to 90/10) the following plot is obtained, which highlights that the change in rate observed is unlikely to emerge from the coupling of the OH stretch of *n*-butanol, as in that case, the opposite trend would be expected (k_{vsc}/k_{cell} higher with higher alcohol amount).

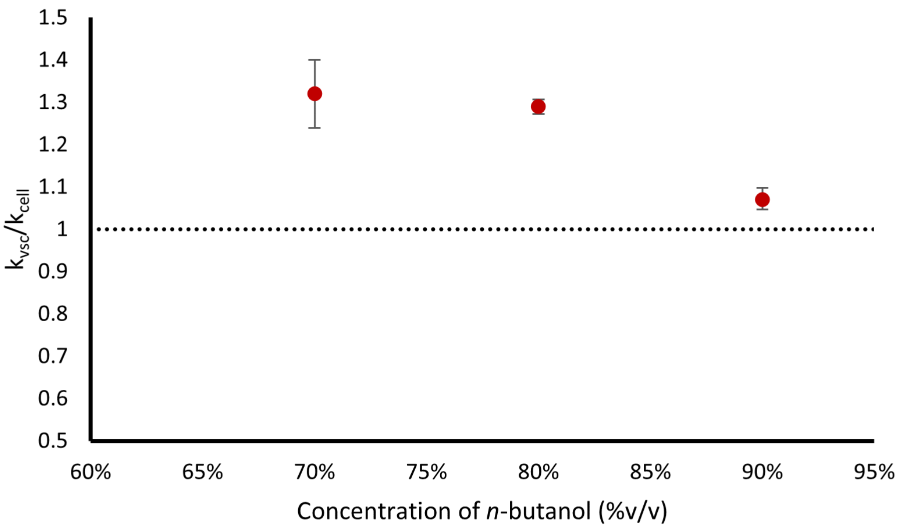

***Figure S16**: Dependence of the k_{VSC}/k_{cell} ratio on the concentration of* n-*butanol. All data was obtained on cavity C2, which couples only the OH stretch of* n-*butanol.*

Reaction of nonanol (N4) with electrophile E1:

Cavity measurements (C5):

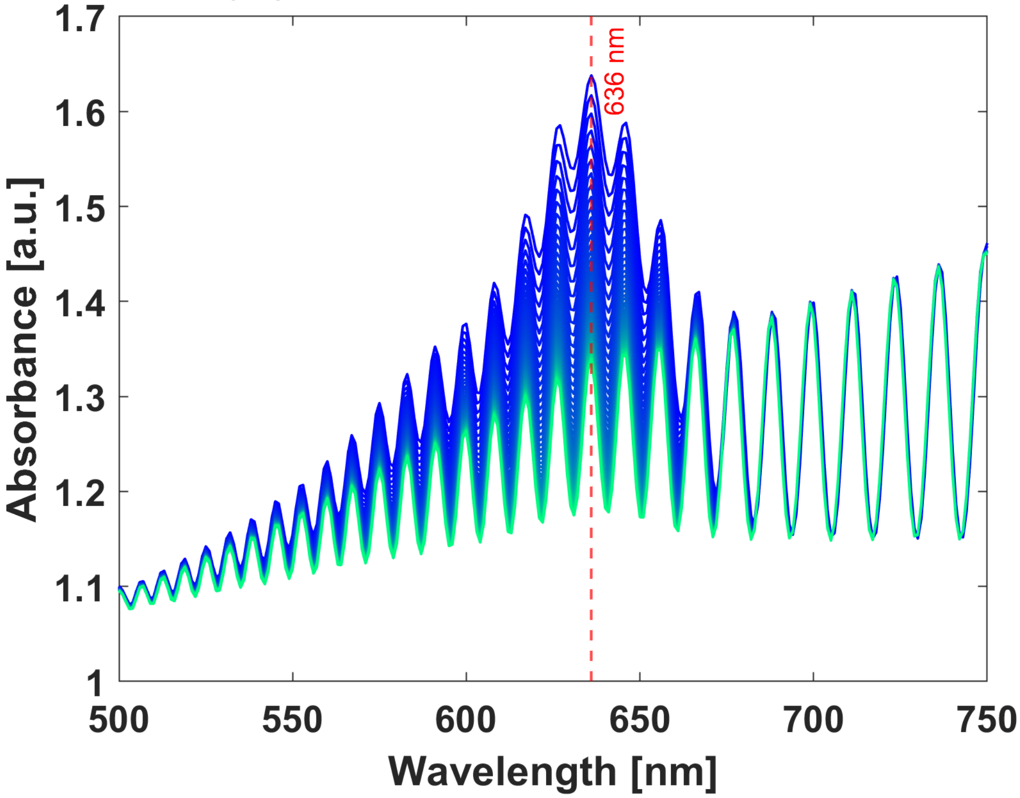

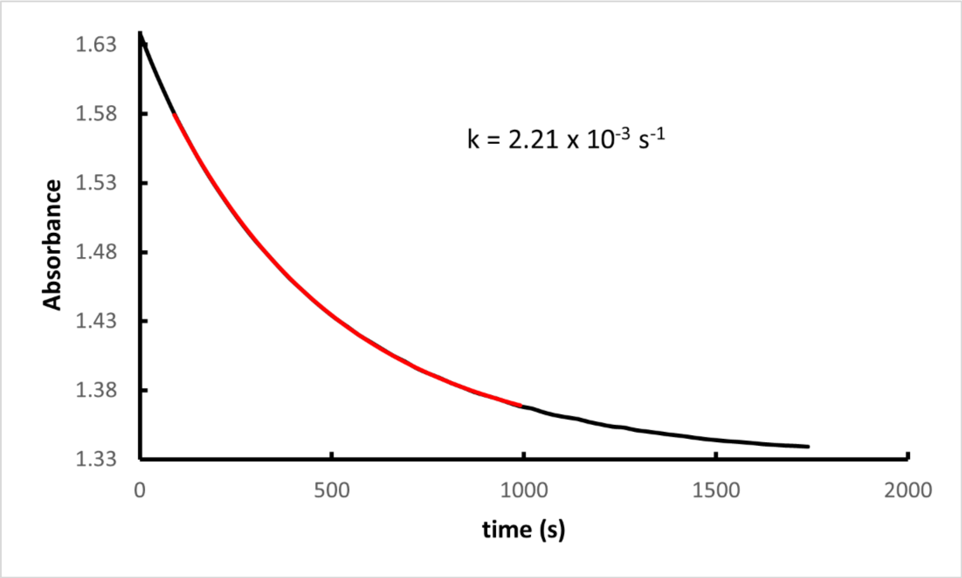

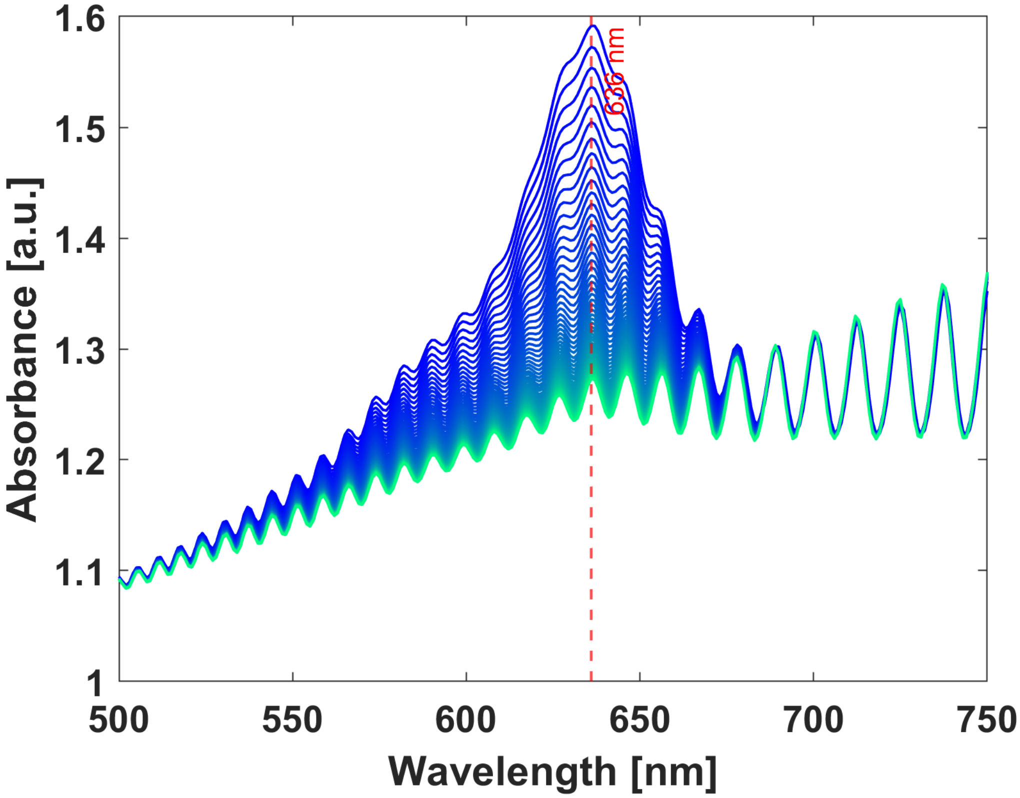

Absorbance [a.u.]
Wavelength [nm]
636 nm
1.6
1.5
1.4
1.3
1.2
1.1
1
500
550
600
650
700
750

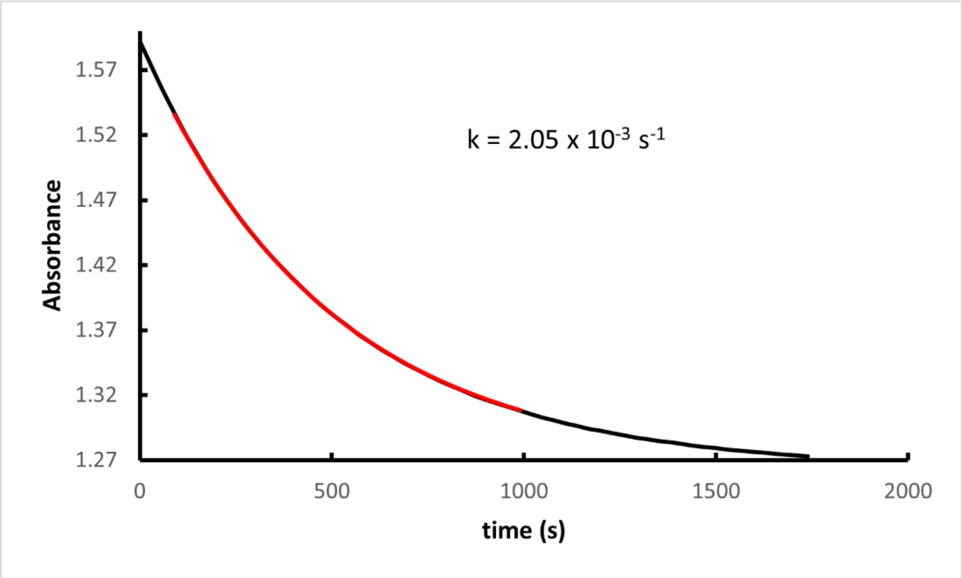

k = 2.05 x 10-3 s-1
Absorbance
time (s)
1.57
1.52
1.47
1.42
1.37
1.32
1.27
0
500
1000
1500
2000

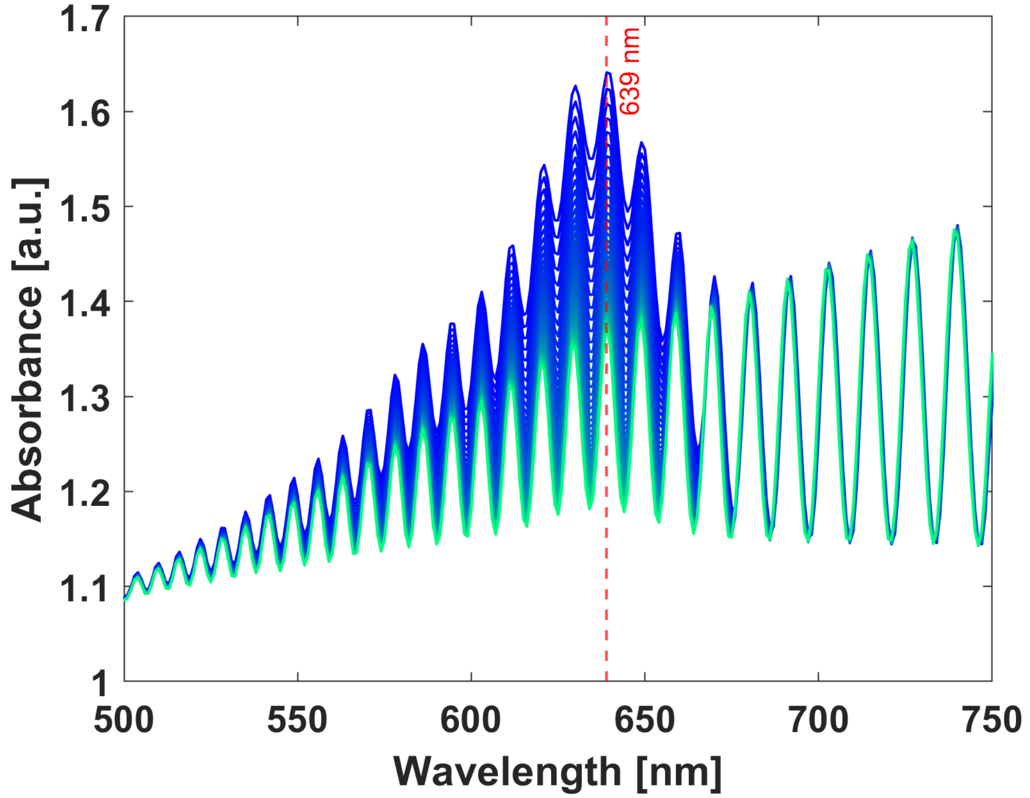
639 nm
Absorbance [a.u.]
1.7
1.6
1.5
1.4
1.3
1.2
1.1
1
500
550
600
650
700
750
Wavelength [nm]

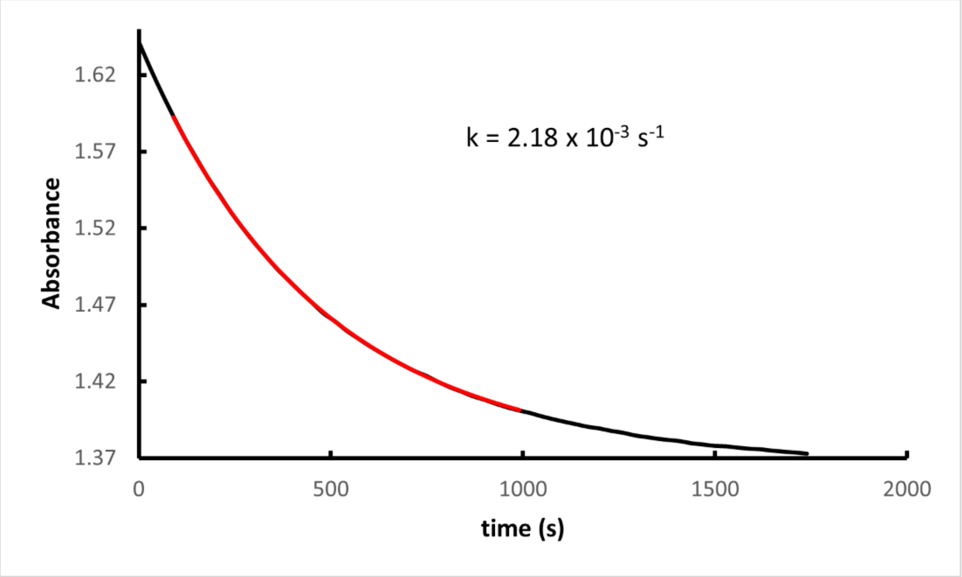
k = 2.18 x 10-3 s-1
Absorbance
1.62
1.57
1.52
1.47
1.42
1.37
0
500
1000
1500
2000
time (s)

Cell measurements (standard)

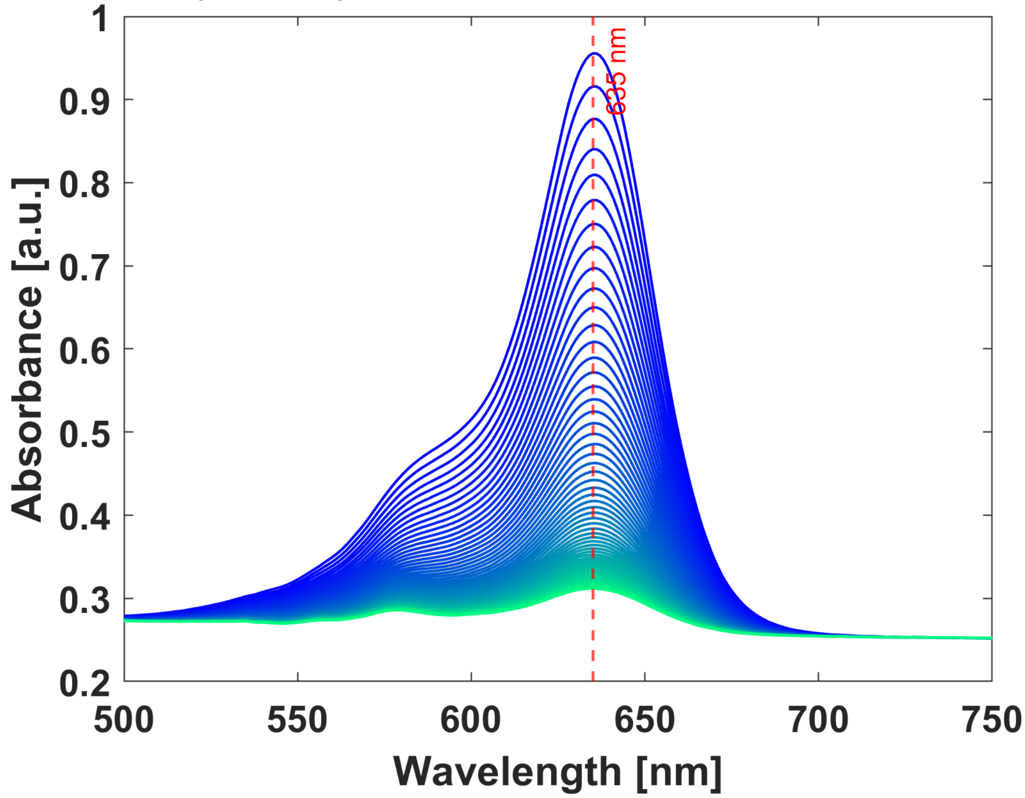

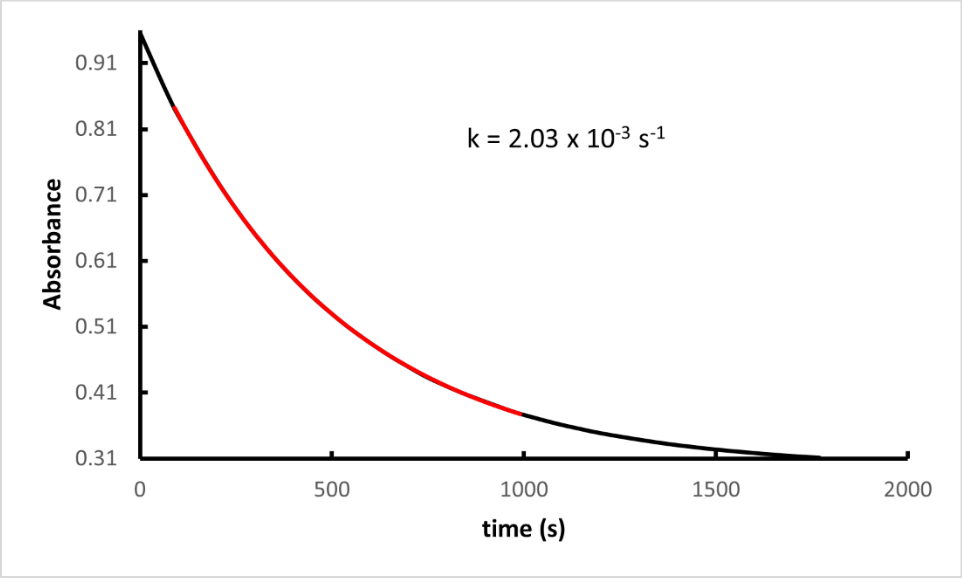

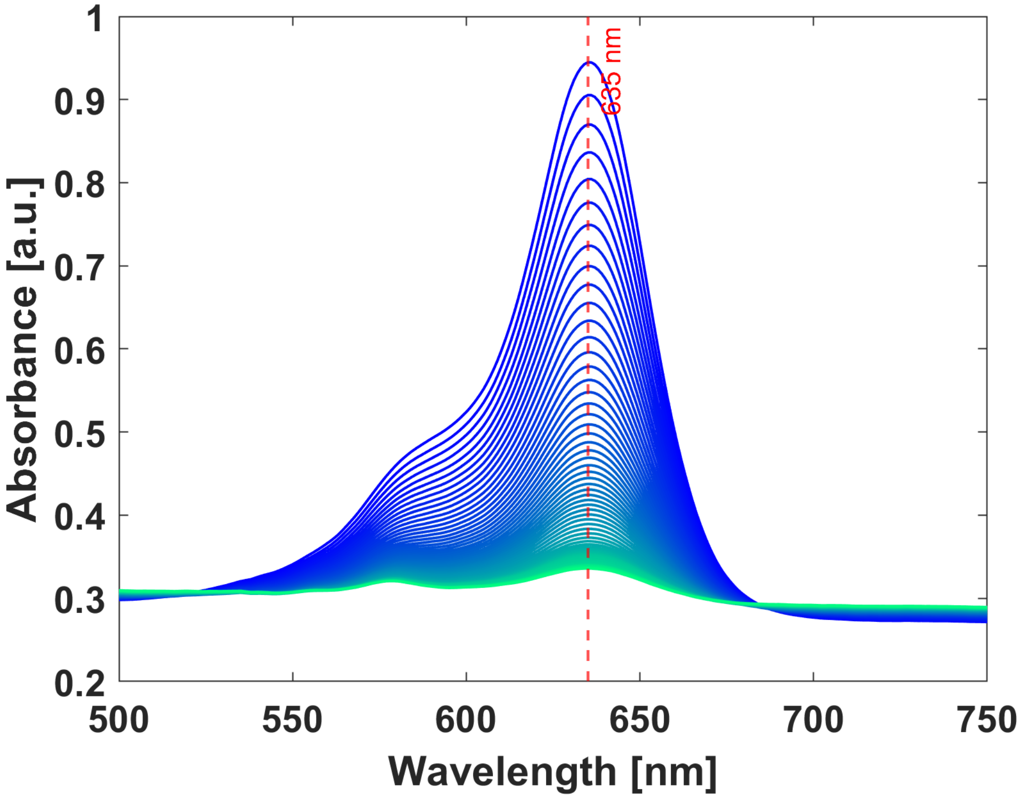

635 nm
Absorbance [a.u.]
Wavelength [nm]
0.2
0.3
0.4
0.5
0.6
0.7
0.8
0.9
1
500
550
600
650
700
750

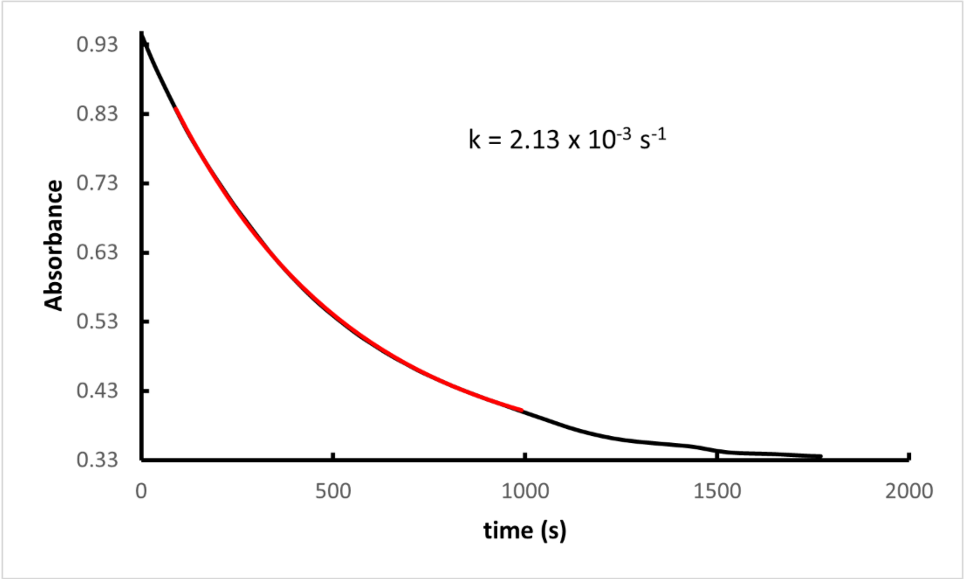

k = 2.13 x 10-3 s-1
Absorbance
time (s)
0.93
0.83
0.73
0.63
0.53
0.43
0.33
0
500
1000
1500
2000

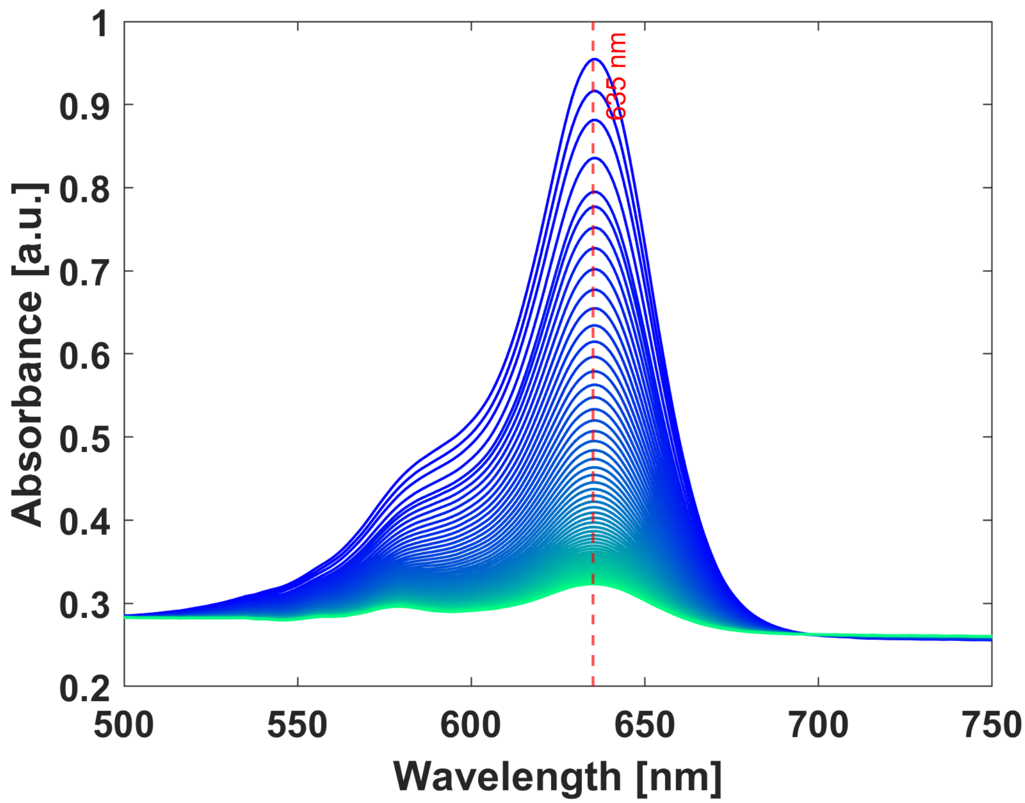

635 nm
Absorbance [a.u.]
0.2
0.3
0.4
0.5
0.6
0.7
0.8
0.9
1
500
550
600
650
700
750
Wavelength [nm]

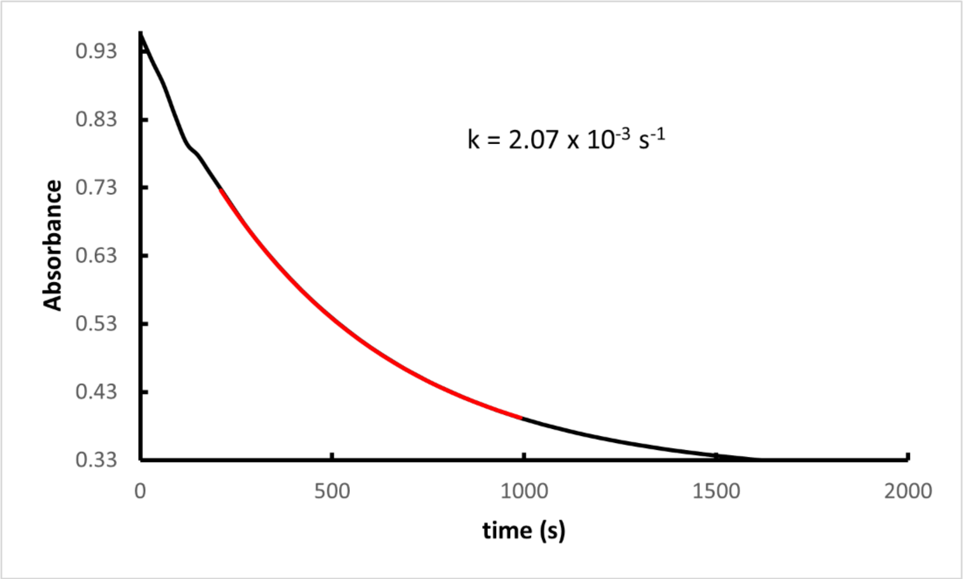

k = 2.07 x 10-3 s-1
Absorbance
0.93
0.83
0.73
0.63
0.53
0.43
0.33
0
500
1000
1500
2000
time (s)

Results

Type	Rate (s^{-1})	Temperature	Type	Rate (s^{-1})	Temperature
C5	$2.21x10^{-3}$	23.8	cell	$2.03x10^{-3}$	23.7
C5	$2.05x10^{-3}$	23.8	cell	$2.13x10^{-3}$	23.6
C5	$2.18x10^{-3}$	23.7	cell	$2.07x10^{-3}$	23.6
Average:	**$2.15x10^{-3}$**	23.8	Average:	**$2.08x10^{-3}$**	23.6
Standard error:	**$4.95x10^{-5}$**		Standard error:	**$2.84x10^{-5}$**	

Reaction of *iso*-propanol (N5) with electrophile E2:

Cavity measurements (C5)

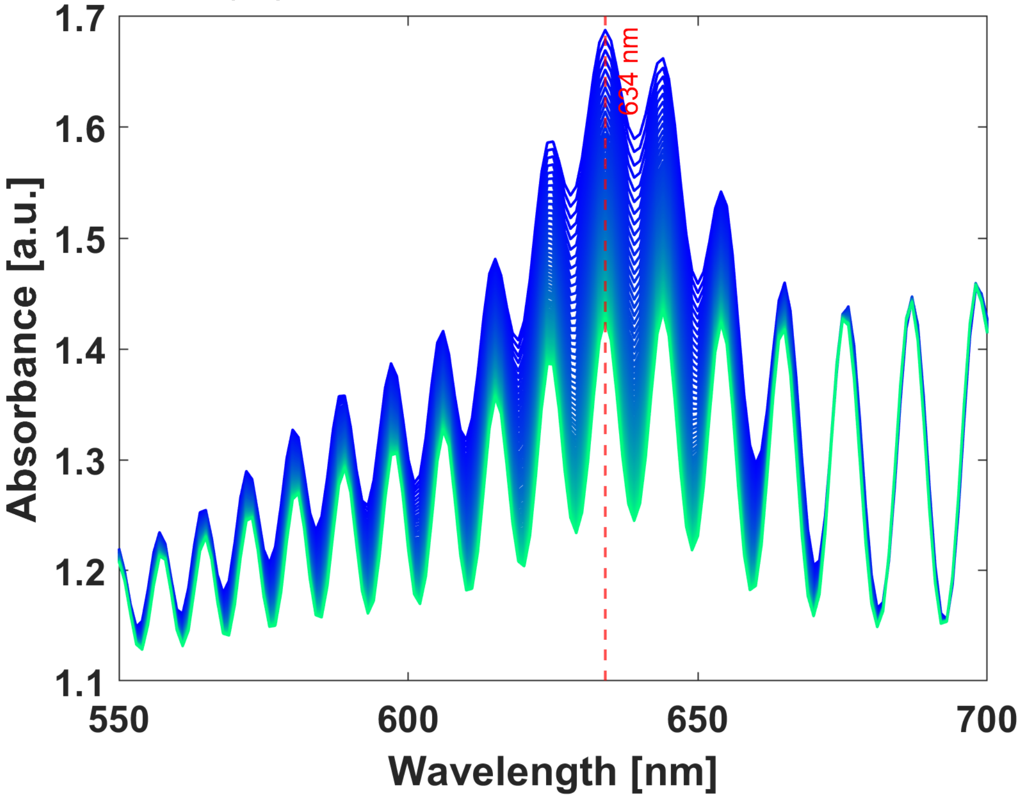

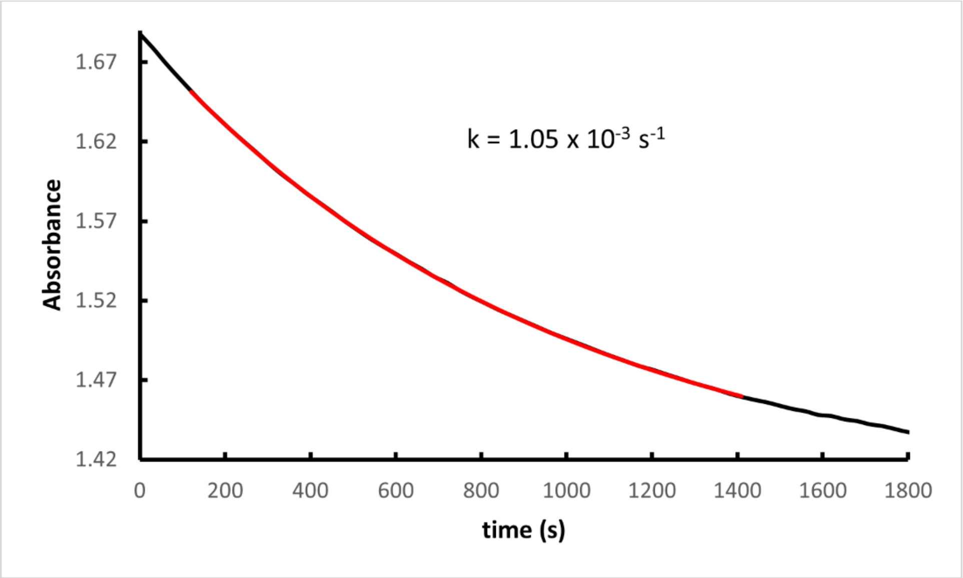

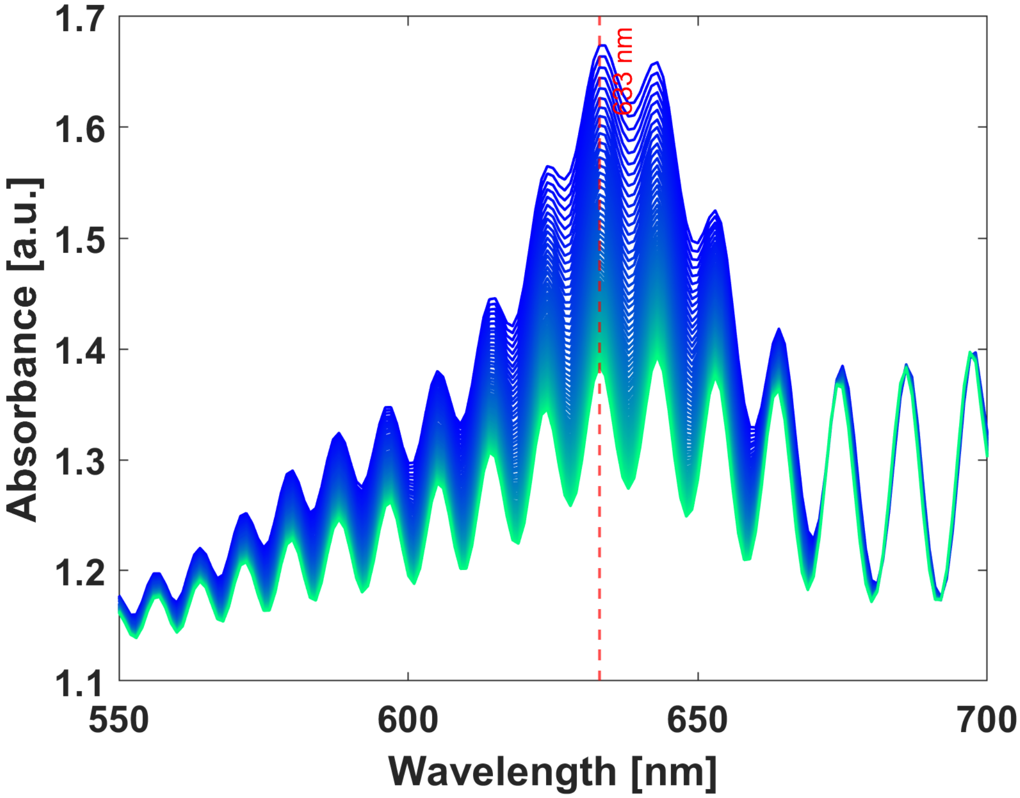

Absorbance [a.u.]
Wavelength [nm]
633 nm
1.7
1.6
1.5
1.4
1.3
1.2
1.1
550
600
650
700

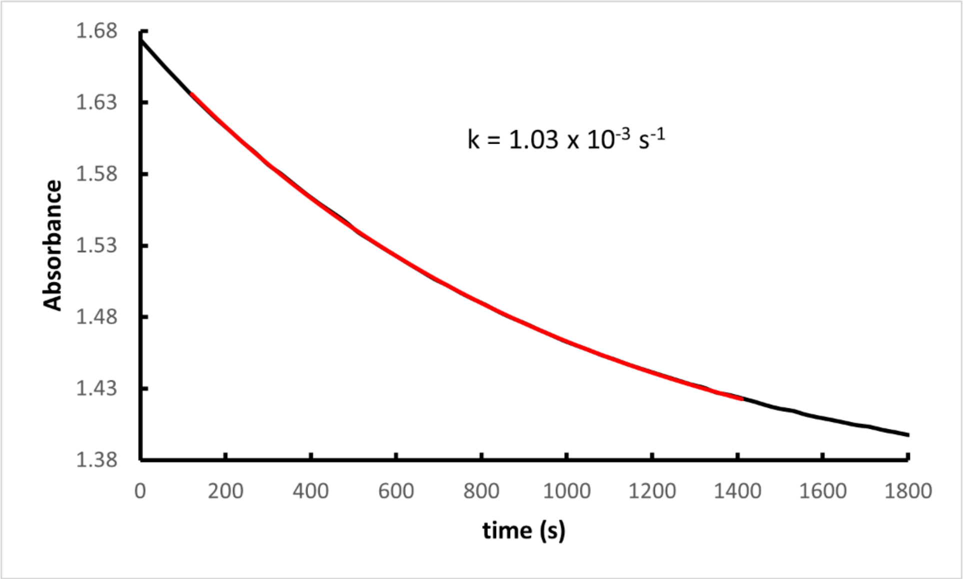

k = 1.03 x 10-3 s-1
Absorbance
time (s)
1.68
1.63
1.58
1.53
1.48
1.43
1.38
0
200
400
600
800
1000
1200
1400
1600
1800

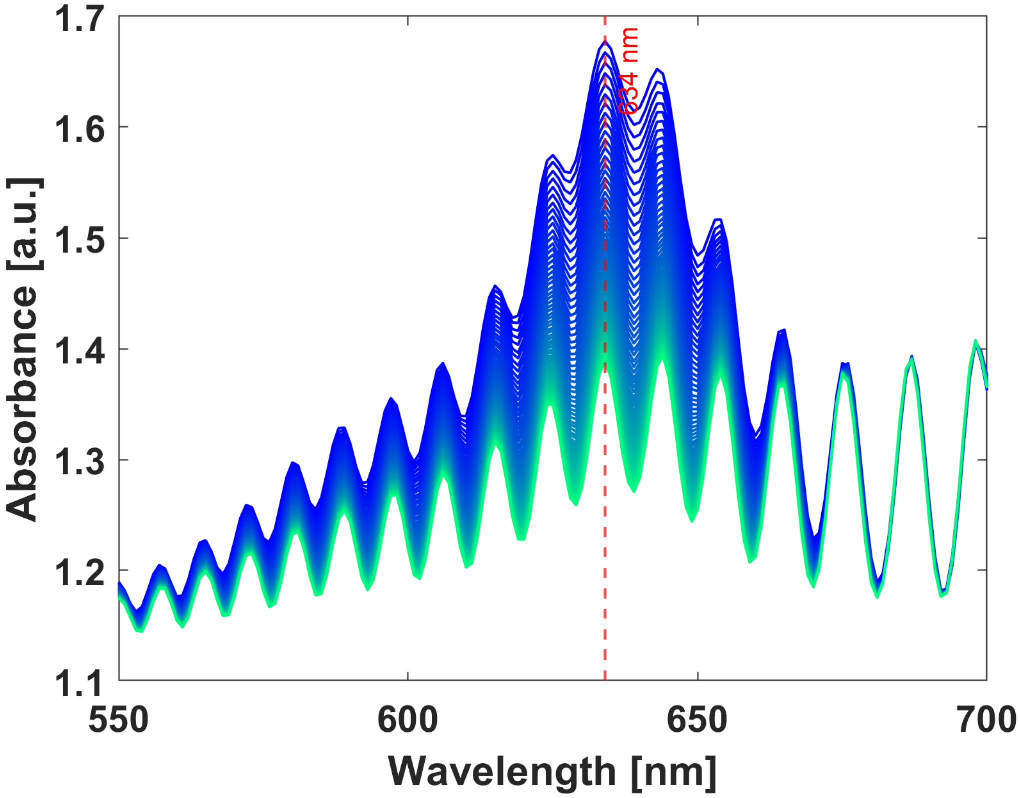

1.7
1.6
1.5
1.4
1.3
1.2
1.1
Absorbance [a.u.]
634 nm
550
600
650
700
Wavelength [nm]

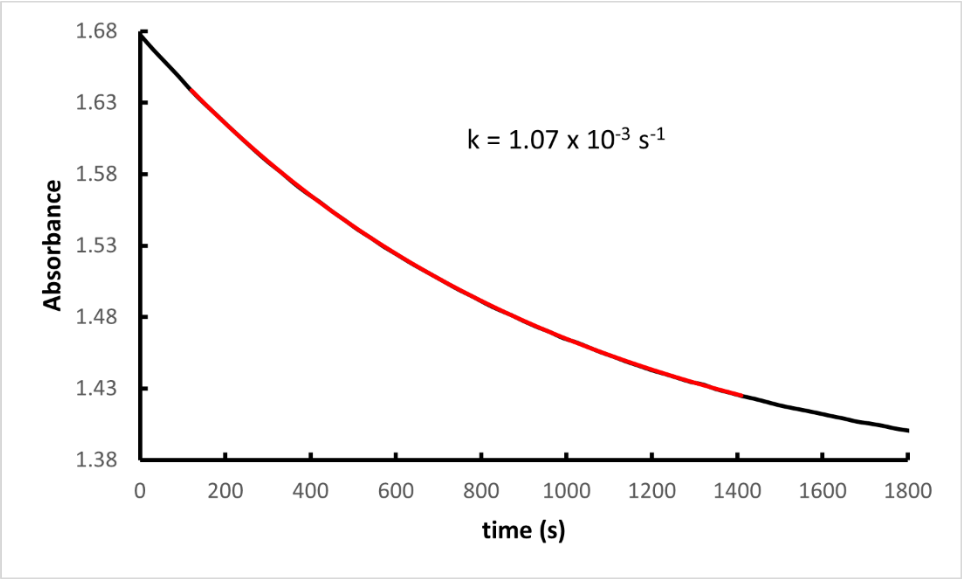

1.68
1.63
1.58
1.53
1.48
1.43
1.38
Absorbance
k = 1.07 x 10-3 s-1
0
200
400
600
800
1000
1200
1400
1600
1800
time (s)

Cell measurements (standard)

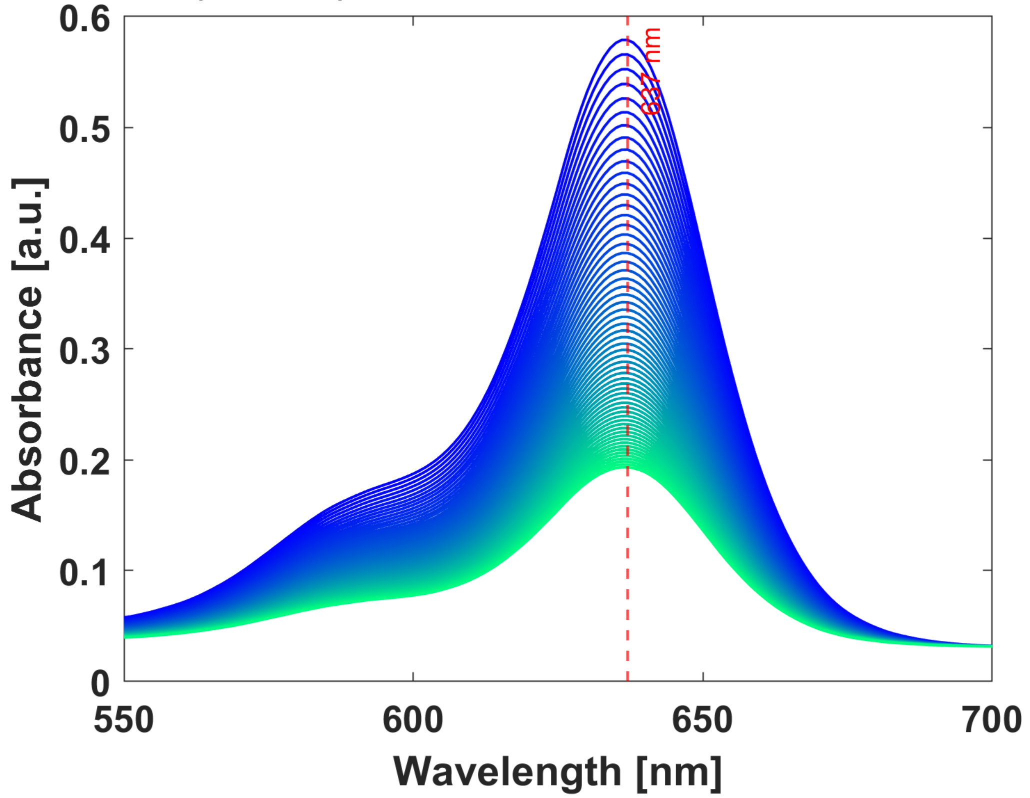

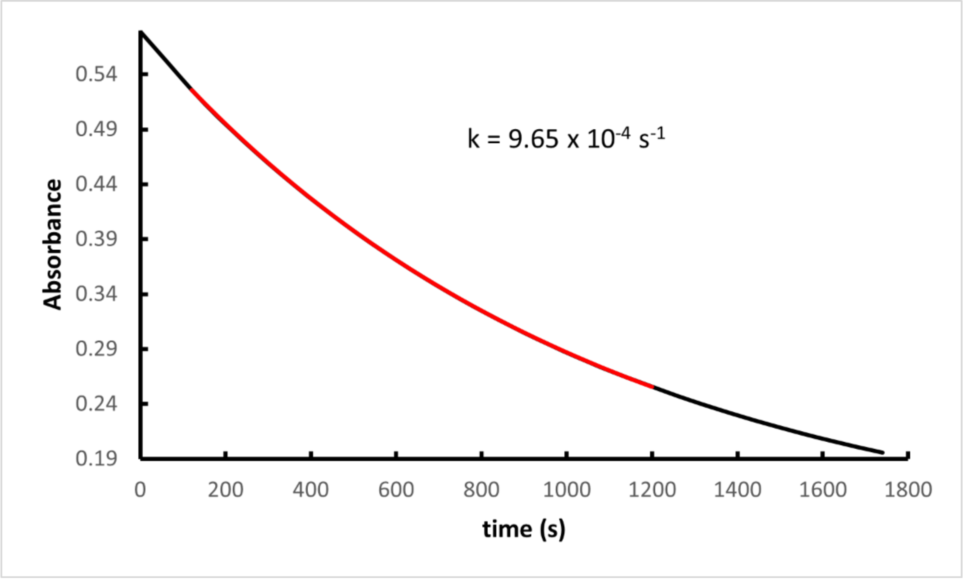

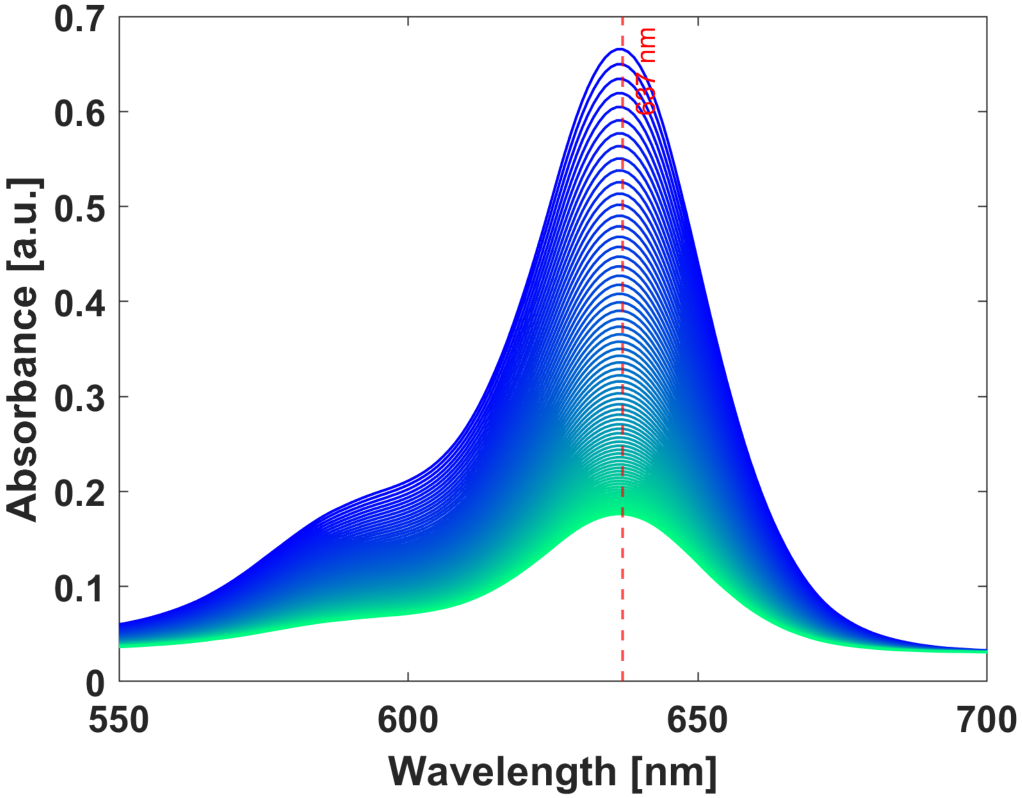
0.7
0.6
0.5
0.4
0.3
0.2
0.1
0
Absorbance [a.u.]
550
600
650
700
Wavelength [nm]
637 nm

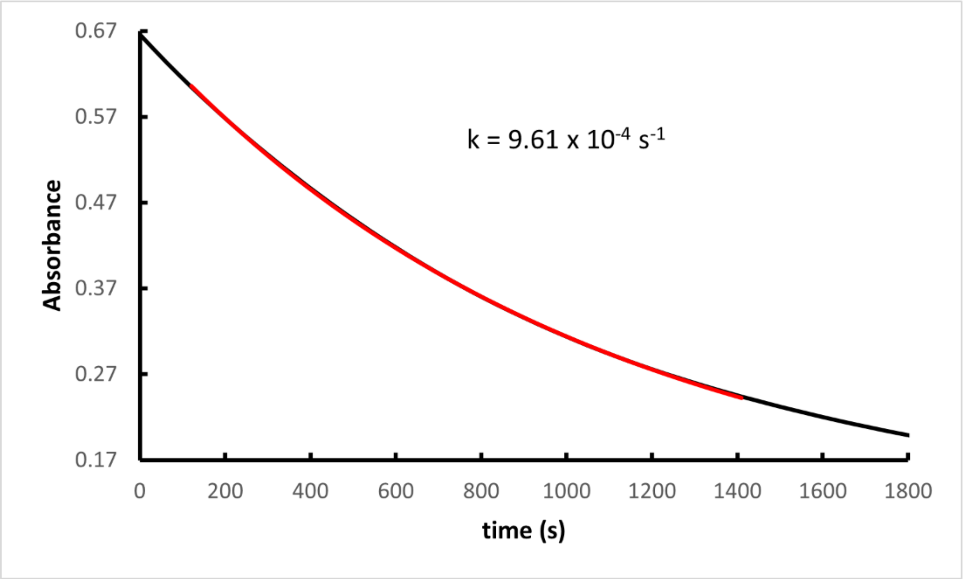
0.67
0.57
0.47
0.37
0.27
0.17
Absorbance
0
200
400
600
800
1000
1200
1400
1600
1800
time (s)
k = 9.61 x 10-4 s-1

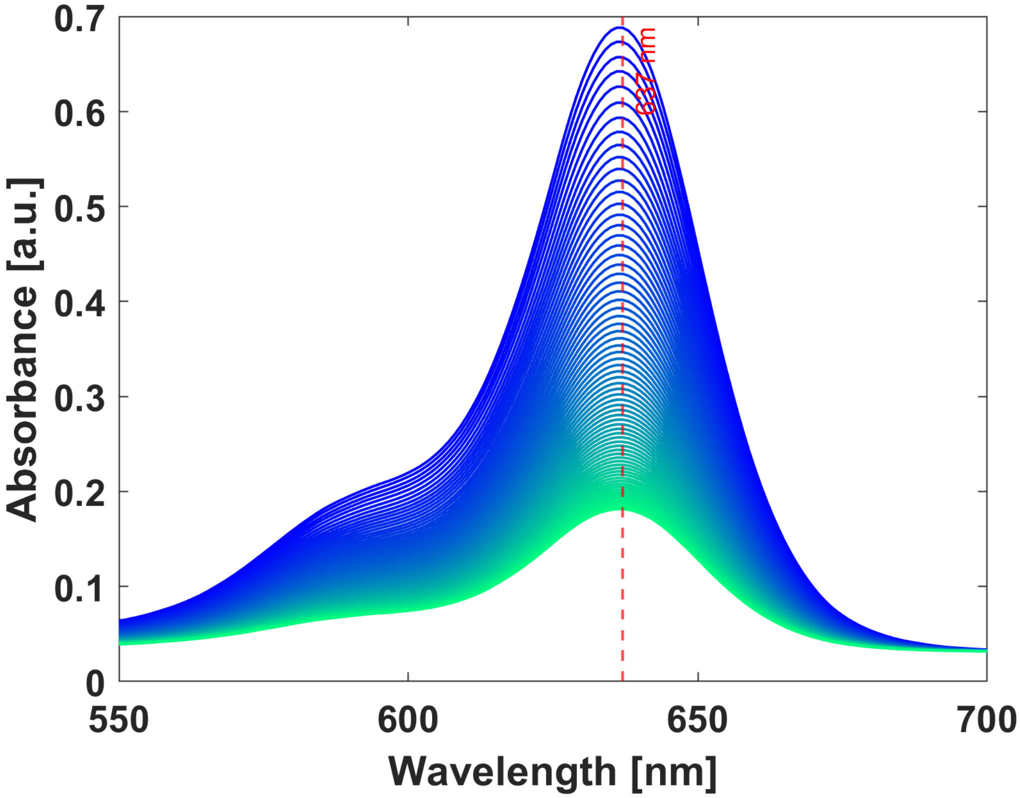

0.7
0.6
0.5
0.4
0.3
0.2
0.1
0
Absorbance [a.u.]
637 nm
550
600
650
700
Wavelength [nm]

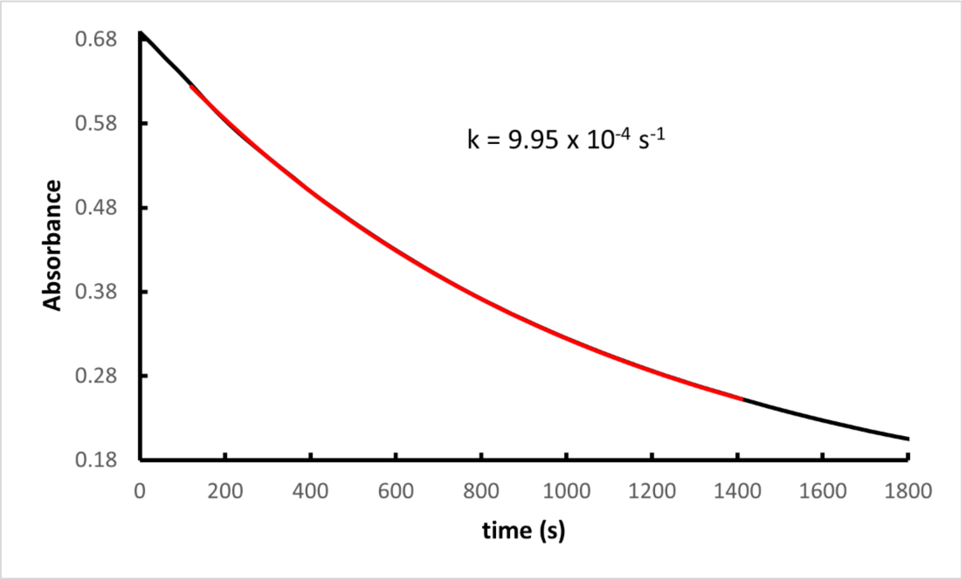

0.68
0.58
0.48
0.38
0.28
0.18
Absorbance
k = 9.95 x 10-4 s-1
0
200
400
600
800
1000
1200
1400
1600
1800
time (s)

Results

Type	Rate (s^{-1})	Temperature	Type	Rate (s^{-1})	Temperature
C5	$9.65x10^{-4}$	19.9	cell	$1.05x10^{-3}$	20.1
C5	$9.61x10^{-4}$	20	cell	$1.03x10^{-3}$	20.1
C5	$9.95x10^{-4}$	20	cell	$1.07x10^{-3}$	20.2
Average:	**$9.74x10^{-4}$**	20.0	Average:	**$1.05x10^{-3}$**	20.1
Standard error:	**$1.06x10^{-5}$**		Standard error:	**$9.07x10^{-6}$**	

Reaction of *iso*-butanol (N6) with electrophile E2:

Cavity measurements (C5)

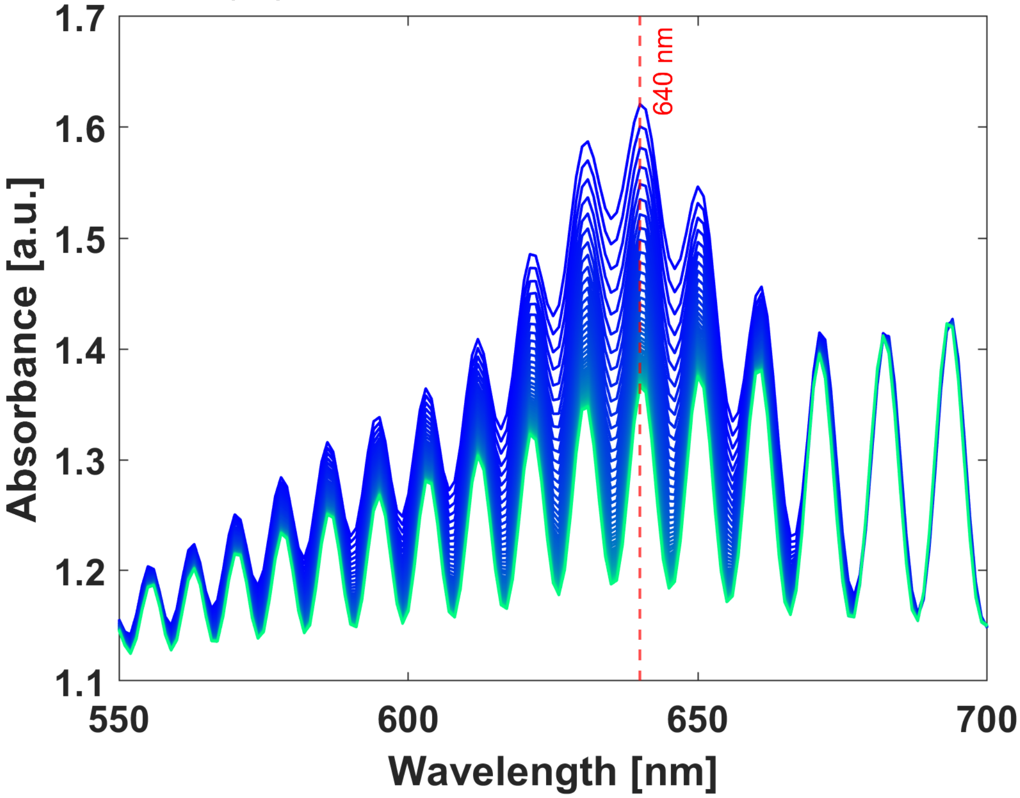

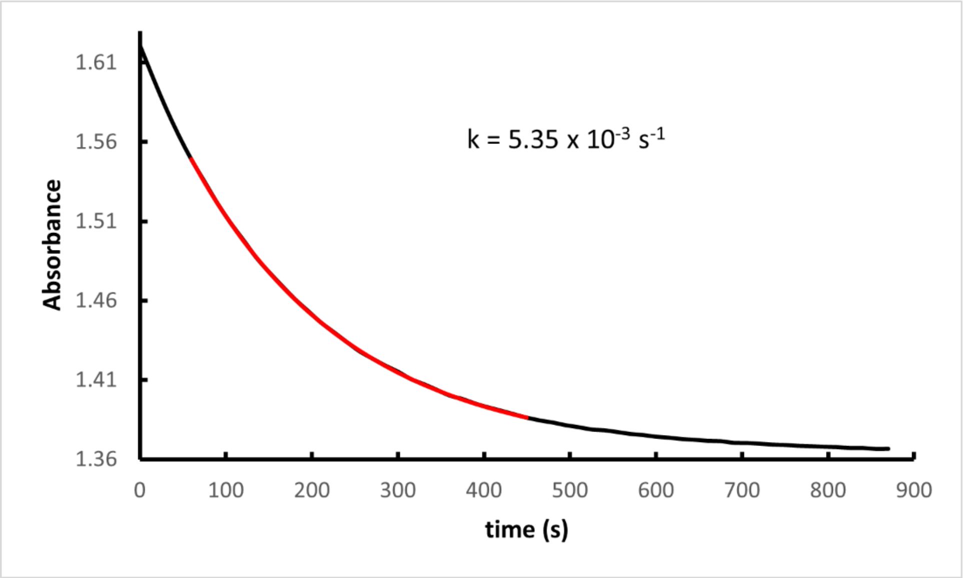

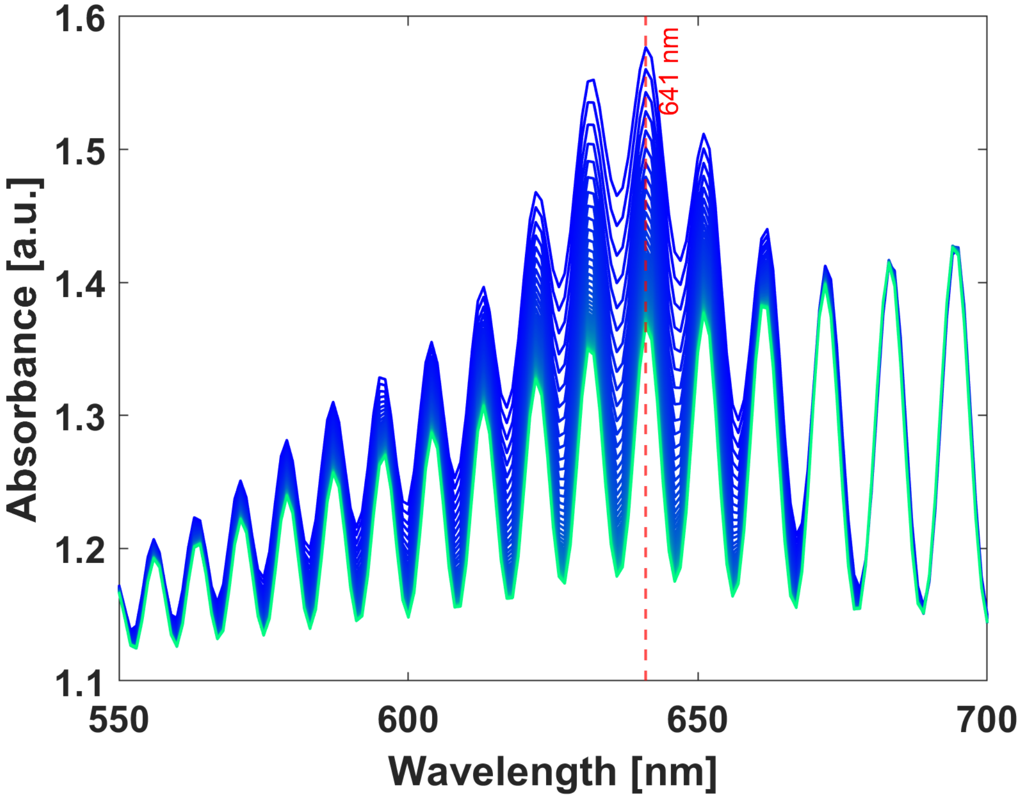

1.6
1.5
1.4
1.3
1.2
1.1
Absorbance [a.u.]
641 nm
550
600
650
700
Wavelength [nm]

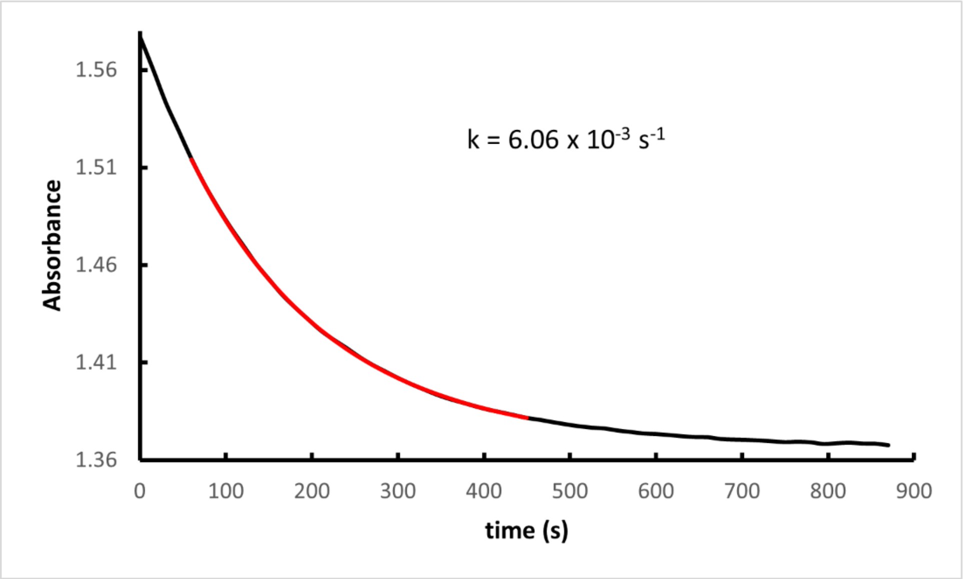

k = 6.06 x 10-3 s-1
Absorbance
1.56
1.51
1.46
1.41
1.36
0
100
200
300
400
500
600
700
800
900
time (s)

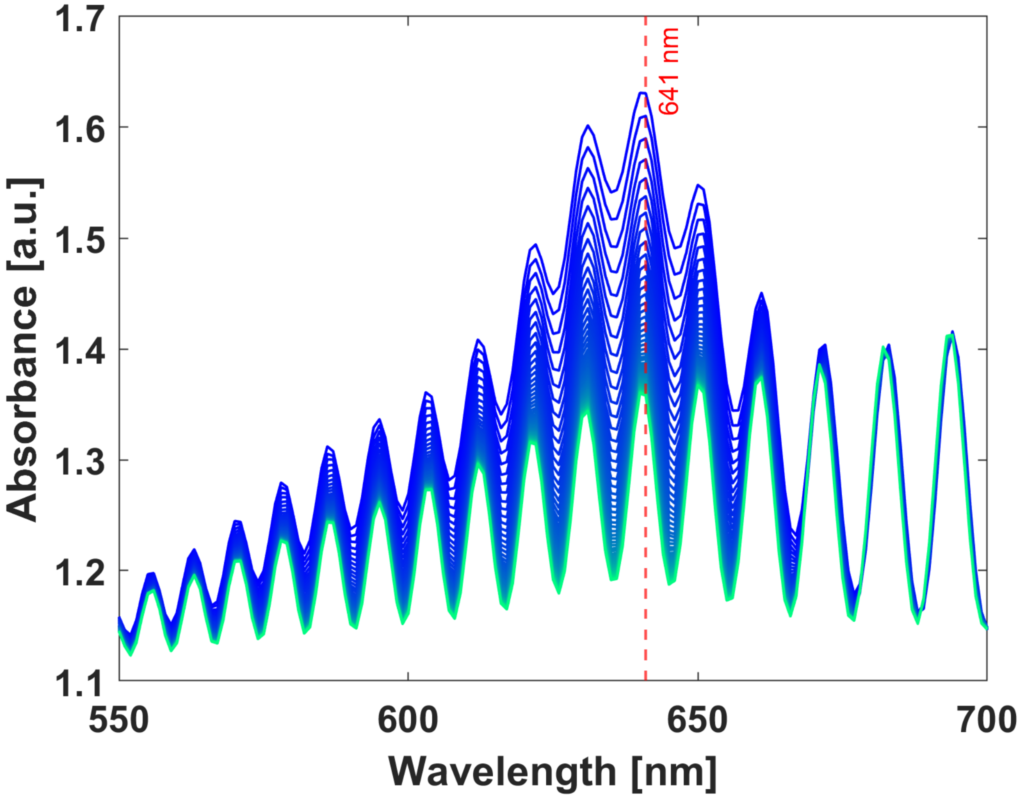

641 nm
Absorbance [a.u.]
Wavelength [nm]

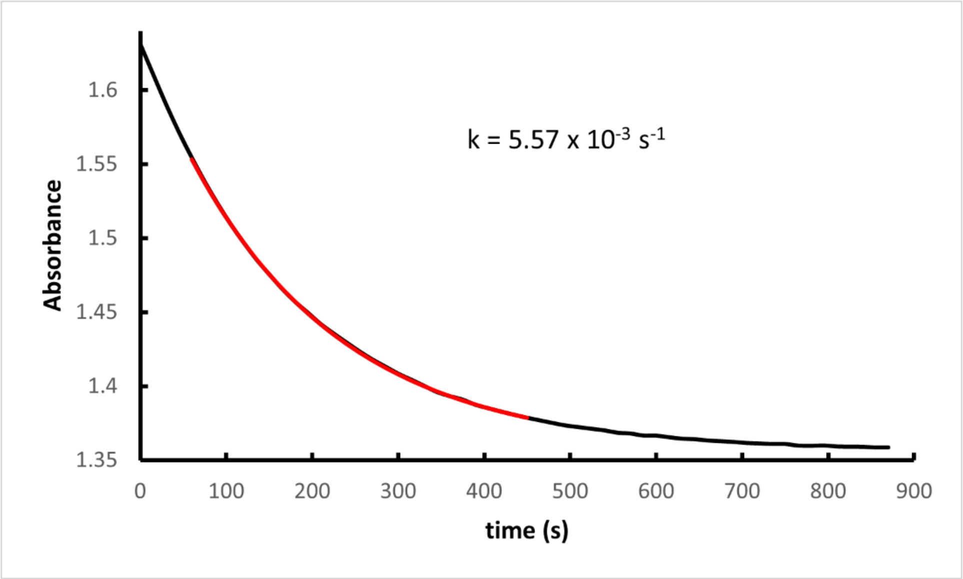

k = 5.57 x 10-3 s-1
Absorbance
time (s)

Cell measurements (standard)

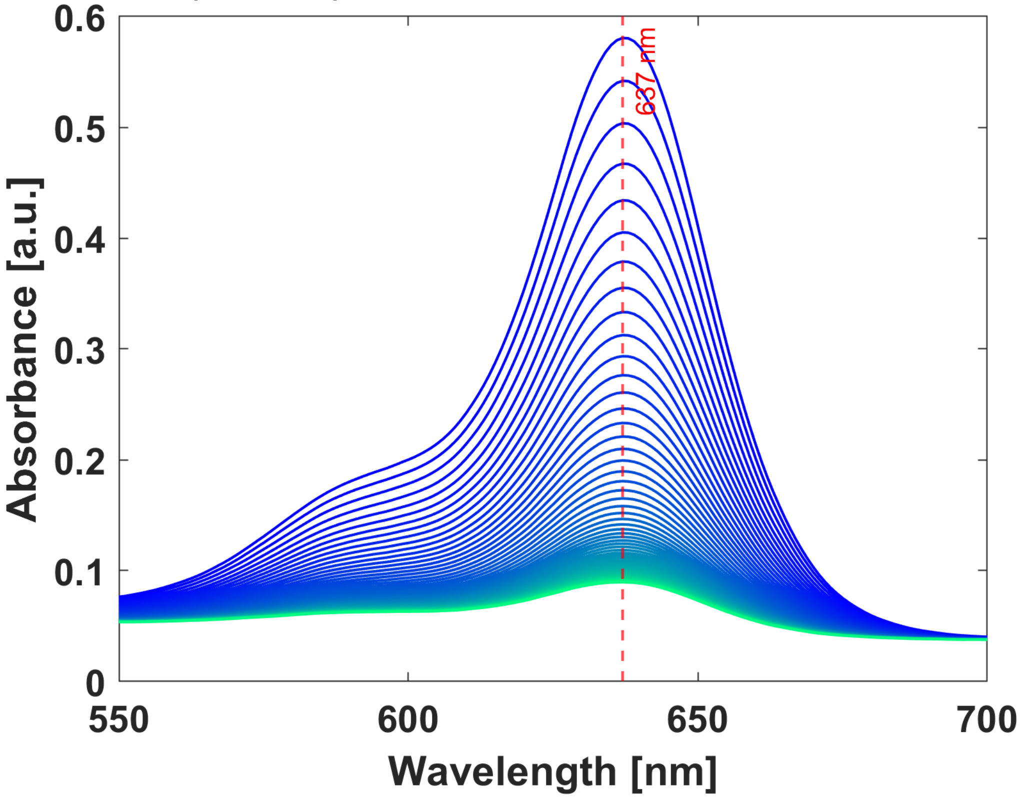

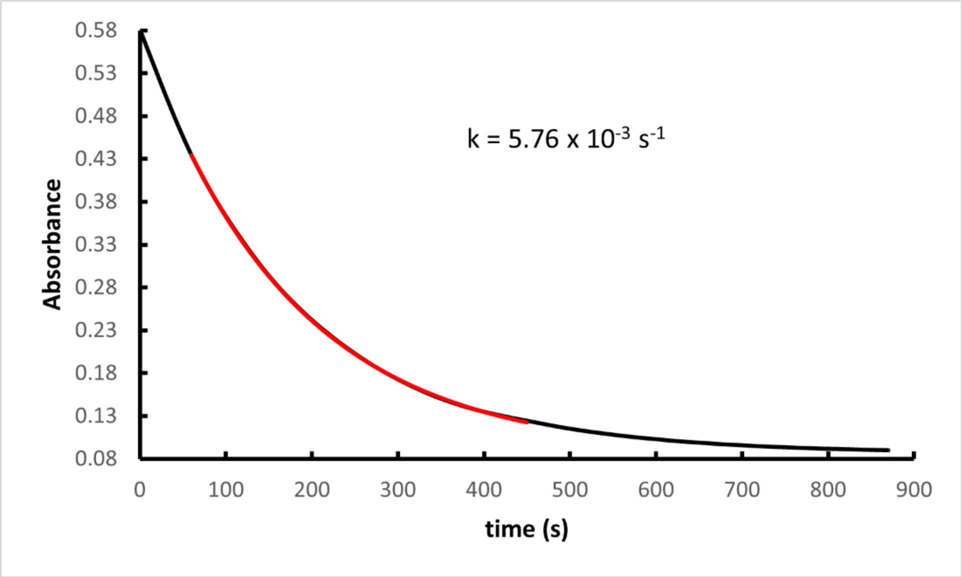

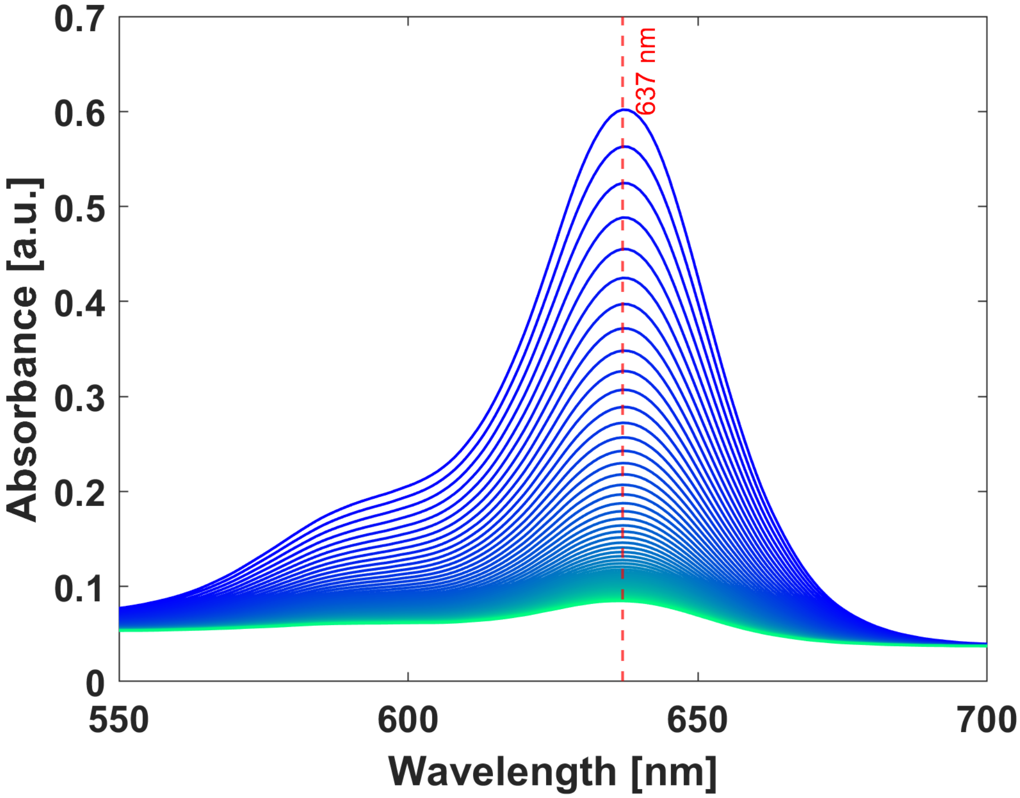
0.7
0.6
0.5
0.4
0.3
0.2
0.1
0
Absorbance [a.u.]
637 nm
550
600
650
700
Wavelength [nm]

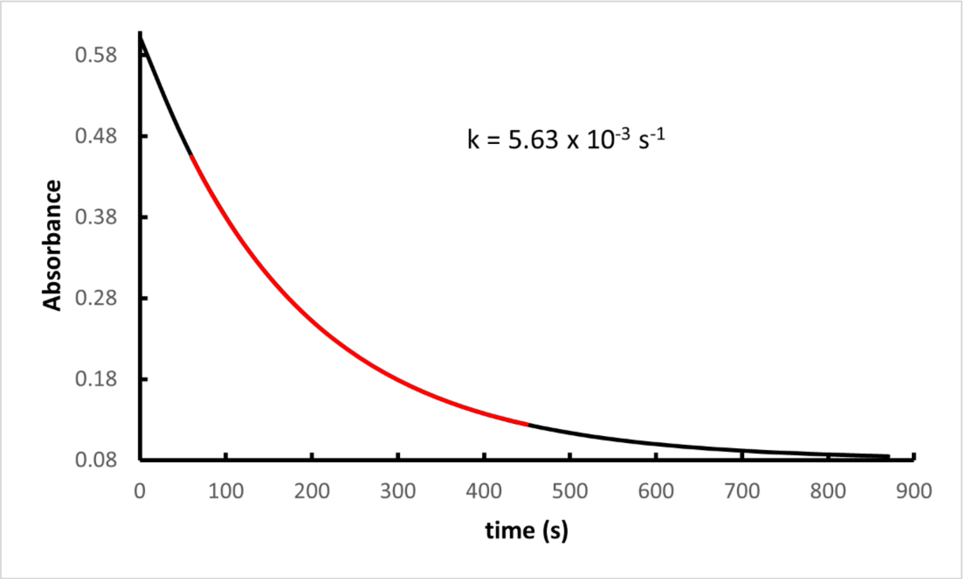
0.58
0.48
0.38
0.28
0.18
0.08
Absorbance
k = 5.63 x 10-3 s-1
0
100
200
300
400
500
600
700
800
900
time (s)

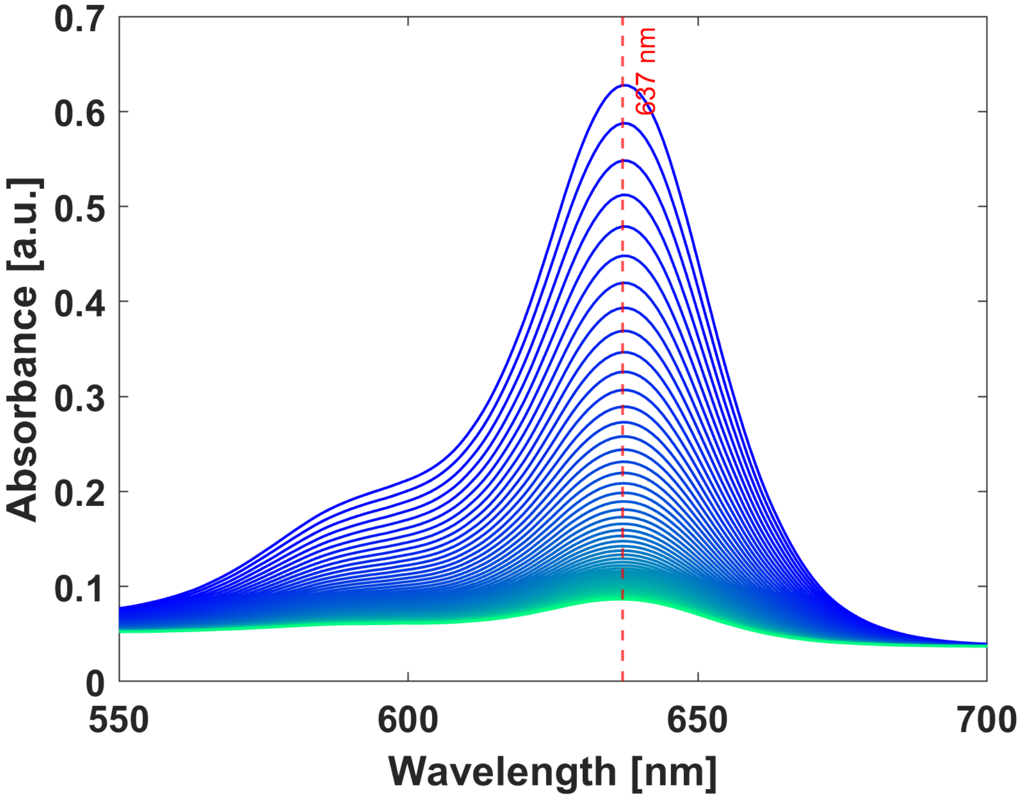

Absorbance [a.u.]
Wavelength [nm]
637 nm
0
0.1
0.2
0.3
0.4
0.5
0.6
0.7
550
600
650
700

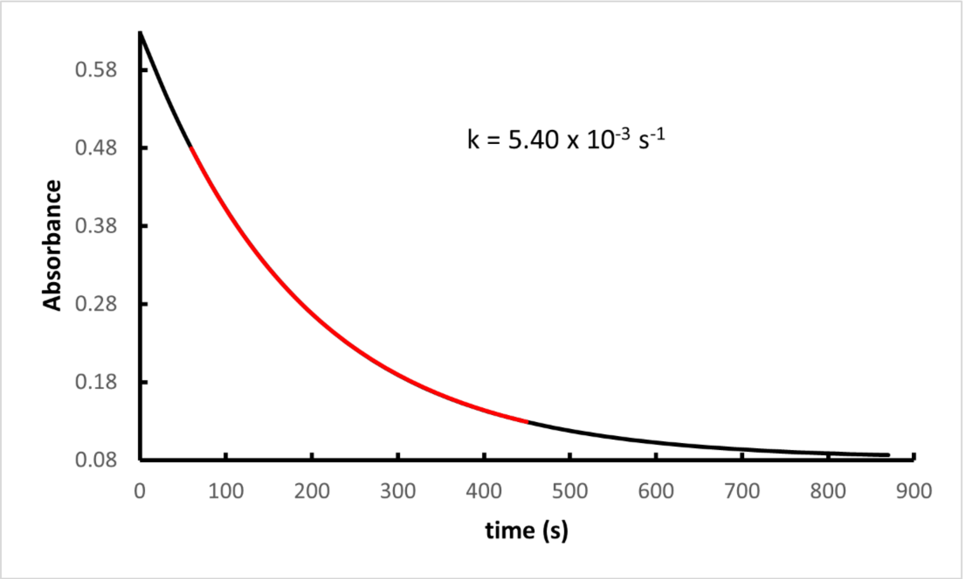

k = 5.40 x 10-3 s-1
Absorbance
time (s)
0.08
0.18
0.28
0.38
0.48
0.58
0
100
200
300
400
500
600
700
800
900

Results

Type	Rate (s^{-1})	Temperature	Type	Rate (s^{-1})	Temperature
C5	$5.35x10^{-3}$	22	cell	$5.76x10^{-3}$	22
C5	$6.06x10^{-3}$	22	cell	$5.63x10^{-3}$	22
C5	$5.57x10^{-3}$	22	cell	$5.40x10^{-3}$	22
Average:	**$5.66x10^{-3}$**	22	Average:	**$5.60x10^{-3}$**	22
Standard error:	**$2.09x10^{-4}$**		Standard error:	**$1.06x10^{-4}$**	

Reaction of *cyclo*-butanol (N7) with electrophile E1:
Cavity measurements (C4)

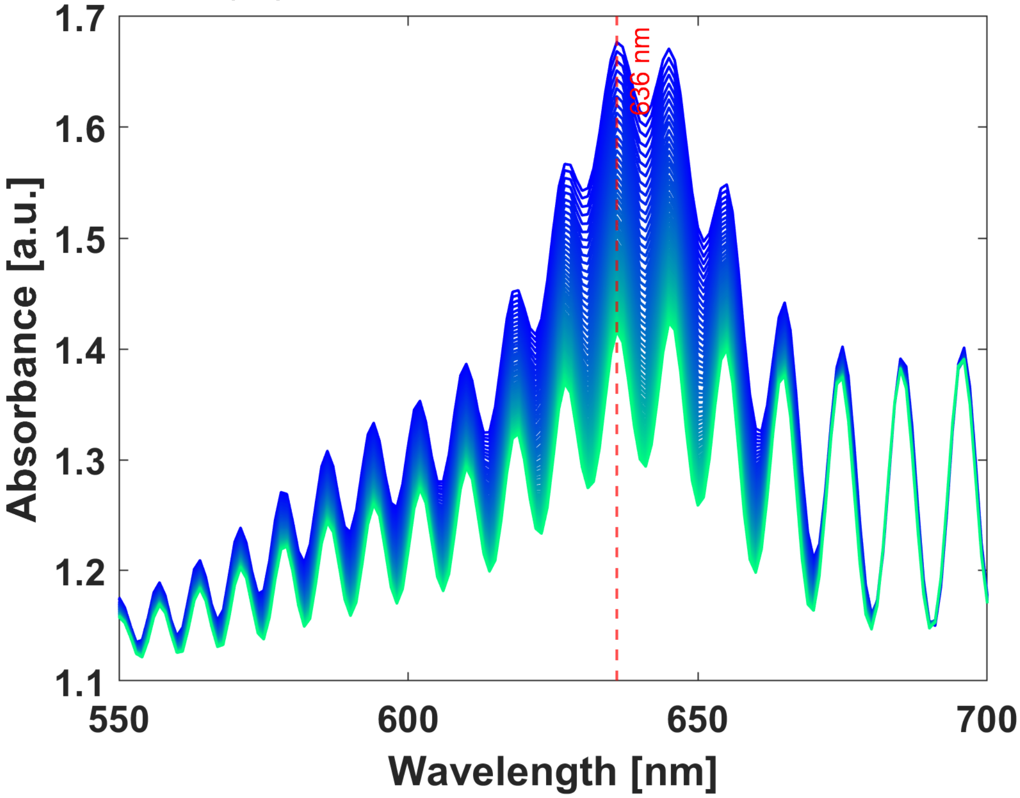

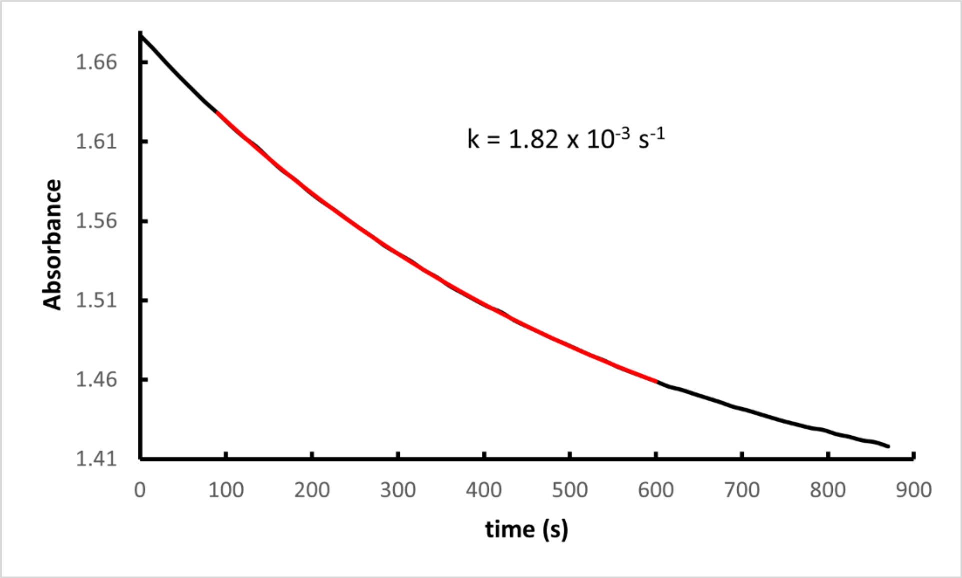

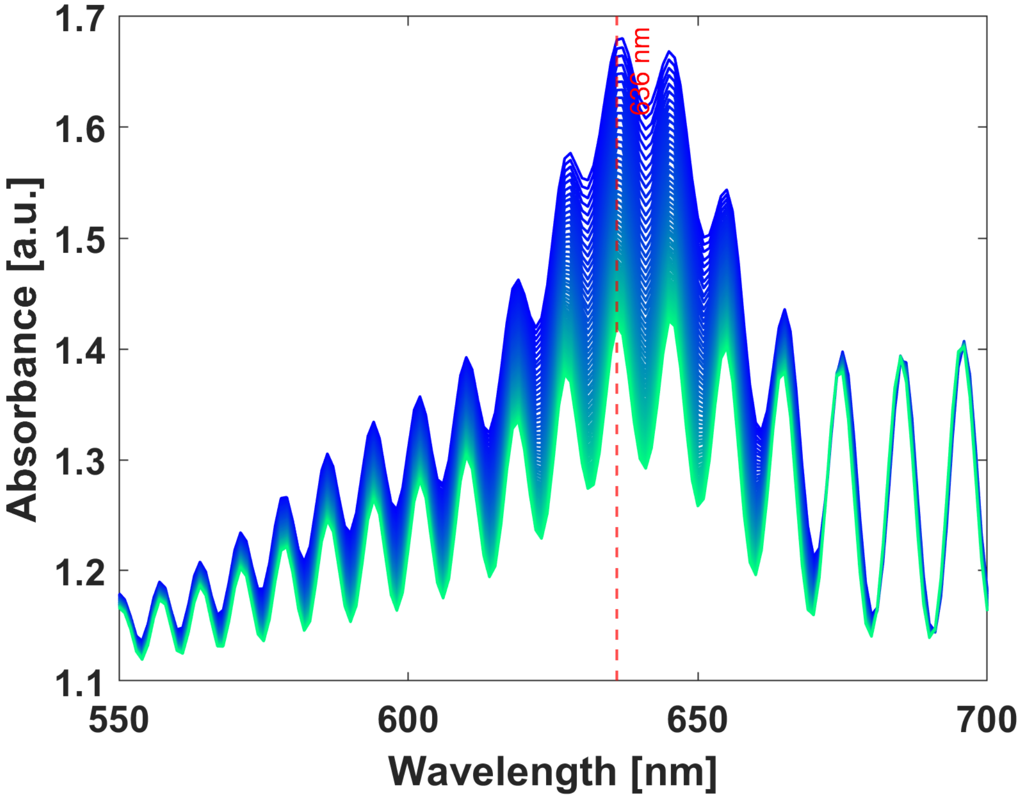
1.7
1.6
1.5
1.4
1.3
1.2
1.1
Absorbance [a.u.]
636 nm
550
600
650
700
Wavelength [nm]

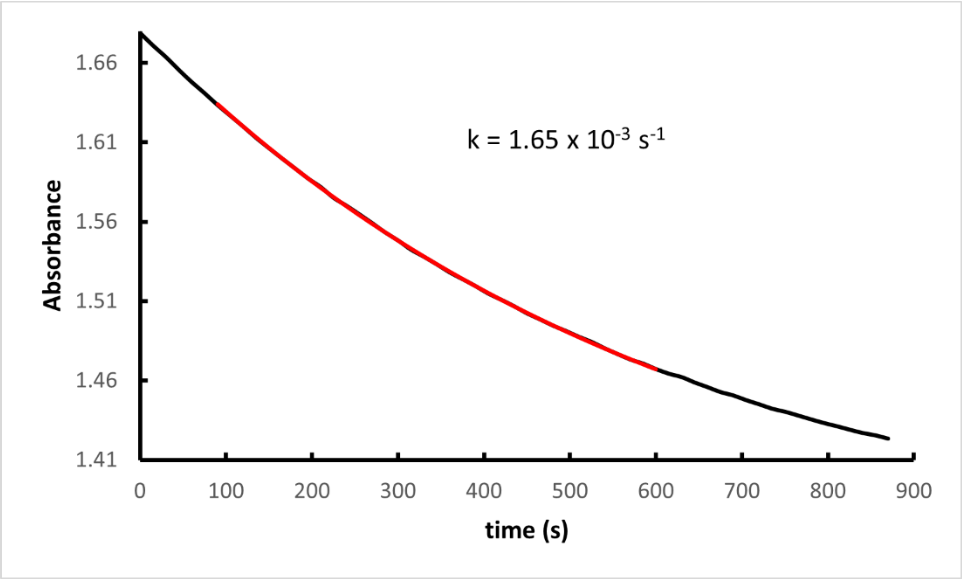
k = 1.65 x 10-3 s-1
Absorbance
1.66
1.61
1.56
1.51
1.46
1.41
0
100
200
300
400
500
600
700
800
900
time (s)

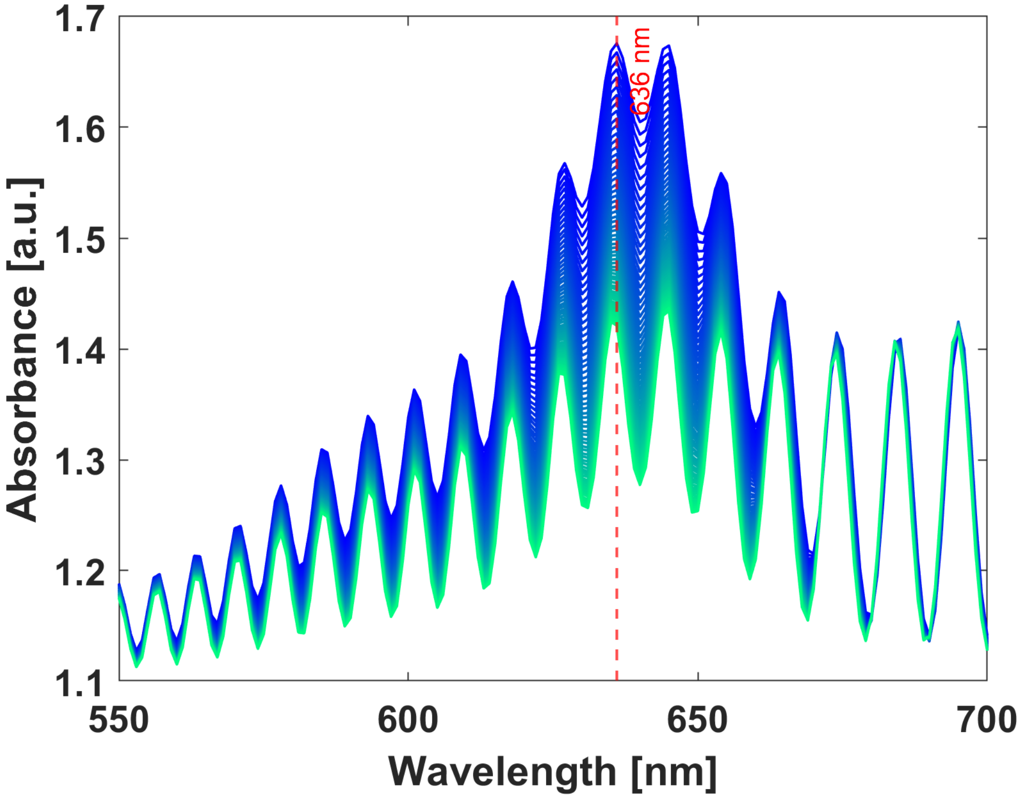
Absorbance [a.u.]
Wavelength [nm]
636 nm
1.7
1.6
1.5
1.4
1.3
1.2
1.1
550
600
650
700

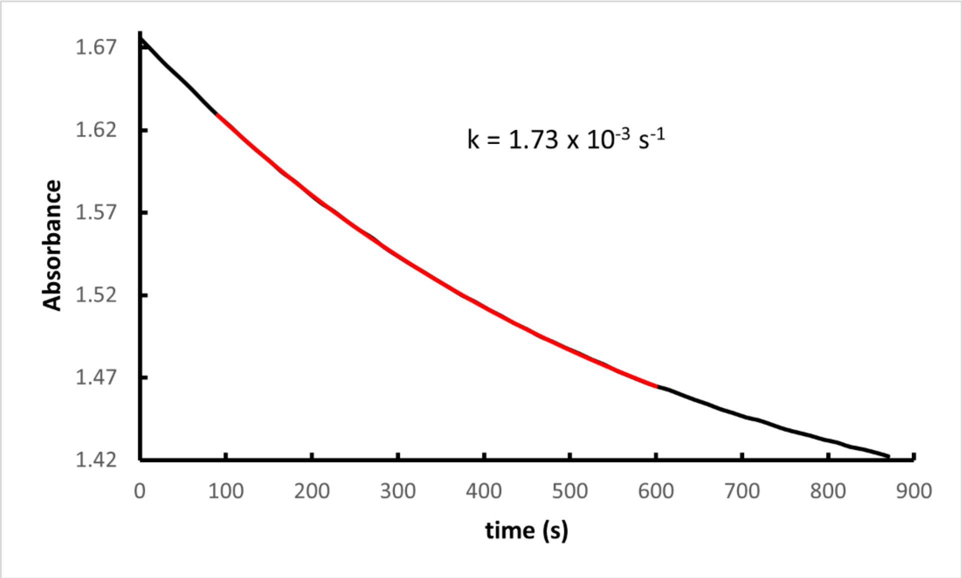
k = 1.73 x 10-3 s-1
Absorbance
time (s)
1.67
1.62
1.57
1.52
1.47
1.42
0
100
200
300
400
500
600
700
800
900

Cell measurements (standard)

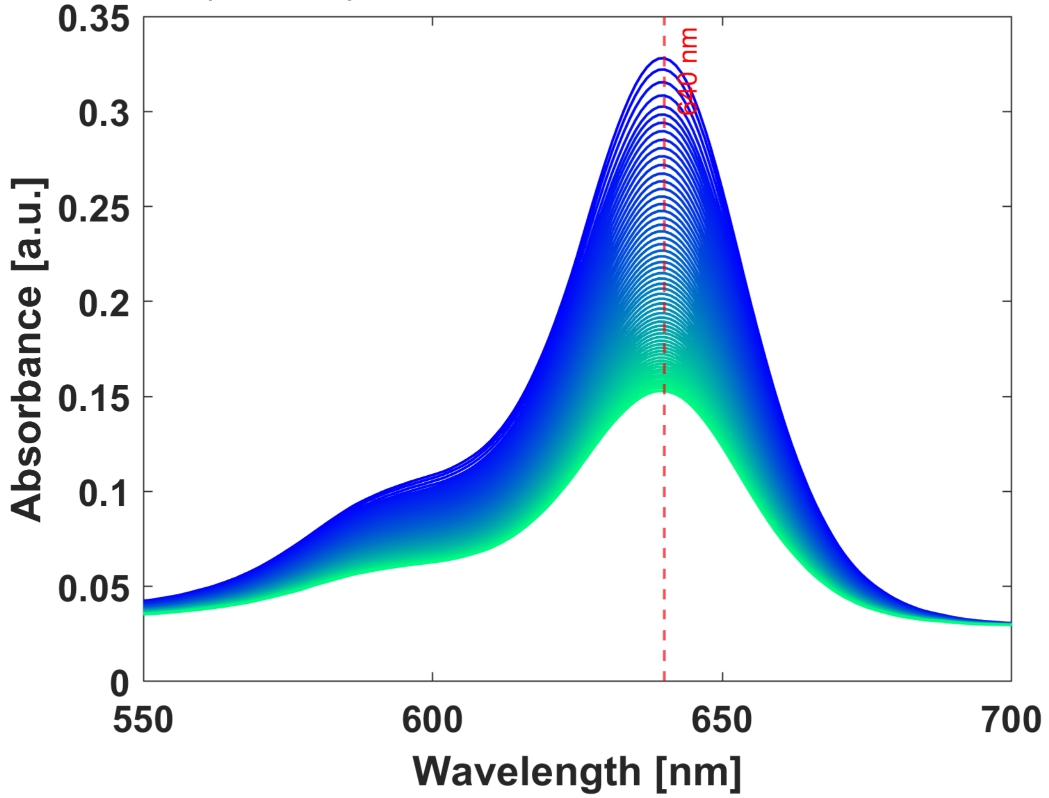

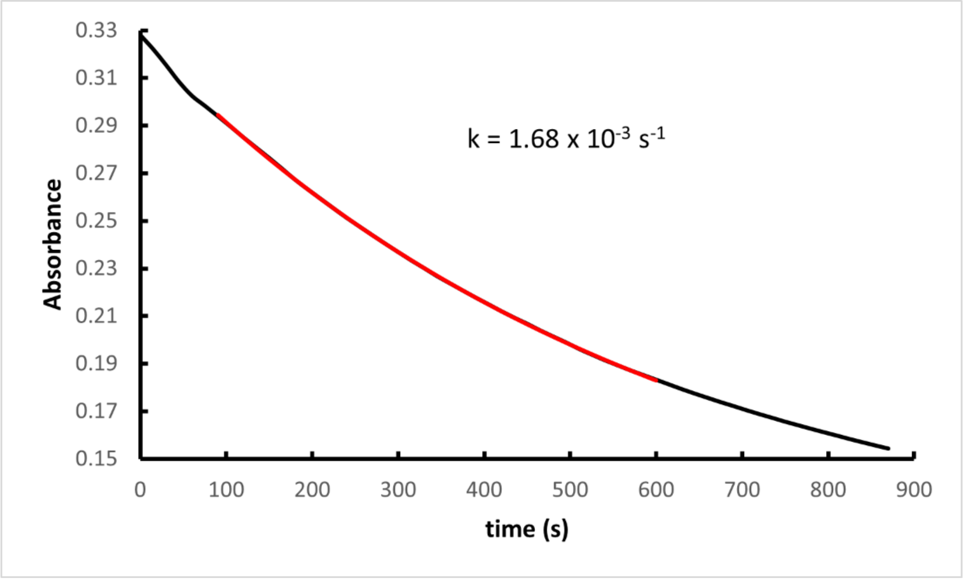

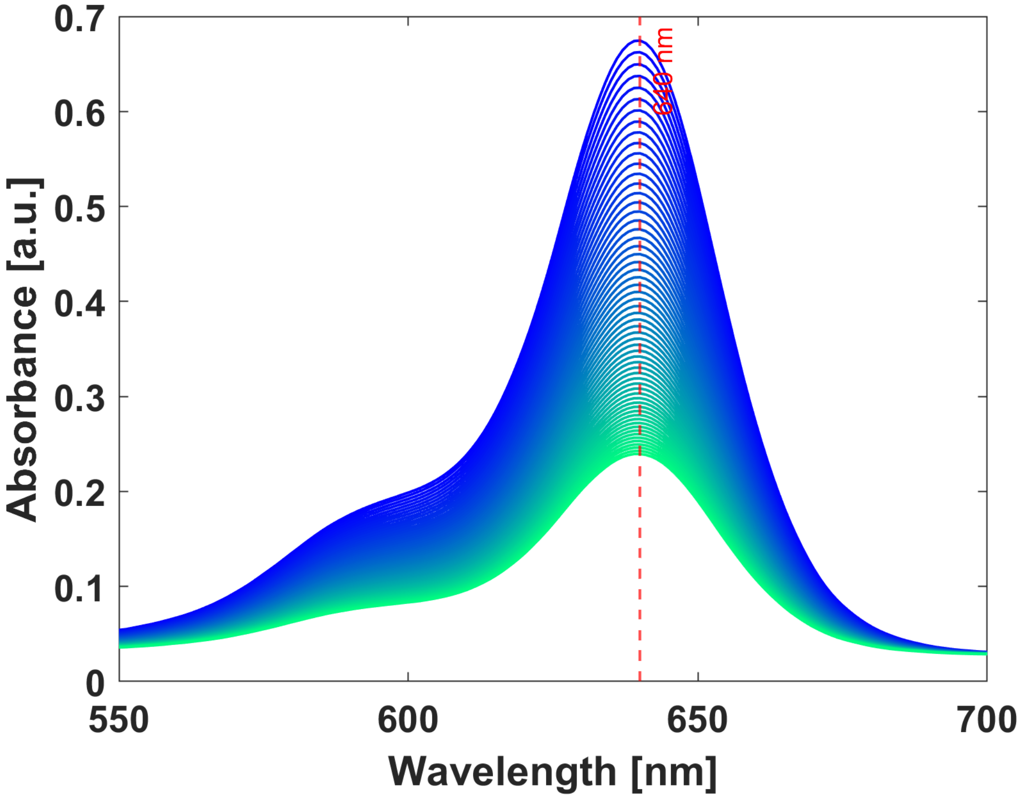

0.7
0.6
0.5
0.4
0.3
0.2
0.1
0
Absorbance [a.u.]
640 nm
550
600
650
700
Wavelength [nm]

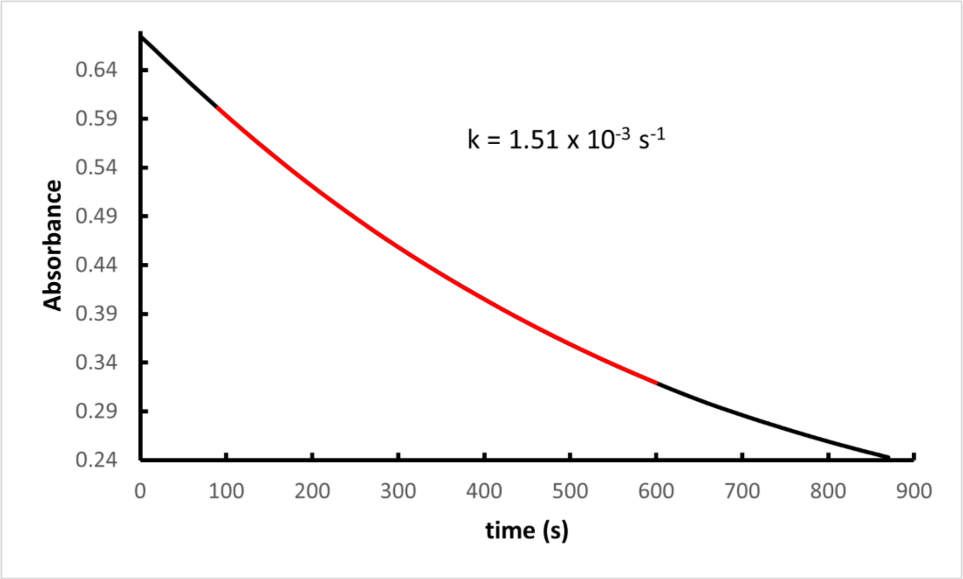

k = 1.51 x 10-3 s-1
Absorbance
0.64
0.59
0.54
0.49
0.44
0.39
0.34
0.29
0.24
0
100
200
300
400
500
600
700
800
900
time (s)

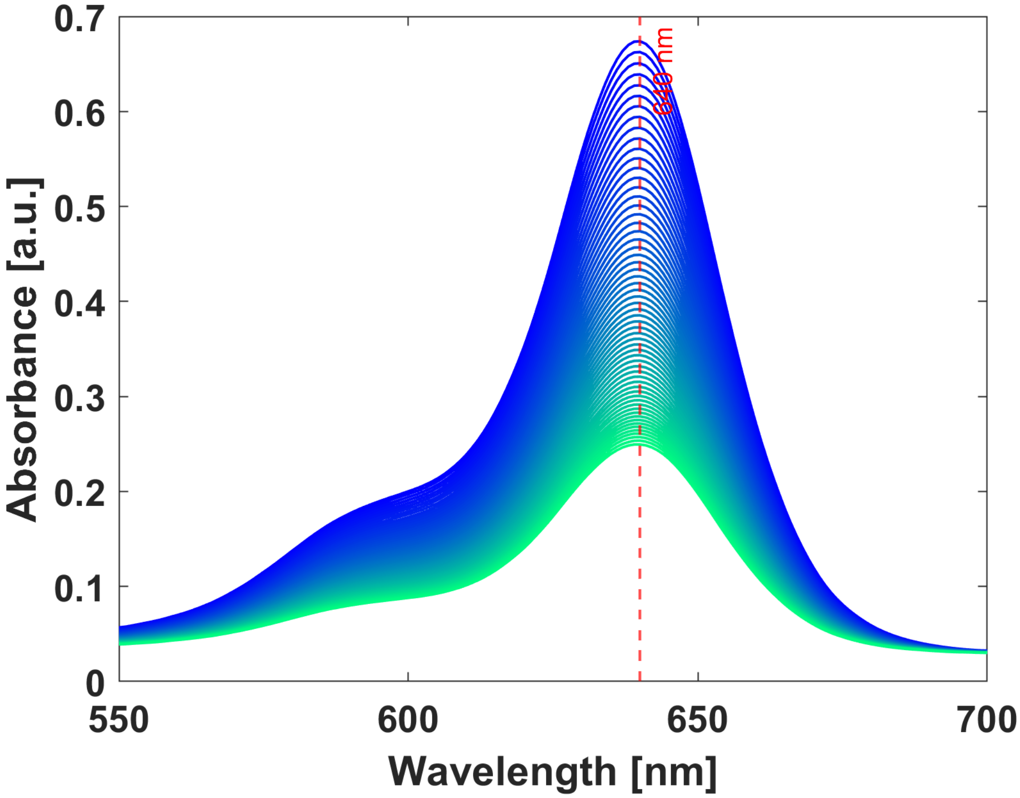

0.7
0.6
0.5
0.4
0.3
0.2
0.1
0
Absorbance [a.u.]
550
600
650
700
Wavelength [nm]
640 nm

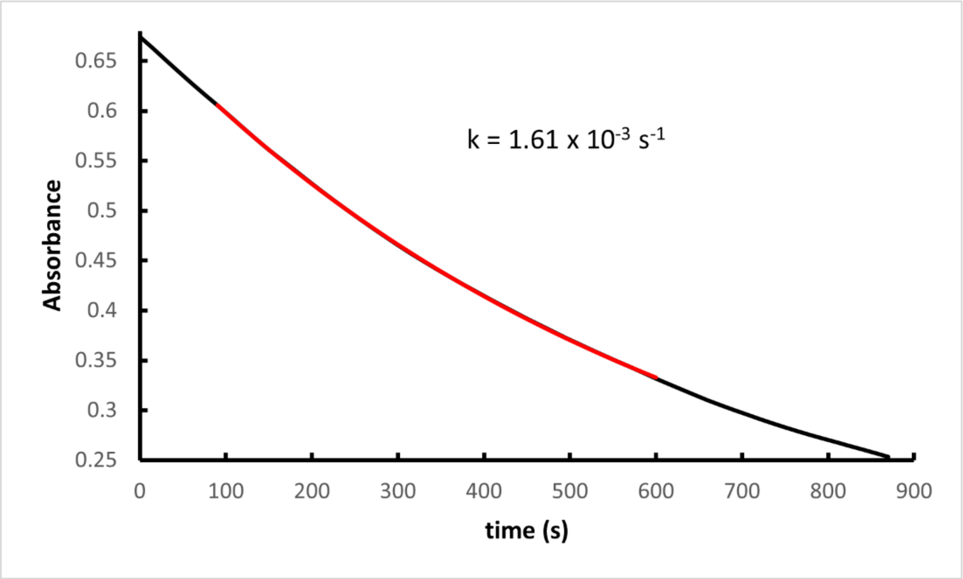

0.65
0.6
0.55
0.5
0.45
0.4
0.35
0.3
0.25
Absorbance
0
100
200
300
400
500
600
700
800
900
time (s)
k = 1.61 x 10-3 s-1

Results

Type	Rate (s^{-1})	Temperature	Type	Rate (s^{-1})	Temperature
cell	$1.68x10^{-3}$	19.6	C4	$1.82x10^{-3}$	19.7
cell	$1.51x10^{-3}$	19.7	C4	$1.65x10^{-3}$	19.7
cell	$1.61x10^{-3}$	19.7	C4	$1.73x10^{-3}$	19.8
Average:	**$1.60x10^{-3}$**	19.7	Average:	**$1.73x10^{-3}$**	19.7
Standard error:	**$4.95x10^{-5}$**		Standard error:	**$4.89x10^{-5}$**	

Reaction of *tert*-butanol (N8) with electrophile E4:

Cavity measurements (C1)

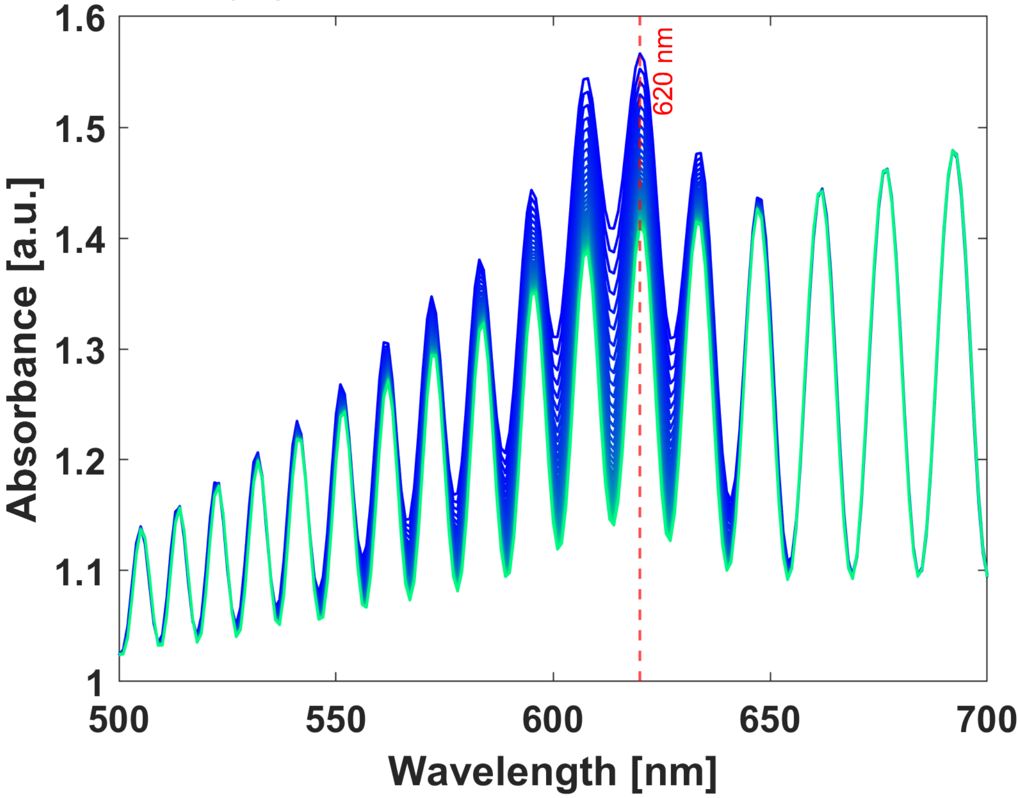

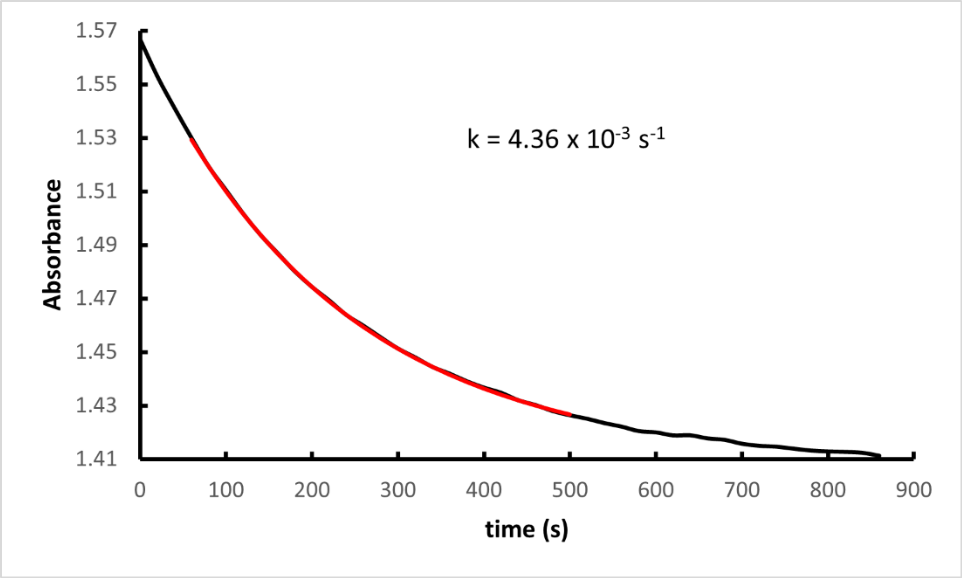

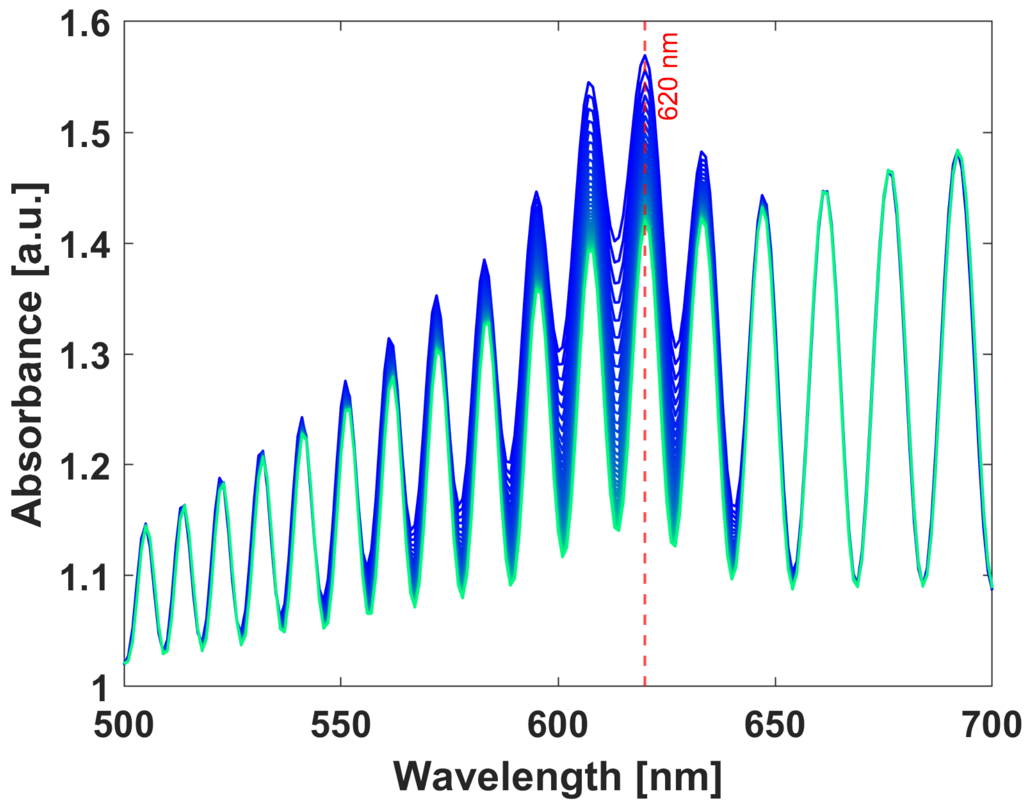
Absorbance [a.u.]
Wavelength [nm]
620 nm
1.6
1.5
1.4
1.3
1.2
1.1
1
500
550
600
650
700

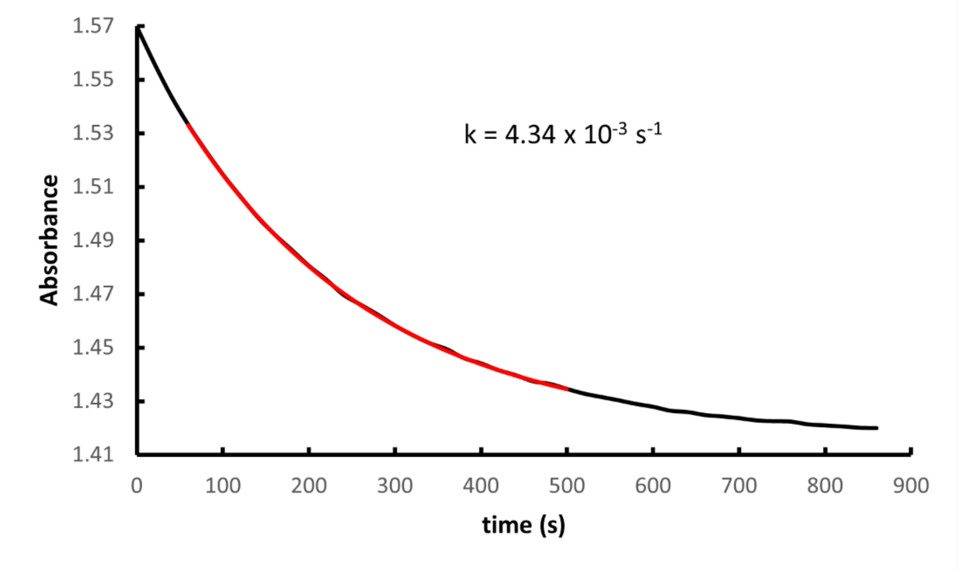
k = 4.34 x 10-3 s-1
Absorbance
time (s)
1.57
1.55
1.53
1.51
1.49
1.47
1.45
1.43
1.41
0
100
200
300
400
500
600
700
800
900

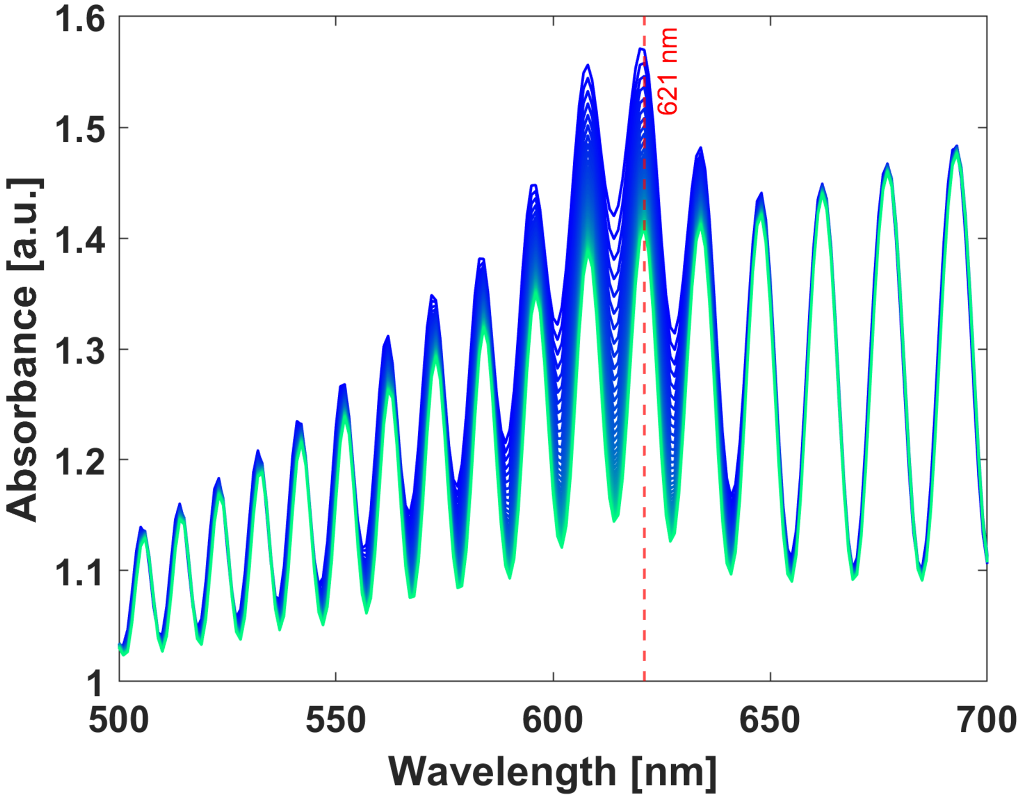

1.6
1.5
1.4
1.3
1.2
1.1
1
Absorbance [a.u.]
500
550
600
650
700
Wavelength [nm]
621 nm

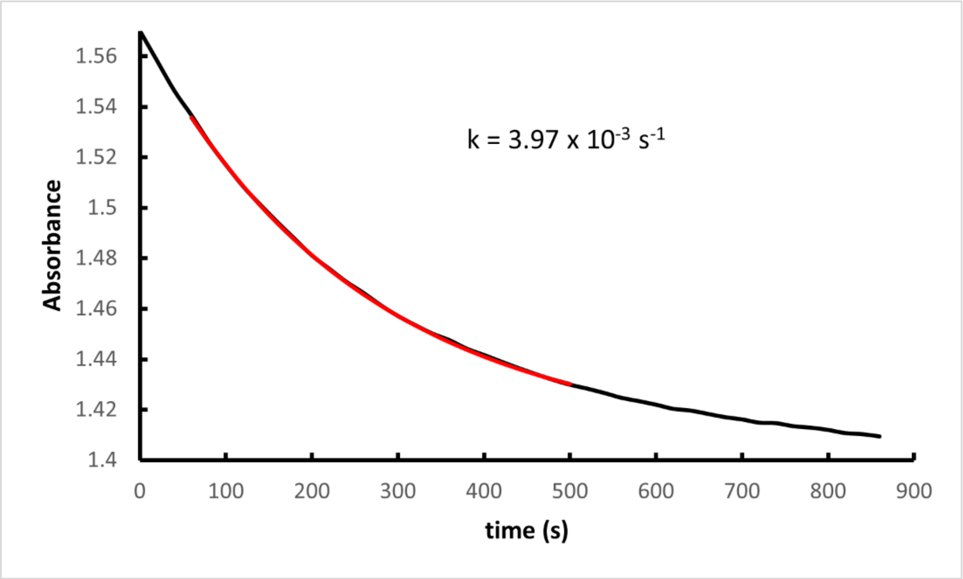

k = 3.97 x 10-3 s-1
Absorbance
1.56
1.54
1.52
1.5
1.48
1.46
1.44
1.42
1.4
0
100
200
300
400
500
600
700
800
900
time (s)

Cavity measurements (C2)

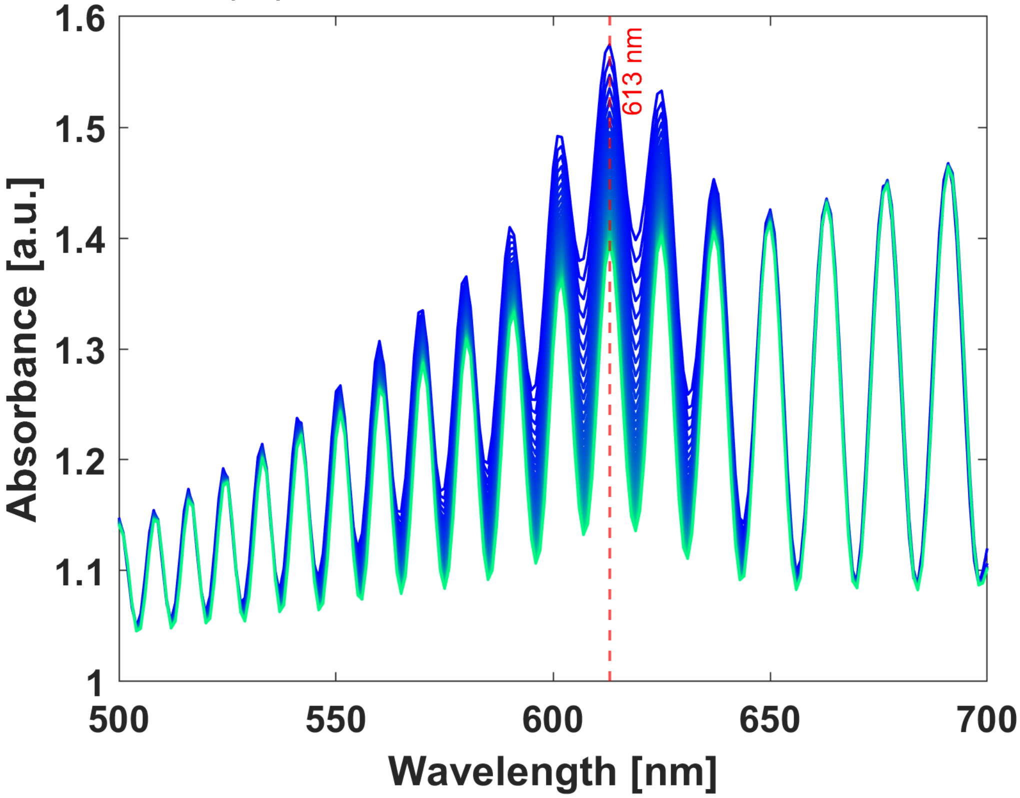

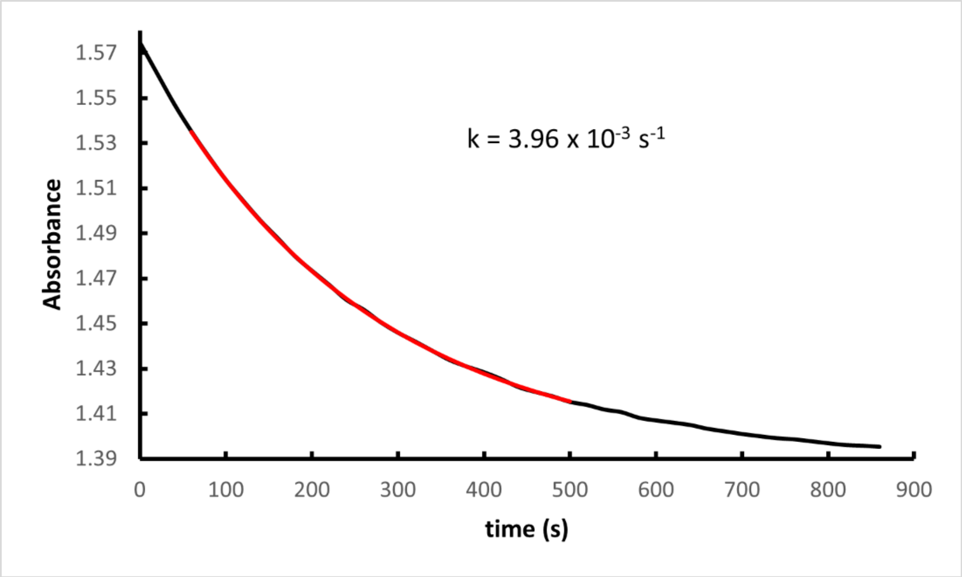

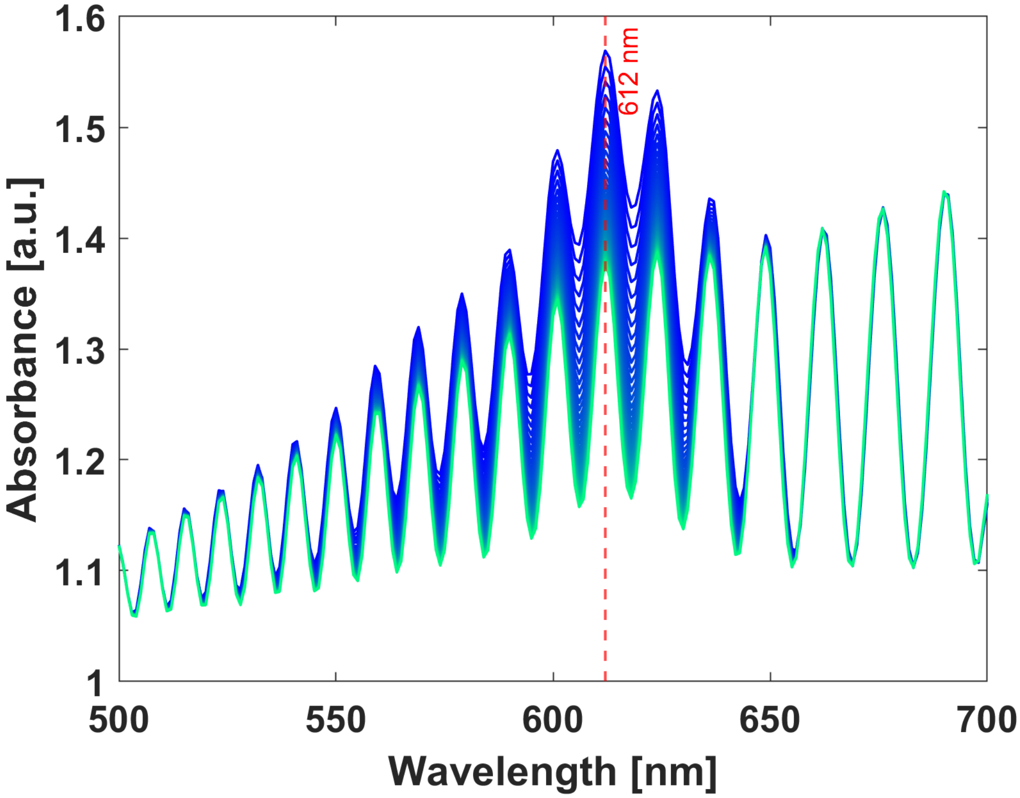

612 nm
Absorbance [a.u.]
Wavelength [nm]
1.6
1.5
1.4
1.3
1.2
1.1
1
500
550
600
650
700

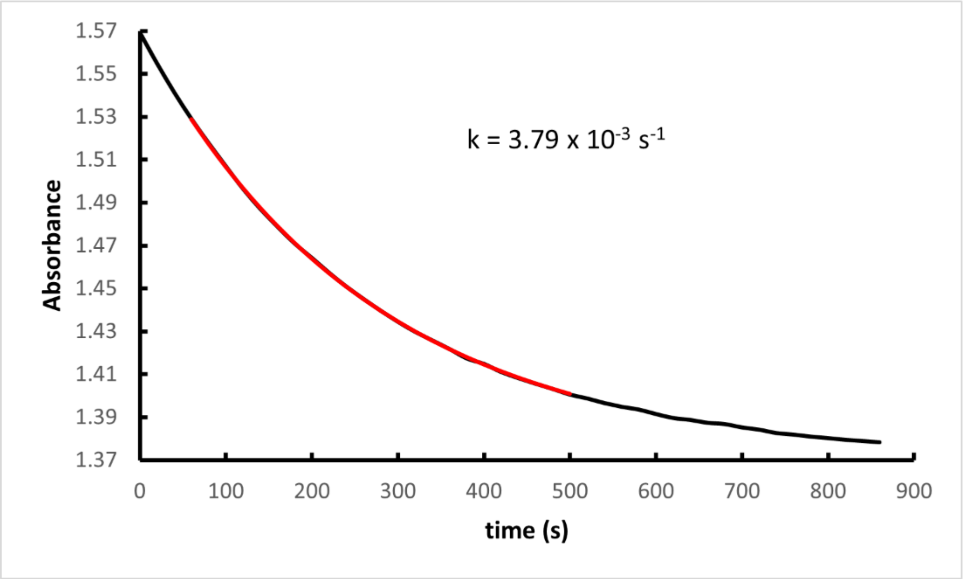

k = 3.79 x 10-3 s-1
Absorbance
time (s)
1.57
1.55
1.53
1.51
1.49
1.47
1.45
1.43
1.41
1.39
1.37
0
100
200
300
400
500
600
700
800
900

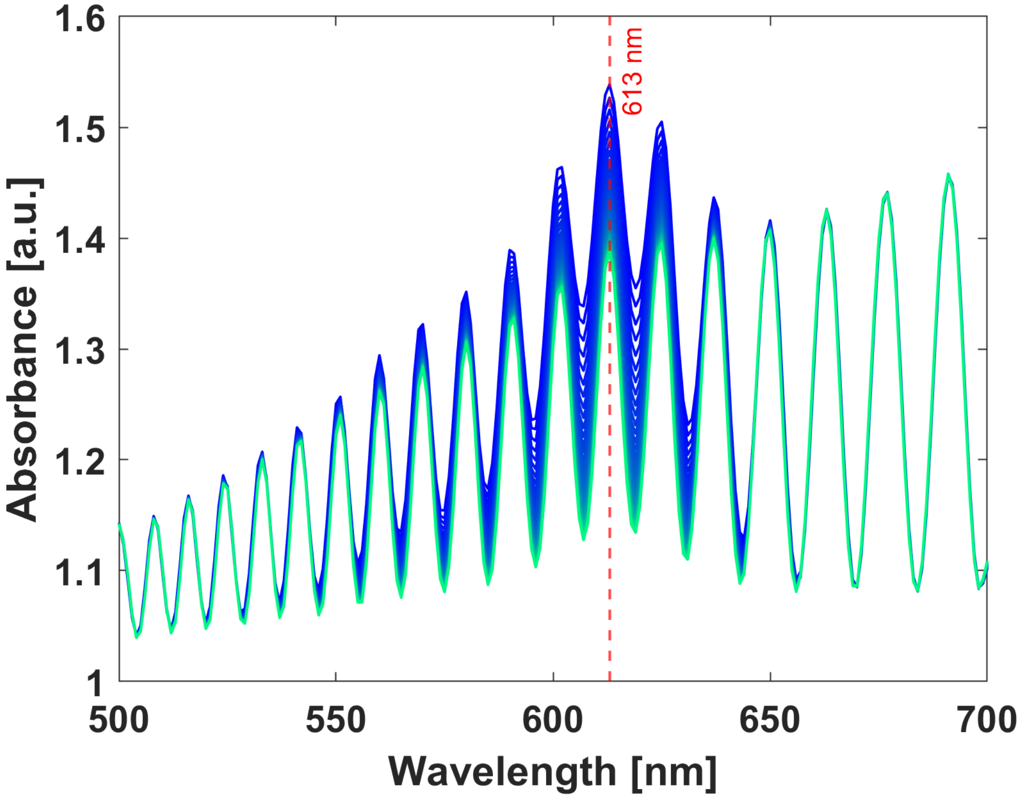

1.6
1.5
1.4
1.3
1.2
1.1
1
500
550
600
650
700
Absorbance [a.u.]
Wavelength [nm]
613 nm

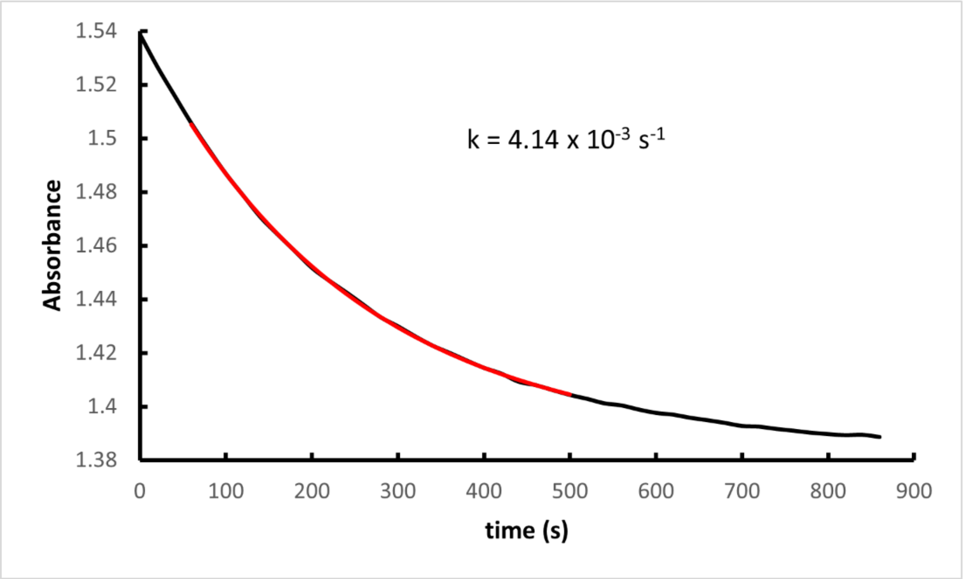

k = 4.14 x 10-3 s-1
Absorbance
time (s)
1.54
1.52
1.5
1.48
1.46
1.44
1.42
1.4
1.38
0
100
200
300
400
500
600
700
800
900

Cavity measurements (C3)

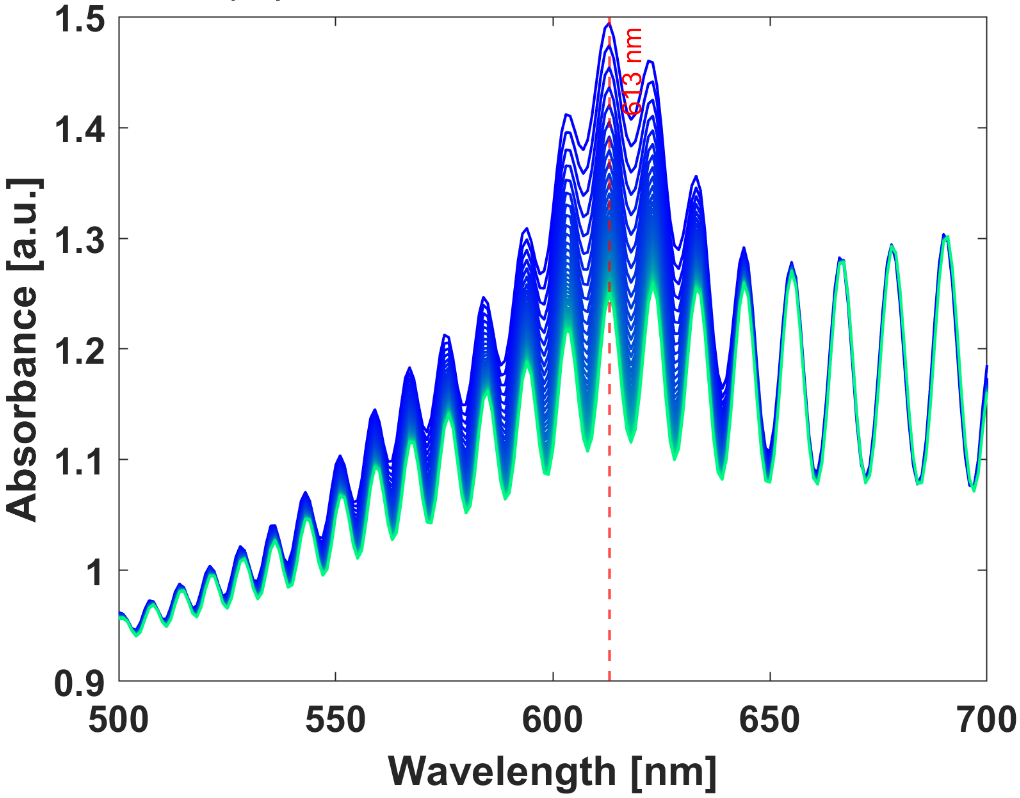

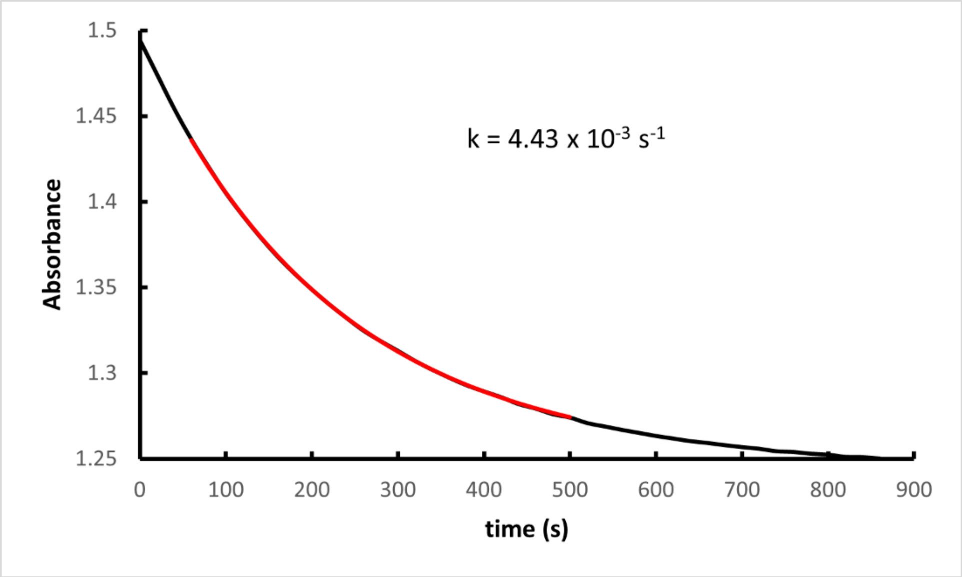

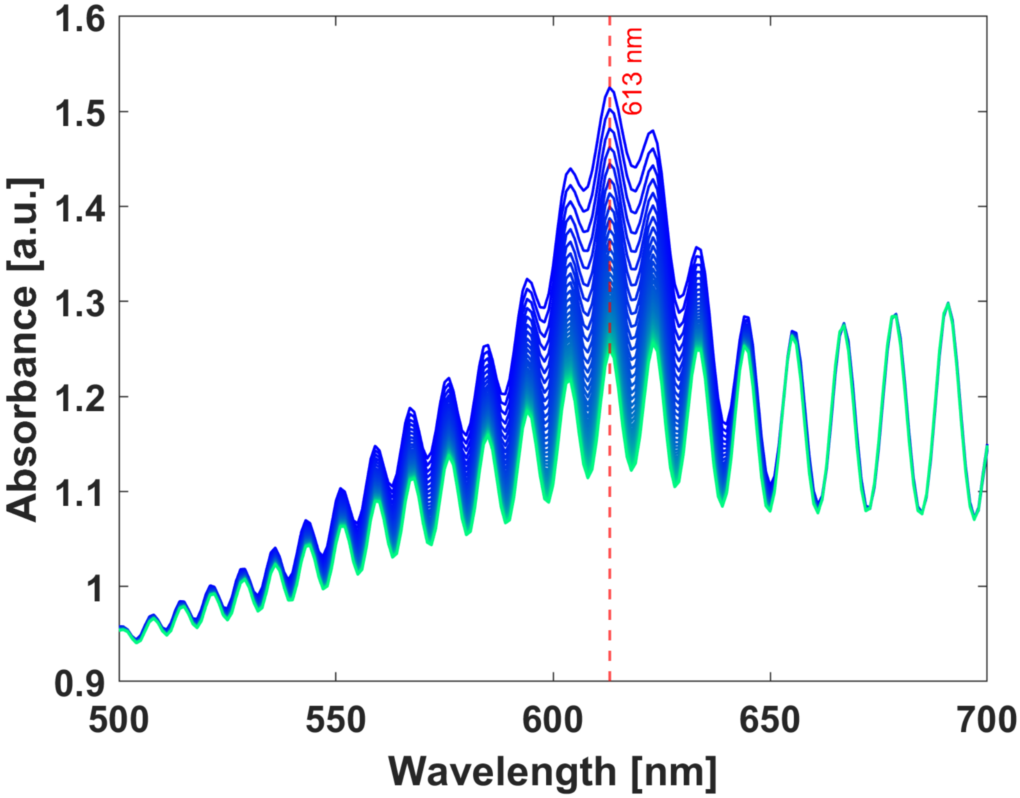

1.6
1.5
1.4
1.3
1.2
1.1
1
0.9
Absorbance [a.u.]
613 nm
500
550
600
650
700
Wavelength [nm]

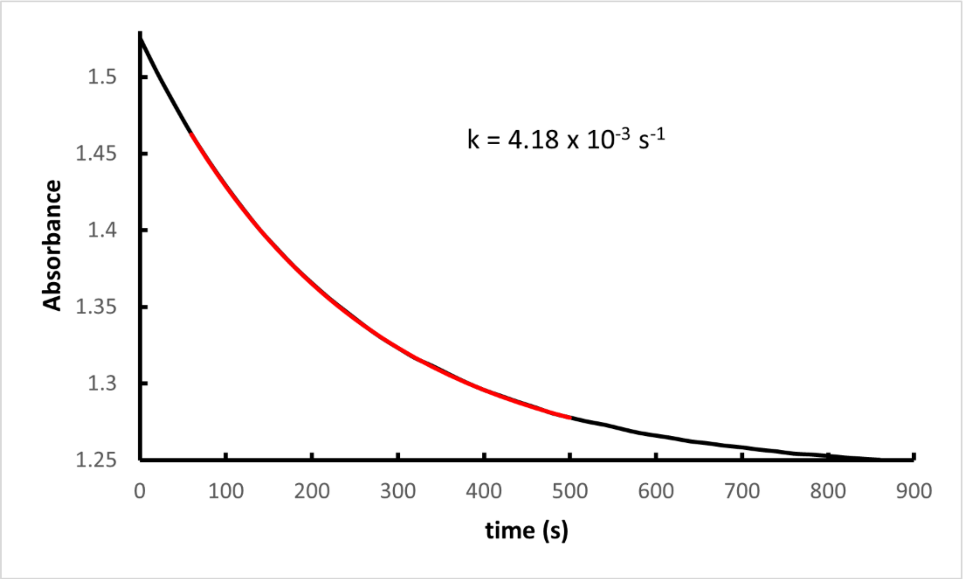

1.5
1.45
1.4
1.35
1.3
1.25
Absorbance
k = 4.18 x 10-3 s-1
0
100
200
300
400
500
600
700
800
900
time (s)

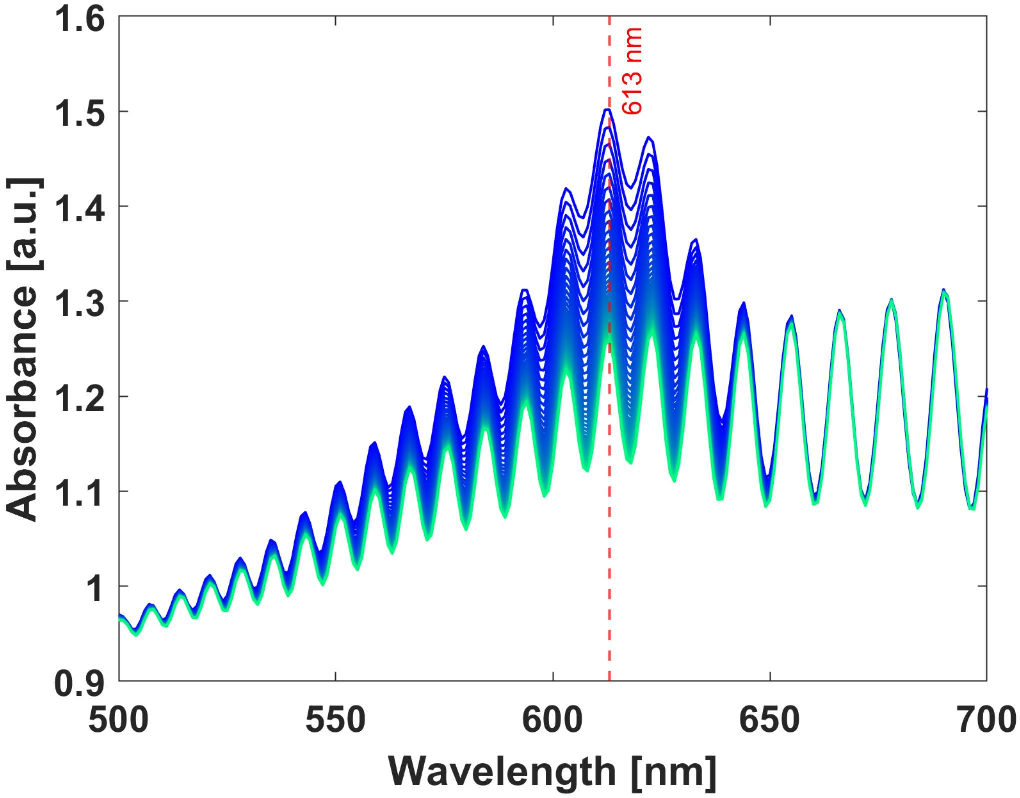

613 nm
Absorbance [a.u.]
Wavelength [nm]
1.6
1.5
1.4
1.3
1.2
1.1
1
0.9
500
550
600
650
700

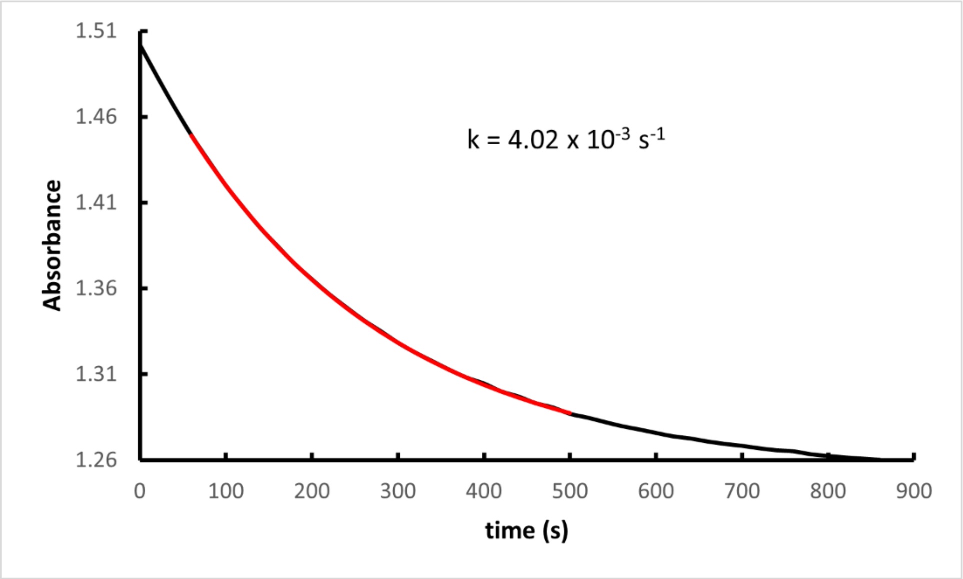

k = 4.02 x 10^{-3} s^{-1}
Absorbance
time (s)
1.51
1.46
1.41
1.36
1.31
1.26
0
100
200
300
400
500
600
700
800
900

Cavity measurements (C5)

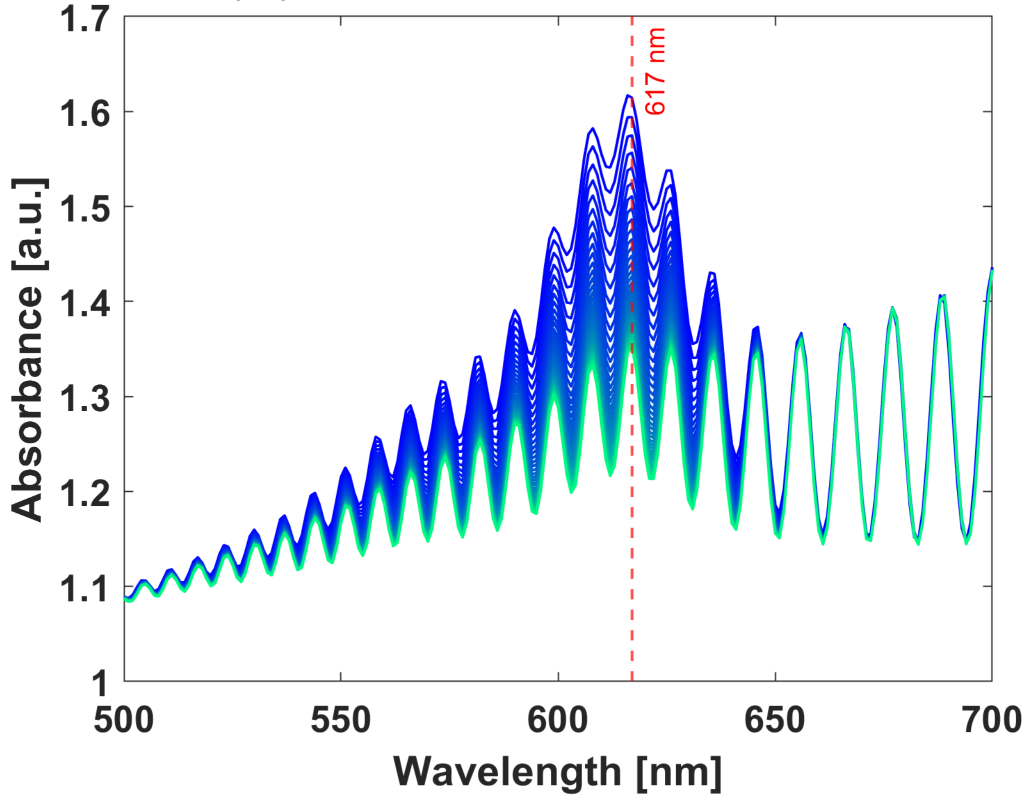

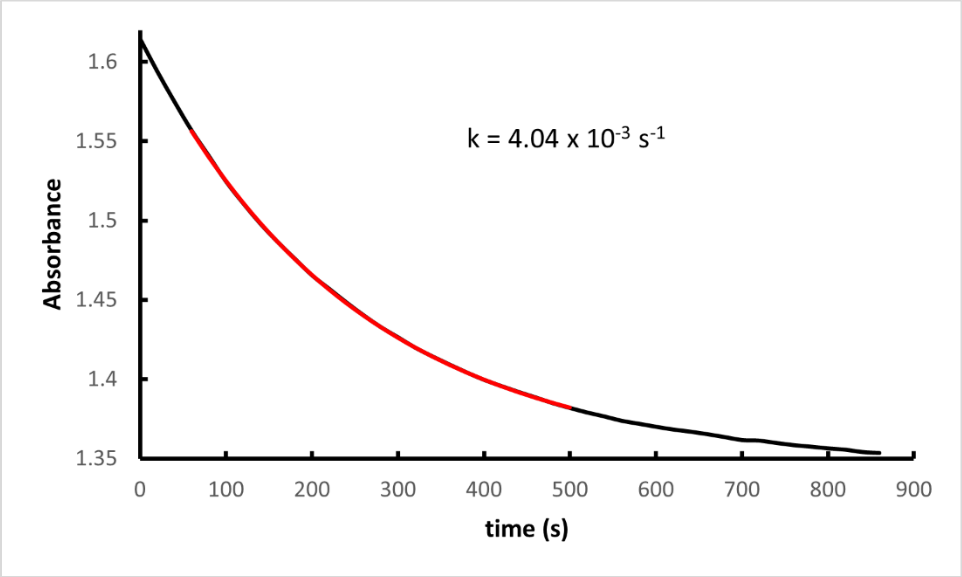

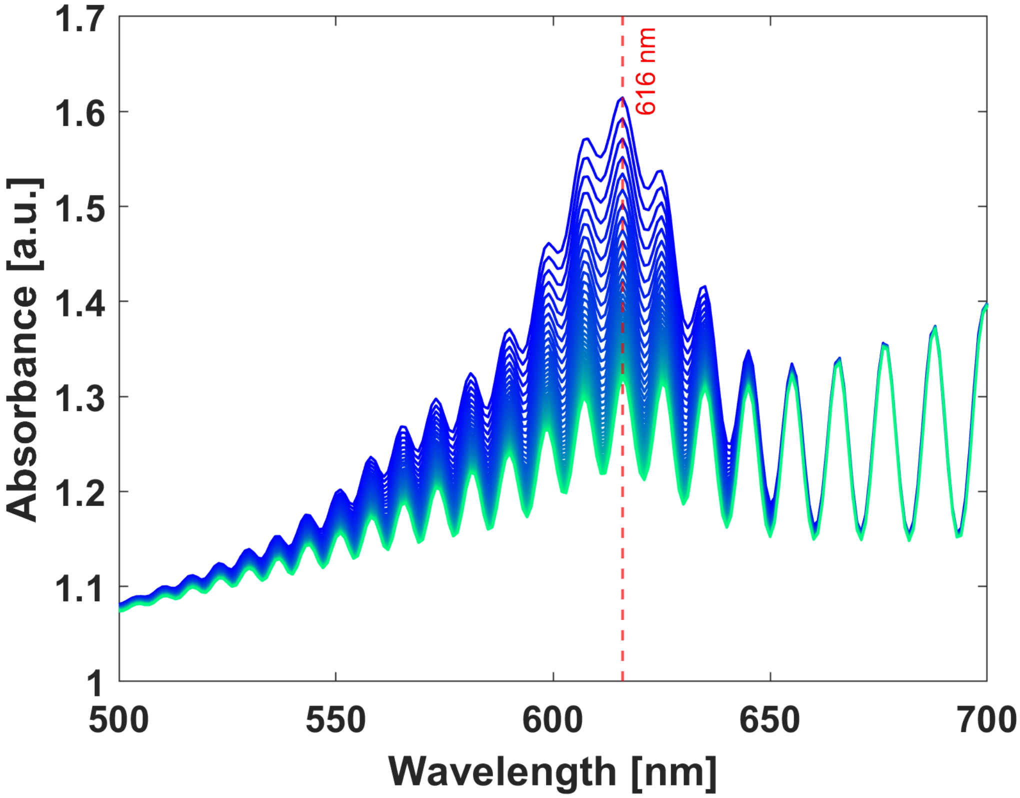

1.7
1.6
1.5
1.4
1.3
1.2
1.1
1
500
550
600
650
700
Absorbance [a.u.]
Wavelength [nm]
616 nm

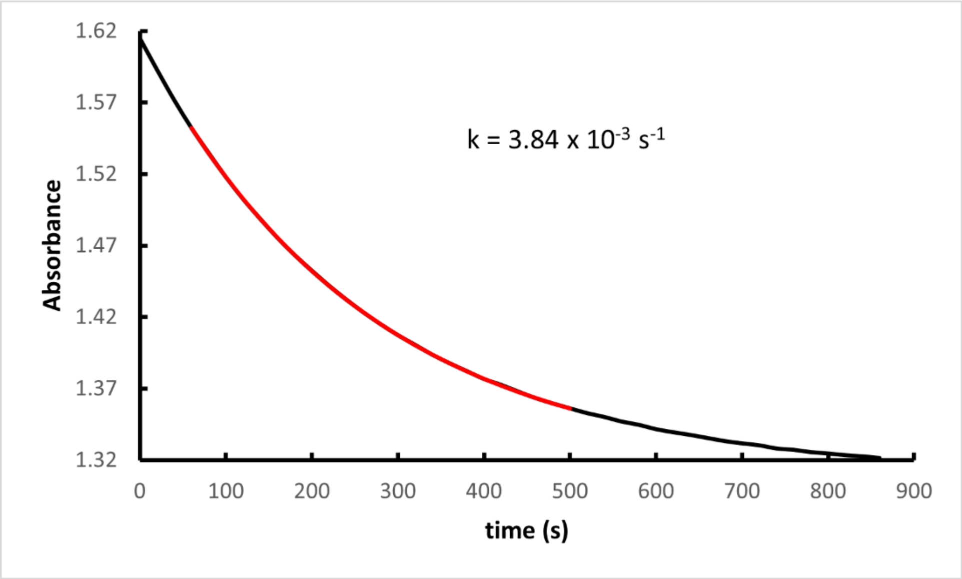

1.62
1.57
1.52
1.47
1.42
1.37
1.32
0
100
200
300
400
500
600
700
800
900
Absorbance
time (s)
k = 3.84 x 10-3 s-1

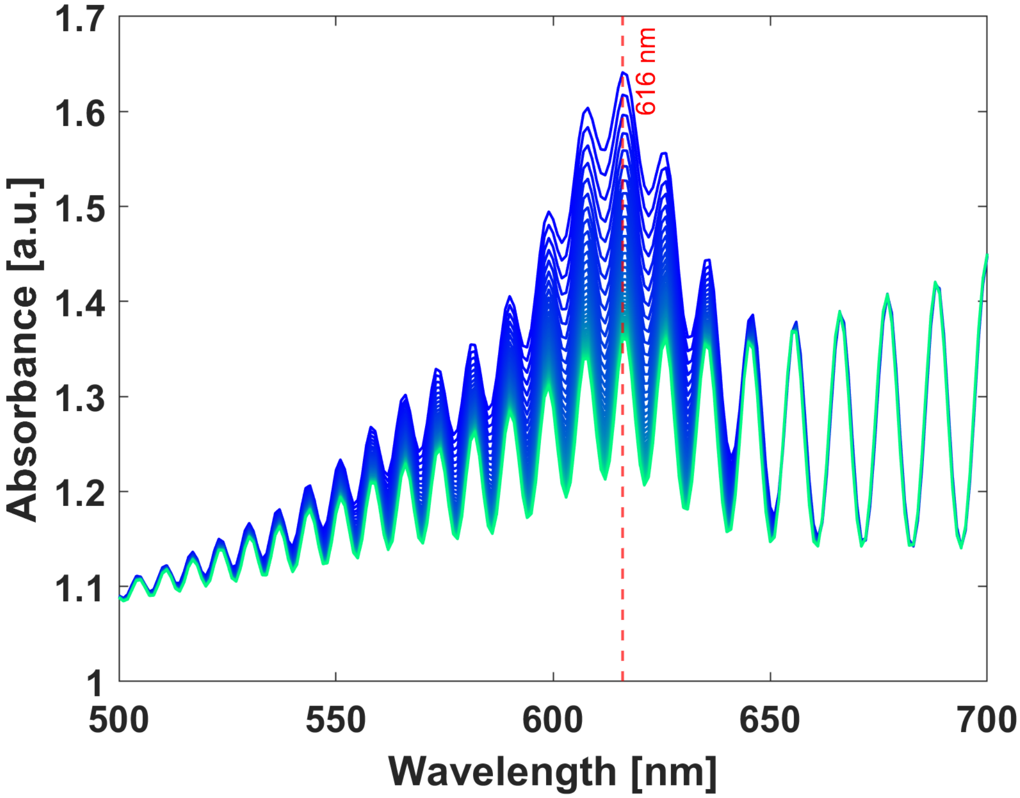

616 nm
Absorbance [a.u.]
Wavelength [nm]

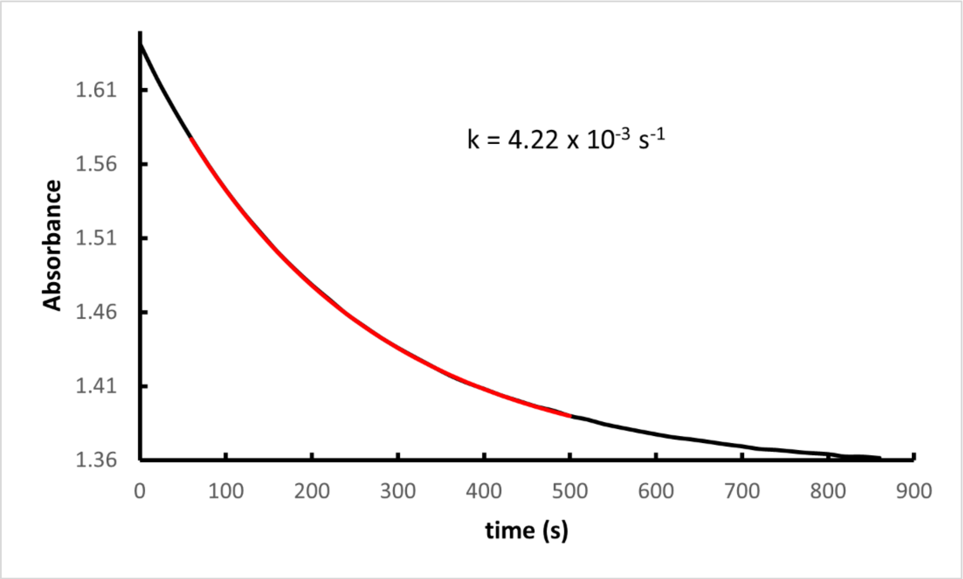

k = 4.22 x 10-3 s-1
Absorbance
time (s)

Cell measurements (standard)

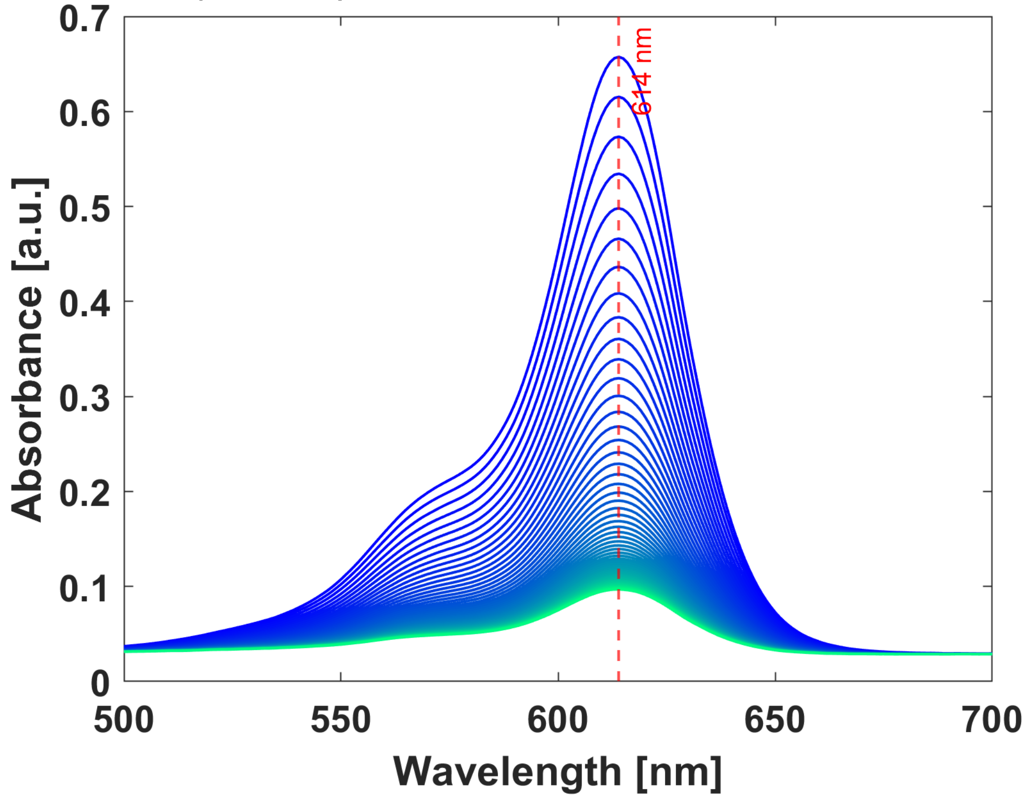

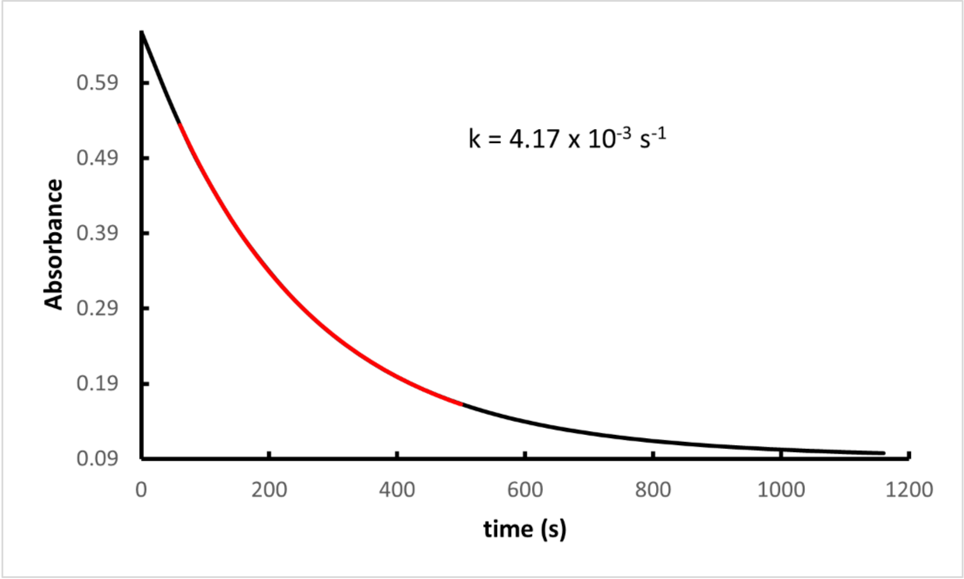

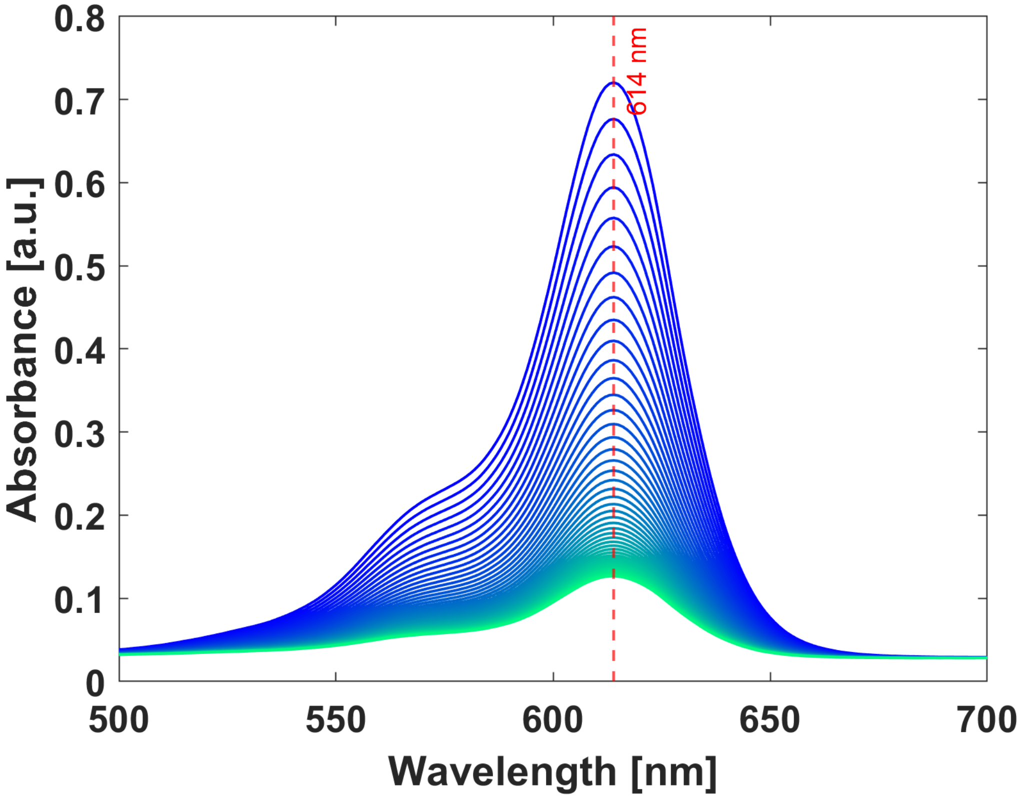
Absorbance [a.u.]
Wavelength [nm]
614 nm
0
0.1
0.2
0.3
0.4
0.5
0.6
0.7
0.8
500
550
600
650
700

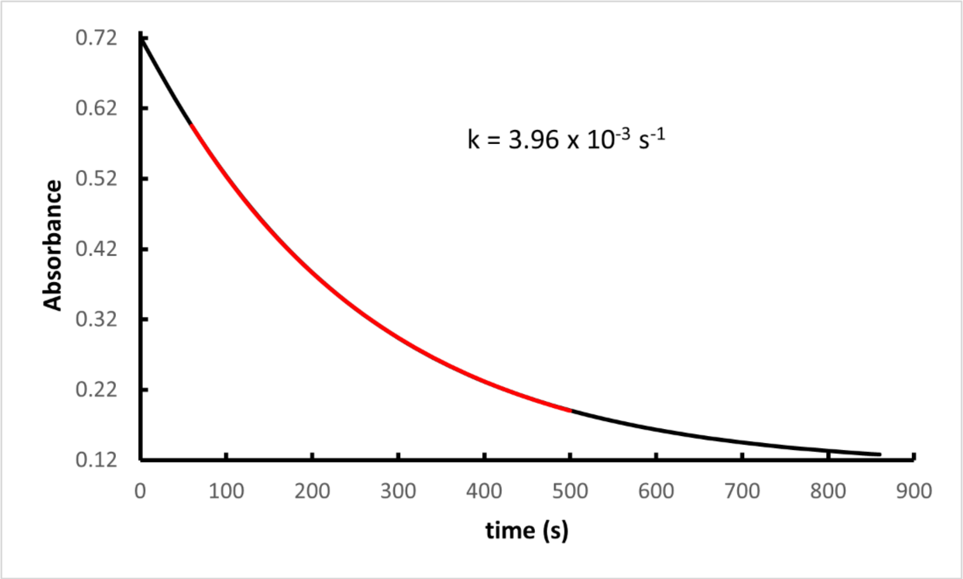
Absorbance
time (s)
k = 3.96 x 10-3 s-1
0.12
0.22
0.32
0.42
0.52
0.62
0.72
0
100
200
300
400
500
600
700
800
900

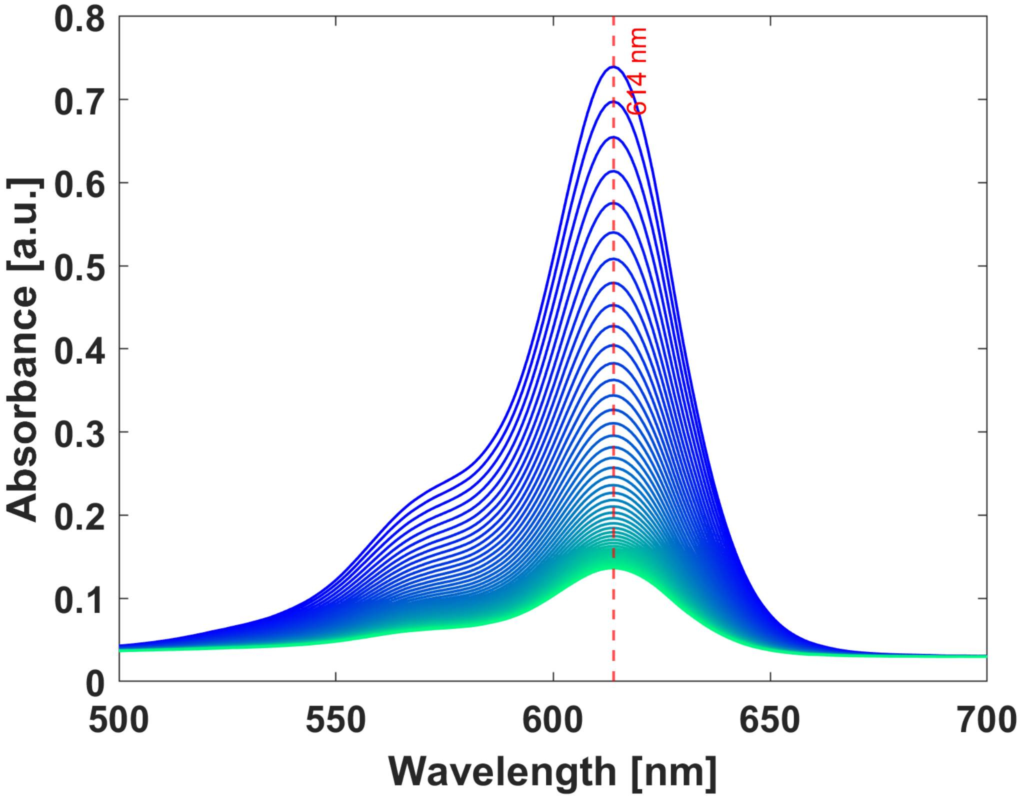

Absorbance [a.u.]
Wavelength [nm]
614 nm
0
0.1
0.2
0.3
0.4
0.5
0.6
0.7
0.8
500
550
600
650
700

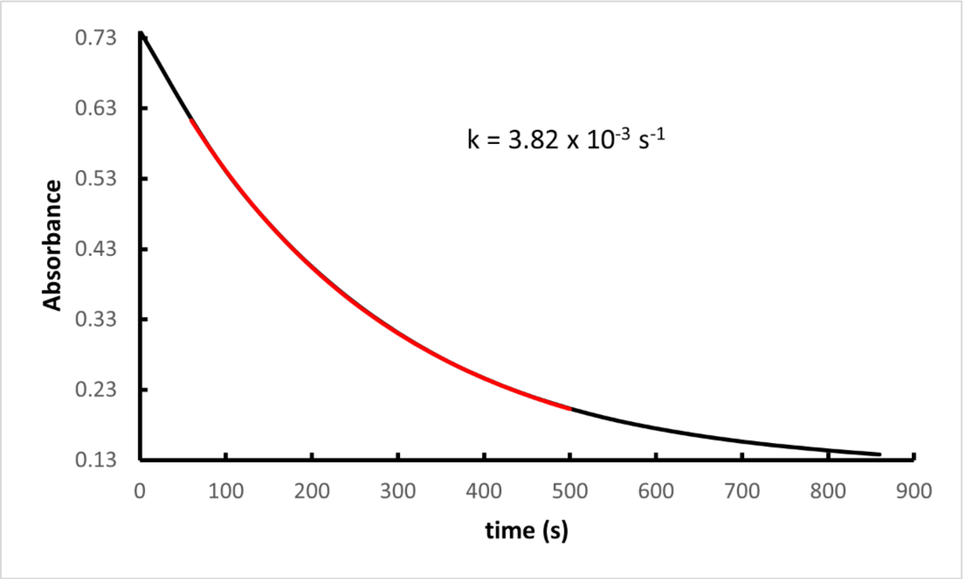

k = 3.82 x 10-3 s-1
Absorbance
time (s)
0.73
0.63
0.53
0.43
0.33
0.23
0.13
0
100
200
300
400
500
600
700
800
900

Results

Type	Rate (s^{-1})	Temperature
C1	4.36×10^{-3}	20.3
C1	4.34×10^{-3}	20.3
C1	3.97×10^{-3}	19.8
Average:	**4.22×10^{-3}**	20.1
Standard error:	**1.26×10^{-4}**	

Type	Rate (s^{-1})	Temperature
C2	3.96×10^{-3}	20.1
C2	3.79×10^{-3}	20
C2	4.14×10^{-3}	20
Average:	**3.97×10^{-3}**	20.0
Standard error:	**1.02×10^{-4}**	

Type	Rate (s^{-1})	Temperature
C3	4.43×10^{-3}	20.3
C3	4.18×10^{-3}	20.2
C3	4.02×10^{-3}	20.1
Average:	**4.21×10^{-3}**	20.2
Standard error:	**1.21×10^{-4}**	

Type	Rate (s^{-1})	Temperature
C5	4.04×10^{-3}	20.2
C5	3.84×10^{-3}	19.9
C5	4.22×10^{-3}	20.1
Average:	**4.03×10^{-3}**	20.1
Standard error:	**1.07×10^{-4}**	

Type	Rate (s^{-1})	Temperature
cell	4.17×10^{-3}	20
cell	3.96×10^{-3}	20.3
cell	3.82×10^{-3}	20
Average:	**3.98×10^{-3}**	20.1
Standard error:	**1.01×10^{-4}**	

Reaction of 3-ethyl-3-pentanol (N9) with electrophile E5:

Cavity measurements (C5)

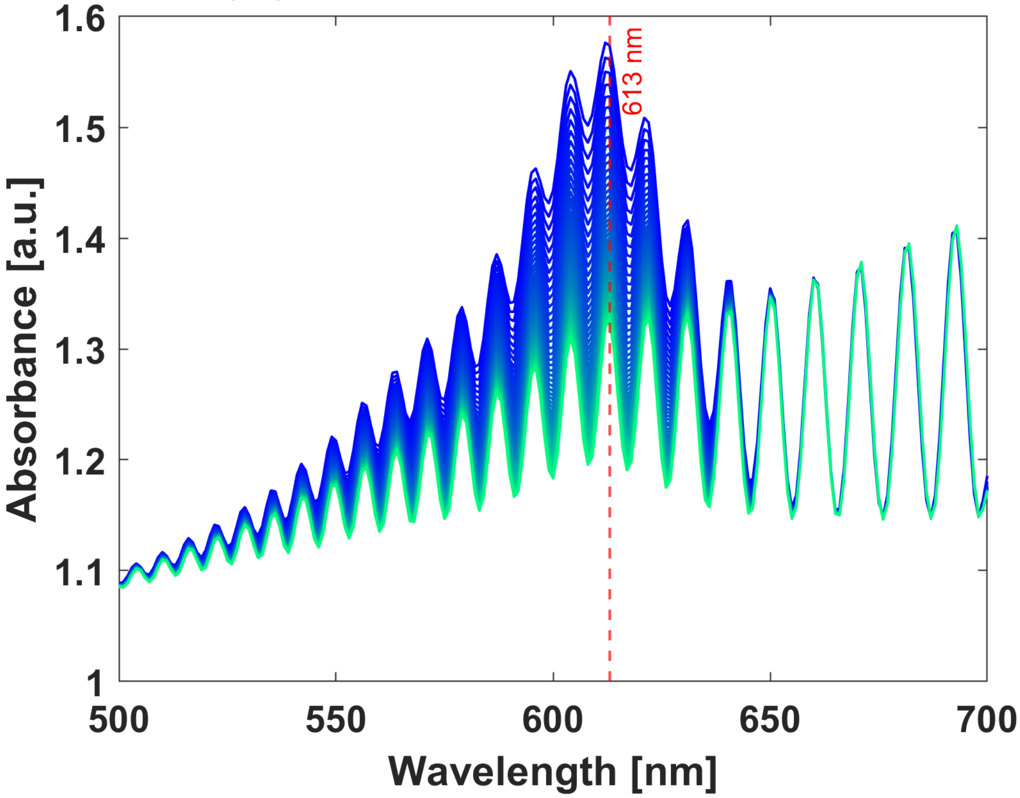

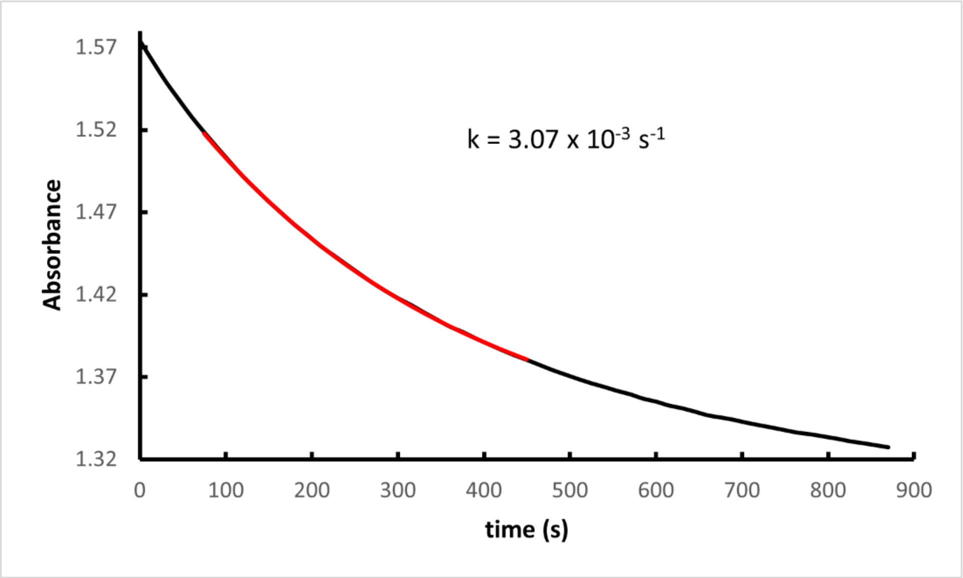

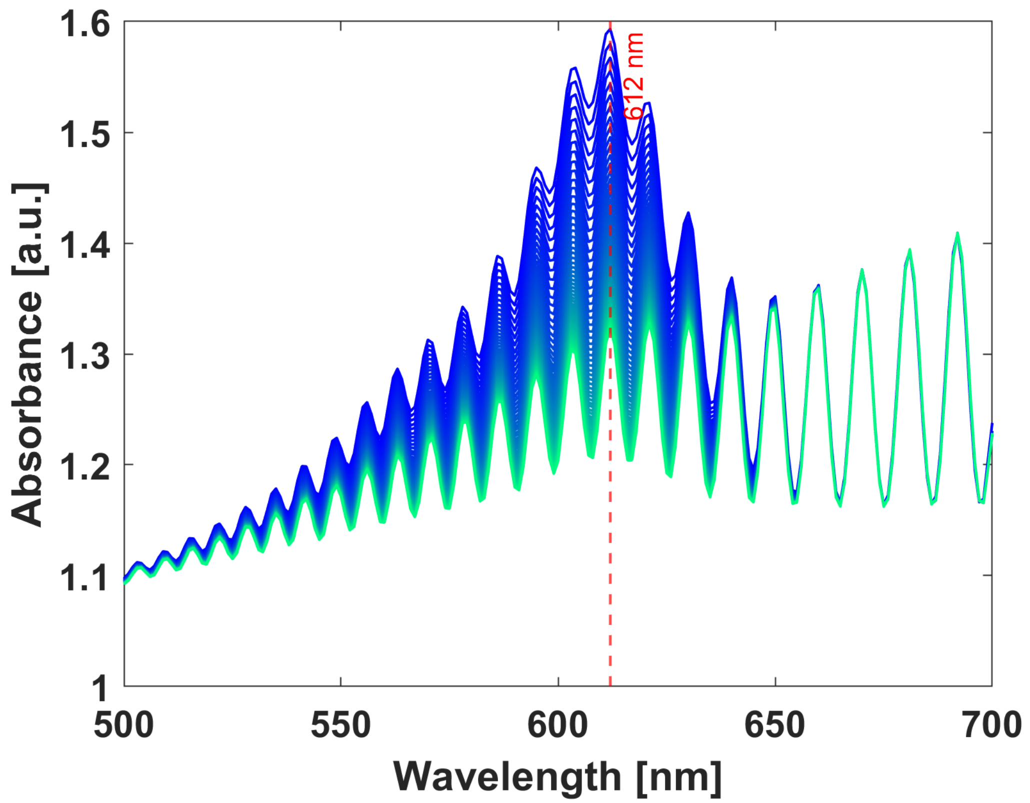

1.6
1.5
1.4
1.3
1.2
1.1
1
Absorbance [a.u.]
612 nm
500
550
600
650
700
Wavelength [nm]

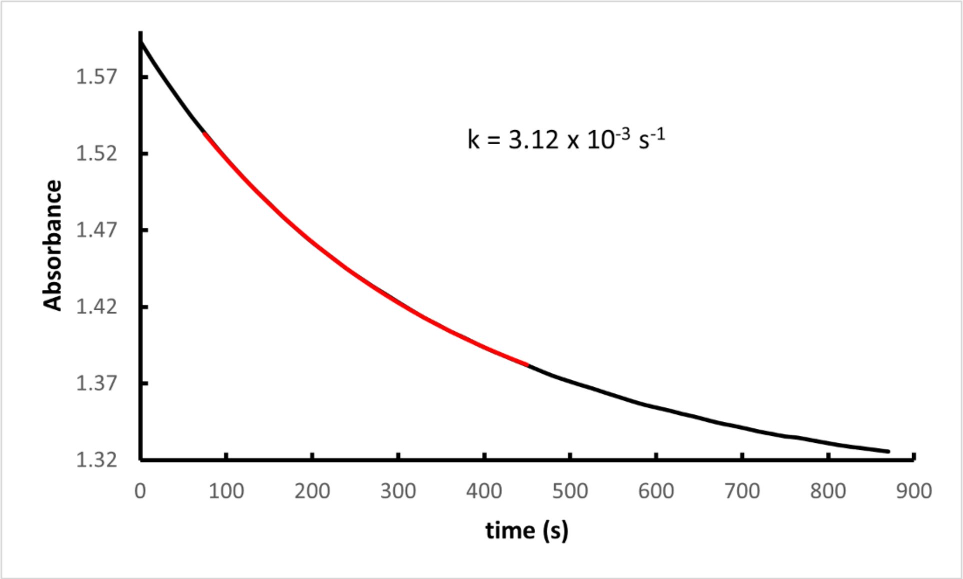

k = 3.12 x 10-3 s-1
1.57
1.52
1.47
1.42
1.37
1.32
Absorbance
0
100
200
300
400
500
600
700
800
900
time (s)

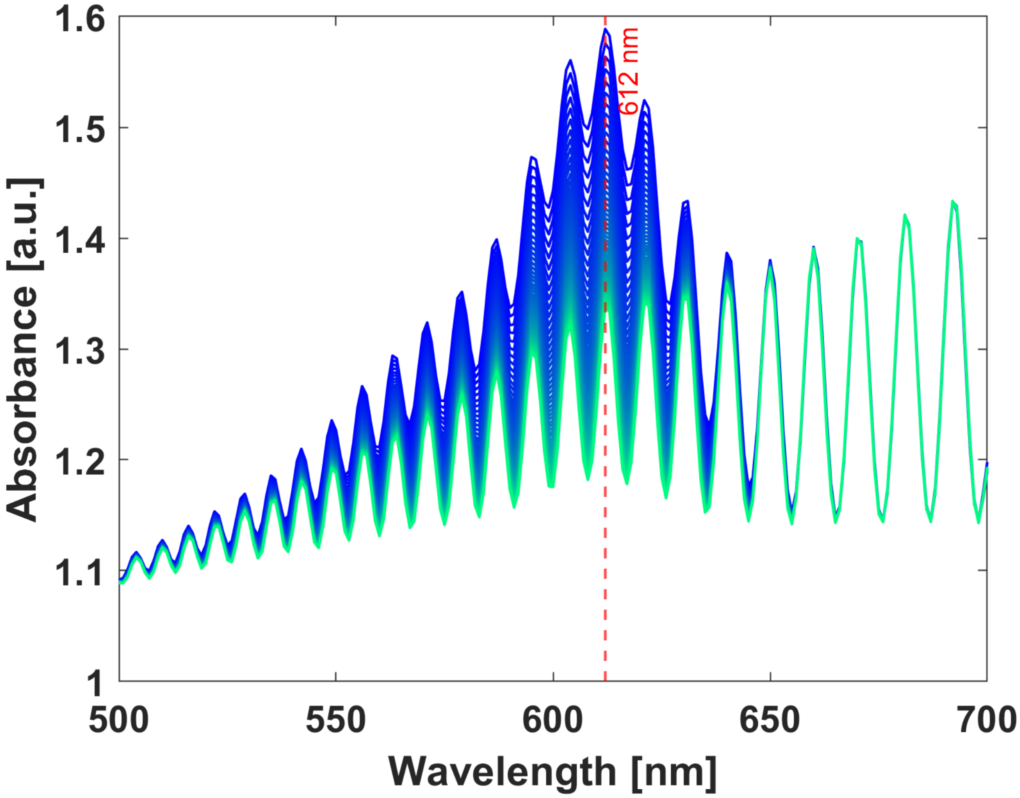

Absorbance [a.u.]
Wavelength [nm]
612 nm
1.6
1.5
1.4
1.3
1.2
1.1
1
500
550
600
650
700

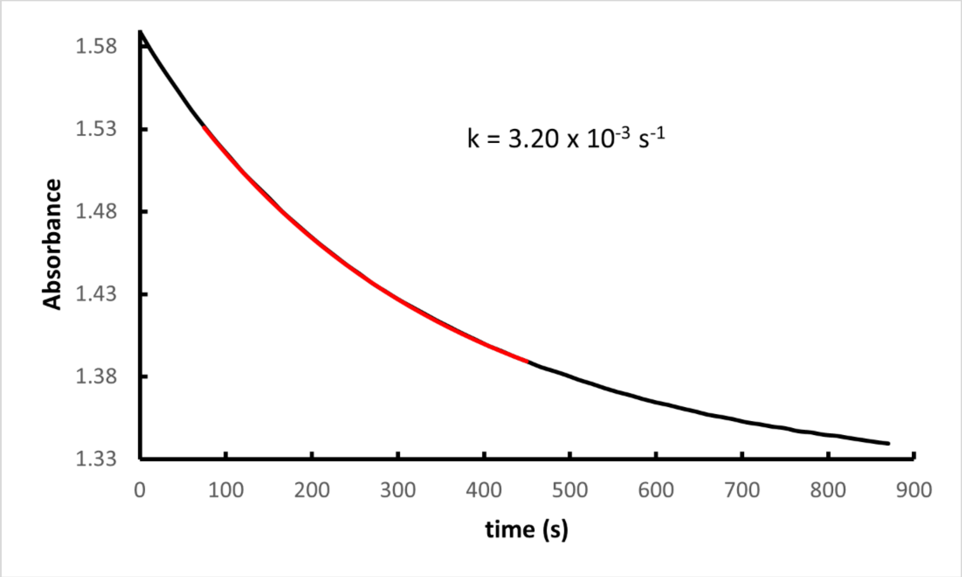

k = 3.20 x 10-3 s-1
Absorbance
time (s)
1.58
1.53
1.48
1.43
1.38
1.33
0
100
200
300
400
500
600
700
800
900

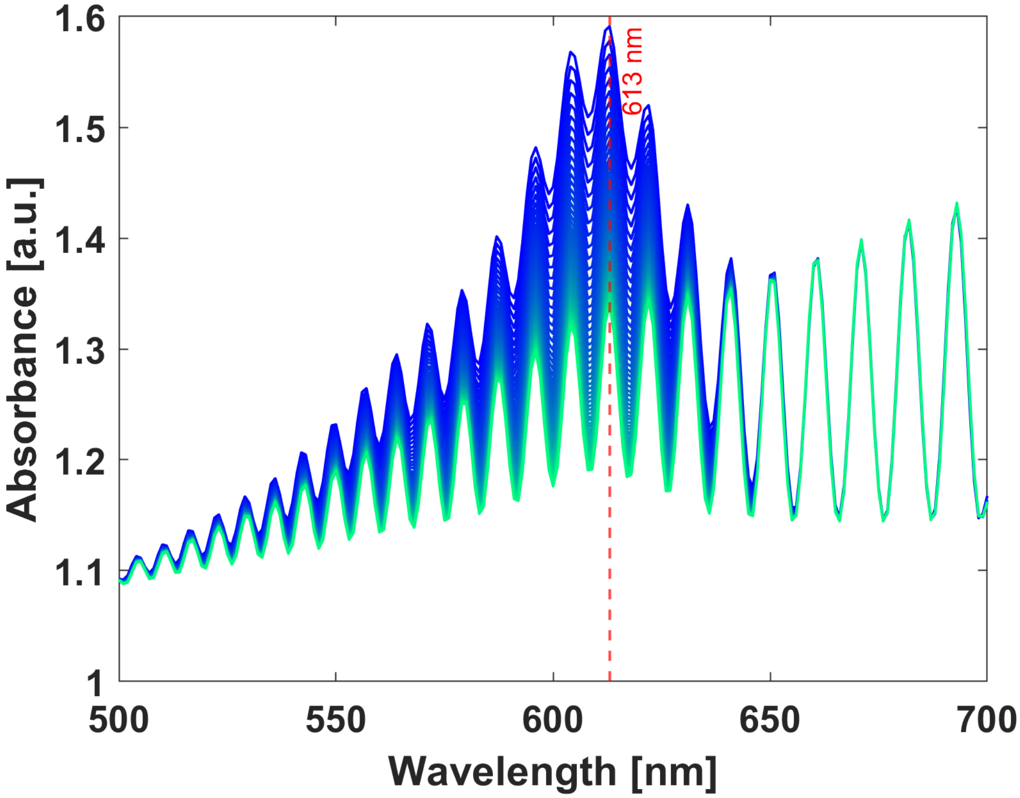

Absorbance [a.u.]
Wavelength [nm]
613 nm
1.6
1.5
1.4
1.3
1.2
1.1
1
500
550
600
650
700

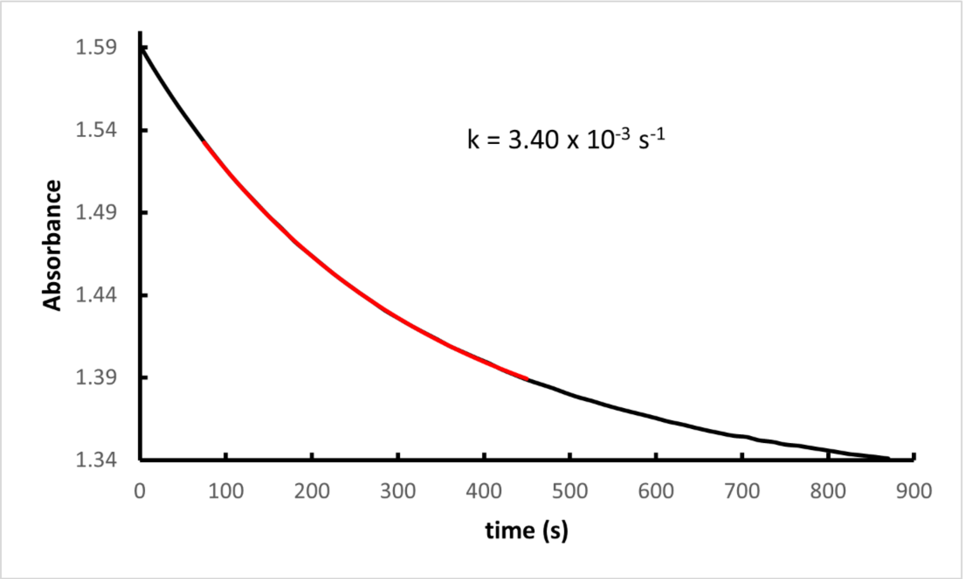

k = 3.40 x 10-3 s-1
Absorbance
time (s)
1.59
1.54
1.49
1.44
1.39
1.34
0
100
200
300
400
500
600
700
800
900

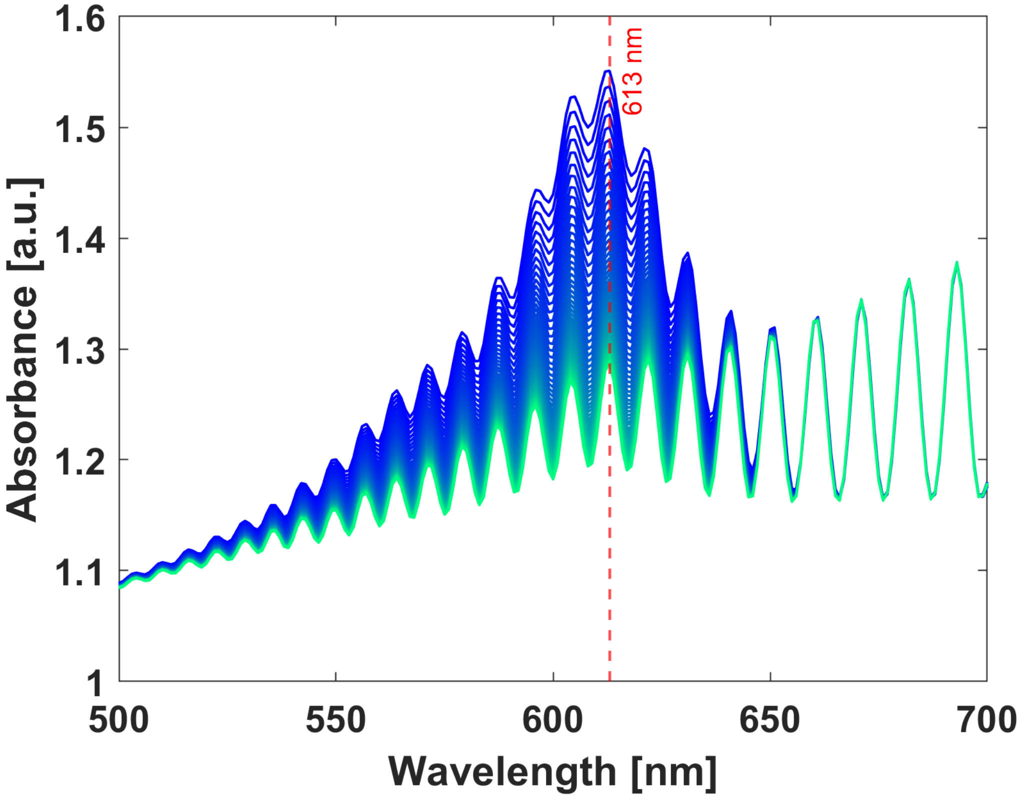

1.6
1.5
1.4
1.3
1.2
1.1
1
Absorbance [a.u.]
613 nm
500
550
600
650
700
Wavelength [nm]

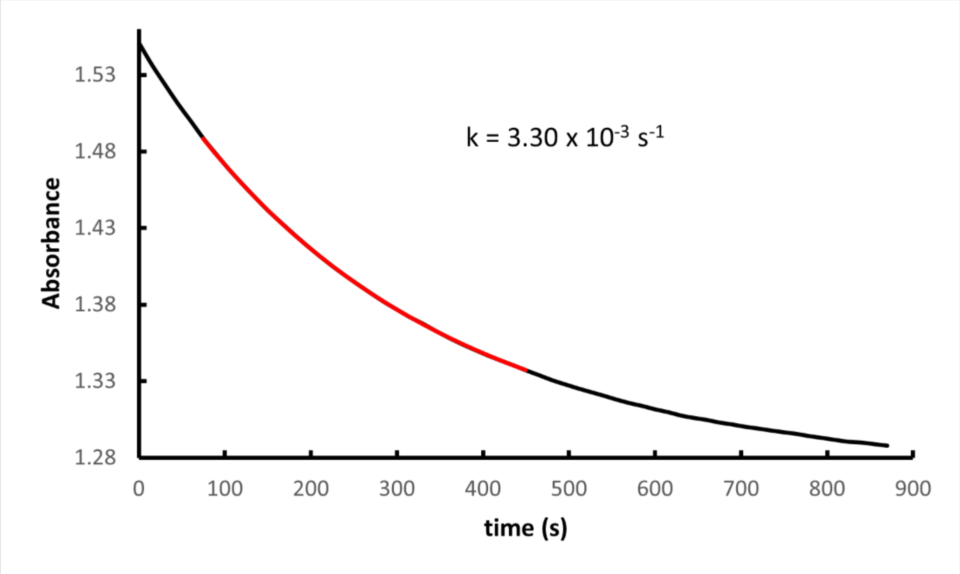

1.53
1.48
1.43
1.38
1.33
1.28
Absorbance
k = 3.30 x 10-3 s-1
0
100
200
300
400
500
600
700
800
900
time (s)

Cell measurements (standard)

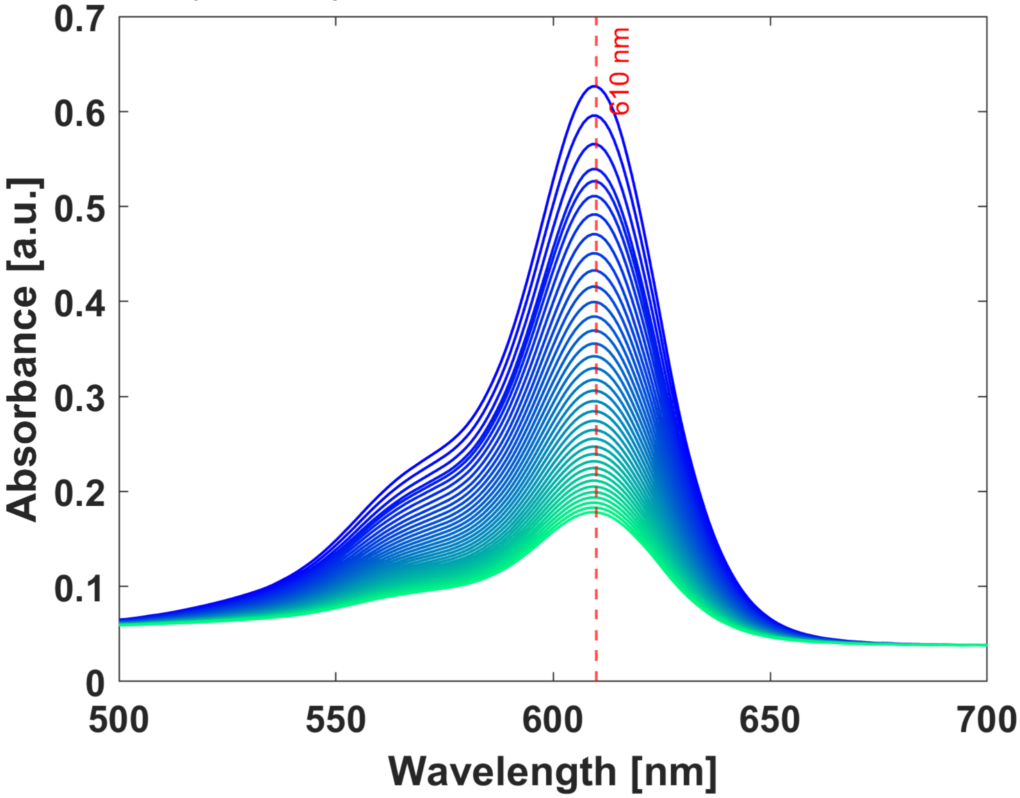

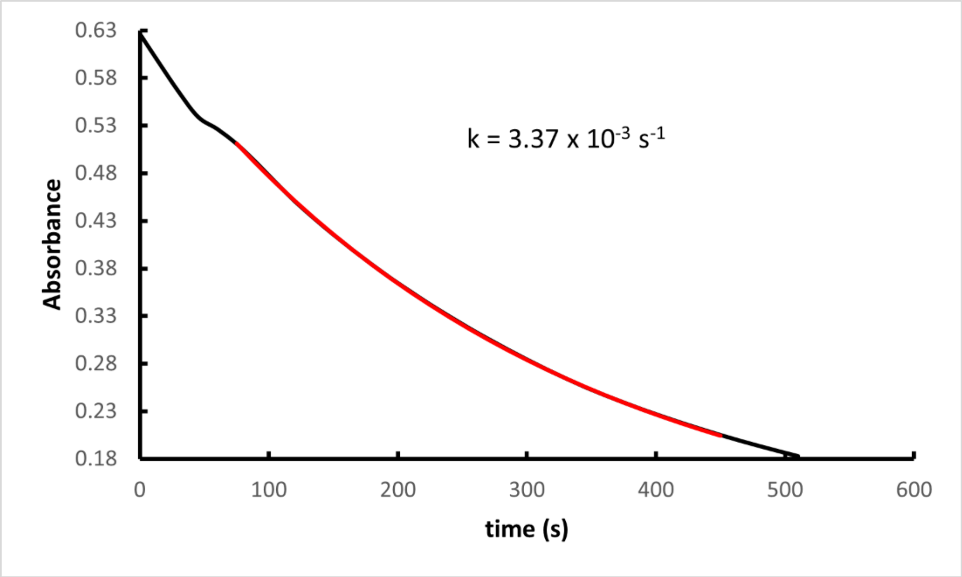

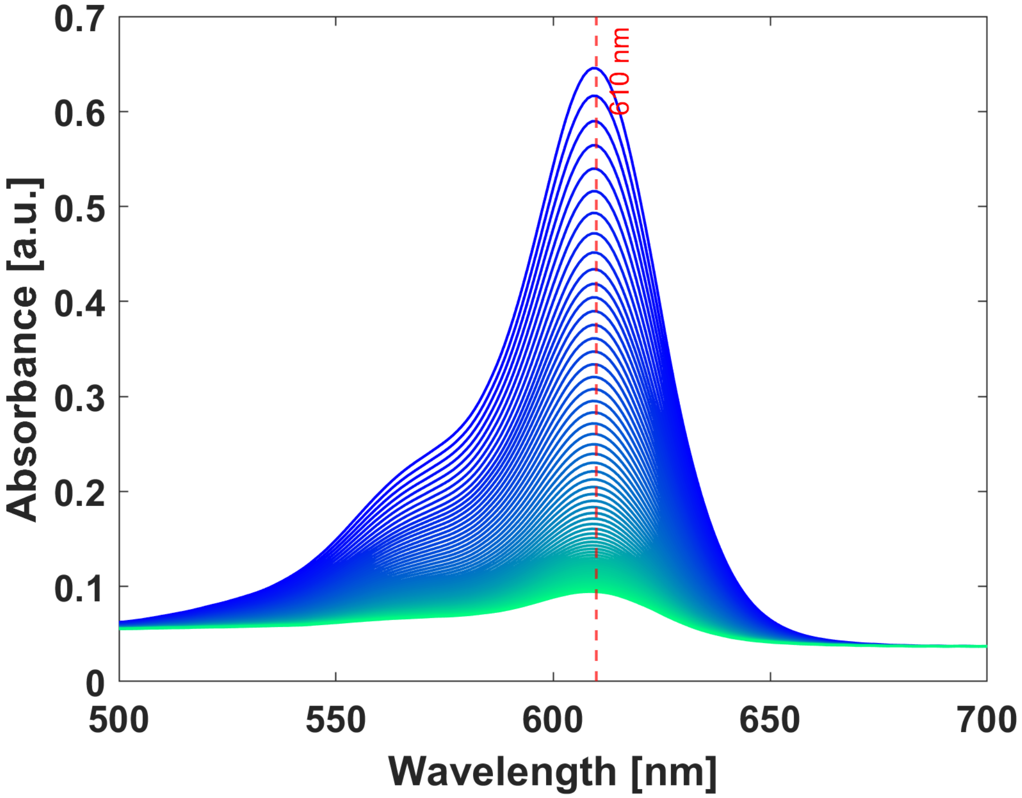

0.7
0.6
0.5
0.4
0.3
0.2
0.1
0
Absorbance [a.u.]
610 nm
500
550
600
650
700
Wavelength [nm]

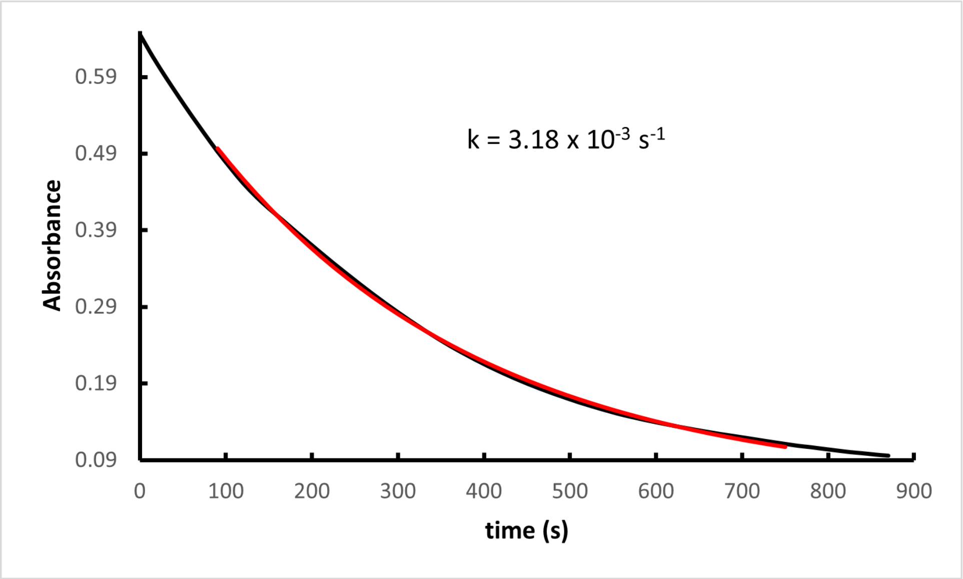

0.59
0.49
0.39
0.29
0.19
0.09
Absorbance
k = 3.18 x 10-3 s-1
0
100
200
300
400
500
600
700
800
900
time (s)

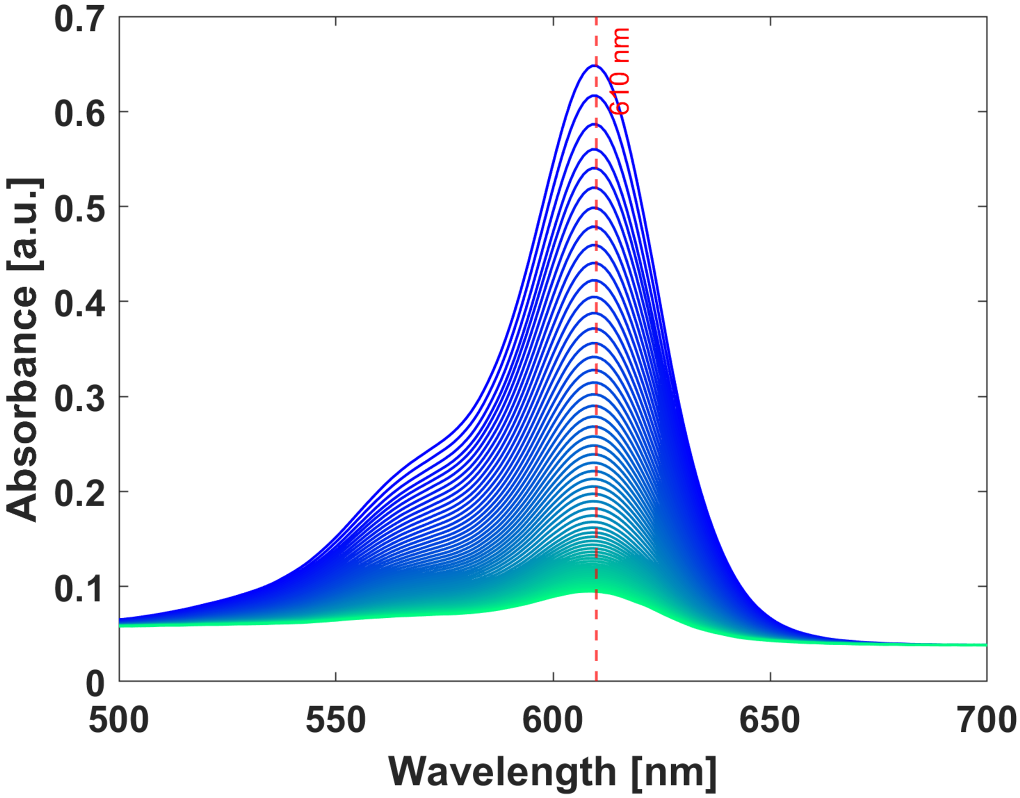

Absorbance [a.u.]
0.7
0.6
0.5
0.4
0.3
0.2
0.1
0
500
550
600
650
700
Wavelength [nm]
610 nm

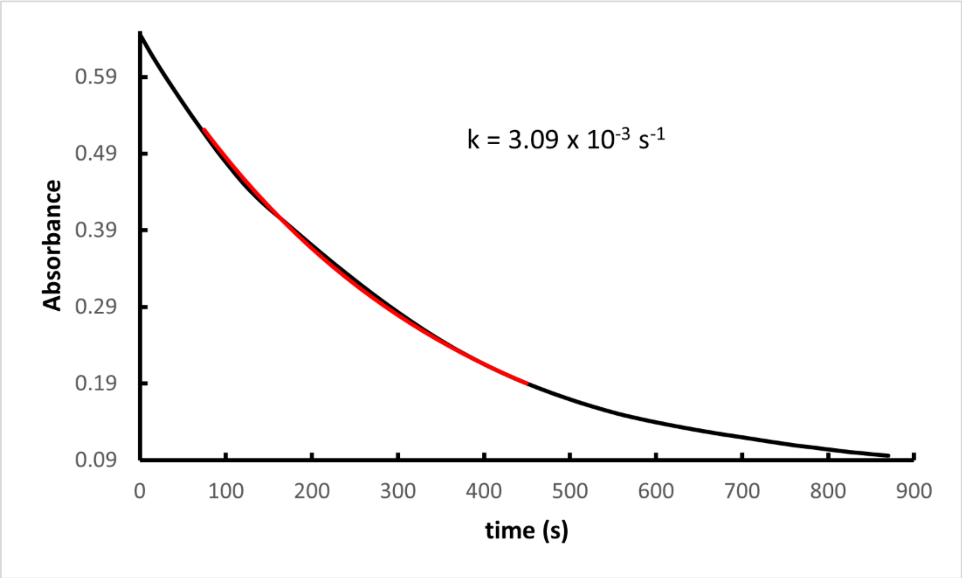

k = 3.09 x 10-3 s-1
Absorbance
0.59
0.49
0.39
0.29
0.19
0.09
0
100
200
300
400
500
600
700
800
900
time (s)

Results

Type	Rate (s^{-1})	Temperature	Type	Rate (s^{-1})	Temperature
C5	$3.07x10^{-3}$	19	cell	$3.37x10^{-3}$	18.9
C5	$3.12x10^{-3}$	19	cell	$3.18x10^{-3}$	18.9
C5	$3.20x10^{-3}$	18.9	cell	$3.09x10^{-3}$	18.9
C5	$3.40x10^{-3}$	18.9	Average:	**$3.22x10^{-3}$**	18.9
C5	$3.30x10^{-3}$	18.8	Standard error:	**$8.06x10^{-5}$**	
Average:	**$3.22x10^{-3}$**	18.9			
Standard error:	**$5.95x10^{-5}$**				

Reaction of benzyl alcohol (N10) with electrophile E2:
Cavity measurements (C5)

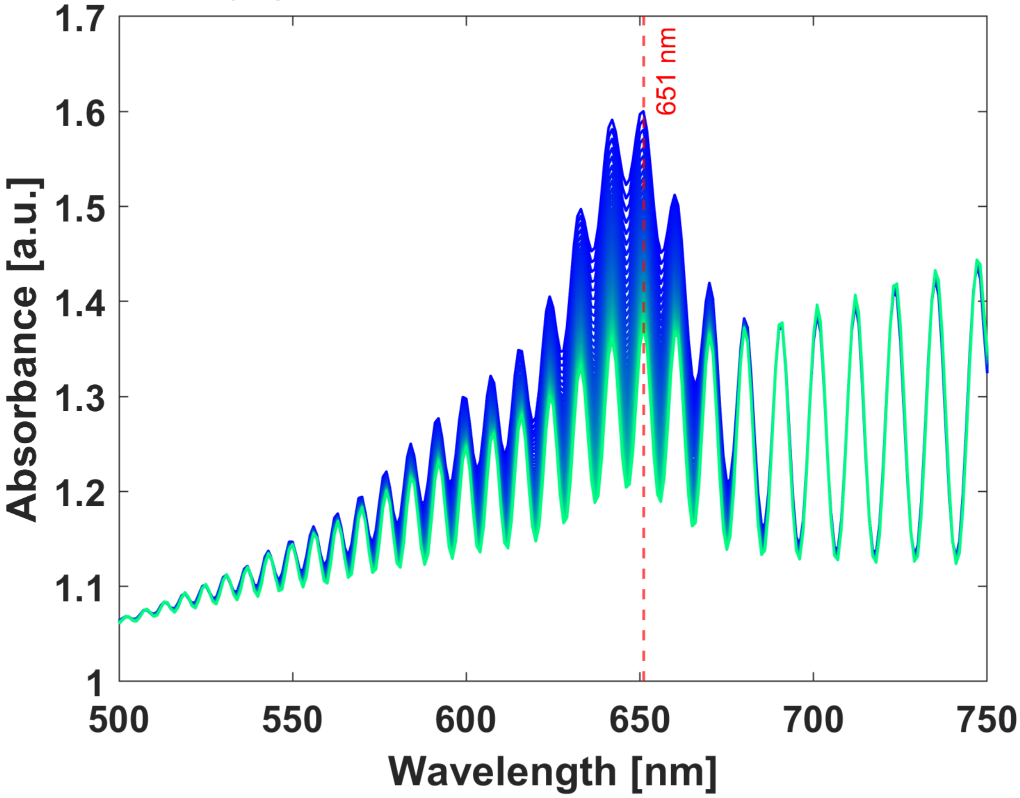

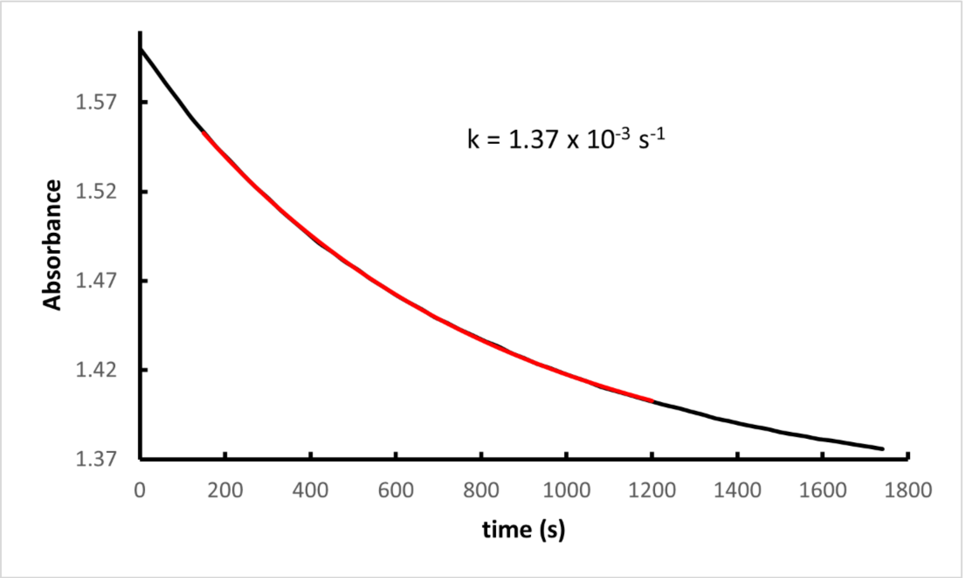

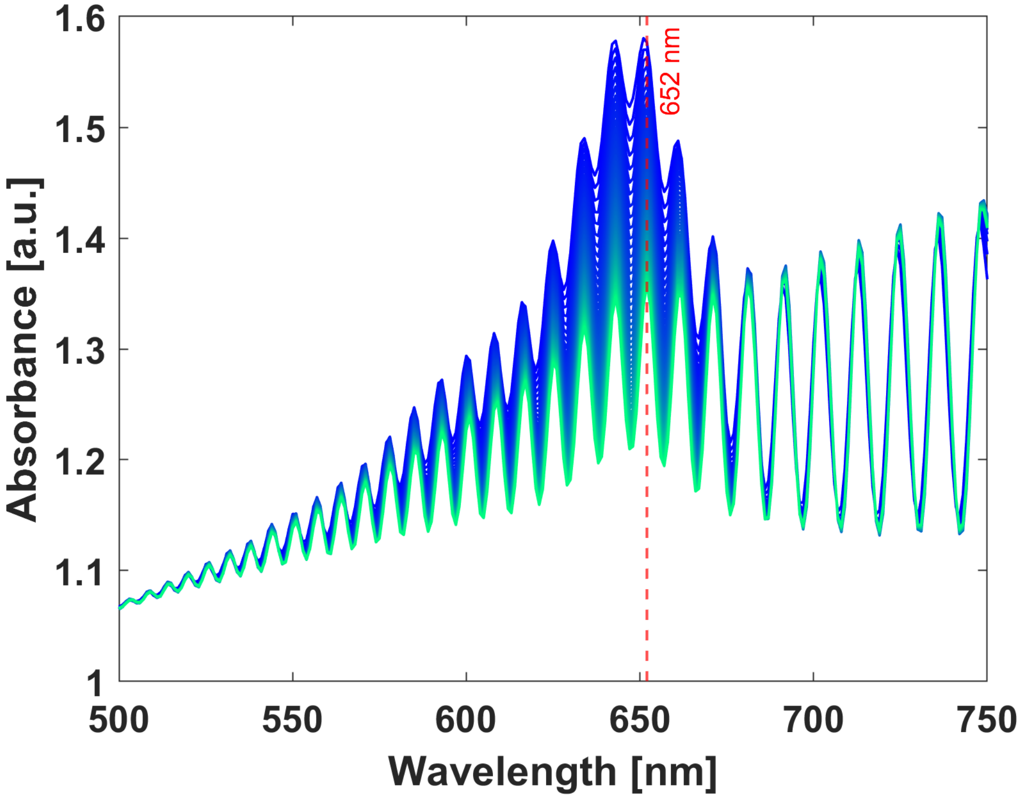

652 nm
Absorbance [a.u.]
Wavelength [nm]
1.6
1.5
1.4
1.3
1.2
1.1
1
500
550
600
650
700
750

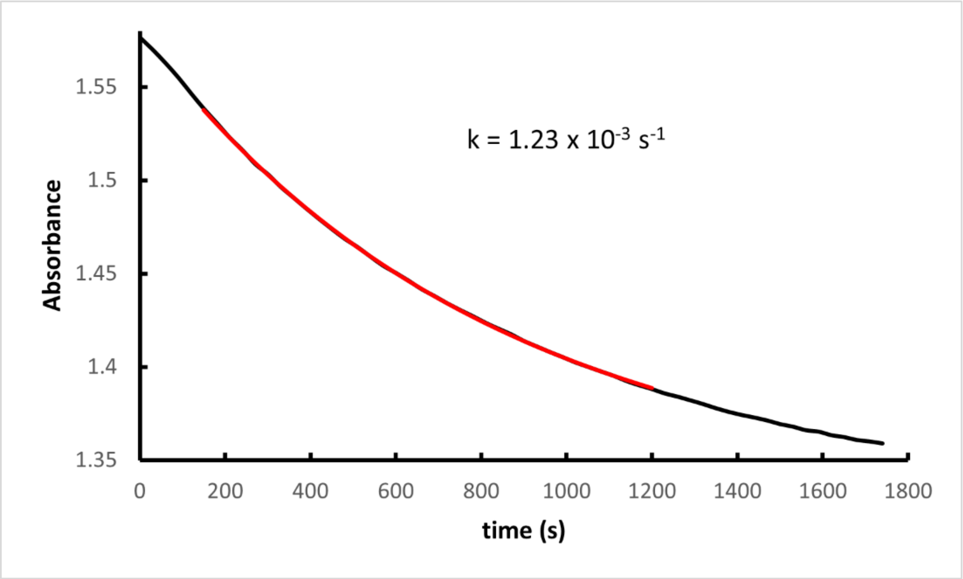

k = 1.23 x 10-3 s-1
Absorbance
time (s)
1.55
1.5
1.45
1.4
1.35
0
200
400
600
800
1000
1200
1400
1600
1800

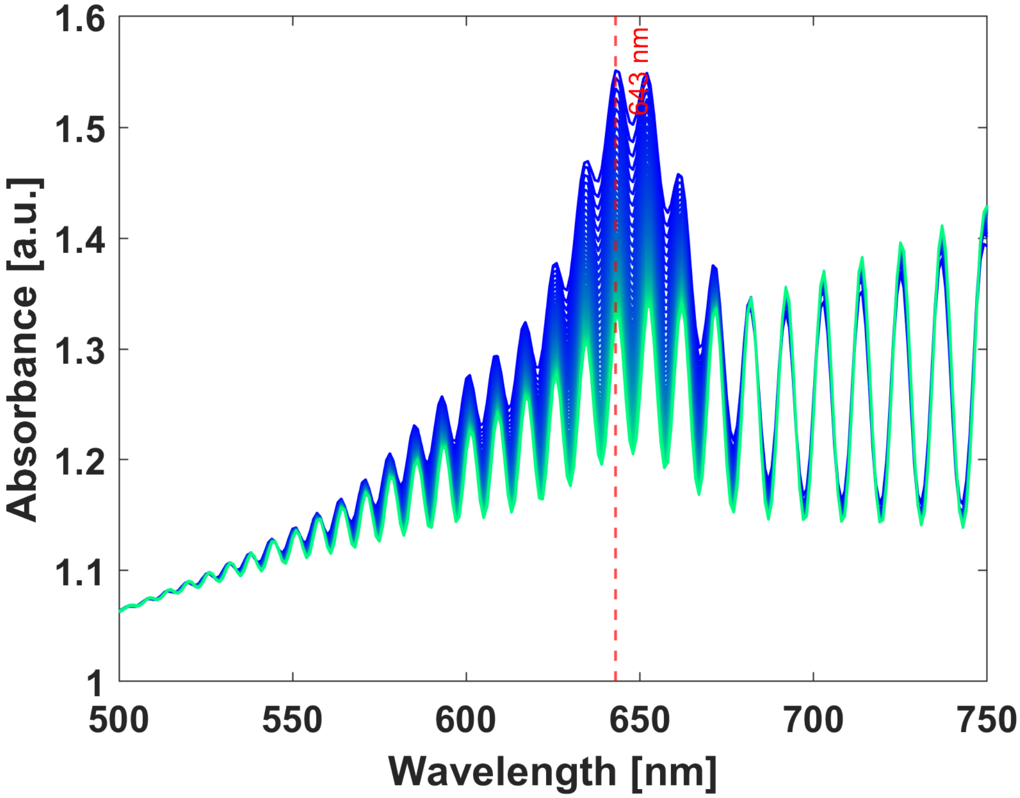

1.6
1.5
1.4
1.3
1.2
1.1
1
500
550
600
650
700
750
Absorbance [a.u.]
Wavelength [nm]
643 nm

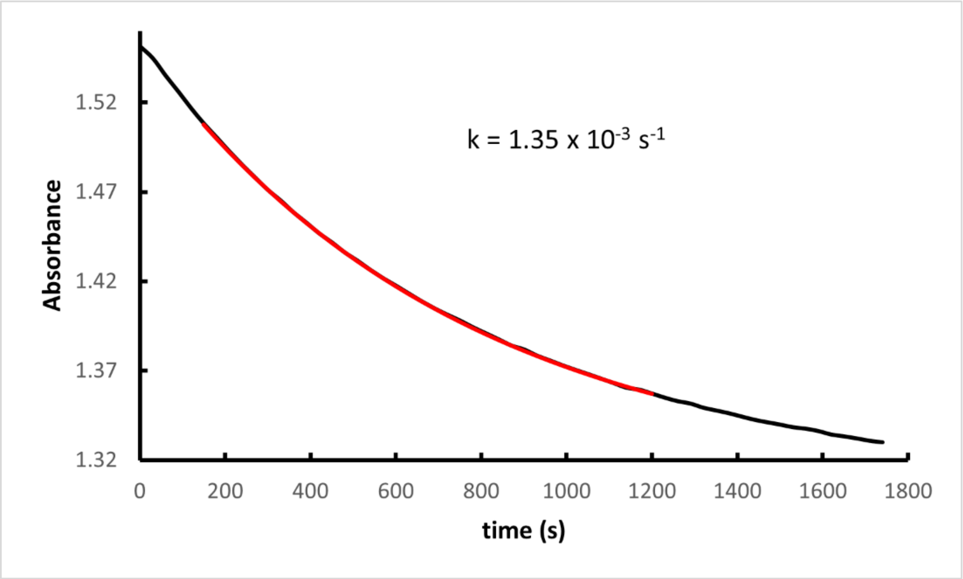

k = 1.35 x 10^{-3} s^{-1}
Absorbance
1.52
1.47
1.42
1.37
1.32
0
200
400
600
800
1000
1200
1400
1600
1800
time (s)

Cell measurements (standard)

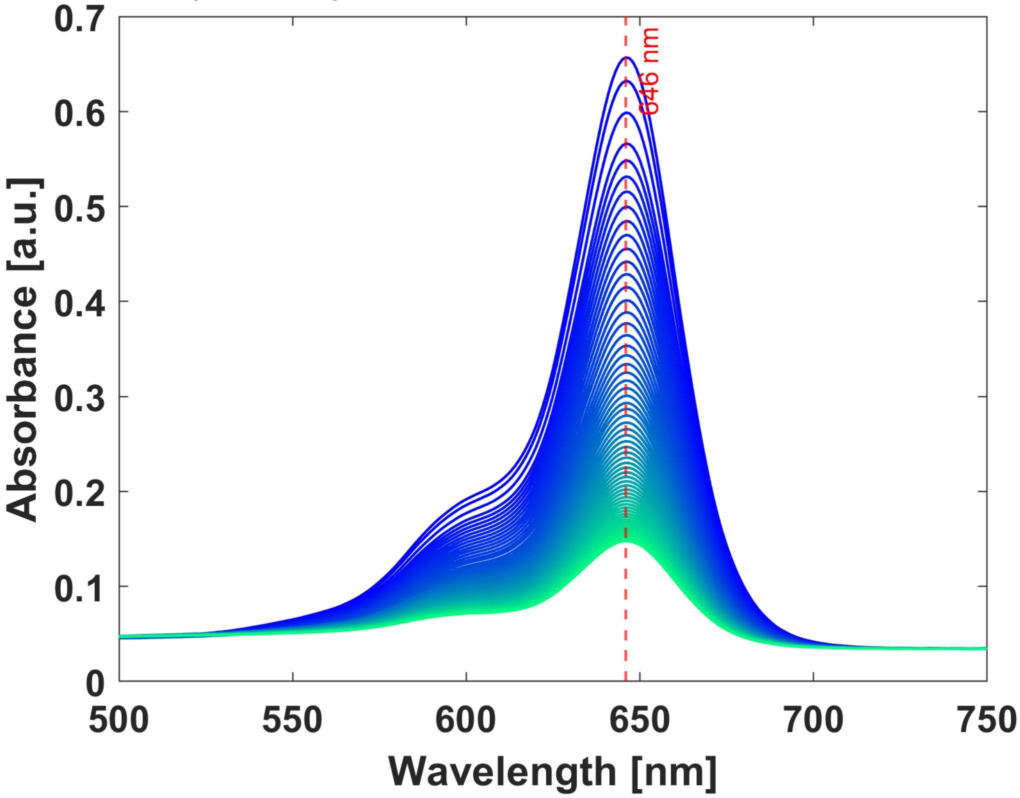

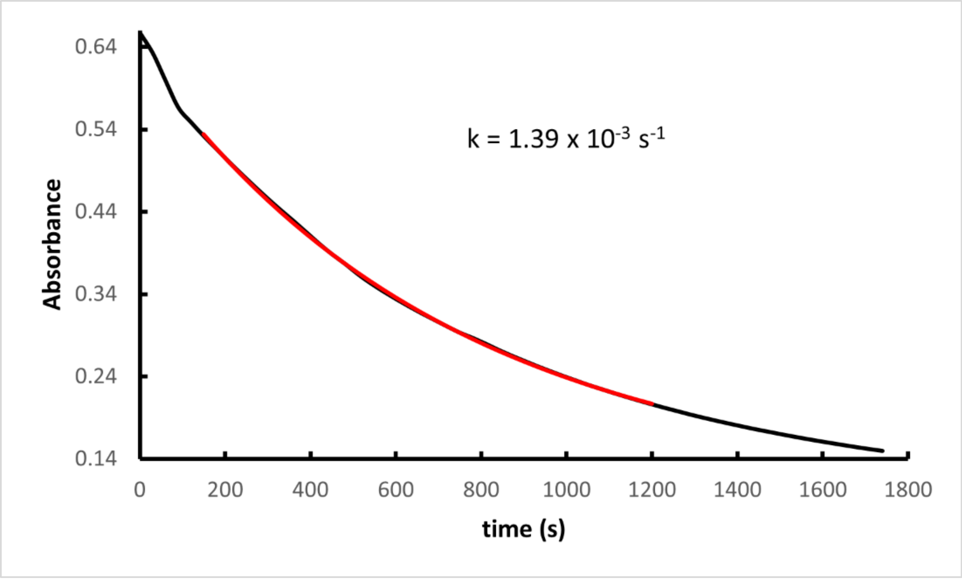

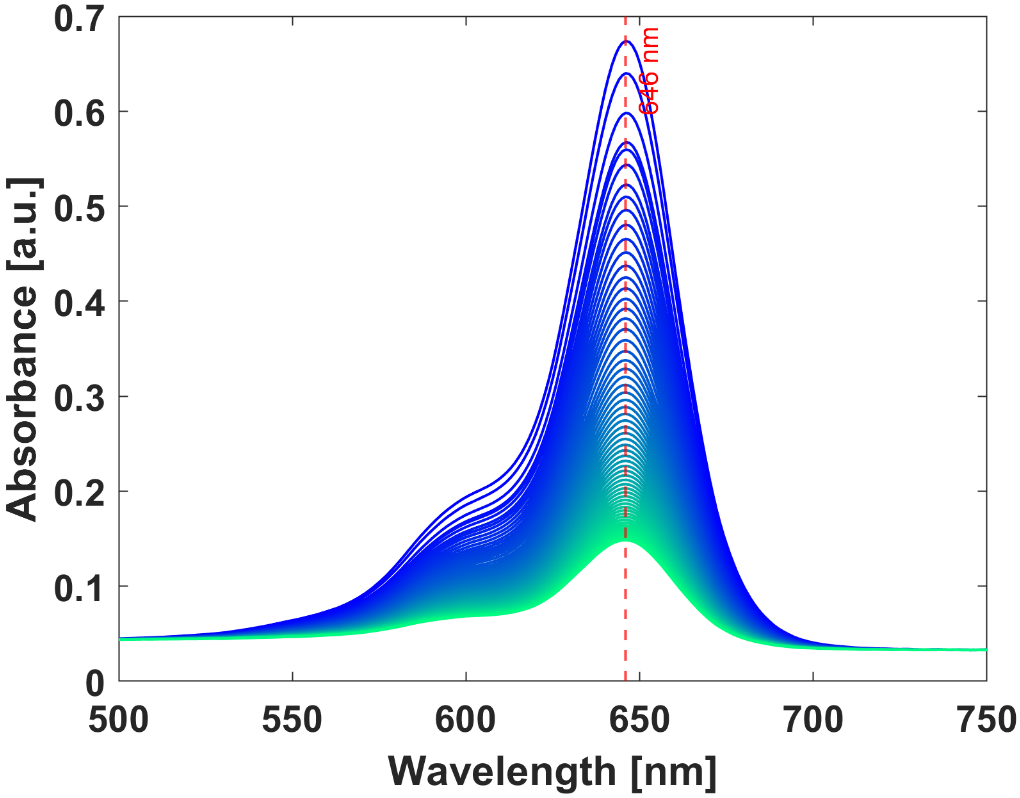
0.7
0.6
0.5
0.4
0.3
0.2
0.1
0
Absorbance [a.u.]
646 nm
500
550
600
650
700
750
Wavelength [nm]

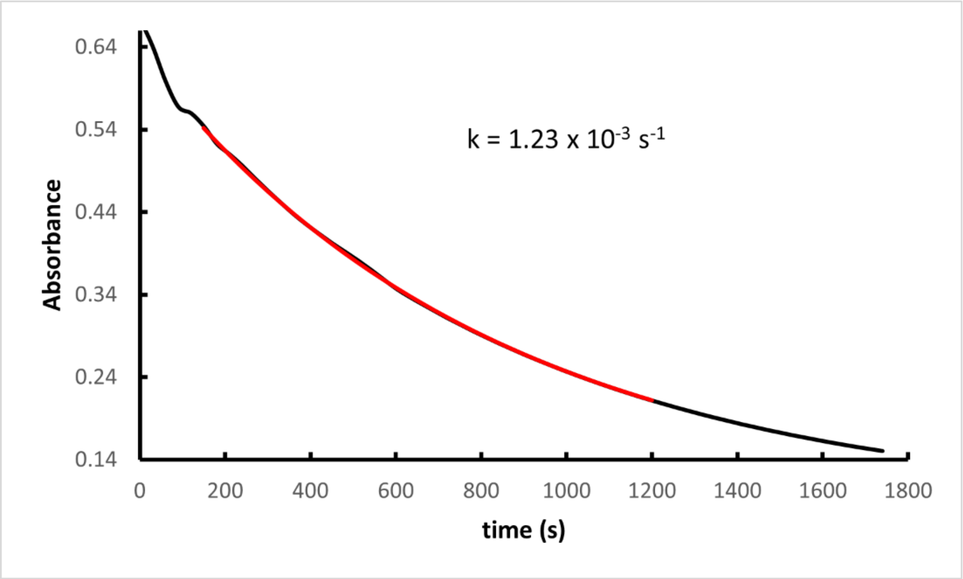
0.64
0.54
0.44
0.34
0.24
0.14
Absorbance
k = 1.23 x 10-3 s-1
0
200
400
600
800
1000
1200
1400
1600
1800
time (s)

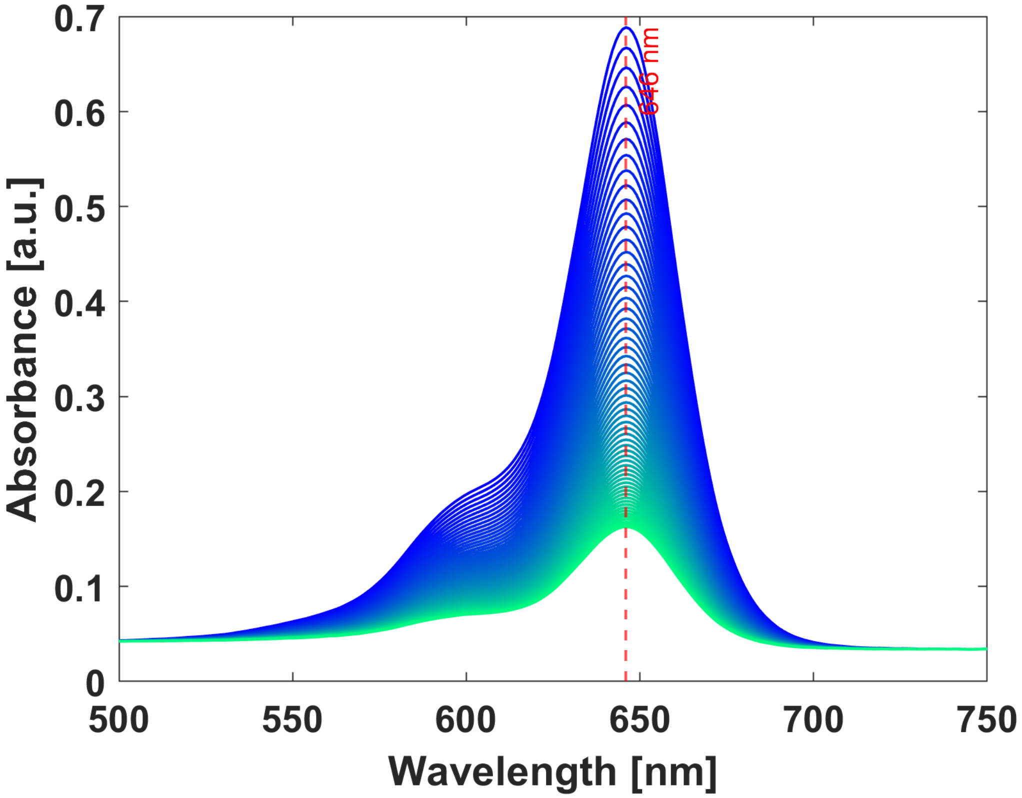

Absorbance [a.u.]
Wavelength [nm]
646 nm
0
0.1
0.2
0.3
0.4
0.5
0.6
0.7
500
550
600
650
700
750

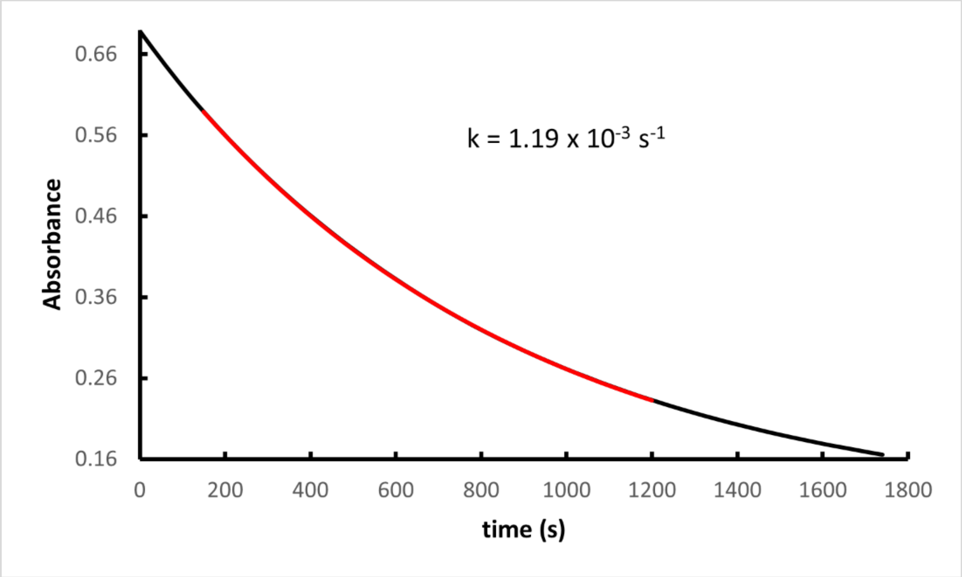

k = 1.19 x 10-3 s-1
Absorbance
time (s)
0.66
0.56
0.46
0.36
0.26
0.16
0
200
400
600
800
1000
1200
1400
1600
1800

Results

Type	Rate (s^{-1})	Temperature	Type	Rate (s^{-1})	Temperature
C5	$1.37x10^{-3}$	22.6	cell	$1.39x10^{-3}$	22.4
C5	$1.23x10^{-3}$	22.5	cell	$1.23x10^{-3}$	22.4
C5	$1.35x10^{-3}$	22.5	cell	$1.19x10^{-3}$	22.3
Average:	**$1.32x10^{-3}$**	22.5	Average:	**$1.27x10^{-3}$**	22.4
Standard error:	**$4.27x10^{-5}$**		Standard error:	**$6.17x10^{-5}$**	

Reaction of water (N11) with electrophile E4:
Cavity measurements (C5)

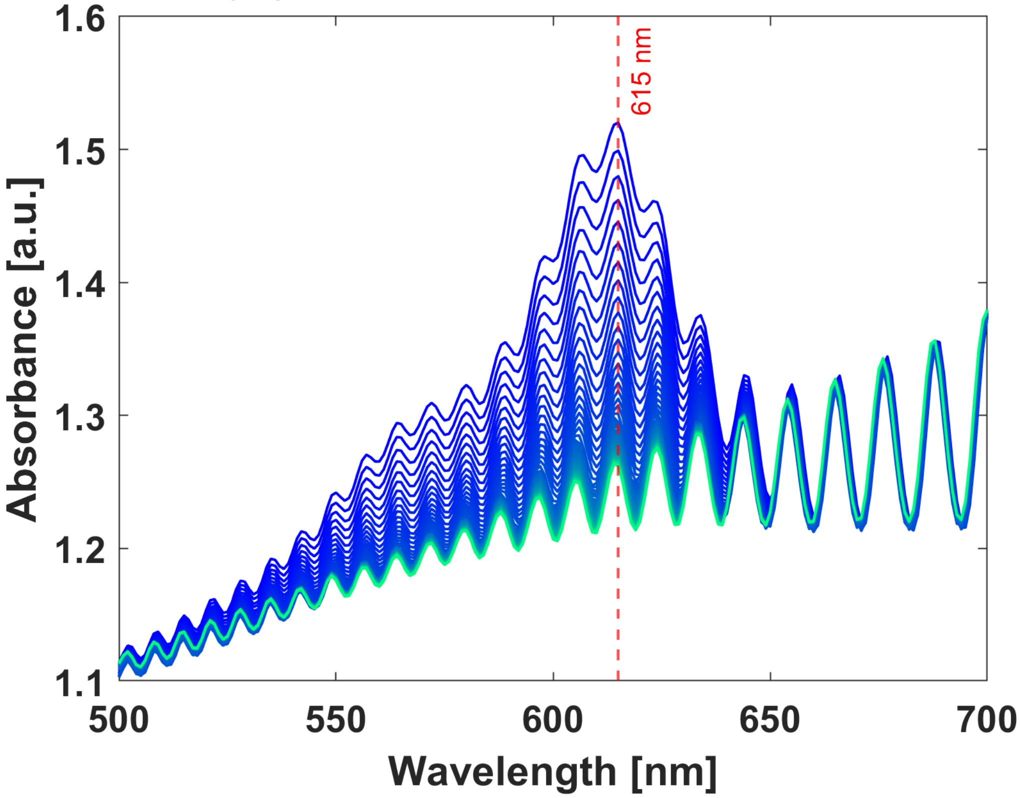

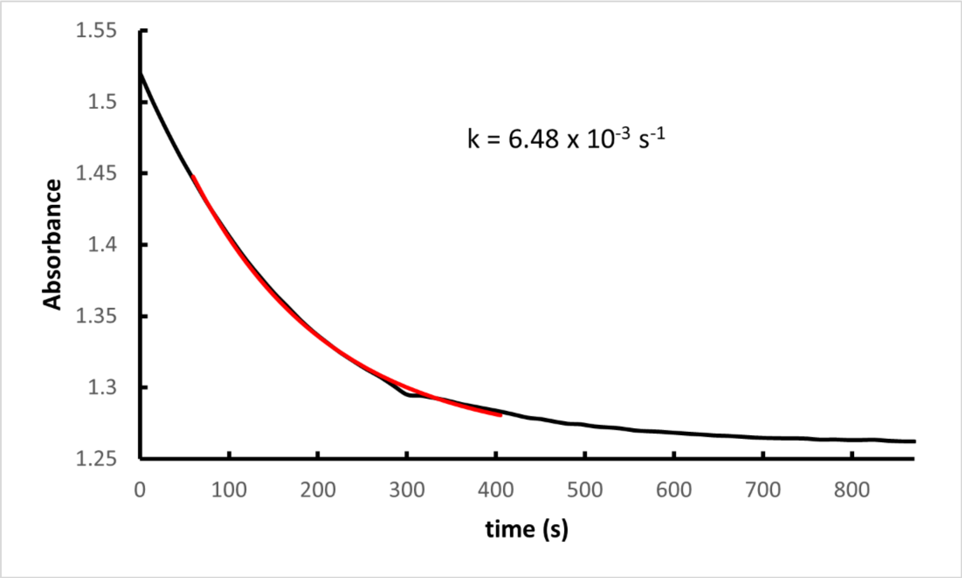

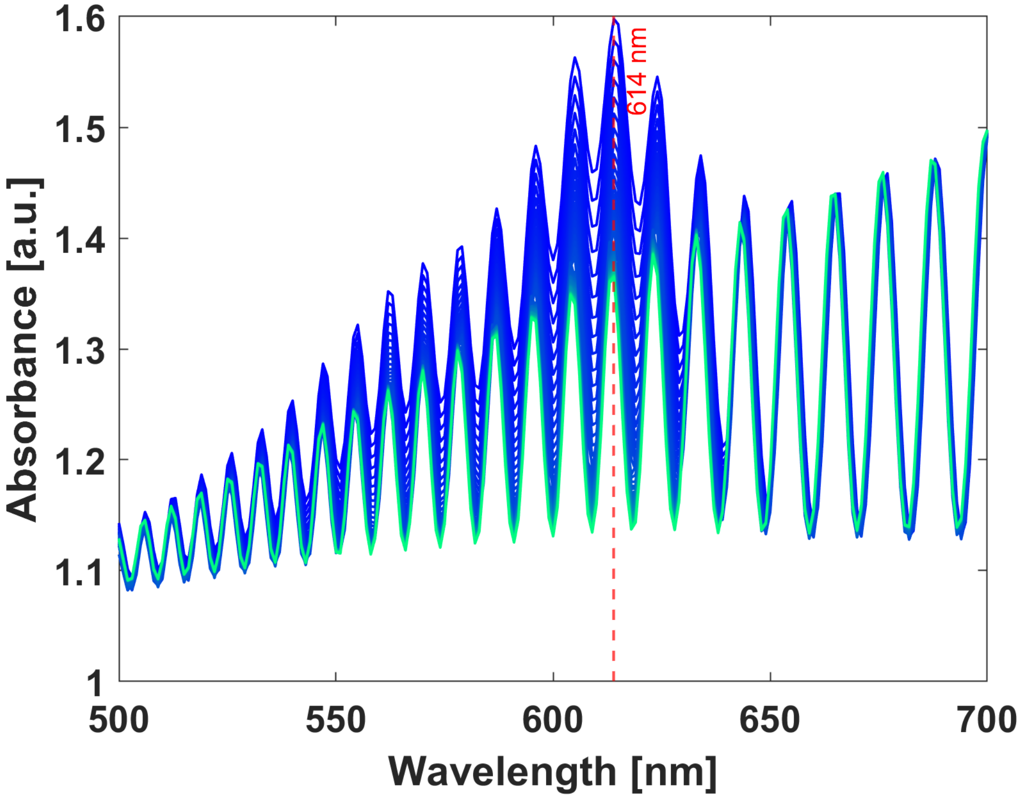

614 nm
Absorbance [a.u.]
Wavelength [nm]

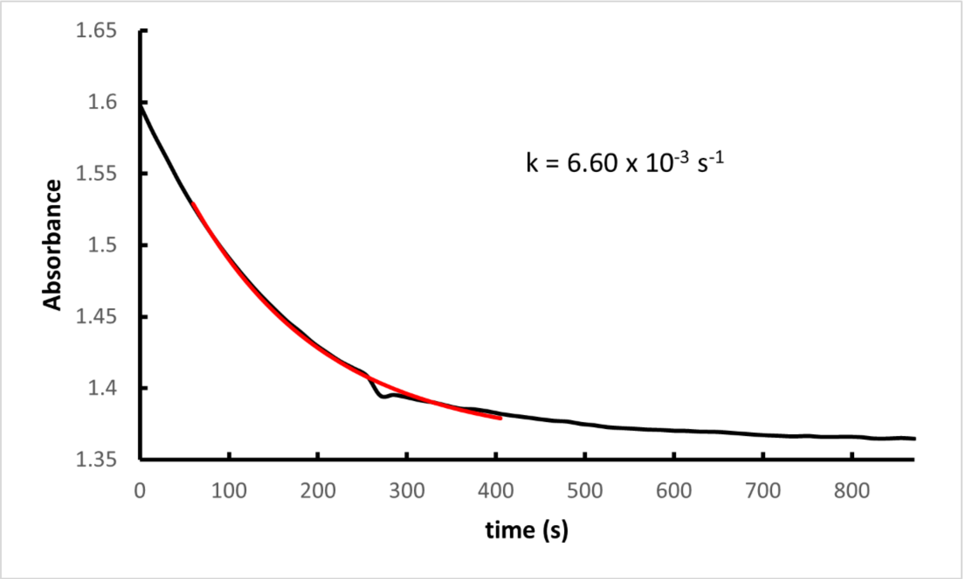

k = 6.60 x 10-3 s-1
Absorbance
time (s)

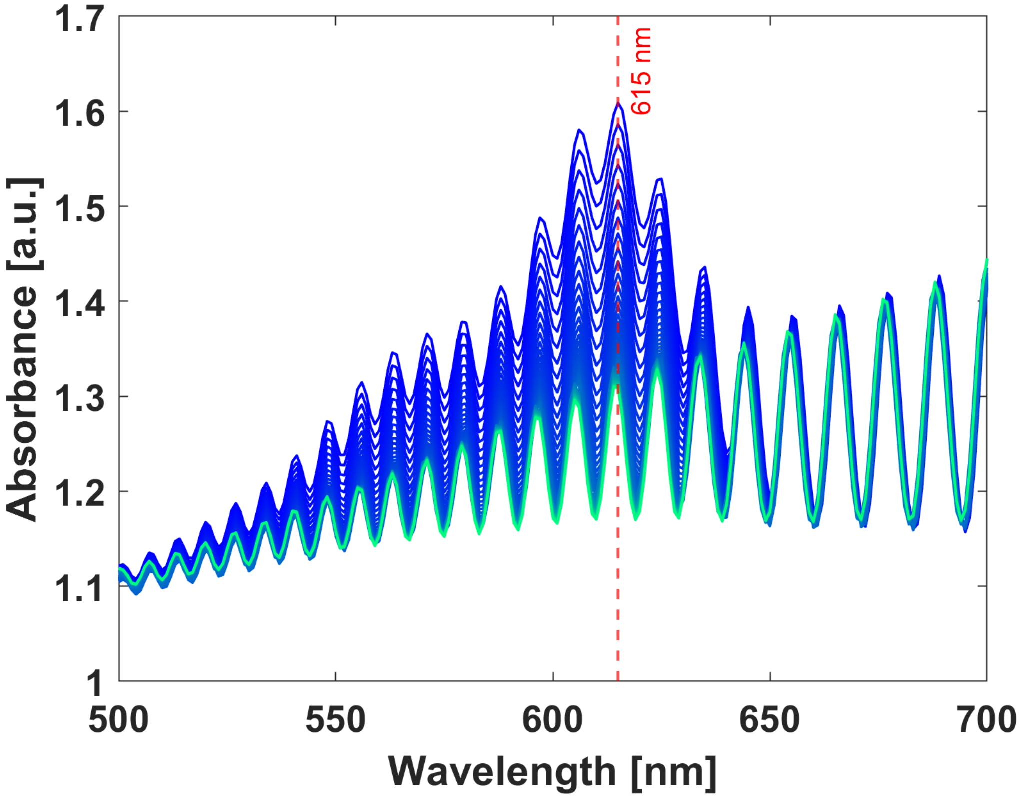

615 nm
Absorbance [a.u.]
Wavelength [nm]
1
1.1
1.2
1.3
1.4
1.5
1.6
1.7
500
550
600
650
700

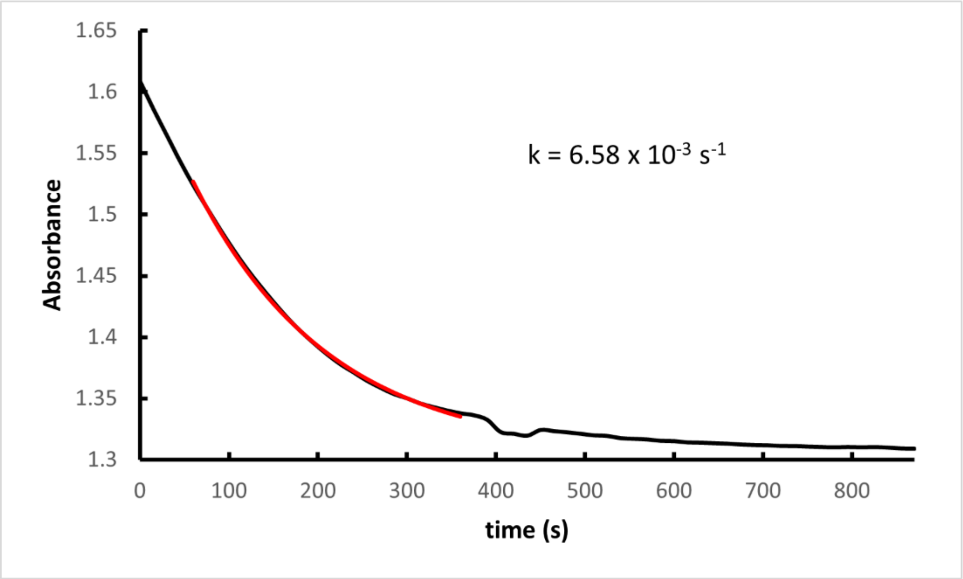

k = 6.58 x 10-3 s-1
Absorbance
time (s)
1.3
1.35
1.4
1.45
1.5
1.55
1.6
1.65
0
100
200
300
400
500
600
700
800

Cell measurements (standard)

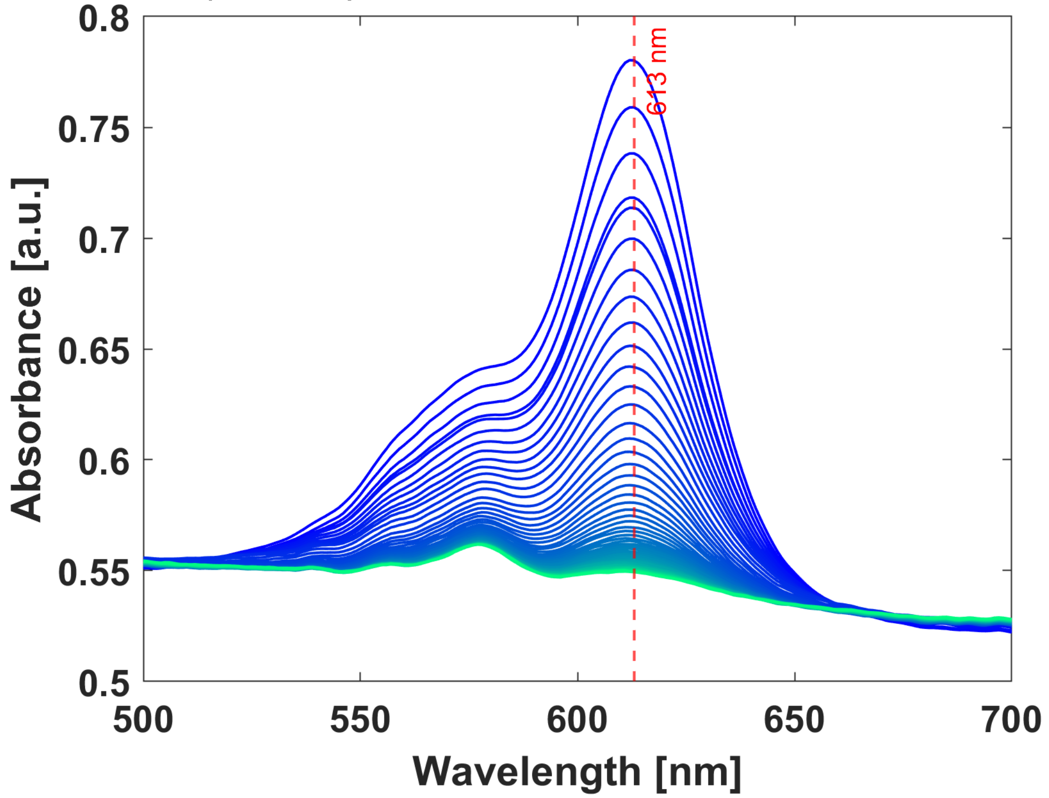

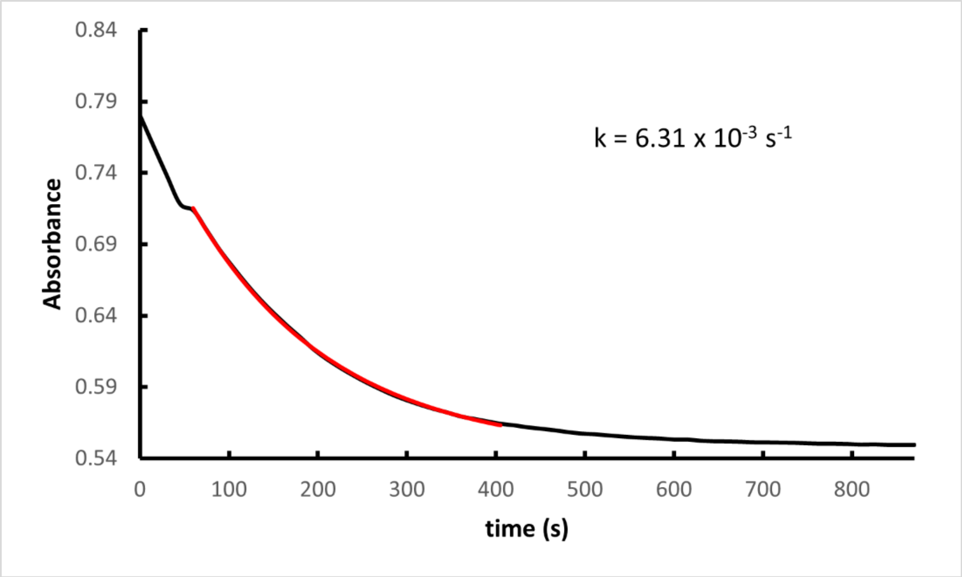

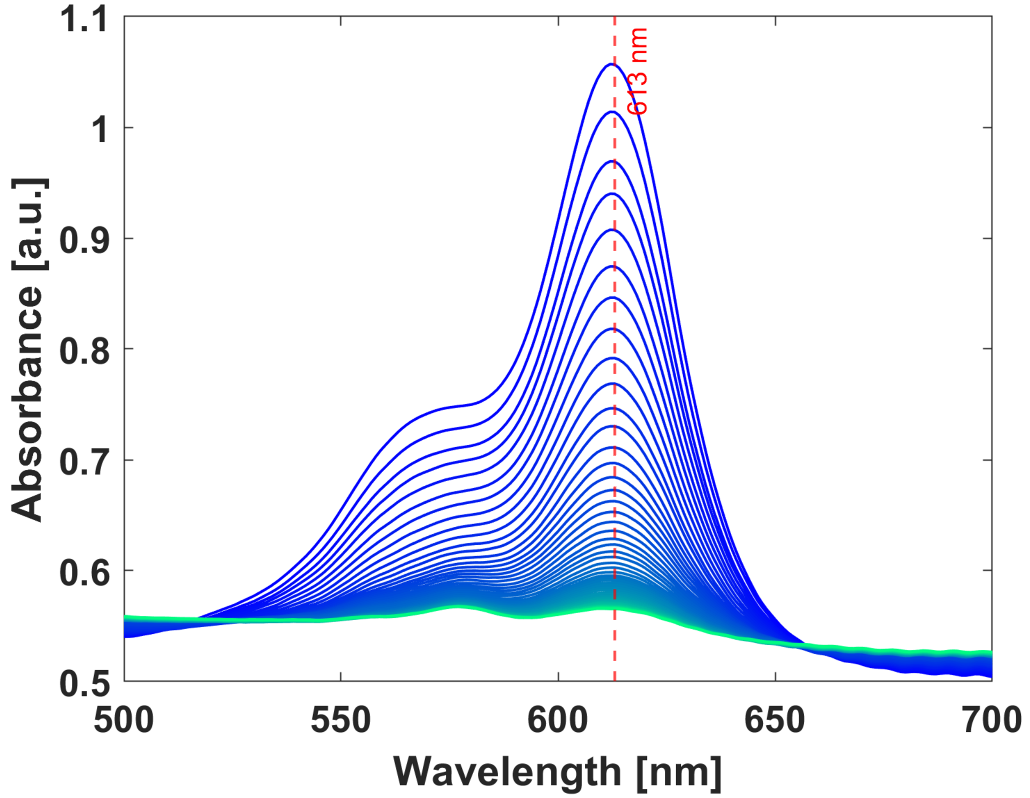

613 nm
1.1
1
0.9
0.8
0.7
0.6
0.5
Absorbance [a.u.]
500
550
600
650
700
Wavelength [nm]

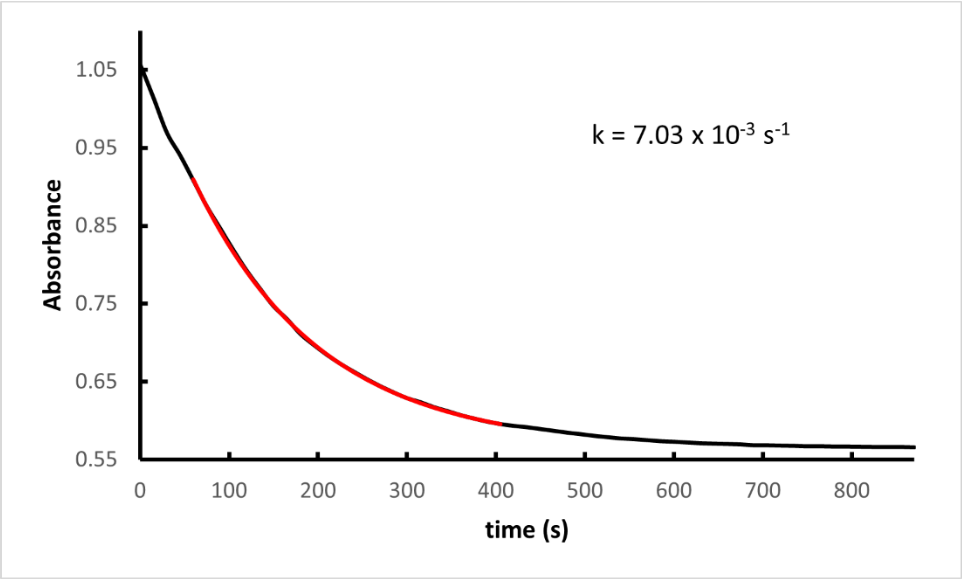

k = 7.03 x 10-3 s-1
1.05
0.95
0.85
0.75
0.65
0.55
Absorbance
0
100
200
300
400
500
600
700
800
time (s)

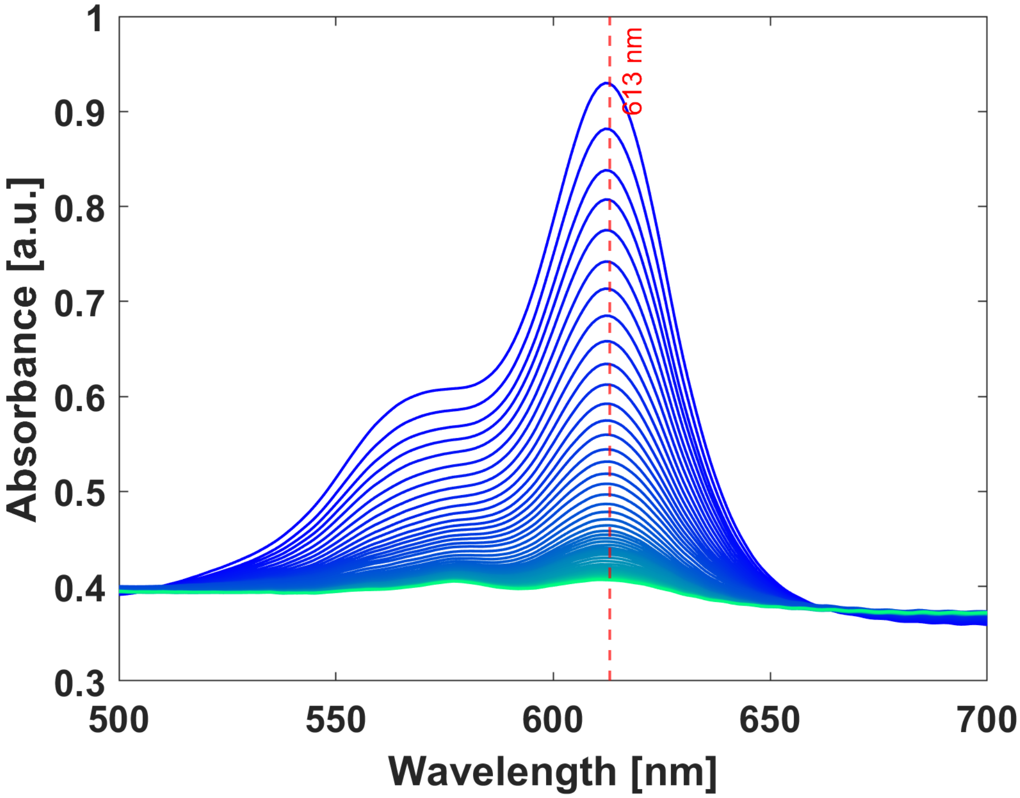

613 nm
Absorbance [a.u.]
Wavelength [nm]
1
0.9
0.8
0.7
0.6
0.5
0.4
0.3
500
550
600
650
700

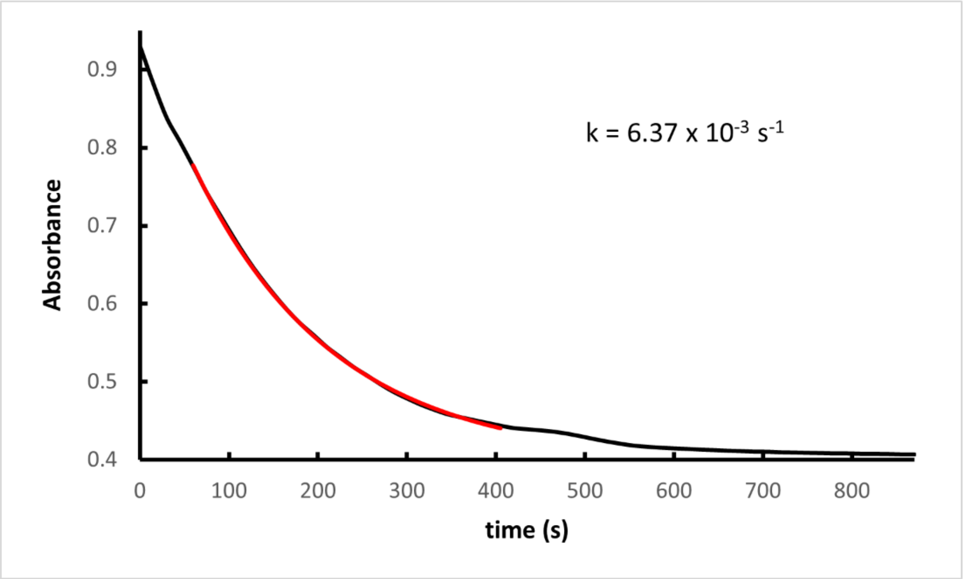

k = 6.37 x 10^-3 s^-1
Absorbance
time (s)
0.9
0.8
0.7
0.6
0.5
0.4
0
100
200
300
400
500
600
700
800

Results

Type	Rate (s^{-1})	Temperature	Type	Rate (s^{-1})	Temperature
C5	6.48×10^{-3}	24	cell	6.31×10^{-3}	23.6
C5	6.60×10^{-3}	23.8	cell	7.04×10^{-3}	23.6
C5	6.58×10^{-3}	23.7	cell	6.37×10^{-3}	23.6
Average:	**6.56×10^{-3}**	23.8	Average:	**6.57×10^{-3}**	23.6
Standard error:	**3.58×10^{-5}**		Standard error:	**2.33×10^{-4}**	

Reaction of water (N11) with electrophile E3:

Cavity measurements (C5)

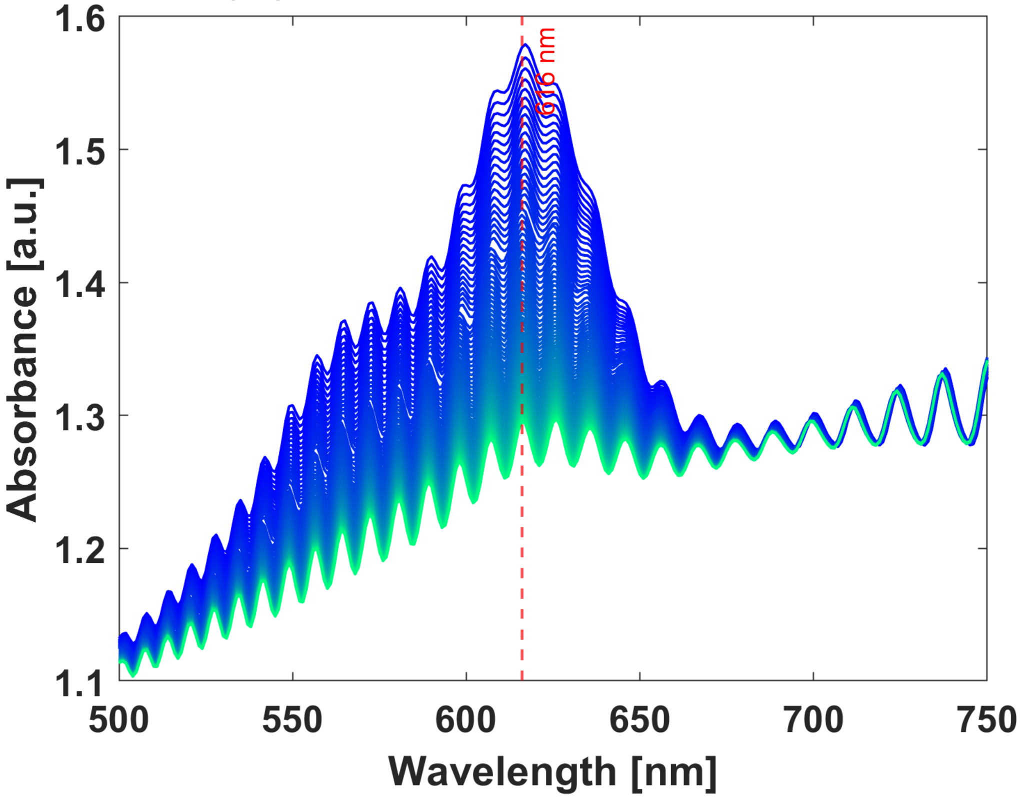

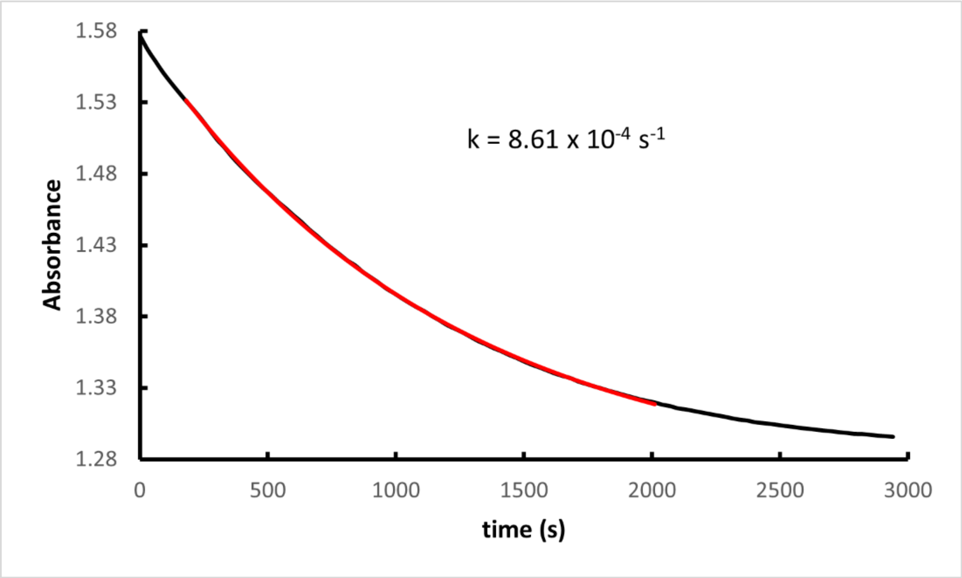

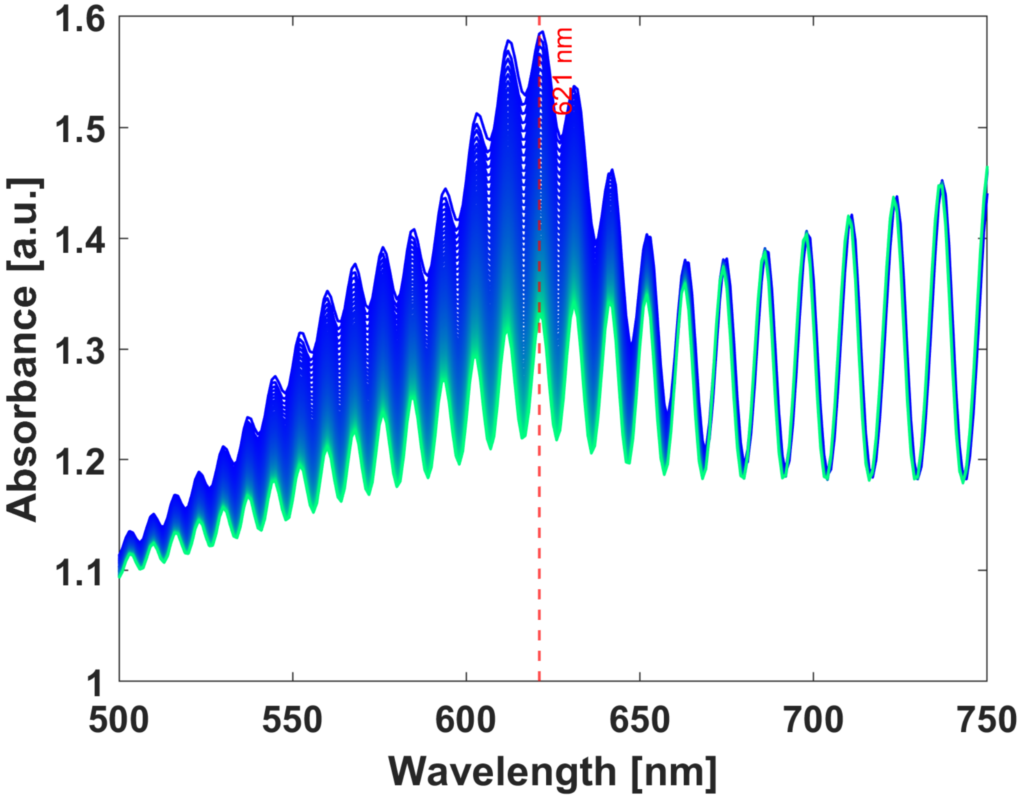

1.6
1.5
1.4
1.3
1.2
1.1
1
Absorbance [a.u.]
500
550
600
650
700
750
Wavelength [nm]
621 nm

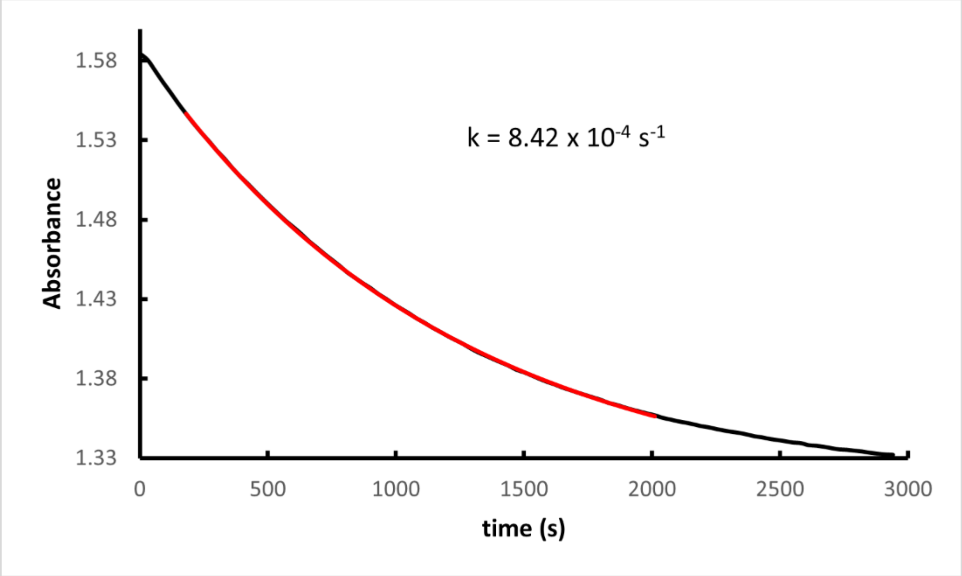

1.58
1.53
1.48
1.43
1.38
1.33
Absorbance
0
500
1000
1500
2000
2500
3000
time (s)
k = 8.42 x 10-4 s-1

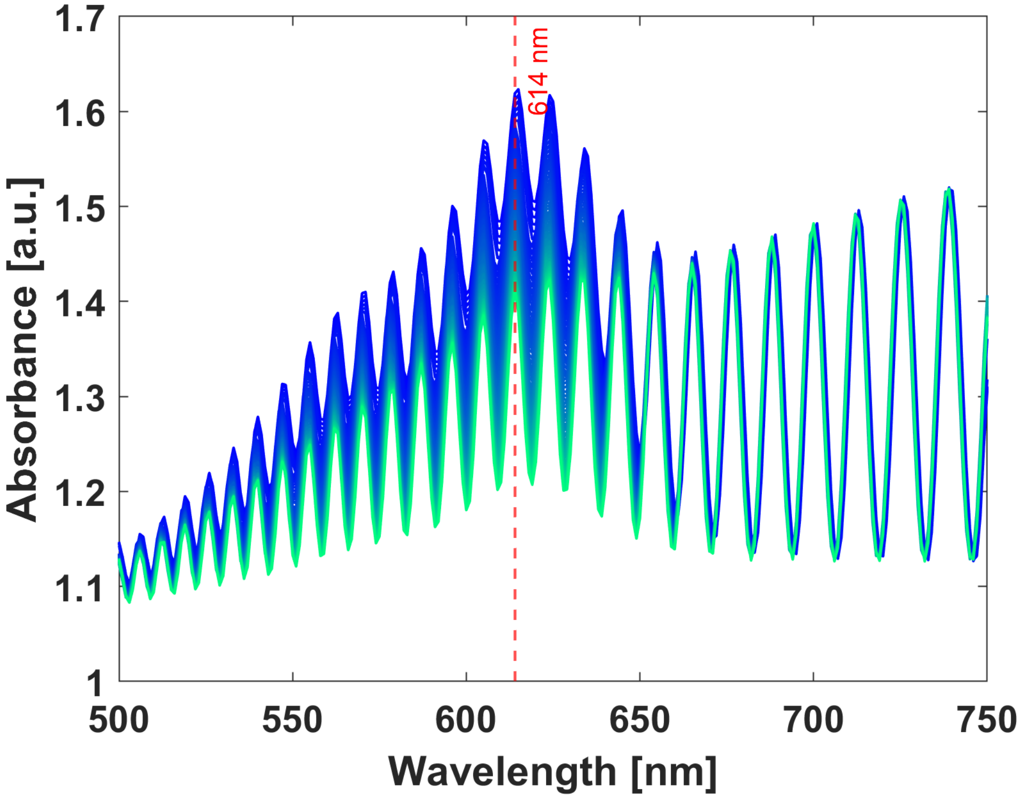
614 nm
Absorbance [a.u.]
Wavelength [nm]
1.7
1.6
1.5
1.4
1.3
1.2
1.1
1
500
550
600
650
700
750

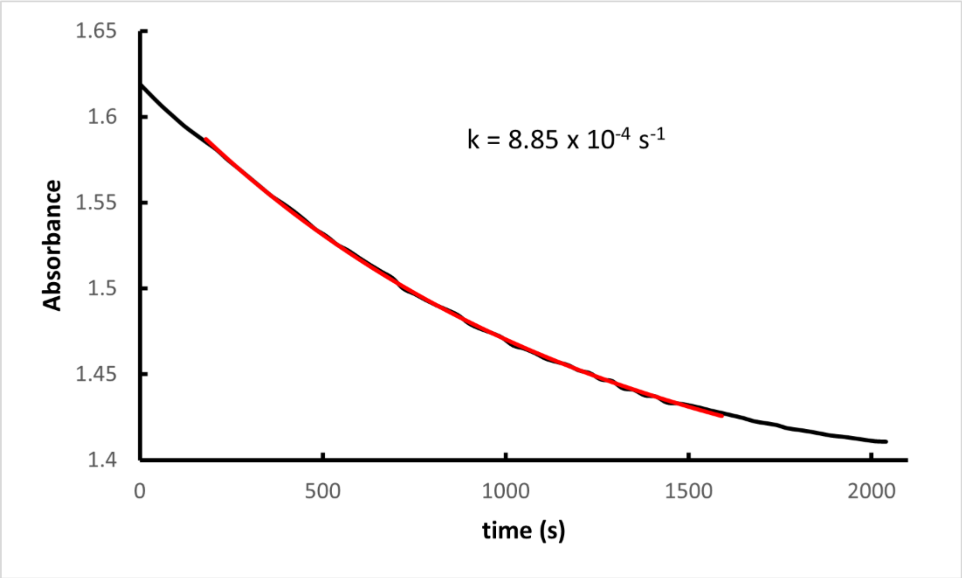
k = 8.85 x 10-4 s-1
Absorbance
time (s)
1.65
1.6
1.55
1.5
1.45
1.4
0
500
1000
1500
2000

Cell measurements (standard)

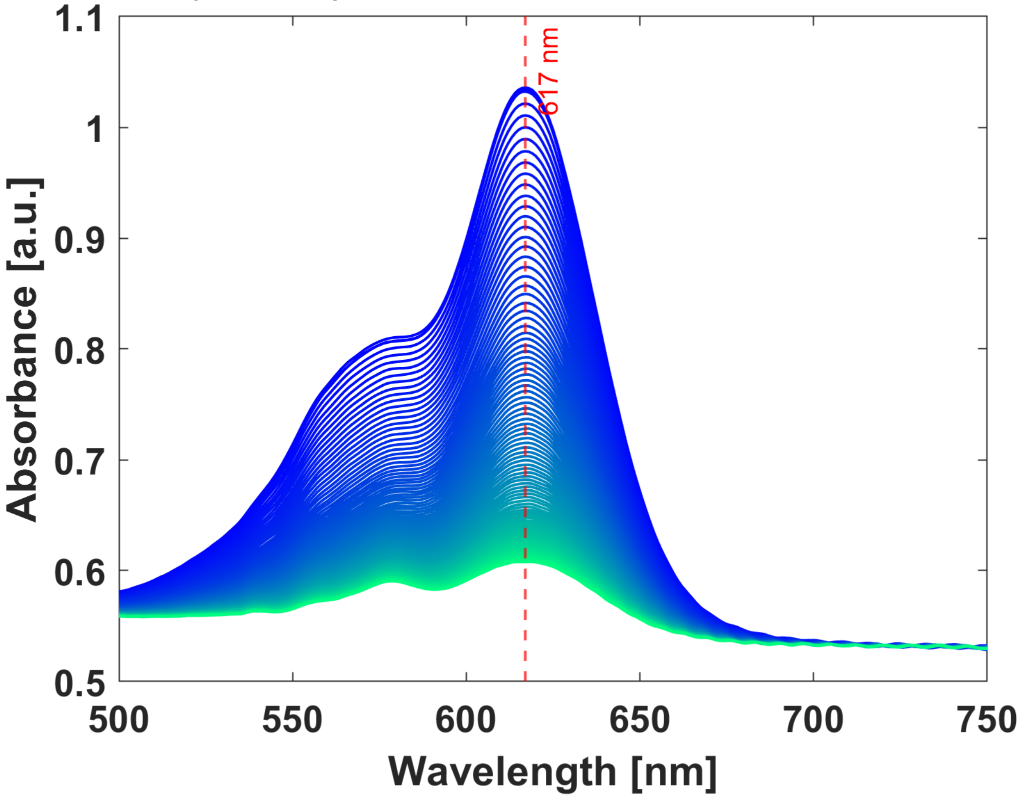

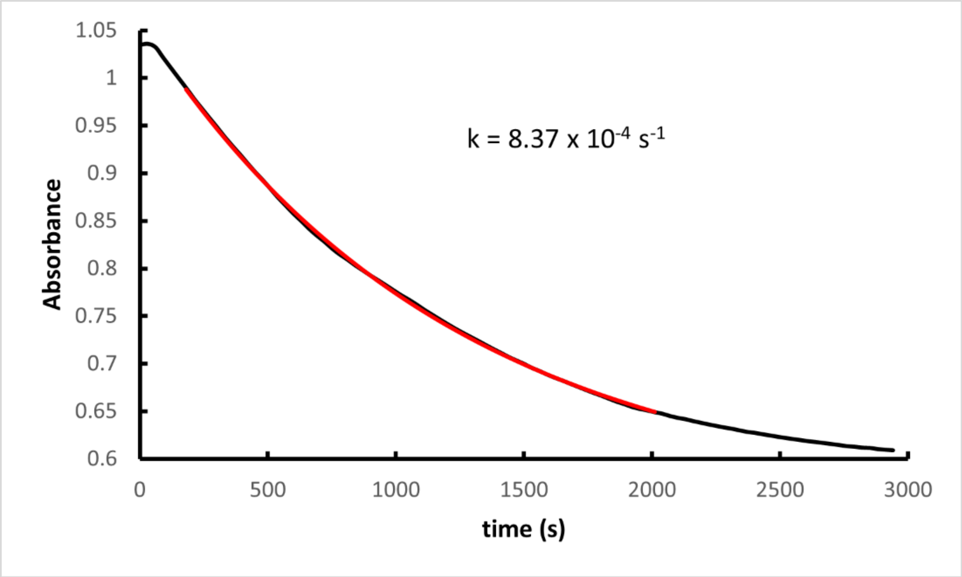

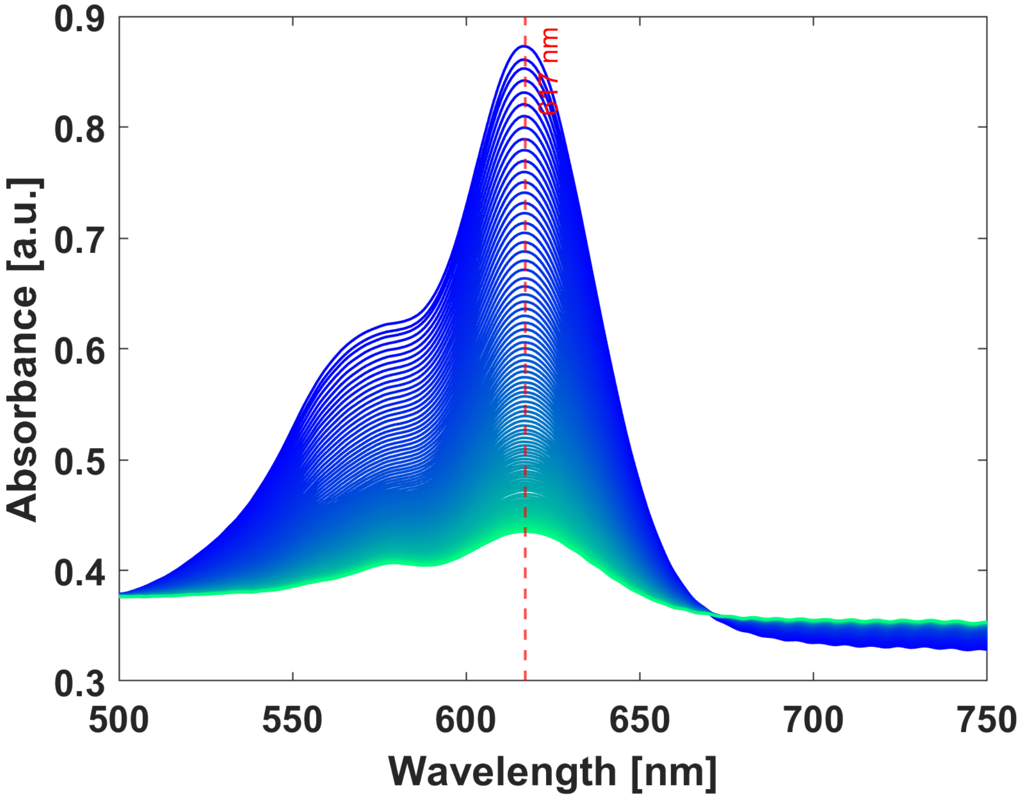

0.9
0.8
0.7
0.6
0.5
0.4
0.3
Absorbance [a.u.]
617 nm
500
550
600
650
700
750
Wavelength [nm]

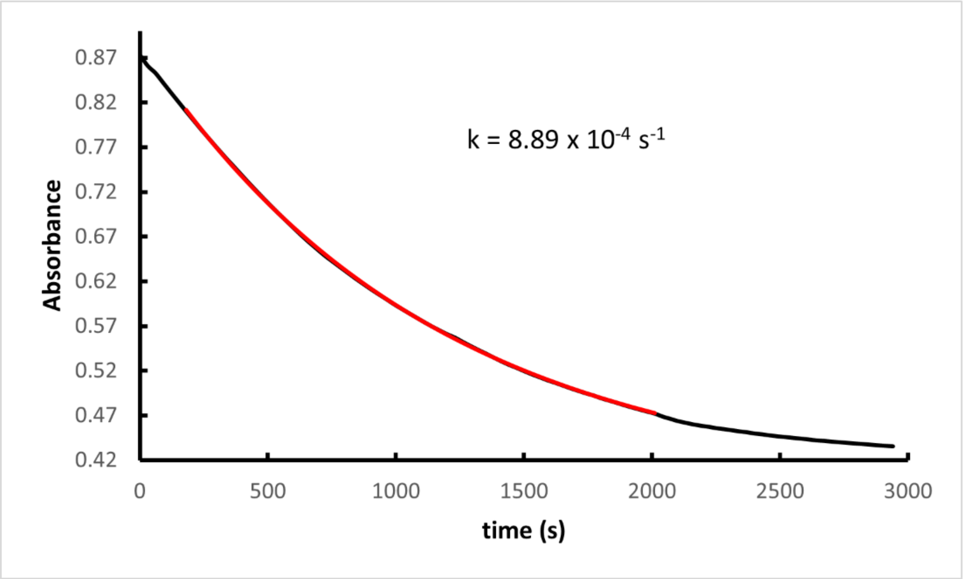

k = 8.89 x 10^{-4} s^{-1}
Absorbance
0.87
0.82
0.77
0.72
0.67
0.62
0.57
0.52
0.47
0.42
0
500
1000
1500
2000
2500
3000
time (s)

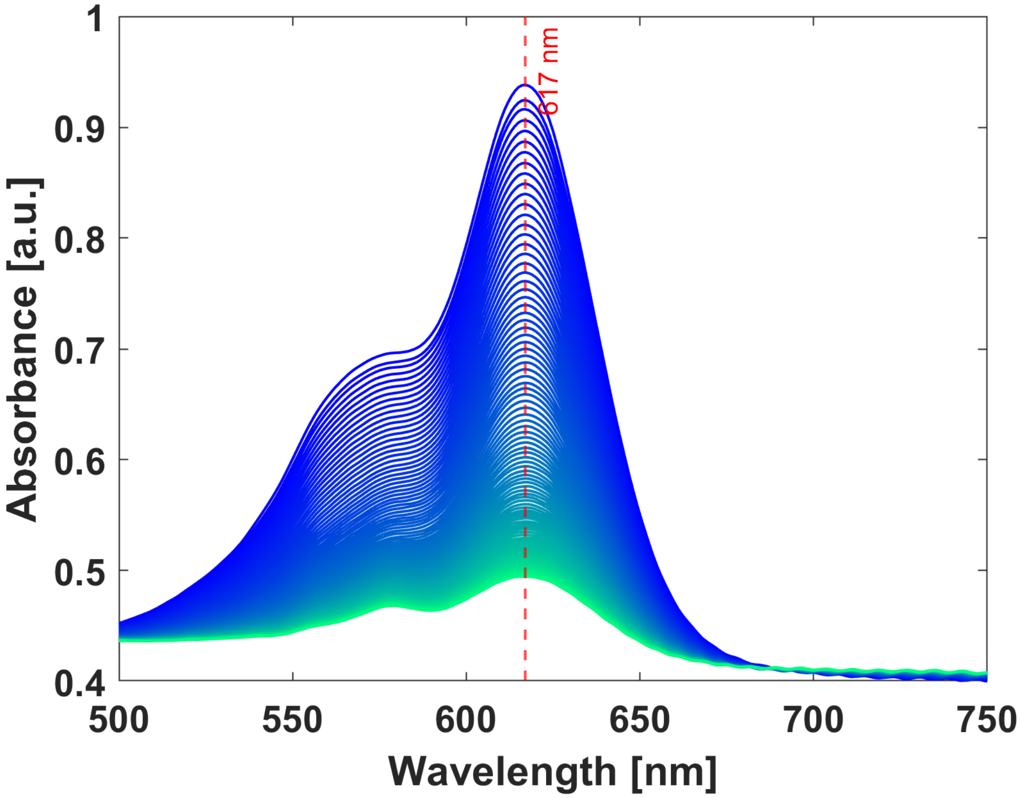

Absorbance [a.u.]
Wavelength [nm]
617 nm
1
0.9
0.8
0.7
0.6
0.5
0.4
500
550
600
650
700
750

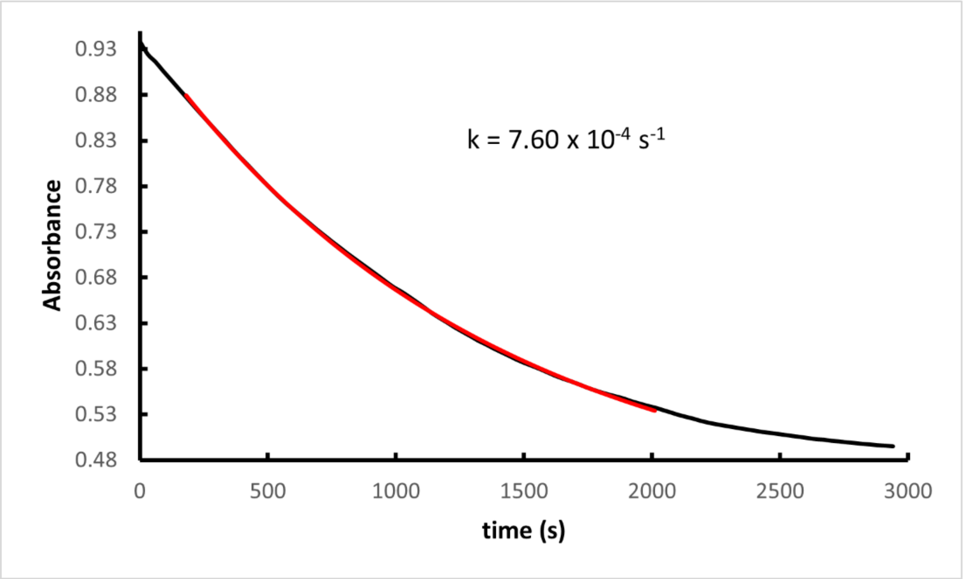

Absorbance
time (s)
k = 7.60 x 10-4 s-1
0.93
0.88
0.83
0.78
0.73
0.68
0.63
0.58
0.53
0.48
0
500
1000
1500
2000
2500
3000

Results

Type	Rate (s^{-1})	Temperature	Type	Rate (s^{-1})	Temperature
C5	$8.61 x 10^{-4}$	23.5	cell	$8.37 x 10^{-4}$	23.2
C5	$8.42 x 10^{-4}$	23.4	cell	$8.89 x 10^{-4}$	23.3
C5	$8.85 x 10^{-4}$	23.3	cell	$7.60 x 10^{-4}$	23.4
Average:	**$8.63 x 10^{-4}$**	23.4	Average:	**$8.29 x 10^{-4}$**	23.3
Standard error:	**$1.23 x 10^{-5}$**		Standard error:	**$3.76 x 10^{-5}$**	

VIII. Other parameters

Temperature

To check whether temperature variations might have resulted in fluctuations in rate constant, we re-plotted k_{obs} hereafter as a function of the temperature at which the kinetic run was measured and found no significant correlation between the two within the range of which temperature fluctuated in our case. The data below correspond to those of the reaction of *n*-butanol (**N3**) with **E1**.

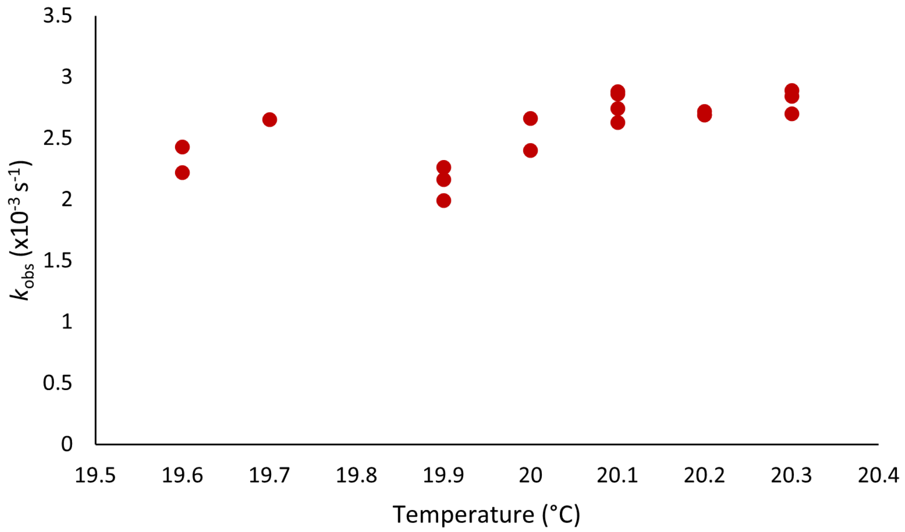

*Figure **S17**: Plot of the* k_{obs} *obtained for the reaction of n-butanol (**N3**) and electrophile **E1** as a function of the temperature at which each experiment was conducted.*

Surface

It could be hypothesized that the small rate changes observed are due to surface effects. In such a case, a trend should be observed between rate and surface/volume ratio, a quantity proportional to the thickness of the cavity. In the following plot, however, we show that there is no correlation between cavity thickness and the rate constant measured in the reaction of **E1** with n-butanol (**N3**).

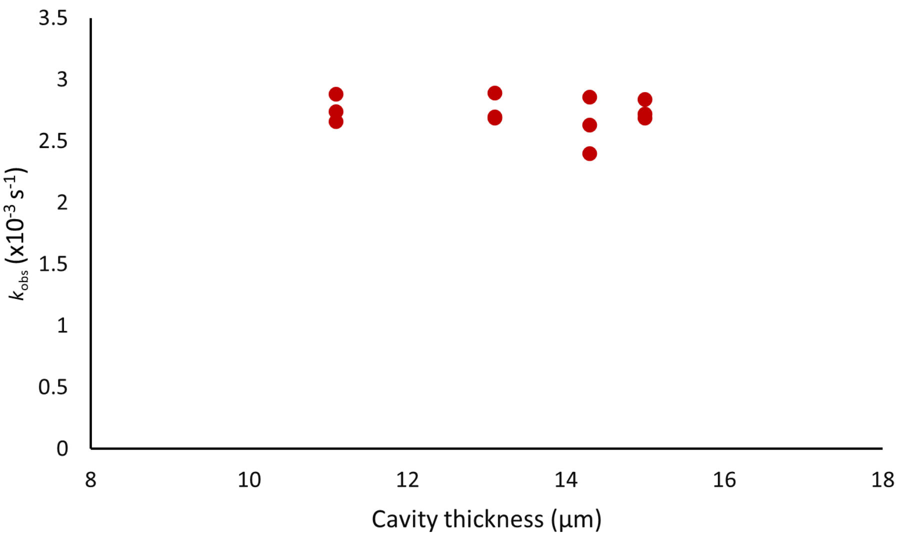

*Figure **S18**: Plot of the* k_{obs} *obtained for the reaction of n-butanol (**N3**) and electrophile **E1** as a function of the thickness of the fixed-width cavity in which each experiment was conducted.*

Linearity of the Spectrometer

As a final control, we looked at how far the domain of linearity extends in the case of our UV-Vis spectrometer (Shimadzu UV-2600i). To perform that control, we weighed 0.25 mg of **E1** into 20 mL of acetonitrile. **E1** was selected owing to its low electrophilicity, which renders it practically inert towards any residual water within acetonitrile. We found that the absorbance increases linearly with concentration (Lambert-Beer law) without any noticeable curvature well up to 3 absorbance units. Accordingly, an absorbance of 1.5, which is commonly reached throughout our study, still lies well within the linearity domain of the instrument.

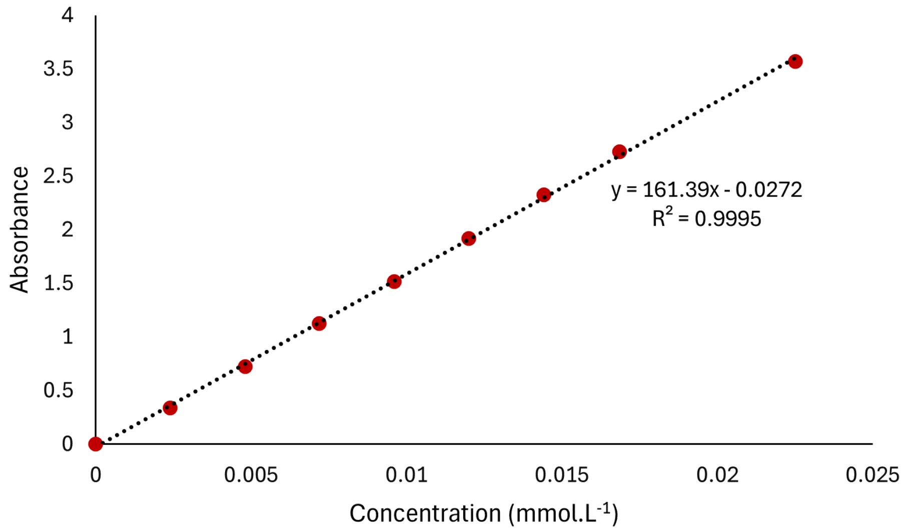

*Figure **S19**: Plot of the absorbance obtained at different concentrations of electrophile **E1** in acetonitrile and linear fitting (dashed line).*

IX. Importance of lutidine

A solution of carbocation **E5** was prepared by dissolving 1.25 mg of the carbocation in 3.75 mL of acetonitrile. Solutions of different concentrations of lutidine in methanol were then prepared. For each run, 0.2 mL of the carbocation solution was dissolved in 1.8 mL of the alcohol solution inside of a 1 cm UV cuvette equipped with a stir bar. The cuvette was then quickly placed inside the chamber of the Jasco V-670 UV-Vis spectrometer with stirring on, and the kinetics were measured by following the disappearance of the blue color.

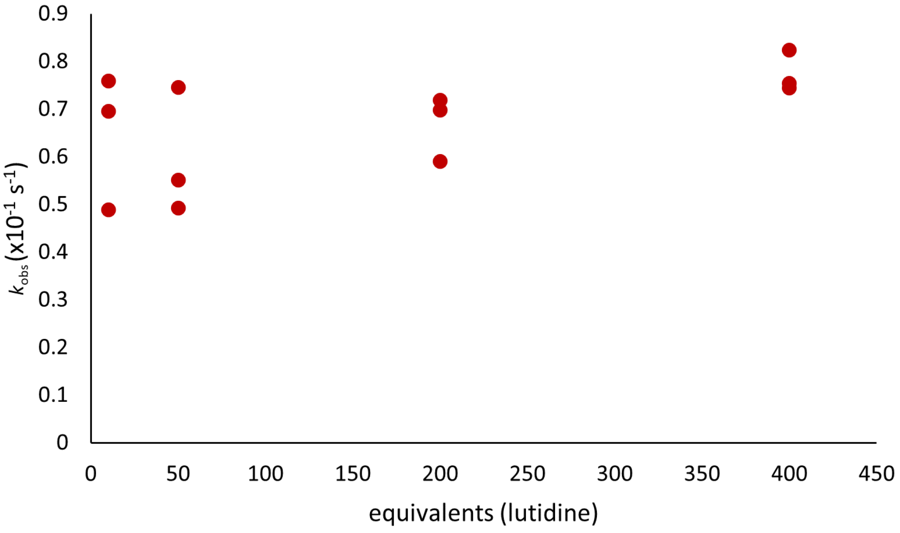

*Figure **S20**: Plot of the* k_{obs} *obtained for the reaction of methanol (**N1**) and electrophile **E5** as a function of the number of equivalents of luditine with which each experiment was conducted.*

Lutidine equivalents	k_{obs} (x 10^{-1} s^{-1})
10	6.47 ± 0.08
50	5.96 ± 0.08
200	6.69 ± 0.04
400	7.73 ± 0.02

***Table S1**: Average* k_{obs} *for the reaction of methanol (N1) with electrophile E5 with different amounts of lutidine.*

X. References

[1] R. J. Mayer, N. Hampel, P. Mayer, A. R. Ofial, H. Mayr, *Eur. J. Org. Chem.* **2019**, *2019*, 412–421.

[2] B. Patrahau, M. Piejko, R. J. Mayer, C. Antheaume, T. Sangchai, G. Ragazzon, A. Jayachandran, E. Devaux, C. Genet, J. Moran, T. W. Ebbesen, *Angew. Chem. Int. Ed.* **2024**, e202401368.

[3] B. Simpkins, M. Michon, **2023**, DOI 10.26434/chemrxiv-2023-c3cqb.

[4] M. Michon, B. Simpkins, **2024**, DOI 10.26434/chemrxiv-2024-880ph.

[5] A. Thomas, J. George, A. Shalabney, M. Dryzhakov, S. J. Varma, J. Moran, T. Chervy, X. Zhong, E. Devaux, C. Genet, J. A. Hutchison, T. W. Ebbesen, *Angew. Chem. Int. Ed.* **2016**, *55*, 11462–11466.
